# Supplementary material for: Characterization of drug-induced transcriptional modules: towards drug repositioning and functional understanding
Source: Mol Syst Biol. 2013 Apr 30;9:662. doi: 10.1038/msb.2013.20 (PMC3658274; doi:10.1038/msb.2013.20)
Supplement: Supplementary Information — Supplementary figures 1–10, Supplementary tables 1, 2, 4, 5 and 6 [file msb201320-s1.pdf]

Supplementary Information:

Characterization of drug-induced transcriptional modules: towards drug repositioning and functional understanding

Murat Iskar, Georg Zeller, Peter Blattmann, Monica Campillos, Michael Kuhn, Kasia Kaminska, Heiko Runz, Anne-Claude Gavin, Rainer Pepperkok, Vera van Noort, Peer Bork*

*Correspondence to: bork@embl.de

Table of Contents

Supplementary Figure 1. Properties and preprocessing of input datasets 3

Supplementary Figure 2. Non-redundant drug-induced modules can be detected across a wide range of ISA parameter settings 4

Supplementary Figure 3. Network of conserved drug-induced transcriptional modules 5

Supplementary Figure 4. Module conservation across model systems is significantly higher than expected for random independent biclusters 6

Supplementary Figure 5. Assessing the impact of drug space on module conservation........ 7

Supplementary Figure 6. Functional coherence of transcriptional modules as benchmarked against STRING 8

Supplementary Figure 7. CODIM2 is enriched in genes known to function in unfolded protein response (UPR) and cholesterol biosynthesis pathways 9

Supplementary Figure 8. Negative controls for assessing anti-proliferative effects of CODIM1 predictions 10

Supplementary Figure 9. Cell cycle analysis using propidium iodide staining and FACS. and assessing FL3-A signal to determine cell cycle phases of cells 11

Supplementary Figure 10. Detailed results of experimentally tested drug-target interactions 12

Supplementary Table 1. Robustness of drug-induced modules to redundancy removal parameters.. 13

Supplementary Table 2. Robustness of constitutive co-expression modules to detection parameter 14

Supplementary Table 3. Comparison of gene and drug members of drug-induced modules across human cell lines and organisms...**Separate File**

Supplementary Table 4. Characterization of gene and drug members of conserved drug-induced modules (CODIMs), CODIM-associated modules of rat liver and selected cell line-specific modules linked to a certain MOA **15**
Supplementary Table 5. Detailed information on the characterization of gene and drug members of unified conserved drug-induced modules (CODIMs), CODIM-associated modules of rat liver and selected cell line-specific modules linked to a certain MOA **17**
Supplementary Table 6. Experimental results of RNAi-screen for selected genes from CODIM2 .... **60**
Supplementary Dataset 1 .... **Separate File**

Supplementary Figures

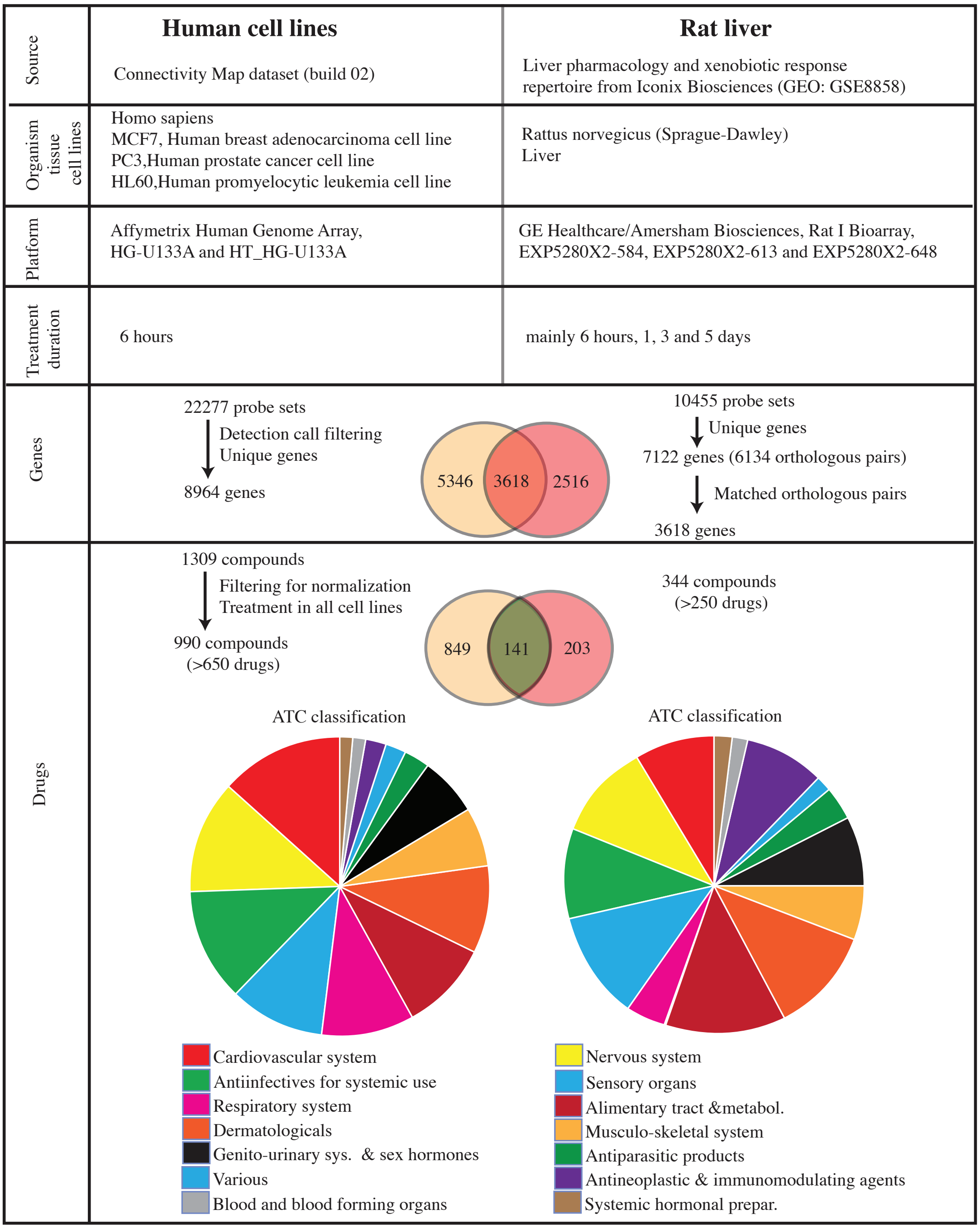

Supplementary Figure 1. Properties and preprocessing of input datasets. Row labeled 'Genes' contains the results of redundancy removal and orthology mapping between probe sets and charts the overlap as Venn diagram in the middle. Row labeled 'Drugs' charts the result of restricting the CMap data set to compounds tested in all three cell lines used here and how these overlap with compounds tested in rat liver (Venn diagram in the middle). Pie charts give the composition of the drug space profiled in each resource.

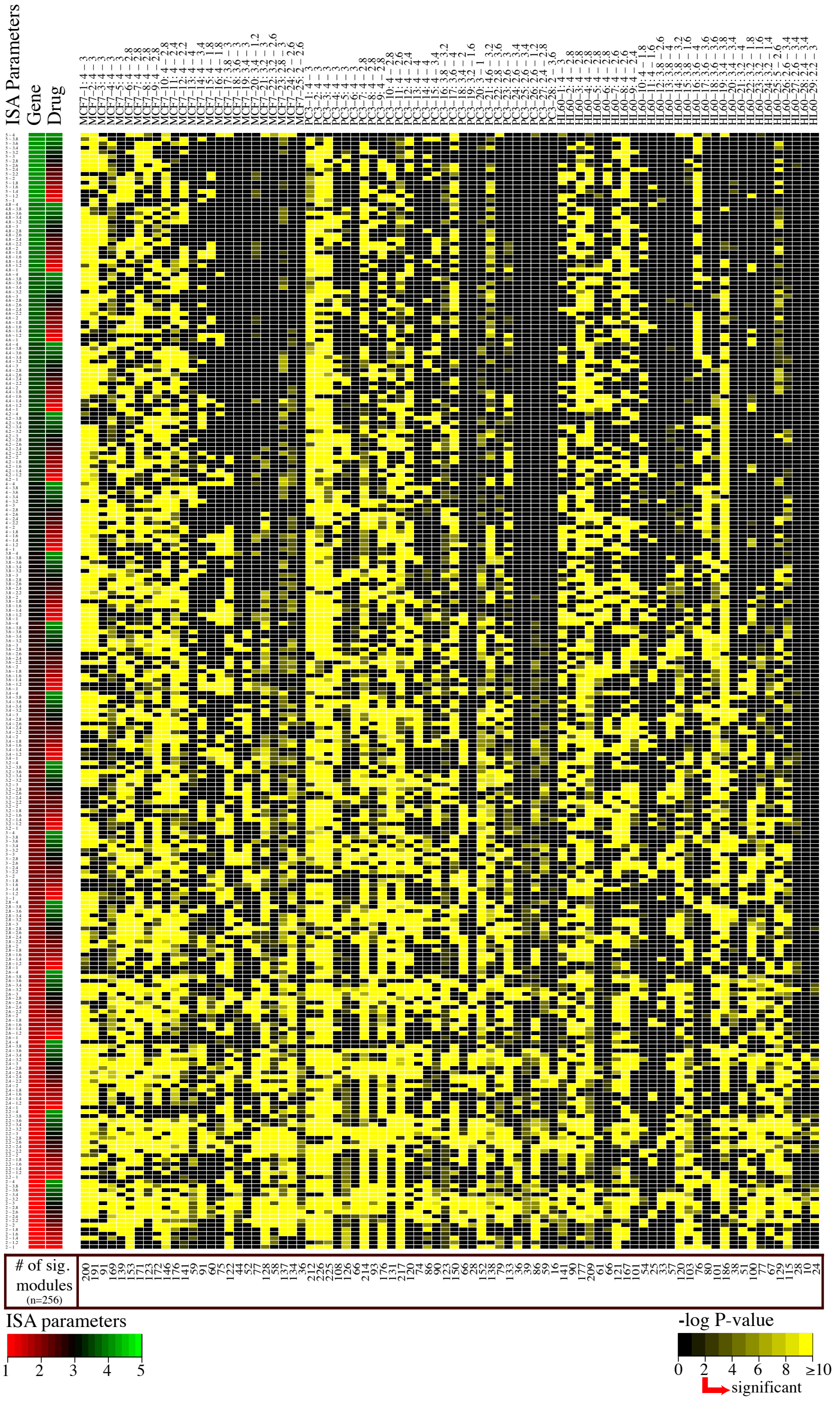

Supplementary Figure 2. Non-redundant drug-induced modules can be detected across a wide range of ISA parameter settings. The non-redundant final set of drug-induced modules from each cell line was compared to the whole set of raw drug-induced modules before redundancy removal (MCF7 n=2255, PC3 n=1661, HL60 n=1783) that were identified under a number of runs of the ISA algorithm that explored an extensive parameter grid (gene s.d. cutoff [from 5 to 2 in steps of 0.2], drug s.d. cutoff [from 4 to 1 in steps of 0.2]). The heatmap indicates the significance of gene overlap (Fisher's exact test, negative log10 of the p-value, see color key) between any of the final non-redundant modules (columns) and the most similar module from a particular ISA parameter set (rows). In total, more than half of the final modules significantly overlapped (FDR-corrected p-value<0.01) with raw modules from more than 100 distinct parameter settings. A high-resolution version is available at http://www.bork.embl.de/Docu/codim/supp-files/Supp_Fig_2.pdf.

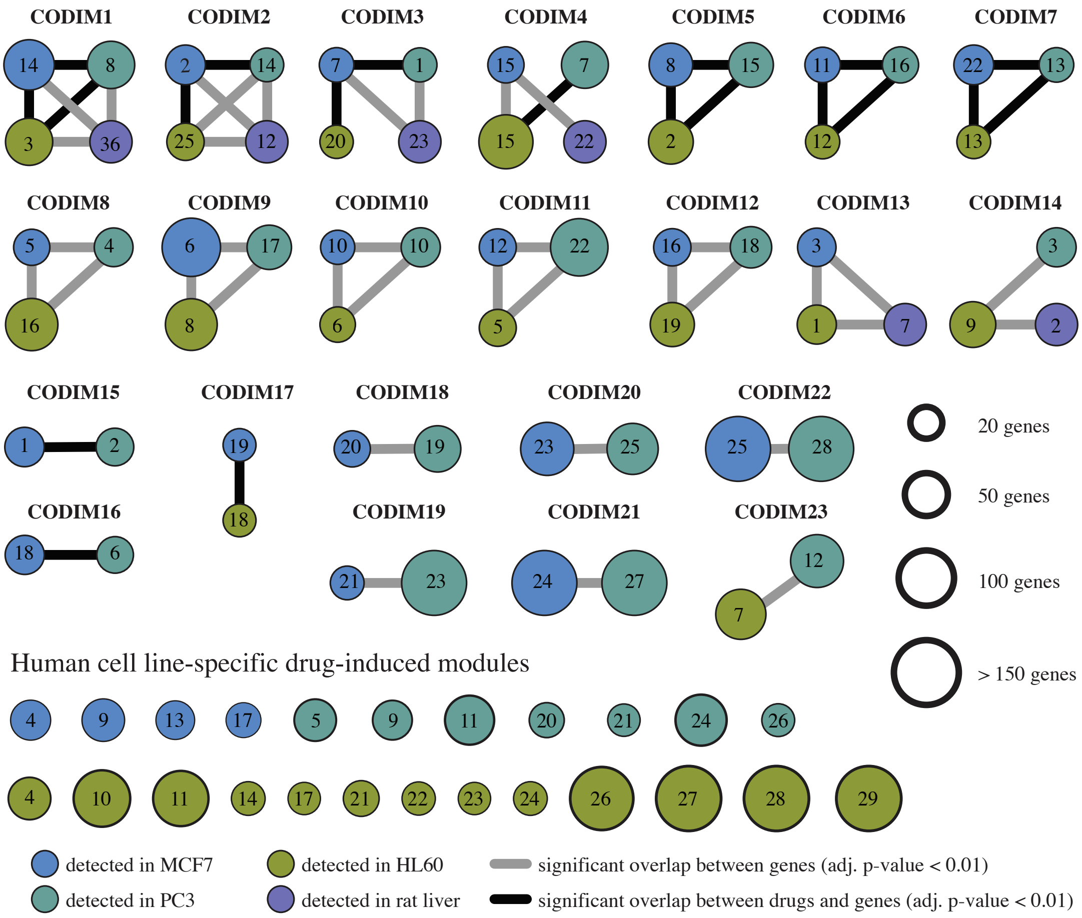

Supplementary Figure 3. Network of conserved drug-induced transcriptional modules (CODIM). Using a reciprocal best hit approach, drug-induced transcriptional modules across three cell lines and rat liver were linked to each other based on the significance of the overlap between their gene and drug members (with FDR corrected p-value < 0.01). Taken together, these modules covered a substantial fraction of the gene and drug space of the underlying expression data: on average 1,857 genes (20% of the input gene set) and 555 drugs (56%) per cell line, and for rat liver 1,587 genes (44%) and 311 drugs (90%) were represented within modules. All drug-induced transcriptional modules were labeled (number) in the order in which they were identified by the ISA procedure.

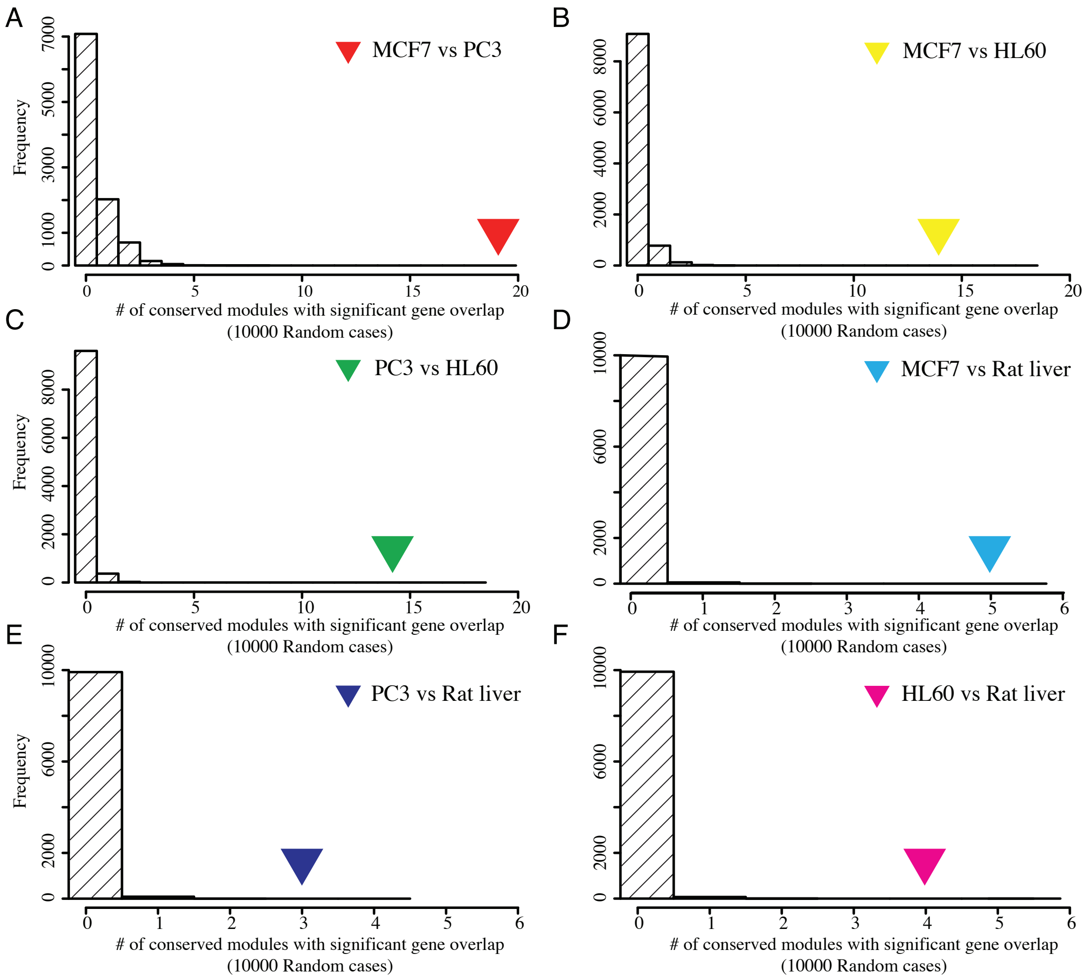

Supplementary Figure 4. Module conservation across model systems is significantly higher than expected for random independent biclusters. For each pair-wise comparison of 4 datasets (3 cancer cell lines and rat liver), the number of conserved modules was compared against the conservation between biclusters that were randomly generated, independently for each data set. Properties of 10,000 randomly generated biclusters resembled those of real ones (same distributions of gene members). In each pair-wise comparison between data sets, we observed significantly more conserved real drug-induced modules as assessed by their gene overlap (empirical p-value < 1E-4) than between independent randomly generated data sets.

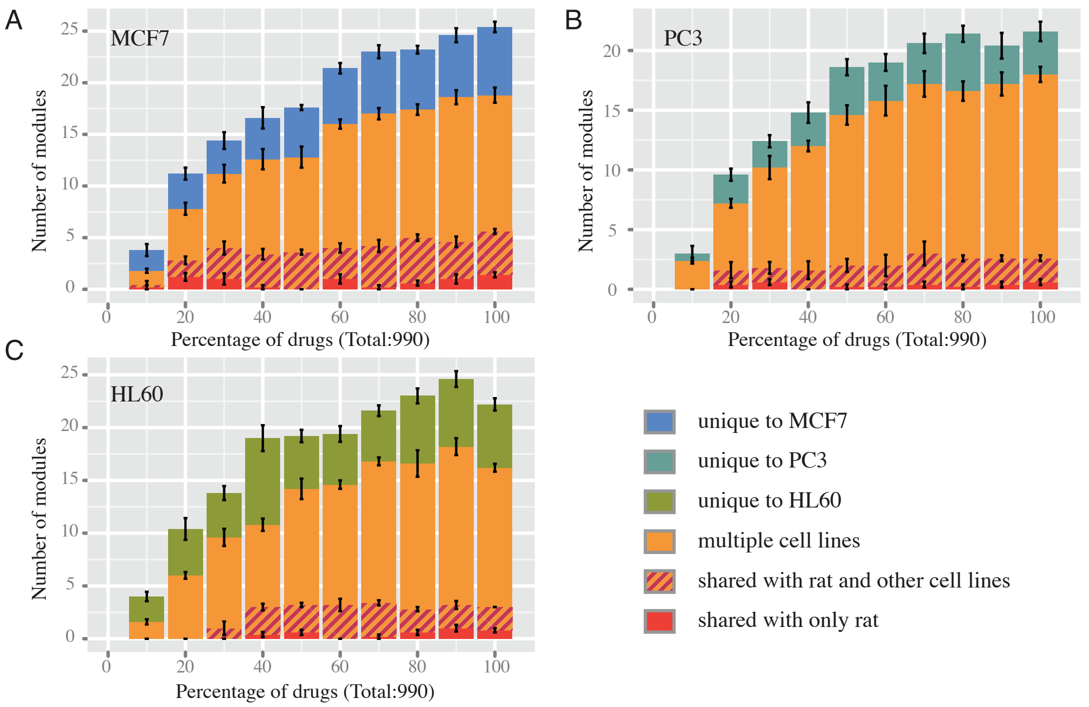

Supplementary Figure 5. Assessing the impact of drug space on module conservation. The total number of treatments (990 drugs) in the CMap dataset were downsampled to smaller drug spaces comprising 10% to 100% (raw data) in increments of 10% (always using the same drug sample for all three cell lines). Five replicates were analyzed for each downsampled drug space. Drug-induced transcriptional modules were identified using exactly the same pipeline and parameters except for the number of seeds, which were reduced to 2,000 to reduce computation time. The total number of identified drug-induce modules and their conservation across cell lines and rat liver are displayed as stacked bar plots with error bars representing the standard error. These results indicate that a drug space of 200-400 drugs (~20-40%, assessed in three cell lines) appears to be a lower limit for robust conservation estimation.

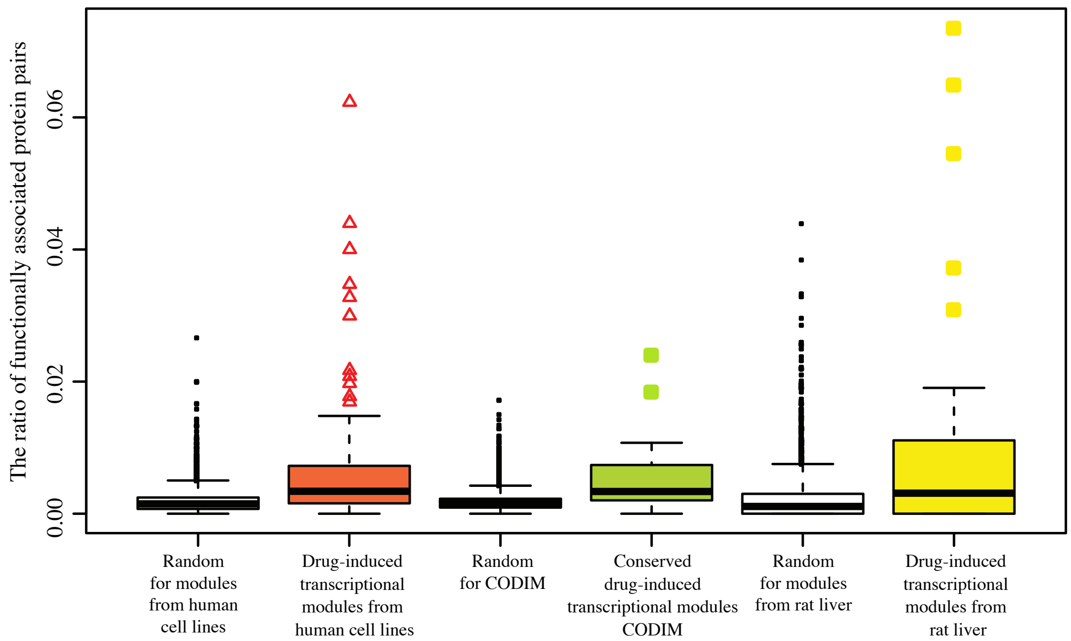

Supplementary Figure 6. Functional coherence of transcriptional modules as benchmarked against STRING. Functionally related protein pairs were extracted from the STRING protein-protein association network for human and rat. Drug-induced transcriptional modules from human cell lines and rat liver were found to have a higher ratio of functionally associated gene pairs in comparison to random modules (1,000 gene sets with the same size and background). 30 out of 82 modules from human cell lines (35%), 12 out of 23 CODIMs (52%) and 13 out of 43 modules from rat liver (30%) clustered together more functionally related genes than expected by chance (at an empirical, permutation-based p-value cutoff < 0.05).

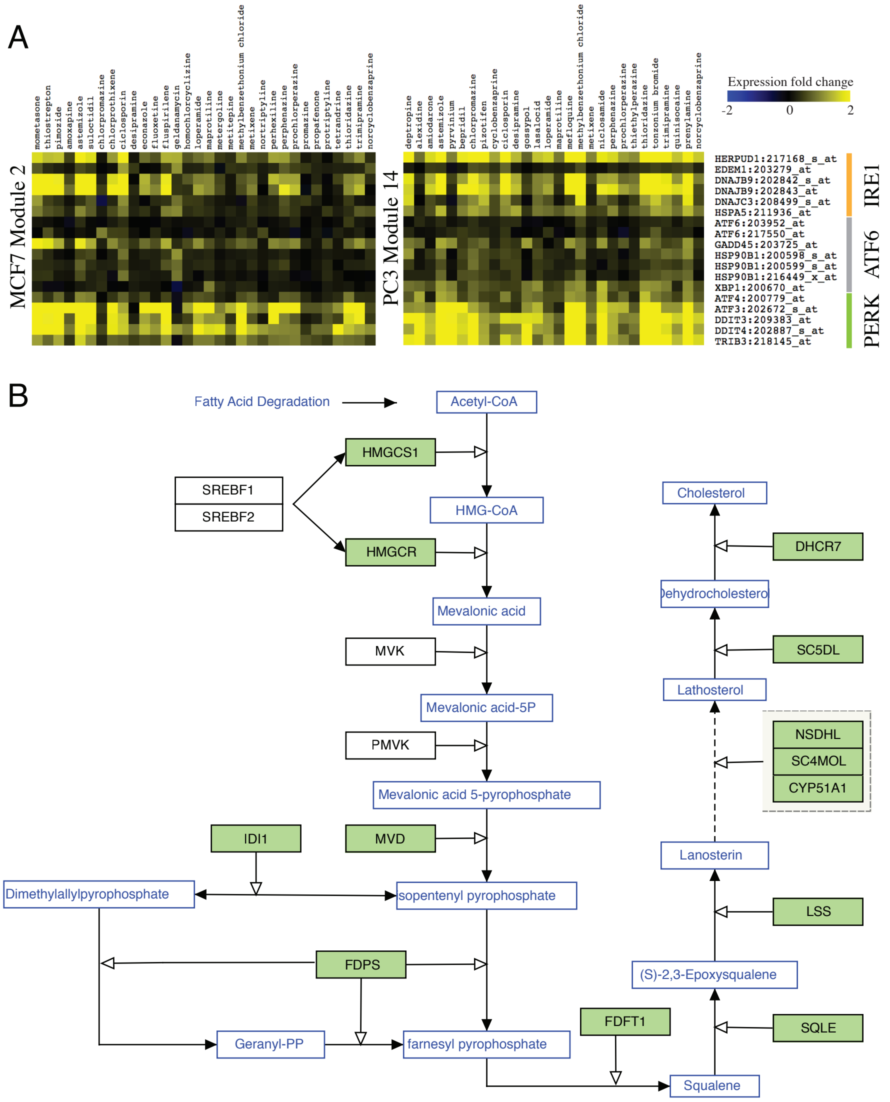

Supplementary Figure 7. CODIM2 is enriched in genes known to function in unfolded protein response (UPR) and cholesterol biosynthesis pathways. (A) The modulatory effect of CODIM2 on three branches of UPR signaling. Shown are genes known to be modulated by one of the key UPR sensors IRE-1, ATF6, PERK and how their expression changes in response to the drugs contained in CODIM2 (enriched for antipsychotics). Overall mean fold changes ±SEM in PC3 module 14, n=28, are in the following indicated in parentheses: for IRE1 targets HERPUD1 (1.75±0.11), EDEM1 (0.32±0.04), DNAJB9 (1.68±0.18), DNAJC3 (0.75±0.11), and HSPA5 (0.89±0.07); for the ATF6 branch ATF6 (0.2±0.03), GADD45 (0.68±0.07), HSP90B1 (0.48±0.06) and XBP1 (0.79±0.06); for PERK targets ATF4 (0.82±0.07), ATF3 (1.66±0.24), DDIT3 (1.92±0.14), DDIT4 (1.92±0.21), and TRIB3 (1.75±0.13) (for genes with multiple probe sets, the one with highest mean FC was selected). In contrary to reported effects of antipsychotics specifically on PERK signaling (Canfran-Duque *et al*, 2012), CODIM2 drugs were found to stimulate all branches of UPR signaling although to a lower extent in the ATF6 branch. Target genes were extracted from the literature (Canfran-Duque *et al*, 2012; Lee *et al*, 2003; Szegezdi *et al*, 2006). (IRE1, inositol required 1; PERK, PKR-like endoplasmic reticulum localized kinase; ATF6, activating transcription factor 6) **(B)** The curated pathway of cholesterol biosynthesis as illustrated in WikiPathways (Kelder *et al*, 2012). Genes in this pathway that were also contained in CODIM2 are labeled in green.

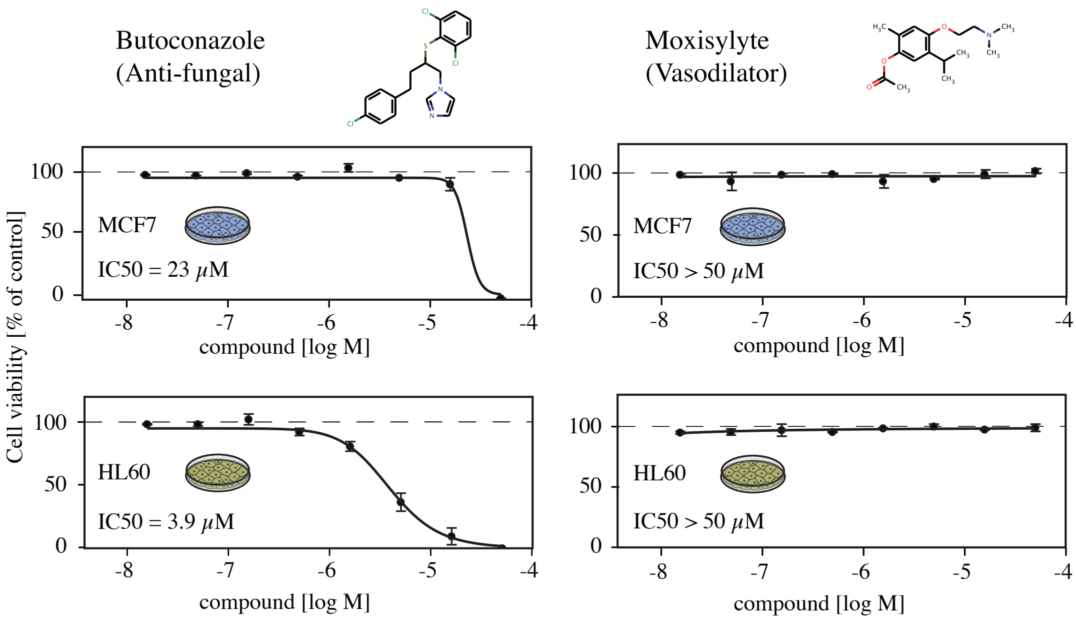

Supplementary Figure 8. Negative controls for assessing anti-proliferative effects of CODIM1 predictions. Three predicted candidate cell-cycle inhibitors were chosen for validation experiments: sulconazole (anti-fungal) and vinburnine (vasodilator) were successfully validated, whereas validation of mephentermine (cardiac stimulant) failed (see Fig. 3 in the main text). Butoconazole and moxisylate (both present in CMap) were chosen as negative controls for validation experiments with results shown here.

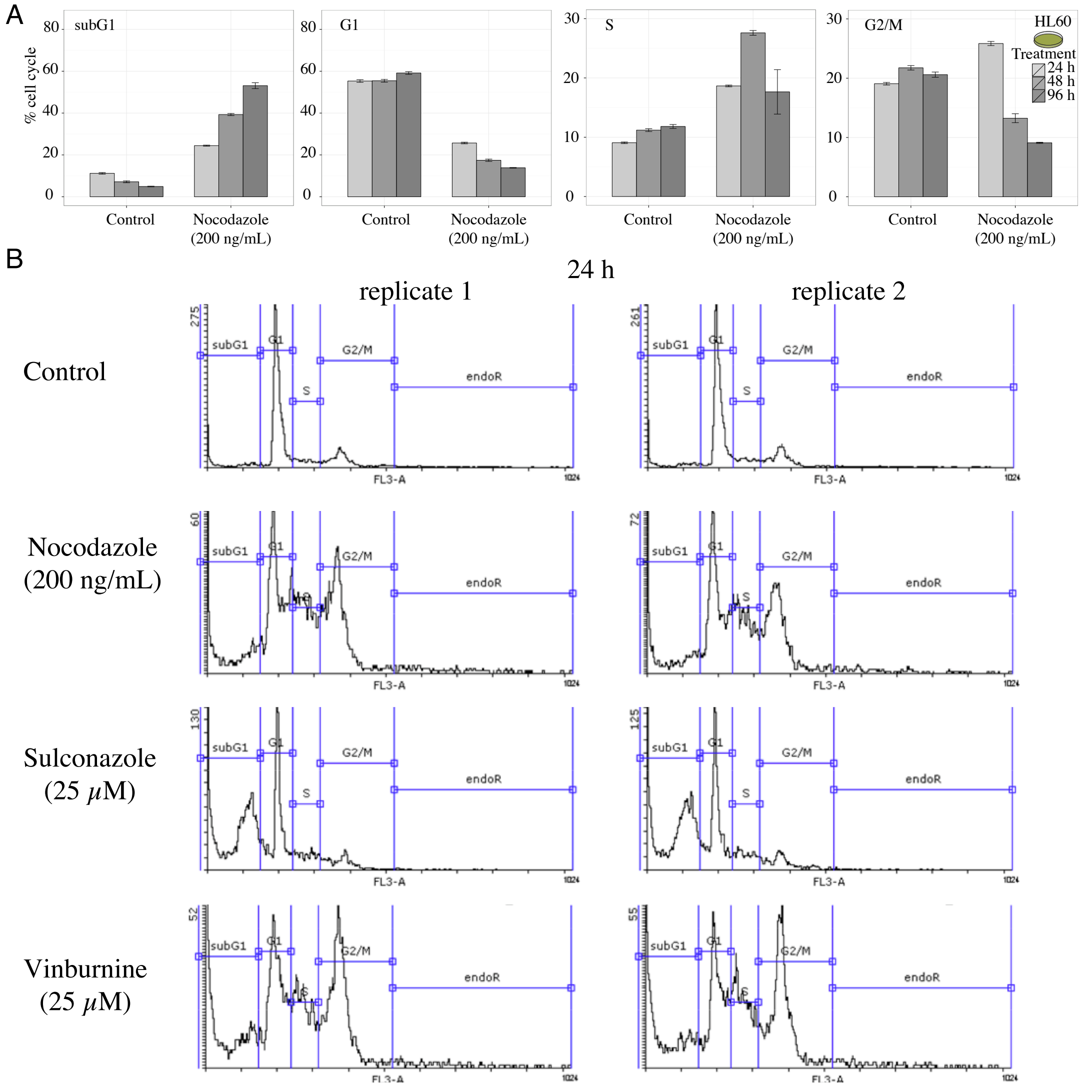

Supplementary Figure 9. Cell cycle analysis using propidium iodide staining and FACS and assessing FL3-A signal to determine cell cycle phases of cells. (A) Treatment of HL-60 cells with reference compound nocodazole (200 ng/mL) resulted in G2/M arrest, most prominent after 24h of treatment. **(B)** Using "Flowing software", the FL3-A signal indicating DNA content was used to determine cells in different cell cycle phases, subG1, G1, G2/M and endoR (endoreduplicated) (see Materials and Methods for details).

Reference Module	Tested Drug	Target	Binding Assays Activity (50μM)	K_i(μM)	nh	Cellular Assays Antag./Inhibitor	Agonist
PC3-9	Zaprinast	PPARγ	**57**	**14**	1.0		
PC3-9	Raubasine	PPARγ	<20				
MCF7-9	Nitrendipine	ER alpha	**49**	**46**	1.9		
MCF7-9	Theobromine	ER alpha	<20				
MCF7-9	Bendroflumethiazide	ER alpha	<20				
MCF7-9	Dilazep	ER alpha	*Assay not available*			26.3	
HL60-17	Hexetidine	ADRA2C	**59**	**6.5**	1.0		
HL60-17	(+)-chelidonine	ADRA2C	**54**	**15**	0.6		
HL60-17	Vigabatrin	ADRA2C	<20				
HL60-17	Podophyllotoxin	ADRA2C	<20				
HL60-17	Hexetidine	ADRA1B	*Assay not available*				<20
HL60-17	(+)-chelidonine	ADRA1B	*Assay not available*				<20
HL60-17	Vigabatrin	ADRA1B	*Assay not available*				<20
HL60-17	Podophyllotoxin	ADRA1B	*Assay not available*				<20

Supplementary Figure 10. Detailed results of experimentally tested drug-target interactions. Ten newly predicted drug-target interactions were experimentally evaluated with *in vitro* binding or cellular assays as indicated. Two drugs from the PC3-9 module, zaprinast (phosphodiesterase inhibitor) and raubasine (anti-hypertensive) were tested for PPARγ binding. Four drug repositioning candidates from MCF7-9 module were screened for binding affinity to estrogen receptor alpha (nitrendipine, antihypertensive; theobromine, alkaloid of the cacao plant, present at high levels in chocolate; bendroflumethiazide, thiazide diuretic) or an antagonistic effect in a cellular assay (dilazep, vasodilator). Predictions based on the HL60-17 module (hexetidine, anti-bacterial; (+)-chelidonine, alkaloid; vigabatrin, antiepileptic; podophyllotoxin, antiviral) were both tested against ADRA2C and ADRA1B, as it was not possible to *a priori* determine the exact target associated with the drugs from the HL60-17 module from cell line data. K_i values were obtained for predictions with 40% and more activity at 50 μM concentration (nh: Hill coefficient).

Supplementary Tables

Supplementary Table 1. Robustness of drug-induced modules to redundancy removal parameters. Redundancies among modules were eliminated within each cell line using the recommended standard procedure ISAUnique (R 'eisa' package). We used a correlation (cor.) threshold of 0.3. Subsequently, modules were subjected to a second round of redundancy removal based on gene overlap between modules (Fisher test, p-value <10^{-5}, using the same priority as pre-defined by ISA). Additionally, we explored a wide range of parameters here (cor. threshold: from 1 to 0.1 in decrements of 0.1 and overlap p-value cutoffs 10^{-2}, 10^{-5}, and 10^{-10}) and assessed the effect on the resulting non-redundant set of modules (percentages in parentheses indicate the coverage of the non-redundant set of modules described in the main text). In conclusion, the combination of these redundancy removal routines yields similar results across a broad range of parameter settings and is thus quite robust to the actual parameters used (shaded in gray).

ISAUnique (cor.) threshold	# of modules after ISAUnique	# of non-redundant modules after removal of (gene) overlapping modules [% overlap with final set]<br>Threshold for Fisher test P value (FDR corr.)		
**MCF7**		10^{-2}	10^{-5}	10^{-10}
1.0	1909	28 [84%]	31 [96%]	35 [92%]
0.9	740	28 [84%]	31 [96%]	35 [92%]
0.8	246	26 [84%]	31 [96%]	35 [92%]
0.7	119	25 [84%]	31 [96%]	34 [92%]
0.6	96	25 [84%]	31 [96%]	33 [92%]
0.5	69	25 [84%]	31 [96%]	33 [92%]
0.4	47	24 [84%]	28 [96%]	31 [96%]
0.3	35	23 [88%]	25 [100%]	29 [96%]
0.2	29	21 [80%]	23 [92%]	27 [92%]
0.1	22	17 [68%]	20 [80%]	22 [80%]
**PC3**		10^{-2}	10^{-5}	10^{-10}
1.0	1405	24 [68%]	30 [100%]	34 [100%]
0.9	447	25 [71%]	30 [100%]	34 [100%]
0.8	157	24 [71%]	30 [100%]	33 [100%]
0.7	86	24 [71%]	30 [100%]	33 [100%]
0.6	67	23 [71%]	30 [100%]	33 [100%]
0.5	60	23 [71%]	30 [100%]	33 [100%]
0.4	50	22 [75%]	28 [100%]	31 [100%]
0.3	38	22 [75%]	28 [100%]	30 [100%]
0.2	32	21 [71%]	27 [96%]	29 [96%]
0.1	25	19 [68%]	24 [86%]	25 [86%]
**HL60**		10^{-2}	10^{-5}	10^{-10}
1.0	1472	28 [76%]	33 [97%]	39 [100%]
0.9	492	28 [76%]	33 [97%]	39 [100%]
0.8	193	28 [76%]	33 [97%]	39 [100%]
0.7	106	28 [79%]	33 [97%]	39 [100%]
0.6	94	27 [79%]	32 [97%]	37 [100%]
0.5	78	26 [86%]	31 [100%]	36 [100%]
0.4	62	26 [86%]	30 [100%]	36 [100%]
0.3	42	25 [86%]	29 [100%]	35 [100%]
0.2	34	25 [86%]	29 [100%]	33 [100%]
0.1	24	22 [76%]	23 [79%]	24 [79%]

Supplementary Table 2. Robustness of constitutive co-expression modules to detection parameter. To obtain a final set of drug-induced modules, we discarded those 'constitutive' co-expression modules in which 10% or more of the gene pairs were correlated (Pearson coefficient > 0.6) in untreated control samples (available at http://www.bork.embl.de/Docu/codim/supp-files/drug-induced-expression-profiles.tar.gz). Here we additionally explored a wider parameter grid by varying the percentage of correlated gene pairs between 1 and 50% (see Table). When we assessed how many of the non-redundant modules filtered with these parameters overlapped with the final modules presented in the main text, we found that for any cutoff, the majority of final drug-induced modules (in most cases the vast majority) were detected invariably.

Threshold for % cor. gene pairs	Drug-induced modules		Constitutive co-expression (background) modules	
	# of redundant modules	# of non-redundant modules [% of overlap with final set]	# of redundant modules	# of non-redundant modules
**MCF7**				
1	1464	21 [80%]	573	9
5	1822	26 [92%]	215	6
10	1909	25 [100%]	128	4
20	1957	26 [84%]	80	3
50	2014	26 [88%]	23	1
**PC3**				
1	1020	21 [61%]	667	15
5	1280	27 [71%]	407	11
10	1405	28 [100%]	282	9
20	1529	29 [75%]	158	8
50	1627	29 [79%]	60	3
**HL60**				
1	969	27 [69%]	730	15
5	1349	29 [90%]	350	7
10	1472	29 [100%]	227	5
20	1614	29 [90%]	85	3
50	1652	30 [90%]	47	2

Supplementary Table 3. Comparison of gene and drug members of drug-induced modules across human cell lines and organisms (separate file).

Supplementary Table 4. Characterization of gene and drug members of conserved drug-induced modules (CODIMs), CODIM-associated modules of rat liver and selected cell line-specific modules linked to a certain MOA. Functional enrichment of genes (biological process) and MOA enrichment of drugs were labeled in green if identified in the automated annotation process. For drug annotation, manually curated drug information was additionally gathered for CODIMs (colored in orange). Moreover, we checked the consistency of gene and drug annotations and categorized these associations in terms of their agreement with the current literature: green labels for previously reported associations, yellow for less obvious cases with poorly characterized drug mechanism of action, and grey for novel associations for which no clear understanding could be gathered from the literature (cases without detectable and interpretable enrichment in white).

	**Functional enrichment of genes (BP: biological process)**	**Most prominent drug mechanism of action (MOA)**	**Literature support for association between BP and MOA**
CODIM1	BP: cell cycle process, M phase	cell cycle blockers	(Crawford & Piwnica-Worms, 2001; Cho *et al*, 2001; Whitfield *et al*, 2002)
CODIM2	BP: sterol biosynthetic process, cholesterol metabolic process	psycholeptic	(Fernø *et al*, 2005; Kristiana *et al*, 2010)
CODIM3	BP: citrullination, nucleosome assembly, chromatin assembly,	Protein synthesis inhibitors	
CODIM4	BP: response to wounding, defense/inflammatory response	Corticosteroid	(Barnes, 2006)
CODIM5	BP: response to unfolded protein and protein stimulus	Heat shock protein inhibitors	(Marcu *et al*, 2002)
CODIM6	BP: Unknown ; enrichment of LIM domain	Histone deactylase inhibitors	
CODIM7	BP: Pyrimidine metabolism, (m)RNA processing	Flavonoids	(Nose, 1984; Kanakis *et al*, 2006, 2007)
CODIM8	BP: inner ear development, sensory organ development, cell adhesion	Unknown	
CODIM9	BP: protein folding; keywords: transcriptional regulation	Unknown	
CODIM10	BP: Unknown; enrichment WD40 repeat	Unknown	
CODIM11	BP: intracellular transport, cellular protein localization	Unknown	
CODIM12	BP: Unknown	Unknown	
CODIM13	BP: Unknown; enrichment metallothionein, chelation	Serpine1	

CODIM14	BP: intracellular protein transport, protein localization	Unknown	
CODIM15	BP: L-serine metabolic process, Glycine, serine and threonine metabolism, Aminoacyl-tRNA biosynthesis	SLC6A2	
CODIM16	BP: ncRNA metabolic process	HRH1	
CODIM17	BP: Unknown; enrichment WD40 repeat	NA+/K+ pump inhibitors	
CODIM18	BP: Unknown; enrichment for transmembrane region and signal peptide	Unknown	
CODIM19	BP: Unknown	Unknown	
CODIM20	BP: vesicle-mediated transport	Unknown	
CODIM21	BP: defense/inflammatory response	Unknown	
CODIM22	BP: Unknown; enrichment for glycoprotein, signal peptide	Unknown	
CODIM23	BP: Unknown; enrichment Krueppel-associated box domain	Unknown	
**Cell line specific modules linked to certain MOA**			
MCF7-9	BP: G-protein coupled receptor protein signaling pathway, epithelium development	Estrogen receptor agonist/antagonist	
PC3-3	BP: response to protein stimulus, unfolded protein and organic substance	Histamine receptor H1 antagonist	
PC3-9	BP: Unknown	Peroxisome proliferator-activated receptor activator	
PC3-11	BP: wound healing; enrichment, complement and coagulation cascades	β-2 adrenergic receptor agonist	
HL60-17	BP: regulation of lymphocyte activation	β-2 adrenergic receptor agonist	
**Modules of rat liver associated with CODIM**			
Rat Liver-2	BP: response to bacterium, wounding and inflammatory, defense response	Non-sterodial anti-inflammatory drug	
Rat Liver-7	BP: Unknown	Corticosteroid	
Rat Liver-12	BP: lipid, sterol biosynthetic process, cholesterol metabolic process	Statins	
Rat Liver-22	BP: chemotaxis, leukocyte migration, migration, immune response	Unknown	
Rat Liver-23	BP: positive regulation of macromolecule biosynthetic process, regulation of transcription	Unknown	
Rat Liver-36	BP: cell cycle process, M phase	Unknown	

Supplementary Table 5. Detailed information on the characterization of gene and drug members of unified conserved drug-induced modules (CODIMs), CODIM-associated modules of rat liver and selected cell line-specific modules linked to a certain MOA.

CODIM 1

108 genes, 41 chemicals

Functional enrichment of genes:

Annotation Cluster 1	Enrichment Score: 32.82	
Category	Term	P-value
GOTERM_BP_FAT	GO:0022403~cell cycle phase	2.17E-49
GOTERM_BP_FAT	GO:0022402~cell cycle process	3.14E-47
GOTERM_BP_FAT	GO:0000279~M phase	1.13E-46
GOTERM_BP_FAT	GO:0000278~mitotic cell cycle	2.02E-45
GOTERM_BP_FAT	GO:0007049~cell cycle	3.92E-43
GOTERM_BP_FAT	GO:0000280~nuclear division	6.23E-43
GOTERM_BP_FAT	GO:0007067~mitosis	6.23E-43
GOTERM_BP_FAT	GO:0000087~M phase of mitotic cell cycle	1.41E-42
SP_PIR_KEYWORDS	cell cycle	1.45E-42
GOTERM_BP_FAT	GO:0048285~organelle fission	5.27E-42
SP_PIR_KEYWORDS	cell division	2.94E-39
SP_PIR_KEYWORDS	mitosis	3.45E-39
GOTERM_BP_FAT	GO:0051301~cell division	2.17E-32
GOTERM_CC_FAT	GO:0005819~spindle	3.78E-30
GOTERM_CC_FAT	GO:0015630~microtubule cytoskeleton	7.66E-28
GOTERM_CC_FAT	GO:0044430~cytoskeletal part	5.66E-22
GOTERM_CC_FAT	GO:0005856~cytoskeleton	6.46E-18
GOTERM_CC_FAT	GO:0043228~non-membrane-bounded organelle	4.09E-17
GOTERM_CC_FAT	GO:0043232~intracellular non-membrane-bounded organelle	4.09E-17
SP_PIR_KEYWORDS	cytoskeleton	2.01E-12
SP_PIR_KEYWORDS	coiled coil	2.77E-04
**Annotation Cluster 2**	**Enrichment Score: 15.65**	
Category	Term	P-value
GOTERM_BP_FAT	GO:0007017~microtubule-based process	1.03E-24
GOTERM_BP_FAT	GO:0000226~microtubule cytoskeleton organization	1.22E-17
GOTERM_BP_FAT	GO:0007051~spindle organization	4.79E-12
GOTERM_BP_FAT	GO:0007010~cytoskeleton organization	3.98E-11
**Annotation Cluster 3**	**Enrichment Score: 10.90**	
Category	Term	P-value
GOTERM_BP_FAT	GO:0007059~chromosome segregation	1.36E-16
GOTERM_BP_FAT	GO:0000819~sister chromatid segregation	2.94E-13
GOTERM_BP_FAT	GO:0000070~mitotic sister chromatid segregation	2.94E-13
GOTERM_BP_FAT	GO:0051276~chromosome organization	1.33E-09
GOTERM_BP_FAT	GO:0051640~organelle localization	3.15E-09
GOTERM_BP_FAT	GO:0051656~establishment of organelle localization	7.56E-08

Genes:

probe set	Gene Symbol	Ensembl ID	Protein name	# of cell lines
201088_at	KPNA2	ENSP00000332455	karyopherin alpha 2 (RAG cohort 1, importin alpha 1)	3
201291_s_at	TOP2A	ENSP00000411532	topoisomerase (DNA) II alpha 170kDa	3
201896_s_at	PSRC1	ENSP00000358925	proline/serine-rich coiled-coil 1	3
202240_at	PLK1	ENSP00000300093	polo-like kinase 1	3
202705_at	CCNB2	ENSP00000288207	cyclin B2	3
202870_s_at	CDC20	ENSP00000308450	cell division cycle 20 homolog (S. cerevisiae)	3

202954_at	UBE2C	ENSP00000348838	ubiquitin-conjugating enzyme E2C	3
203554_x_at	PTTG1	ENSP00000344936	pituitary tumor-transforming 1	3
203755_at	BUB1B	ENSP00000287598	budding uninhibited by benzimidazoles 1 homolog beta (yeast)	3
203764_at	DLGAP5	ENSP00000247191	discs, large (Drosophila) homolog-associated protein 5	3
204092_s_at	AURKA	ENSP00000216911	aurora kinase A	3
204170_s_at	CKS2	ENSP00000364976	CDC28 protein kinase regulatory subunit 2	3
204641_at	NEK2	ENSP00000355966	NIMA (never in mitosis gene a)-related kinase 2	3
204649_at	TROAP	ENSP00000257909	trophinin associated protein (tastin)	3
204709_s_at	KIF23	ENSP00000260363	kinesin family member 23	3
204826_at	CCNF	ENSP00000380256	cyclin F	3
204962_s_at	CENPA	ENSP00000336868	centromere protein A	3
205046_at	CENPE	ENSP00000265148	centromere protein E, 312kDa	3
205240_at	GPSM2	ENSP00000264126	G-protein signaling modulator 2	3
206364_at	KIF14	ENSP00000356319	kinesin family member 14	3
207165_at	HMMR	ENSP00000377492	hyaluronan-mediated motility receptor (RHAMM)	3
207828_s_at	CENPF	ENSP00000355922	centromere protein F, 350/400kDa (mitosin)	3
209408_at	KIF2C	ENSP00000361298	kinesin family member 2C	3
209464_at	AURKB	ENSP00000313950	aurora kinase B	3
209642_at	BUB1	ENSP00000302530	budding uninhibited by benzimidazoles 1 homolog (yeast)	3
210052_s_at	TPX2	ENSP00000300403	TPX2, microtubule-associated, homolog (Xenopus laevis)	3
212020_s_at	MKI67	ENSP00000357643	antigen identified by monoclonal antibody Ki-67	3
214710_s_at	CCNB1	ENSP00000256442	cyclin B1	3
218009_s_at	PRC1	ENSP00000377793	protein regulator of cytokinesis 1	3
218039_at	NUSAP1	ENSP00000260359	nucleolar and spindle associated protein 1	3
218355_at	KIF4A	ENSP00000363524	kinesin family member 4A	3
218726_at	HJURP	ENSP00000414109	Holliday junction recognition protein	3
218755_at	KIF20A	ENSP00000378356	kinesin family member 20A	3
219918_s_at	ASPM	ENSP00000356379	asp (abnormal spindle) homolog, microcephaly associated (Drosophila)	3
220295_x_at	DEPDC1	ENSP00000412292	DEP domain containing 1	3
221436_s_at	CDCA3	ENSP00000229265	cell division cycle associated 3	3
221520_s_at	CDCA8	ENSP00000316121	cell division cycle associated 8	3
222039_at	KIF18B	ENSP00000341466	kinesin family member 18B	3
201774_s_at	NCAPD2	ENSP00000325017	non-SMC condensin I complex, subunit D2	2
201853_s_at	CDC25B	ENSP00000245960	cell division cycle 25 homolog B (S. pombe)	2
202094_at	BIRC5	ENSP00000301633	baculoviral IAP repeat containing 5	2
202779_s_at	UBE2S	ENSP00000264552	ubiquitin-conjugating enzyme E2S	2
203145_at	SPAG5	ENSP00000323300	sperm associated antigen 5	2
203213_at	CDK1	ENSP00000306043	cyclin-dependent kinase 1	2
203418_at	CCNA2	ENSP00000274026	cyclin A2	2
204162_at	NDC80	ENSP00000261597	NDC80 homolog, kinetochore complex component (S. cerevisiae)	2
204244_s_at	DBF4	ENSP00000265728	DBF4 homolog (S. cerevisiae)	2
204315_s_at	GTSE1	ENSP00000160874	G-2 and S-phase expressed 1	2
204444_at	KIF11	ENSP00000260731	kinesin family member 11	2
204822_at	TTK	ENSP00000230510	TTK protein kinase	2
205167_s_at	CDC25C	ENSP00000321656	cell division cycle 25 homolog C (S. pombe)	2
209714_s_at	CDKN3	ENSP00000335357	cyclin-dependent kinase inhibitor 3	2
212738_at	ARHGAP19	ENSP00000351333	Rho GTPase activating protein 19	2
212832_s_at	CKAP5	ENSP00000346566	cytoskeleton associated protein 5	2
218252_at	CKAP2	ENSP00000367276	cytoskeleton associated protein 2	2

218308_at	TACC3	ENSP00000326550	transforming, acidic coiled-coil containing protein 3	2
218542_at	CEP55	ENSP00000360540	centrosomal protein 55kDa	2
219544_at	C13ORF34	ENSP00000375082	Protein aurora borealis	2
219787_s_at	ECT2	ENSP00000232458	epithelial cell transforming sequence 2 oncogene	2
221258_s_at	KIF18A	ENSP00000263181	kinesin family member 18A	2
221591_s_at	FAM64A	ENSP00000308470	family with sequence similarity 64, member A	2
221685_s_at	CCDC99	ENSP00000265295	coiled-coil domain containing 99	2
222077_s_at	RACGAP1	ENSP00000309871	Rac GTPase activating protein 1	2
200974_at	ACTA2	ENSP00000224784	actin, alpha 2, smooth muscle, aorta	1
201236_s_at	BTG2	ENSP00000290551	BTG family, member 2	1
201464_x_at	JUN	ENSP00000360266	jun proto-oncogene	1
201663_s_at	SMC4	ENSP00000341382	structural maintenance of chromosomes 4	1
201795_at	LBR	ENSP00000272163	lamin B receptor	1
202149_at	NEDD9	ENSP00000368759	neural precursor cell expressed, developmentally down-regulated 9	1
202227_s_at	BRD8	ENSP00000254900	bromodomain containing 8	1
202284_s_at	CDKN1A	ENSP00000244741	cyclin-dependent kinase inhibitor 1A (p21, Cip1)	1
202672_s_at	ATF3	ENSP00000344352	activating transcription factor 3	1
202859_x_at	IL8	ENSP00000306512	interleukin 8	1
203409_at	DDB2	ENSP00000256996	damage-specific DNA binding protein 2, 48kDa	1
203574_at	NFIL3	ENSP00000297689	nuclear factor, interleukin 3 regulated	1
203725_at	GADD45A	ENSP00000360025	growth arrest and DNA-damage-inducible, alpha	1
204285_s_at	PMAIP1	ENSP00000326119	phorbol-12-myristate-13-acetate-induced protein 1	1
204492_at	ARHGAP11A	ENSP00000355090	Rho GTPase activating protein 11A	1
204748_at	PTGS2	ENSP00000356438	prostaglandin-endoperoxide synthase 2 (prostaglandin G/H synthase and cyclooxygenase)	1
204780_s_at	FAS	ENSP00000347979	Fas (TNF receptor superfamily, member 6)	1
204817_at	ESPL1	ENSP00000257934	extra spindle pole bodies homolog 1 (S. cerevisiae)	1
204855_at	SERPINB5	ENSP00000372221	serpin peptidase inhibitor, clade B (ovalbumin), member 5	1
204977_at	DDX10	ENSP00000314348	DEAD (Asp-Glu-Ala-Asp) box polypeptide 10	1
205114_s_at	CCL3	ENSP00000225245	chemokine (C-C motif) ligand 3	1
205220_at	GPR109B	ENSP00000375066	G protein-coupled receptor 109B	1
205235_s_at	KIF20B	ENSP00000260753	kinesin family member 20B	1
205266_at	LIF	ENSP00000249075	leukemia inhibitory factor (cholinergic differentiation factor)	1
205281_s_at	PIGA	ENSP00000369820	phosphatidylinositol glycan anchor biosynthesis, class A	1
205436_s_at	H2AFX	ENSP00000364310	H2A histone family, member X	1
205967_at	HIST1H4C	ENSP00000367034	histone cluster 1, H4c	1
207813_s_at	FDXR	ENSP00000293195	ferredoxin reductase	1
208808_s_at	HMGB2	ENSP00000296503	high-mobility group box 2	1
208886_at	H1F0	ENSP00000344504	H1 histone family, member 0	1
209122_at	PLIN2	ENSP00000276914	perilipin 2	1
209457_at	DUSP5	ENSP00000358596	dual specificity phosphatase 5	1
209610_s_at	SLC1A4	ENSP00000234256	solute carrier family 1 (glutamate/neutral amino acid transporter), member 4	1
209795_at	CD69	ENSP00000228434	CD69 molecule	1
210609_s_at	TP53I3	ENSP00000238721	tumor protein p53 inducible protein 3	1
211935_at	ARL6IP1	ENSP00000306788	ADP-ribosylation factor-like 6 interacting protein 1	1
212949_at	NCAPH	ENSP00000240423	non-SMC condensin I complex, subunit H	1
217373_x_at	MDM2	ENSP00000417281	Mdm2 p53 binding protein homolog (mouse)	1
217996_at	PHLDA1	ENSP00000266671	pleckstrin homology-like domain, family A, member 1	1
218346_s_at	SESN1	ENSP00000306734	sestrin 1	1
218662_s_at	NCAPG	ENSP00000251496	non-SMC condensin I complex, subunit G	1

219306_at	KIF15	ENSP00000324020	kinesin family member 15	1
219392_x_at	PRR11	ENSP00000262293	proline rich 11	1
219936_s_at	GPR87	ENSP00000260843	G protein-coupled receptor 87	1
221586_s_at	E2F5	ENSP00000398124	E2F transcription factor 5, p130-binding	1

Drug annotation:

No enriched terms found.

Drugs	# of cell lines	Drug annotation	Literature evidence
ciclopirox	3	cell cycle blocker; antifungal (ATC: D01AE14)	(Zhou *et al*, 2010)
etoposide	3	cell cycle blocker; anticancer agent (ATC: L01CB01)	(Gordaliza *et al*, 2004)
methotrexate	3	cell cycle blocker; antimetabolite (ATC: L01BA01)	(Hertz *et al*, 1956)
monobenzone	3	depigmentation agent (ATC: D11AX13)	
pyrimethamine	3	cell cycle blocker; antimalarial (ATC: P01BD01)	(Giammarioli *et al*, 2008)
trifluridine	3	cell cycle blocker; thymidine analog/antiviral (ATC: S01AD02)	(Temmink *et al*, 2004)
vidarabine	3	cell cycle blocker; antiviral (ATC: J05AB03)	(Brink & Lepage, 1965)
thioguanosine	2	cell cycle blocker; guanine analog	(Lepage, 1963)
triamterene	2	diuretic (ATC: C03DB02)	
vinburnine	2	vasodilator (ATC: C04AX17)	
(+)-chelidonine	1	cell cycle blocker	(Panzer *et al*, 2001)
15-delta prostaglandin J2	1	NSAID; inhibits cell proliferation	(Kim <i>et al</i>, 2007)
8-azaguanine	1	cell cycle blocker; purine analog	(Colsky *et al*, 1955)
atovaquone	1	analog of ubiquinone (ATC: P01AX06); cytotoxic derivatives	(Zhou *et al*, 2009)
betulinic acid	1	anti-retroviral, anti-malarial, and anti-inflammatory; cell cycle blocker	(Fulda, 2008)
cefotiam	1	inhibit proliferation; antibiotic (ATC: J01DC07)	(Akira *et al*, 2000)
cinchonine	1	alkaloid	
clioquinol	1	cell cycle blocker; antifungal (ATC: G01AC02)	(Zhai *et al*, 2010)
dequalinium chloride	1	anticarcinogenic activity; antiseptic (ATC: D08AH01)	(Weiss *et al*, 1987)
diethylstilbestrol	1	inhibits proliferation of human prostate cancer cell lines; nonsteroidal estrogen (ATC: G03CB02)	(Koike *et al*, 2005)
ethaverine	1	Vasodilator and antispasmodic; structurally similar to papaverine	
ethoxyquin	1	antioxidant; genotoxic/induces apoptosis	(Błaszczyk & Skolimowski, 2005)
gossypol	1	antiproliferative activity	(Le Blanc *et al*, 2002)
hycanthone	1	cytotoxic; cell cycle blocker; inhibits RNA synthesis	(Sieber *et al*, 1973; Wong *et al*, 1990)
mephentermine	1	cardiac stimulant (ATC: C01CA11)	
methylbenzethonium chloride	1	induces apoptosis	(Yip *et al*, 2006)
nifuroxazide	1	inhibits myeloma survival; antibiotic (ATC: A07AX03)	(Nelson *et al*, 2008)
paclitaxel	1	cell cycle blocker; stabilizes microtubules	(Jordan *et al*, 1993)
primaquine	1	anti-malaria drug (ATC: P01BA03)	
progesterone	1	inhibits epithelial cell proliferatione; hormon (ATC: G03DA04)	(Hsu *et al*, 2008)
pyrvinium	1	inhibits proliferation in colon cancer ; antiparasitic (ATC: P02CX01)	(Thorne *et al*, 2010)
rimexolone	1	cell cycle blocker; corticosteroid for systemic use (ATC: H02AB12)	(Spies *et al*, 2010)
seneciphylline	1	cytotoxic/mutagenic agent (not anti-proliferative)	(Griffin & Segall, 1986)
sirolimus	1	inhibits proliferation; immunosuppressant (ATC: L04AA10)	(Wang *et al*, 2007)
sulconazole	1	antifungal (ATC: D01AC09)	
thioridazine	1	cytogenetic damage; inhibits cell proliferation; antipsychotic drug (ATC: N05AC02)	(Strobl *et al*, 1990)
ticarcillin	1	antiproliferative effect on human cell lines; beta-lactam inhibitor (antibiotic) (ATC: J01CA13)	(Weston *et al*, 1986)
trifluoperazine	1	reduces cell proliferation; anticalmodulin and blocker of phospholipase A2; antipsychotic (ATC: N05AB06)	(Izaguirre Roncal *et al*, 2001)

trimethylcolchicinic acid	1	cell cycle blocker; inhibits microtubule polymerization	(Lessner *et al*, 1963)
zalcitabine	1	mitochondrial toxicity; nucleoside analog reverse transcriptase inhibitor (ATC: J05AF03)	(Walker *et al*, 2003)
zidovudine	1	cell cycle blocker; nucleoside analog reverse-transcriptase inhibitor (ATC: J05AF01)	(Wagner *et al*, 1997)

CODIM 2

62 genes, 63 chemicals

Functional enrichment of genes:

**Annotation Cluster 1**	**Enrichment Score: 19.04**	
Category	Term	P-value
SP_PIR_KEYWORDS	lipid synthesis	3.64E-30
SP_PIR_KEYWORDS	Steroid biosynthesis	1.08E-29
GOTERM_BP_FAT	GO:0016126~sterol biosynthetic process	2.97E-25
GOTERM_BP_FAT	GO:0006694~steroid biosynthetic process	2.38E-24
SP_PIR_KEYWORDS	sterol biosynthesis	1.19E-23
GOTERM_BP_FAT	GO:0016125~sterol metabolic process	3.59E-23
GOTERM_BP_FAT	GO:0008202~steroid metabolic process	4.89E-23
GOTERM_BP_FAT	GO:0008610~lipid biosynthetic process	7.93E-21
GOTERM_BP_FAT	GO:0008203~cholesterol metabolic process	1.27E-18
GOTERM_BP_FAT	GO:0006695~cholesterol biosynthetic process	2.22E-18
SP_PIR_KEYWORDS	Cholesterol biosynthesis	5.52E-18
KEGG_PATHWAY	hsa00100:Steroid biosynthesis	2.16E-17
SP_PIR_KEYWORDS	oxidoreductase	6.00E-09
GOTERM_BP_FAT	GO:0055114~oxidation reduction	9.65E-08
SP_PIR_KEYWORDS	nadp	2.53E-07
**Annotation Cluster 2**	**Enrichment Score: 9.49**	
Category	Term	P-value
KEGG_PATHWAY	hsa00100:Steroid biosynthesis	2.16E-17
GOTERM_CC_FAT	GO:0005783~endoplasmic reticulum	1.10E-15
GOTERM_CC_FAT	GO:0042175~nuclear envelope-endoplasmic reticulum network	1.11E-09
GOTERM_CC_FAT	GO:0005789~endoplasmic reticulum membrane	6.74E-09
GOTERM_CC_FAT	GO:0044432~endoplasmic reticulum part	1.21E-08
GOTERM_CC_FAT	GO:0012505~endomembrane system	1.53E-06
GOTERM_CC_FAT	GO:0031090~organelle membrane	1.07E-04
**Annotation Cluster 3**	**Enrichment Score: 9.38**	
Category	Term	P-value
SP_PIR_KEYWORDS	sterol biosynthesis	1.19E-23
GOTERM_BP_FAT	GO:0008203~cholesterol metabolic process	1.27E-18
GOTERM_BP_FAT	GO:0006695~cholesterol biosynthetic process	2.22E-18
SP_PIR_KEYWORDS	Cholesterol biosynthesis	5.52E-18
GOTERM_BP_FAT	GO:0008299~isoprenoid biosynthetic process	4.13E-08
KEGG_PATHWAY	hsa00900:Terpenoid backbone biosynthesis	4.10E-07
GOTERM_BP_FAT	GO:0006720~isoprenoid metabolic process	1.27E-06
SP_PIR_KEYWORDS	Isoprene biosynthesis	9.93E-04
GOTERM_CC_FAT	GO:0042579~microbody	0.014490138
GOTERM_CC_FAT	GO:0005777~peroxisome	0.014490138
SP_PIR_KEYWORDS	peroxisome	0.066043141

Genes:

**probe**	**gene symbol**	**Protein name**	**cluster label**	**# of cell lines**	**Selected genes for siRNA screening**
201790_s_at	DHCR7	7-dehydrocholesterol reductase	blue	3	Not tested
202245_at	LSS	lanosterol synthase (2,3-oxidosqualene-lanosterol cyclase)	blue	3	Not tested
201275_at	FDPS	farnesyl diphosphate synthase	blue	3	Not tested
208647_at	FDFT1	farnesyl-diphosphate farnesyltransferase 1	blue	3	Not tested
200831_s_at	SCD	stearoyl-CoA desaturase (delta-9-desaturase)	blue	3	Not tested

202539_s_at	HMGCR	3-hydroxy-3-methylglutaryl-Coenzyme A reductase	blue	3	3 siRNAs
211423_s_at	SC5DL	sterol-C5-desaturase (ERG3 delta-5-desaturase homolog, S. cerevisiae)-like	blue	3	Not tested
209218_at	SQLE	squalene epoxidase	blue	3	2 siRNAs
201625_s_at	INSIG1	insulin induced gene 1	blue	3	2 siRNAs
204615_x_at	IDI1	isopentenyl-diphosphate delta isomerase 1	blue	3	Not tested
209146_at	SC4MOL	sterol-C4-methyl oxidase-like	blue	3	Not tested
220081_x_at	HSD17B7	hydroxysteroid (17-beta) dehydrogenase 7	green	3	Not tested
202218_s_at	FADS2	fatty acid desaturase 2	blue	2	Not tested
212272_at	LPIN1	lipin 1	blue	2	2 siRNAs
212218_s_at	FASN	fatty acid synthase	blue	2	Not tested
202679_at	NPC1	Niemann-Pick disease, type C1	blue	2	3 siRNAs
208926_at	NEU1	sialidase 1 (lysosomal sialidase)	blue	2	Not tested
209608_s_at	ACAT2	acetyl-CoA acetyltransferase 2	blue	2	Not tested
202314_at	CYP51A1	cytochrome P450, family 51, subfamily A, polypeptide 1	blue	2	Not tested
209279_s_at	NSDHL	NAD(P) dependent steroid dehydrogenase-like	blue	2	Not tested
202067_s_at	LDLR	low density lipoprotein receptor	blue	2	3 siRNAs
221249_s_at	FAM117 A	family with sequence similarity 117, member A	green	2	2 siRNAs
212622_at	TMEM41 B	transmembrane protein 41B	green	2	4 siRNAs
209118_s_at	TUBA1A	tubulin, alpha 1a	blue	1	4 siRNAs
203027_s_at	MVD	mevalonate (diphospho) decarboxylase	blue	1	Not tested
202735_at	EBP	emopamil binding protein (sterol isomerase)	blue	1	Not tested
201127_s_at	ACLY	ATP citrate lyase	blue	1	Not tested
200862_at	DHCR24	24-dehydrocholesterol reductase	blue	1	Not tested
202562_s_at	C14orf1	chromosome 14 open reading frame 1	blue	1	Not tested
208962_s_at	FADS1	fatty acid desaturase 1	blue	1	Not tested
205822_s_at	HMGCS1	3-hydroxy-3-methylglutaryl-CoA synthase 1 (soluble)	blue	1	Not tested
201193_at	IDH1	isocitrate dehydrogenase 1 (NADP+), soluble	blue	1	4 siRNAs
217869_at	HSD17B1 2	hydroxysteroid (17-beta) dehydrogenase 12	blue	1	Not tested
200632_s_at	NDRG1	N-myc downstream regulated 1	brown	1	Not tested
209122_at	PLIN2	perilipin 2	brown	1	Not tested
202497_x_at	SLC2A3	solute carrier family 2 (facilitated glucose transporter), member 3	brown	1	Not tested
216236_s_at	SLC2A14	solute carrier family 2 (facilitated glucose transporter), member 14	brown	1	3 siRNAs
202988_s_at	RGS1	regulator of G-protein signaling 1	brown	1	4 siRNAs
209875_s_at	SPP1	secreted phosphoprotein 1	brown	1	4 siRNAs
204014_at	DUSP4	dual specificity phosphatase 4	dark green	1	4 siRNAs
204698_at	ISG20	interferon stimulated exonuclease gene 20kDa	dark green	1	4 siRNAs
219117_s_at	FKBP11	FK506 binding protein 11, 19 kDa	green	1	2 siRNAs
209173_at	AGR2	anterior gradient homolog 2 (Xenopus laevis)	green	1	4 siRNAs
202375_at	SEC24D	SEC24 family, member D (S. cerevisiae)	green	1	Not tested
218696_at	EIF2AK3	eukaryotic translation initiation factor 2-alpha kinase 3	green	1	4 siRNAs
219390_at	FKBP14	FK506 binding protein 14, 22 kDa	green	1	Not tested
210041_s_at	PGM3	phosphoglucomutase 3	green	1	4 siRNAs
217858_s_at	ARMCX3	armadillo repeat containing, X-linked 3	green	1	4 siRNAs
212345_s_at	CREB3L2	cAMP responsive element binding protein 3-like 2	green	1	Not tested
211936_at	HSPA5	heat shock 70kDa protein 5 (glucose-regulated protein, 78kDa)	green	1	4 siRNAs
202842_s_at	DNAJB9	DnaJ (Hsp40) homolog, subfamily B, member 9	green	1	4 siRNAs
217168_s_at	HERPUD 1	homocysteine-inducible, endoplasmic reticulum stress-inducible, ubiquitin-like domain member 1	green	1	Not tested
203675_at	NUCB2	nucleobindin 2	green	1	2 siRNAs

202721_s_at	GFPT1	glutamine--fructose-6-phosphate transaminase 1	green	1	Not tested
203827_at	WIPI1	WD repeat domain, phosphoinositide interacting 1	green	1	4 siRNAs
202769_at	CCNG2	cyclin G2	green	1	Not tested
202887_s_at	DDIT4	DNA-damage-inducible transcript 4	green	1	Not tested
202341_s_at	TRIM2	tripartite motif containing 2	green	1	4 siRNAs
218437_s_at	LZTFL1	leucine zipper transcription factor-like 1	green	1	4 siRNAs
218976_at	DNAJC12	DnaJ (Hsp40) homolog, subfamily C, member 12	green	1	4 siRNAs
206683_at	ZNF165	zinc finger protein 165	green	1	4 siRNAs
205014_at	FGFBP1	fibroblast growth factor binding protein 1	light blue	1	4 siRNAs

Drug annotation:

**Annotation type**	**Enriched term**	**p-value (FDR)**
Drug target	HTR2A; 5-hydroxytryptamine (serotonin) receptor 2A	3.60E-05
Drug target	HRH1; histamine receptor H1	8.94E-03
Drug target	DRD1; dopamine receptor D1	1.10E-02
Drug target	DRD2; dopamine receptor D2	1.45E-02
Drug target	HTR1A; 5-hydroxytryptamine (serotonin) receptor 1A	1.48E-02
Drug target	DRD4; dopamine receptor D4	2.22E-02
Drug target	CHRM5; cholinergic receptor, muscarinic 5	2.40E-02
Drug target	SLC6A4; solute carrier family 6 (neurotransmitter transporter, serotonin), member 4	2.65E-02
Drug target	DRD3; dopamine receptor D3	2.72E-02
Drug target	CYP2D6; cytochrome P450, family 2, subfamily D, polypeptide 6	3.04E-02
Drug target	CHRM3; cholinergic receptor, muscarinic 3	3.10E-02
Drug target	SLC6A2; solute carrier family 6 (neurotransmitter transporter, noradrenalin), member 2	3.10E-02
Drug target	ADRA1A; adrenergic, alpha-1A-, receptor	3.58E-02
Drug target	HTR2C; 5-hydroxytryptamine (serotonin) receptor 2C	6.47E-02
Drug target	CHRM1; cholinergic receptor, muscarinic 1	6.53E-02
Drug target	CHRM4; cholinergic receptor, muscarinic 4	8.95E-02
ATC code (2nd level)	N05; psycholeptic	4.25E-03
Side effect	paralytic ileus	2.00E-04
Side effect	galactorrhea	3.33E-04
Side effect	adenitis	3.33E-04
Side effect	gynecomastia	6.19E-03
Side effect	SIADH	1.07E-02
Side effect	breast enlargement	1.21E-02
Side effect	testicular swelling	1.57E-02
Chemical Fragment	O=C(c1ccc(F)cc1)CCC	7.73E-04
Chemical Fragment	c1c3OCOc3(cc2c1CN(C)CC2)	7.73E-04
Chemical Fragment	C(c1ccccc1)c2ccccc2	7.73E-04
Chemical Fragment	C(=C(NCc1cnc(nc1(N))C)C)S	7.73E-04
Chemical Fragment	c1ccc(cc1)C[N+](C)C	7.73E-04
Chemical Fragment	C1N(C)CCCC1	7.73E-04
Chemical Fragment	Cc1c(cccc1Cl)Cl	2.02E-03
Chemical Fragment	c1ccc(cc1)CN2CCN(C)CC2	7.68E-03

**Drugs**	**# of cell lines**	**Drug annotation**	**Literature evidence**
metixene	3	N04 anti-parkinson drug	
tetrandrine	3	calcium channel blocker	(Chen *et al*, 2011)
astemizole	2	histamine H1-receptor antagonist	

fluspirilene	2	N05 psycholeptic	
maprotiline	2	N06 psychoanaleptic	
metergoline	2	dopamine agonist and serotonin antagonist (ATC: G02CB05)	(Hamon *et al*, 1981)
nortriptyline	2	N05 osycholeptic	
perhexiline	2	antianginal agent (ATC: C08EX02)	
perphenazine	2	N05 psycholeptic	
prochlorperazine	2	N05 psycholeptic	
pregnenolone	1	GABAA antagonist, increases neurogenesis in the hippocampus	(Mayo *et al*, 2005)
mometasone	1	D07 corticosteroid, dermatological preparation; glucocorticosteroid (ATC: D07AC13)	
nifuroxazide	1	inhibits myeloma survival; antibiotic (ATC: A07AX03)	(Nelson *et al*, 2008)
thiostrepton	1	oligo-peptide antibiotic	
pimozide	1	N05 psycholeptic	
karakoline	1	ganglioblocker; nicotinic receptor antagonist	
trimethylcolchicinic acid	1	cell cycle blocker; inhibits microtubule polymerization	(Lessner *et al*, 1963)
piperacetazine	1	antipsychotic prodrug	(Rada & Donlon, 1974)
amodiaquine	1	anti-inflammatory drug (ATC: P01BA06)	
amoxapine	1	N06 psychoanaleptic	
etiocholanolone	1	metabolite of testosterone	
suloctidil	1	vasodilator (ATC: C04AX19)	
chlorpromazine	1	N05 psycholeptic	
chlorprothixene	1	N05 psycholeptic	
ciclopirox	1	cell cycle blocker; antifungal (ATC: D01AE14)	(Zhou *et al*, 2010)
ciclosporin	1	immunosuppressant drug (ATC: L04AD01)	
dequalinium chloride	1	anticarcinogenic activity; antiseptic (ATC: D08AH01)	(Weiss *et al*, 1987)
desipramine	1	N06 psychoanaleptic	
dydrogesterone	1	progesterone hormone (ATC: G03DB01)	
econazole	1	antifungal (ATC: D01AC03)	
ethaverine	1	vasodilator and antispasmodic	
famprofazone	1	NSAID	
fluoxetine	1	N06 psychoanaleptic	
geldanamycin	1	HSP90 inhibitor	(Miyata, 2005a)
glafenine	1	N02 analgesic	
hecogenin	1	has antioxidant and anti-inflammatory properties	(Santos Cerqueira *et al*, 2012)
homochlorcyclizine	1	antihistamine	(Haraguchi *et al*, 1997)
hydrocotarnine	1	alkaloid	
ketoconazole	1	antifungal drug (ATC: D01AC08)	
loperamide	1	opioid drug used against diarrhea (ATC: A07DA03)	
mestranol	1	estrogen	
metitepine	1	antipsychotic	(Monachon *et al*, 1972)
methylbenzethonium chloride	1	induces apoptosis	(Yip *et al*, 2006)
nabumetone	1	NSAID	(Gonzalo-Garijo *et al*, 2007)
nitrendipine	1	calcium channel blocker (ATC: C08CA08)	
oxetacaine	1	local anesthetic (ATC: C05AD06)	
papaverine	1	antispasmodic (ATC: A03AD01); inhibits phosphodiesterase causing elevation of cyclic AMP	
procyclidine	1	N04 anti-parkinson drug	
promazine	1	N05 psycholeptic	
propafenone	1	anti-arrhythmic medication (ATC: C01BC03)	
protriptyline	1	N06 psychoanaleptic	
puromycin	1	antibiotic; protein synthesis inhibitor	

raloxifene	1	selective estrogen receptor modulator (ATC: G03XC01)	
terconazole	1	antifungal (ATC: G01AG02)	
thiethylperazine	1	antiemetic (ATC: R06AD03); potential antipsychotic activity	(Rotrosen *et al*, 1978)
thioridazine	1	N05 psycholeptic (ATC: N05AC02); cytogenetic damage; inhibit cell proliferation;	(Strobl *et al*, 1990)
tracazolate	1	anxiolytic drug	(Patel & Malick, 1982)
trimipramine	1	N06 psychoanaleptic	
saquinavir	1	antiretroviral (ATC: J05AE01)	
atovaquone	1	analog of ubiquinone (ATC: P01AX06)	(Ittarat *et al*, 1995)
emetine	1	anti-protozoal; protein synthesis inhibitor	
thioproperazine	1	N05 psycholeptic	
norcyclobenzaprine	1	structurally similar to tricyclic antidepressants	(Van Hoey)

CODIM 3

58 genes, 51 chemicals

Functional enrichment of genes:

**Annotation Cluster 1**	**Enrichment Score: 5.10**	
Category	Term	P-value
SP_PIR_KEYWORDS	citrullination	8.85E-12
SP_PIR_KEYWORDS	nucleosome core	2.58E-11
INTERPRO	IPR007125:Histone core	4.91E-11
GOTERM_CC_FAT	GO:0000786~nucleosome	3.85E-10
SP_PIR_KEYWORDS	methylation	8.40E-10
GOTERM_CC_FAT	GO:0032993~protein-DNA complex	1.93E-08
GOTERM_BP_FAT	GO:0006334~nucleosome assembly	4.46E-08
GOTERM_BP_FAT	GO:0031497~chromatin assembly	6.02E-08
KEGG_PATHWAY	hsa05322:Systemic lupus erythematosus	7.18E-08
GOTERM_BP_FAT	GO:0065004~protein-DNA complex assembly	1.06E-07
SP_PIR_KEYWORDS	DNA binding	1.12E-07
GOTERM_BP_FAT	GO:0034728~nucleosome organization	1.21E-07
GOTERM_CC_FAT	GO:0000785~chromatin	1.42E-07
INTERPRO	IPR002119:Histone H2A	3.17E-07
SMART	SM00414:H2A	6.64E-07
GOTERM_BP_FAT	GO:0006323~DNA packaging	7.45E-07
SP_PIR_KEYWORDS	dna-binding	9.17E-07
SP_PIR_KEYWORDS	chromosomal protein	1.02E-06
PIR_SUPERFAMILY	PIRSF002048:histone H2A	1.13E-06
GOTERM_BP_FAT	GO:0006333~chromatin assembly or disassembly	1.44E-06
SP_PIR_KEYWORDS	ubl conjugation	7.21E-06
SP_PIR_KEYWORDS	isopeptide bond	9.47E-06
GOTERM_MF_FAT	GO:0003677~DNA binding	1.32E-05
UP_SEQ_FEATURE	cross-link:Glycyl lysine isopeptide (Lys-Gly) (interchain with G-Cter in ubiquitin)	2.62E-05
GOTERM_CC_FAT	GO:0044427~chromosomal part	4.99E-05
INTERPRO	IPR009072:Histone-fold	5.53E-05
SP_PIR_KEYWORDS	acetyllysine	1.13E-04
GOTERM_CC_FAT	GO:0005694~chromosome	1.76E-04
GOTERM_BP_FAT	GO:0034622~cellular macromolecular complex assembly	0.001213214
SP_PIR_KEYWORDS	nucleus	0.001282232
GOTERM_BP_FAT	GO:0034621~cellular macromolecular complex subunit organization	0.002299677
GOTERM_BP_FAT	GO:0006325~chromatin organization	0.002814147
SP_PIR_KEYWORDS	methylated amino acid	0.00376949
SP_PIR_KEYWORDS	transcription regulation	0.010608754
GOTERM_BP_FAT	GO:0051276~chromosome organization	0.012543863
GOTERM_BP_FAT	GO:0065003~macromolecular complex assembly	0.022578447
GOTERM_BP_FAT	GO:0043933~macromolecular complex subunit organization	0.029709331
UP_SEQ_FEATURE	mutagenesis site	0.036883111
GOTERM_CC_FAT	GO:0043232~intracellular non-membrane-bounded organelle	0.230087898
GOTERM_CC_FAT	GO:0043228~non-membrane-bounded organelle	0.230087898

SP_PIR_KEYWORDS	acetylation	0.625796507
**Annotation Cluster 2**	**Enrichment Score: 2.79**	
Category	Term	P-value
GOTERM_BP_FAT	GO:0006915~apoptosis	8.31E-04
GOTERM_BP_FAT	GO:0016265~death	8.76E-04
GOTERM_BP_FAT	GO:0012501~programmed cell death	9.47E-04
GOTERM_BP_FAT	GO:0008219~cell death	0.003019056
SP_PIR_KEYWORDS	Apoptosis	0.005314882

Drug annotation:

**Annotation type**	**Enriched term**	**p-value**
Drug target	CYP2D6; cytochrome P450, family 2, subfamily D, polypeptide 6	2.19E-02
Drug target	KCNH2; potassium voltage-gated channel, subfamily H (eag-related), member 2	2.40E-02
Drug target	CYP3A4; cytochrome P450, family 3, subfamily A, polypeptide 4	2.40E-02
Drug target	CALM1; calmodulin 1 (phosphorylase kinase, delta)	3.04E-02

**Drugs**	**# of cell lines**	**Drug annotation**	**Literature evidence**
anisomycin	3	protein synthesis inhibitor	(Chan, 2004)
cephaeline	3	protein synthesis inhibitor	(Chan, 2004)
emetine	3	protein synthesis inhibitor; anti-protozoal (ATC: P01AX02)	(Jiménez *et al*, 1977)
cicloheximide	2	protein synthesis inhibitor	(Baliga *et al*, 1969)
digoxigenin	2	Na+/K+-ATPase inhibitor	(Yoda, 1974)
azacyclonol	2	ataractive	(Braun *et al*, 1956)
helveticoside	2	Na+/K+-ATPase inhibitor	(Yoda, 1974)
astemizole	2	histamine H1-receptor antagonist	
suloctidil	2	vasodilator (ATC: C04AX19)	
digoxin	2	Na+/K+-ATPase inhibitor; cardiac glycoside (ATC: C01AA05)	
econazole	2	antifungal (ATC: D01AC03)	
lanatoside C	2	Na+/K+-ATPase inhibitor; cardiac glycoside (ATC: C01AA06)	
lycorine	2	toxic alkaloid	
mefloquine	2	quinine analogue; anti-malarial (ATC: P01BC02)	
ouabain	2	Na+/K+-ATPase inhibitor; cardiac glycoside (ATC: C01AC01)	
strophanthidin	2	Na+/K+-ATPase inhibitor	(Deitmer & Ellis, 1978)
digitoxigenin	2	Na+/K+-ATPase inhibitor	(Erlenkamp *et al*, 1998)
thioridazine	2	cytogenetic damage; inhibits cell proliferation; antipsychotic drug (ATC: N05AC02)	(Strobl *et al*, 1990)
prenylamine	2	calcium channel blocker (ATC: C01DX02)	
mometasone	1	D07 corticosteroid, dermatological preparation; glucocorticosteroid (ATC: D07AC13)	
pimozide	1	N05 psycholeptic	
bepridil	1	calcium channel blocker (ATC: C08EA02)	
bisacodyl	1	Na+/K+-ATPase inhibitor; stimulant laxative drug (ATC: A06AB02)	(Schreiner *et al*, 1980)
chlorprothixene	1	N05 psycholeptic	
clioquinol	1	cell cycle blocker; antifungal (ATC: G01AC02)	(Zhai *et al*, 2010)
dicycloverine	1	muscarinic receptor antagonist (ATC: A03AA07)	
felodipine	1	calcium channel blocker (ATC: C08CA02)	
fluoxetine	1	N06 psychoanaleptic	
fluspirilene	1	N05 psycholeptic	
hexestrol	1	steroid with veterinarian use	
hexetidine	1	anti-bacterial and anti-fungal (ATC: A01AB12)	
isoconazole	1	antifungal drug (ATC: D01AC05)	
vanoxerine	1	nicotinic acetylcholine receptor antagonist	PUBCHEM ID: 104920
Prestwick-664	1	nicotinic acetylcholine receptor antagonist	PUBCHEM ID:

			104920
loperamide	1	opioid drug used against diarrhea (ATC: A07DA03)	
LY-294002	1	PI3K inhibitor	(Maira *et al*, 2009)
8-azaguanine	1	cell cycle blocker; purine analog	(Colsky *et al*, 1955)
methylbenzethonium chloride	1	induces apoptosis	(Yip *et al*, 2006)
mianserin	1	psychoactive drug (ATC: N06AX03)	
miconazole	1	antifungal (ATC: D01AC02)	
beta-escin	1		
oxybutynin	1	anticholinergic medication (ATC: G04BD04)	
perhexiline	1	antianginal agent (ATC: C08EX02)	
proadifen	1	inhibits cytochrome P450 enzymes	(Marshall & Williamson, 1964)
propofol	1	hypnotic agent (ATC: N01AX10)	
sulconazole	1	antifungal (ATC: D01AC09)	
tamoxifen	1	estrogen receptor antagonist (ATC: L02BA01)	
tonzonium bromide	1	mono-catioinc detergent	
ticlopidine	1	antiplatelet drug (ATC: B01AC05)	
trimipramine	1	N06 psychoanaleptic	
quinisocaine	1	antipruritic (ATC: D04AB05)	

CODIM 4

173 genes, 43 chemicals

Functional enrichment of genes:

**Annotation Cluster 1**	**Enrichment Score: 6.59**	
Category	Term	P-value
GOTERM_BP_FAT	GO:0009611~response to wounding	3.46E-08
GOTERM_BP_FAT	GO:0006952~defense response	8.47E-08
GOTERM_BP_FAT	GO:0006954~inflammatory response	5.52E-06
**Annotation Cluster 2**	**Enrichment Score: 5.25**	
Category	Term	P-value
SP_PIR_KEYWORDS	signal	2.38E-08
UP_SEQ_FEATURE	signal peptide	2.47E-08
GOTERM_CC_FAT	GO:0005576~extracellular region	4.26E-06
SP_PIR_KEYWORDS	disulfide bond	5.11E-06
UP_SEQ_FEATURE	disulfide bond	1.09E-05
GOTERM_CC_FAT	GO:0044421~extracellular region part	1.91E-05
SP_PIR_KEYWORDS	glycoprotein	2.10E-05
UP_SEQ_FEATURE	glycosylation site:N-linked (GlcNAc...)	3.97E-05
GOTERM_CC_FAT	GO:0005615~extracellular space	4.67E-05
SP_PIR_KEYWORDS	Secreted	2.79E-04
**Annotation Cluster 3**	**Enrichment Score: 4.26**	
Category	Term	P-value
SP_PIR_KEYWORDS	chelation	2.00E-06
SP_PIR_KEYWORDS	metal-thiolate cluster	9.05E-06
UP_SEQ_FEATURE	metal ion-binding site:Divalent metal cation; cluster B	9.09E-06
UP_SEQ_FEATURE	region of interest:Beta	9.09E-06
UP_SEQ_FEATURE	region of interest:Alpha	9.09E-06
UP_SEQ_FEATURE	metal ion-binding site:Divalent metal cation; cluster A	9.09E-06
INTERPRO	IPR000006:metallothionein, vertebrate	1.07E-05
INTERPRO	IPR018064:metallothionein, vertebrate, metal binding site	1.07E-05
INTERPRO	IPR003019:metallothionein superfamily, eukaryotic	1.07E-05
PIR_SUPERFAMILY	PIRSF002564:metallothionein	1.97E-05
SP_PIR_KEYWORDS	metal binding	4.08E-05
SP_PIR_KEYWORDS	cadmium	1.38E-04
GOTERM_MF_FAT	GO:0046870~cadmium ion binding	2.79E-04
SP_PIR_KEYWORDS	acetylated amino end	0.001450927
SP_PIR_KEYWORDS	copper	0.019899055

GOTERM_MF_FAT	GO:0005507~copper ion binding	0.036920952

Drug annotation:

Annotation type	Enriched term	p-value
Drug target	NR3C1; nuclear receptor subfamily 3, group C, member 1 (glucocorticoid receptor)	1.45E-02
ATC code (2nd level)	D07; Corticosteroid, dermatological preparations	4.40E-18
ATC code (2nd level)	R01; Nasal preparations	1.10E-06
ATC code (2nd level)	H02; Corticosteroid for systemic use	2.36E-06
ATC code (2nd level)	R03; Drugs for obstructive airway diseases	8.18E-04
Side effect	striae	1.94E-13
Side effect	skin atrophy	1.69E-09
Side effect	hypopigmentation	9.66E-07
Side effect	miliaria	9.74E-07
Side effect	cataract	4.65E-05
Side effect	hypertrichosis	1.93E-04
Side effect	folliculitis	1.93E-04
Side effect	Aseptic necrosis	3.33E-04
Side effect	paraparesis	7.45E-04
Side effect	nasal septum perforation	7.45E-04
Side effect	alkalosis	9.74E-04
Side effect	ocular infection	1.18E-03
Side effect	exophthalmos	2.37E-03
Side effect	paraplegia	3.12E-03
Side effect	glaucoma	6.89E-03
Side effect	osteoporosis	1.07E-02
Side effect	pseudotumor cerebri	2.11E-02
Side effect	arachnoiditis	3.90E-02
Side effect	latent diabetes	3.97E-02
Side effect	infection	5.10E-02
Side effect	pathological fracture	5.65E-02
Side effect	abscess	5.65E-02
Side effect	papilledema	5.65E-02
Chemical Fragment	C=C(NC)NCC	2.45E-12
Chemical Fragment	C(=NCCCCCC)N	9.28E-10
Chemical Fragment	C(=NCCCC)N	9.38E-10
Chemical Fragment	C(=NCCCCCC)N	3.62E-09
Chemical Fragment	c1ccc(cc1)CC	1.87E-08
Chemical Fragment	C(=NCCCC)N	3.20E-08
Chemical Fragment	C(=NCCCC)N	4.62E-08
Chemical Fragment	C(=C(NCc1cnc(nc1(N))C)C)S	5.01E-08
Chemical Fragment	C(=NOC(C)C)c1nc(N)sc1	4.20E-07
Chemical Fragment	C=C1CCCC2(C)(C(CC)CCC12)	1.07E-06
Chemical Fragment	Cc1c(cccc1Cl)Cl	1.07E-06
Chemical Fragment	C(C)CCCC(C)C	1.48E-06
Chemical Fragment	C(c1ccccc1)c2ccccc2	5.73E-06
Chemical Fragment	C(C)CCCC(C)C	6.09E-06
Chemical Fragment	C(=NCCCCCC)N	6.70E-06
Chemical Fragment	C(=NOC)c1nc(N)sc1	1.88E-05
Chemical Fragment	C(=NCCCCC)N	4.12E-05
Chemical Fragment	C(c1ccccc1)CC	4.69E-05
Chemical Fragment	C(C)(C)CC(C)(C)C	4.69E-05
Chemical Fragment	C(=NCCCCCCN=CN)N	2.08E-04
Chemical Fragment	c1ccc(cc1)C[N+](C)C	2.42E-03
Chemical Fragment	CCc1ccc(cc1)Cl	2.42E-03
Chemical Fragment	C(NC)NCCS	2.42E-03

Drugs	# of cell lines	Drug annotation	Literature evidence
alclometasone	2	D07 corticosteroid, dermatological preparation	
beclometasone	2	D07 corticosteroid, dermatological preparation	
betamethasone	2	D07 corticosteroid, dermatological preparation	

budesonide	2	D07 corticosteroid, dermatological preparation	
dexamethasone	2	D07 corticosteroid, dermatological preparation	
diflorasone	2	D07 corticosteroid, dermatological preparation	
fludroxycortide	2	D07 corticosteroid, dermatological preparation	
flumetasone	2	D07 corticosteroid, dermatological preparation	
fluorometholone	2	D07 corticosteroid, dermatological preparation	
halcinonide	2	D07 corticosteroid, dermatological preparation	
methylprednisolone	2	D07 corticosteroid, dermatological preparation	
mometasone	2	D07 corticosteroid, dermatological preparation; glucocorticosteroid (ATC: D07AC13)	
prednisolone	2	D07 corticosteroid, dermatological preparation	
triamcinolone	2	D07 corticosteroid, dermatological preparation	
fludrocortisone	2	H02 Corticosteroid for systemic use	
rimexolone	2	H02 Corticosteroid for systemic use (ATC: H02AB12); cell cycle blocker	(Spies et al, 2010)
corticosterone	2	corticosteroid (steroid hormone)	
flunisolide	2	corticosteroid (ATC: R03BA03)	
fluticasone	2	glucocorticoid (synthetic, ATC: D07AC17)	
isoflupredone	2	antiinflammatory steroid	
simvastatin	2	hypolipidemic drug (ATC: C10AA01)	
tracazolate	2	anxiolytic	(Patel & Malick, 1982)
(+)-chelidonine	1	cell cycle blocker	(Panzer *et al*, 2001)
amylocaine	1	local anesthetic	
carbachol	1	acetylcholine receptor agonist (ATC: N07AB01)	
cyclic adenosine monophosphate	1	second messenger for intracellular signal transduction	
etynodiol	1	hormonal contraceptive (ATC: G03DC06)	
gabexate	1	serine protease inhibitor	(Yuksel *et al*, 2003)
haloperidol	1	antipsychotic (ATC:N05AD01)	
hemicholinium	1	indirect acetylcholine antagonist	(Sandberg & Coyle, 1985)
isosorbide	1	diuretic	(McLean *et al*, 1994)
letrozole	1	non-steroidal aromatase inhibitor (ATC: L02BG04)	
lithocholic acid	1	bile acid	
metergoline	1	dopamine agonist and serotonin antagonist (ATC: G02CB05)	(Hamon *et al*, 1981)
moracizine	1	antiarrhythmic (ATC: C01BG01)	
naloxone	1	opioid antagonist (ATC: V03AB15)	
phenylpropanolamine	1	psychoactive drug	
piretanide	1	diuretic (ATC: C03CA03)	
practolol	1	selective beta blocker (ATC: C07AB01)	
repaglinide	1	used to treat type II diabetes (ATC: A10BX02)	
ribavirin	1	anti-viral drug (ATC: J05AB04)	
rolipram	1	PDE4-inhibitor; anti-inflammatory drug	
terbutaline	1	ADRB2 agonist (ATC: R03AC03)	

CODIM 5

111 genes, 23 chemicals

Functional enrichment of genes:

**Annotation Cluster 1**	**Enrichment Score: 14.76**	
Category	Term	P-value
GOTERM_BP_FAT	GO:0006986~response to unfolded protein	6.64E-19
SP_PIR_KEYWORDS	stress response	2.10E-18
GOTERM_BP_FAT	GO:0051789~response to protein stimulus	1.52E-16
SP_PIR_KEYWORDS	Chaperone	2.07E-16
GOTERM_BP_FAT	GO:0006457~protein folding	1.71E-15

GOTERM_BP_FAT	GO:0010033~response to organic substance	5.82E-13
GOTERM_MF_FAT	GO:0051082~unfolded protein binding	9.75E-10
**Annotation Cluster 2**	**Enrichment Score: 3.62**	
Category	Term	P-value
SP_PIR_KEYWORDS	molecular chaperone	6.86E-11
INTERPRO	IPR013126:Heat shock protein 70	7.22E-06
INTERPRO	IPR001023:Heat shock protein Hsp70	7.22E-06
INTERPRO	IPR018181:Heat shock protein 70, conserved site	1.07E-05
KEGG_PATHWAY	hsa04612:Antigen processing and presentation	4.83E-04
PIR_SUPERFAMILY	PIRSF002581:chaperone HSP70	0.007261831
SP_PIR_KEYWORDS	ATP	0.073358956
KEGG_PATHWAY	hsa03040:Spliceosome	0.421685642
KEGG_PATHWAY	hsa04144:Endocytosis	0.581097351

Drug annotation:

No enriched terms found.

Drugs	# of cell lines	Drug annotation	Literature evidence
alvespimycin	3	HSP90 inhibitor	(Taldone *et al*, 2008)
geldanamycin	3	HSP90 inhibitor	(Miyata, 2005a)
monorden	3	HSP90 inhibitor	(Sharma *et al*, 1998)
tanespimycin	3	HSP90 inhibitor	
securinine	3	GABAA receptor antagonist	(Beutler *et al*, 1985)
15-delta prostaglandin J2	3	NSAID; inhibits cell proliferation	(Kim *et al*, 2007)
disulfiram	3	used to treat chronic alcoholism; proteasome inhibitor	(Cvek & Dvorak, 2008)
parthenolide	3	apoptosis/antiparasitic/anticancer/micrtubule interfering/anti-inflammatory; HDAC1 depletion	(López-Franco *et al*, 2006; Gopal *et al*, 2007)
thiostrepton	3	oligo-peptide antibiotic	
ebselen	2	antiinflammatory antioxidant	(Schewe, 1995)
LY-294002	2	PI3K inhibitor	(Maira *et al*, 2009)
oxyphenbutazone	2	anti-inflammatory (ATC: M01AA03)	
puromycin	2	antibiotic; protein synthesis inhibitor	
(+)-chelidonine	1	cell cycle blocker	(Panzer *et al*, 2001)
etacrynic acid	1	diuretic (ATC: C03CC01)	
mefloquine	1	quinine analogue; anti-malarial (ATC: P01BC02)	
mometasone	1	D07 corticosteroid, dermatological preparation; glucocorticosteroid (ATC: D07AC13)	
myricetin	1	flavanoid/anti-oxidant; HSP70 inhibitor	(Jinwal *et al*, 2009)
nifuroxazide	1	inhibits myeloma survival; antibiotic (ATC: A07AX03)	(Nelson *et al*, 2008)
oxolamine	1	anti-inflammatory/cough suppressant (ATC: R05DB07)	
scoulerine	1	GABAA receptor agonist	(Eisenreich *et al*, 2003)
Prestwick-642	1	used to treat cystic acne (ATC: D10AD04)	
Prestwick-674	1	oral anaesthetic (ATC: N01BX02)	

CODIM 6

75 genes, 21 chemicals

Functional enrichment of genes:

**Annotation Cluster 1**	**Enrichment Score: 1.42**	
Category	Term	P-value
UP_SEQ_FEATURE	domain:LIM zinc-binding 2	0.02025079
UP_SEQ_FEATURE	domain:LIM zinc-binding 1	0.02025079
SP_PIR_KEYWORDS	LIM domain	0.04782628
INTERPRO	IPR001781:Zinc finger, LIM-type	0.048255967
SMART	SM00132:LIM	0.078441179

Drug annotation:

Annotation type	Enriched term	p-value (FDR)
Chemical Fragment	C(=NOC(C)C)c1nc(N)sc1	9.47E-03

Drugs	# of cell lines	Drug annotation	Literature evidence
vorinostat	2	histone deacetylase inhibitor	
trichostatin A	2	histone deacetylase (HDAC, class I/II) inhibitor	(Vanhaecke *et al*, 2004)
fulvestrant	1	estrogen receptor antagonist (ATC: L02BA03)	
bufexamac	1	anti-inflammatory (ATC: M01AB17); HDAC inhibitor	(Bantscheff *et al*, 2011)
parthenolide	1	apoptosis/antiparasitic/anticancer/micrtubule interfering/anti-inflammatory; HDAC1 depletion	(López-Franco *et al*, 2006; Gopal *et al*, 2007)
alprostadil	1	prostaglandin; used to treat erectile dysfunction (ATC: G04BE01); increases phosphorylation of ADRB2 protein	(Davis *et al*, 2011)
tanespimycin	1	HSP90 inhibitor	
dilazep	1	adenosine reuptake inhibitor (ATC: C01DX10)	
ellipticine	1	antineoplastic agent, uncoupling agent	
geldanamycin	1	HSP90 inhibitor	(Miyata, 2005b)
hycanthone	1	cytotoxic; cell cycle blocker; inhibits RNA synthesis	(Sieber *et al*, 1973; Wong *et al*, 1990)
LY-294002	1	PI3K inhibitor	(Maira *et al*, 2009)
thiostrepton	1	oligo-peptide antibiotic	
15-delta prostaglandin J2	1	NSAID; inhibits cell proliferation	(Kim *et al*, 2007)
protriptyline	1	N06 psychoanaleptic	
monorden	1	HSP90 inhibitor	(Sharma *et al*, 1998)
raloxifene	1	selective estrogen receptor modulator (ATC: G03XC01)	
solasodine	1	glycoalkaloid	
syrosingopine	1		
corticosterone	1	corticosteroid (steroid hormone)	
alvespimycin	1	HSP90 inhibitor	(Taldone *et al*, 2008)

CODIM 7

74 genes, 20 chemicals

Functional enrichment of genes:

**Annotation Cluster 1**	**Enrichment Score: 1.88**	
Category	Term	P-value
SP_PIR_KEYWORDS	rna-binding	0.001024947
GOTERM_MF_FAT	GO:0003723~RNA binding	0.002184082
SMART	SM00360:RRM	0.005501749
INTERPRO	IPR000504:RNA recognition motif, RNP-1	0.005810571
INTERPRO	IPR012677:Nucleotide-binding, alpha-beta plait	0.006337525
UP_SEQ_FEATURE	domain:RRM 1	0.11692093
UP_SEQ_FEATURE	domain:RRM 2	0.11692093
UP_SEQ_FEATURE	domain:RRM	0.130687405
**Annotation Cluster 2**	**Enrichment Score: 1.40**	
Category	Term	P-value
KEGG_PATHWAY	hsa00240:Pyrimidine metabolism	0.016908657
KEGG_PATHWAY	hsa00230:Purine metabolism	0.04279547
GOTERM_BP_FAT	GO:0006259~DNA metabolic process	0.084506223
**Annotation Cluster 3**	**Enrichment Score: 1.39**	
Category	Term	P-value

GOTERM_BP_FAT	GO:0006396~RNA processing	6.02E-04
GOTERM_BP_FAT	GO:0006397~mRNA processing	0.016105021
SP_PIR_KEYWORDS	mrna processing	0.02283707
GOTERM_BP_FAT	GO:0016071~mRNA metabolic process	0.028036716
GOTERM_BP_FAT	GO:0008380~RNA splicing	0.03948499
SP_PIR_KEYWORDS	mrna splicing	0.045741516
GOTERM_BP_FAT	GO:0000375~RNA splicing, via transesterification reactions	0.300744596
GOTERM_BP_FAT	GO:0000377~RNA splicing, via transesterification reactions with bulged adenosine as nucleophile	0.300744596
GOTERM_BP_FAT	GO:0000398~nuclear mRNA splicing, via spliceosome	0.300744596

Drug annotation:

**Annotation type**	**Enriched term**	**p-value (FDR)**
Drug target	CYP1B1;cytochrome P450, family 1, subfamily B, polypeptide 1	1.14E-02
Chemical Fragment	C(=NCCCCCC)N	1.84E-04

**Drugs**	**# of cell lines**	**Drug annotation**	**Literature evidence**
luteolin	3	flavonoid; controls nucleic acid syntheses	(Harris *et al*, 2012)
apigenin	3	flavonoid	
acacetin	3	flavonoid	
harmol	2	metabolite of harmine that binds to yeast RNA	(Nafisi *et al*, 2010)
nifedipine	2	calcium channel blocker (ATC: C08CA05); decreases RNA synthesis	(Lijnen *et al*, 1998)
nocodazole	2	anti-neoplastic agent; depolymerizes microtubules	
harmine	2	fluorescent harmala alkaloid; inhibits monoamine oxidase A; binds to yeast RNA	(Nafisi *et al*, 2010)
atovaquone	1	analog of ubiquinone (ATC: P01AX06)	
simvastatin	1	hypolipidemic drug (ATC: C10AA01)	
harman	1	monoamine oxidase inhibitor; binds to yeast RNA	(Nafisi *et al*, 2010)
kaempferol	1	flavonoid; decreases RNA synthesis	(Kanakis *et al*, 2006; Nose, 1984)
quercetin	1	flavonoid; decreases RNA synthesis	(Kanakis *et al*, 2006; Nose, 1984)
minoxidil	1	vasodilator (ATC: D11AX01)	
meptazinol	1	opoid and acetylcholinesterase inhibitor (ATC: N02AX05)	
hycanthone	1	cytotoxic; cell cycle blocker; inhibits RNA synthesis	(Sieber *et al*, 1973; Wong *et al*, 1990)
nystatin	1	antifungal (ATC: D01AA01)	
ellipticine	1	antineoplastic agent, uncoupling agent	
cyclopenthiazide	1	diuretic (ATC: C03AA07)	
canavanine	1	non-proteinogenic α-amino acid found in certain leguminous plants	(McMahon & Langstroth, 1972)
molindone	1	antipsychotic (ATC: N05AE02)	

<u>CODIM 8</u>

115 genes, 32 chemicals

Functional enrichment of genes:

**Annotation Cluster 1**	**Enrichment Score: 4.16**	
Category	Term	P-value
GOTERM_CC_FAT	GO:0005576~extracellular region	6.40E-06
SP_PIR_KEYWORDS	signal	2.53E-05
UP_SEQ_FEATURE	signal peptide	2.59E-05
SP_PIR_KEYWORDS	Secreted	2.79E-05
SP_PIR_KEYWORDS	glycoprotein	8.35E-05
UP_SEQ_FEATURE	disulfide bond	2.69E-04
UP_SEQ_FEATURE	glycosylation site:N-linked (GlcNAc...)	3.43E-04
SP_PIR_KEYWORDS	disulfide bond	5.42E-04

Annotation Cluster 2	Enrichment Score: 1.95	
Category	Term	P-value
GOTERM_BP_FAT	GO:0048839~inner ear development	0.004380241
GOTERM_BP_FAT	GO:0043583~ear development	0.00938752
GOTERM_BP_FAT	GO:0007423~sensory organ development	0.033885801
**Annotation Cluster 3**	**Enrichment Score: 1.35**	
Category	Term	P-value
GOTERM_CC_FAT	GO:0044421~extracellular region part	7.30E-04
GOTERM_CC_FAT	GO:0031012~extracellular matrix	0.016885878
GOTERM_CC_FAT	GO:0005578~proteinaceous extracellular matrix	0.039250179
GOTERM_BP_FAT	GO:0007155~cell adhesion	0.06362705
GOTERM_BP_FAT	GO:0022610~biological adhesion	0.06362705
SP_PIR_KEYWORDS	extracellular matrix	0.255837168
SP_PIR_KEYWORDS	cell adhesion	0.605670239

Drug annotation:

No enriched terms found.

CODIM 9

176 genes, 56 chemicals

Functional enrichment of genes:

Annotation Cluster 1	Enrichment Score: 4.60	
Category	Term	P-value
GOTERM_CC_FAT	GO:0031981~nuclear lumen	9.54E-07
GOTERM_CC_FAT	GO:0005654~nucleoplasm	7.86E-06
GOTERM_CC_FAT	GO:0070013~intracellular organelle lumen	1.36E-05
GOTERM_CC_FAT	GO:0031974~membrane-enclosed lumen	1.63E-05
GOTERM_CC_FAT	GO:0043233~organelle lumen	2.19E-05
GOTERM_CC_FAT	GO:0005730~nucleolus	0.006116859
**Annotation Cluster 2**	**Enrichment Score: 2.16**	
Category	Term	P-value
SP_PIR_KEYWORDS	Rotamase	9.73E-04
GOTERM_MF_FAT	GO:0003755~peptidyl-prolyl cis-trans isomerase activity	0.001343264
GOTERM_MF_FAT	GO:0016859~cis-trans isomerase activity	0.001585145
SP_PIR_KEYWORDS	Isomerase	0.003811761
INTERPRO	IPR002130:Peptidyl-prolyl cis-trans isomerase, cyclophilin-type	0.02067277
UP_SEQ_FEATURE	domain:PPIase cyclophilin-type	0.022566346
GOTERM_MF_FAT	GO:0008144~drug binding	0.036267974
GOTERM_BP_FAT	GO:0006457~protein folding	0.039090865
**Annotation Cluster 3**	**Enrichment Score: 2.12**	
Category	Term	P-value
SP_PIR_KEYWORDS	rna-binding	1.65E-05
UP_SEQ_FEATURE	domain:RRM 2	0.013041137
UP_SEQ_FEATURE	domain:RRM 1	0.013041137
INTERPRO	IPR000504:RNA recognition motif, RNP-1	0.018483294
INTERPRO	IPR012677:Nucleotide-binding, alpha-beta plait	0.020470083
UP_SEQ_FEATURE	domain:RRM 3	0.029104132
SMART	SM00360:RRM	0.041657864
**Annotation Cluster 4**	**Enrichment Score: 2.06**	
Category	Term	P-value
SP_PIR_KEYWORDS	dna-binding	7.39E-04
SP_PIR_KEYWORDS	transcription regulation	0.002432614
GOTERM_MF_FAT	GO:0003677~DNA binding	0.004340302
SP_PIR_KEYWORDS	Transcription	0.00444635
GOTERM_BP_FAT	GO:0006350~transcription	0.004866742
GOTERM_BP_FAT	GO:0045449~regulation of transcription	0.00989707
GOTERM_MF_FAT	GO:0030528~transcription regulator activity	0.024243333
GOTERM_BP_FAT	GO:0051252~regulation of RNA metabolic process	0.059107977

GOTERM_BP_FAT	GO:0006355~regulation of transcription, DNA-dependent	0.105091817

Drug annotation:
No enriched terms found.

CODIM 10
51 genes, 55 chemicals
Functional enrichment of genes:

**Annotation Cluster 1**	**Enrichment Score: 2.91**	
Category	Term	P-value
INTERPRO	IPR019782:WD40 repeat 2	4.68E-04
UP_SEQ_FEATURE	repeat:WD 3	5.72E-04
INTERPRO	IPR017986:WD40 repeat, region	5.92E-04
INTERPRO	IPR019781:WD40 repeat, subgroup	6.39E-04
UP_SEQ_FEATURE	repeat:WD 1	6.65E-04
UP_SEQ_FEATURE	repeat:WD 2	6.65E-04
SP_PIR_KEYWORDS	wd repeat	6.68E-04
INTERPRO	IPR001680:WD40 repeat	9.77E-04
INTERPRO	IPR019775:WD40 repeat, conserved site	0.001170208
SMART	SM00320:WD40	0.001624547
INTERPRO	IPR015943:WD40/YVTN repeat-like	0.001664433
UP_SEQ_FEATURE	repeat:WD 5	0.002967375
UP_SEQ_FEATURE	repeat:WD 4	0.003871282
UP_SEQ_FEATURE	repeat:WD 6	0.013103737
**Annotation Cluster 2**	**Enrichment Score: 2.29**	
Category	Term	P-value
GOTERM_CC_FAT	GO:0044430~cytoskeletal part	0.00125971
GOTERM_CC_FAT	GO:0005856~cytoskeleton	0.004753477
GOTERM_CC_FAT	GO:0043232~intracellular non-membrane-bounded organelle	0.010529529
GOTERM_CC_FAT	GO:0043228~non-membrane-bounded organelle	0.010529529

Drug annotation:
No enriched terms found.

CODIM 11
154 genes, 66 chemicals
Functional enrichment of genes:

**Annotation Cluster 1**	**Enrichment Score: 3.09**	
Category	Term	P-value
GOTERM_BP_FAT	GO:0046907~intracellular transport	1.39E-06
GOTERM_BP_FAT	GO:0034613~cellular protein localization	1.80E-05
GOTERM_BP_FAT	GO:0070727~cellular macromolecule localization	1.95E-05
GOTERM_BP_FAT	GO:0006886~intracellular protein transport	2.96E-05
GOTERM_BP_FAT	GO:0008104~protein localization	3.00E-04
GOTERM_BP_FAT	GO:0015031~protein transport	4.32E-04
GOTERM_BP_FAT	GO:0045184~establishment of protein localization	4.79E-04
GOTERM_BP_FAT	GO:0006605~protein targeting	6.91E-04
GOTERM_BP_FAT	GO:0006913~nucleocytoplasmic transport	0.002762323
GOTERM_BP_FAT	GO:0051169~nuclear transport	0.002942796
GOTERM_BP_FAT	GO:0017038~protein import	0.006407508
GOTERM_BP_FAT	GO:0006606~protein import into nucleus	0.007359192
GOTERM_BP_FAT	GO:0051170~nuclear import	0.007972003
GOTERM_BP_FAT	GO:0033365~protein localization in organelle	0.009997456
GOTERM_BP_FAT	GO:0034504~protein localization in nucleus	0.010008898
SP_PIR_KEYWORDS	protein transport	0.178793179
**Annotation Cluster 2**	**Enrichment Score: 2.06**	
Category	Term	P-value
GOTERM_BP_FAT	GO:0051674~localization of cell	0.001738566
GOTERM_BP_FAT	GO:0048870~cell motility	0.001738566

GOTERM_BP_FAT	GO:0006928~cell motion	0.0035237
GOTERM_BP_FAT	GO:0016477~cell migration	0.011982647
GOTERM_BP_FAT	GO:0030036~actin cytoskeleton organization	0.052259267
GOTERM_BP_FAT	GO:0030029~actin filament-based process	0.06519111

Drug annotation:

No enriched terms found.

CODIM 12

106 genes, 118 chemicals

Functional enrichment of genes:

**Annotation Cluster 1**	**Enrichment Score: 1.33**	
Category	Term	P-value
SP_PIR_KEYWORDS	isopeptide bond	0.017434813
SP_PIR_KEYWORDS	ubl conjugation	0.024665376
UP_SEQ_FEATURE	cross-link:Glycyl lysine isopeptide (Lys-Gly) (interchain with G-Cter in ubiquitin)	0.235862924

Drug annotation:

**Annotation type**	**Enriched term**	**p-value (FDR)**
Side Effect	exfoliative dermatitis	9.96E-02

CODIM 13

53 genes, 41 chemicals

Functional enrichment of genes:

**Annotation Cluster 1**	**Enrichment Score: 10.32**	
Category	Term	P-value
UP_SEQ_FEATURE	region of interest:Alpha	5.55E-14
UP_SEQ_FEATURE	region of interest:Beta	5.55E-14
UP_SEQ_FEATURE	metal ion-binding site:Divalent metal cation; cluster B	1.22E-13
UP_SEQ_FEATURE	metal ion-binding site:Divalent metal cation; cluster A	1.22E-13
INTERPRO	IPR018064:metallothionein, vertebrate, metal binding site	1.66E-13
SP_PIR_KEYWORDS	metal-thiolate cluster	2.34E-13
PIR_SUPERFAMILY	PIRSF002564:metallothionein	2.71E-13
INTERPRO	IPR003019:metallothionein superfamily, eukaryotic	3.30E-13
INTERPRO	IPR000006:metallothionein, vertebrate	3.30E-13
SP_PIR_KEYWORDS	cadmium	5.96E-12
SP_PIR_KEYWORDS	chelation	5.96E-12
GOTERM_MF_FAT	GO:0046870~cadmium ion binding	5.34E-11
SP_PIR_KEYWORDS	metal binding	1.21E-09
SP_PIR_KEYWORDS	copper	4.04E-07
GOTERM_MF_FAT	GO:0005507~copper ion binding	2.08E-06
SP_PIR_KEYWORDS	acetylated amino end	6.67E-06
SP_PIR_KEYWORDS	acetylation	0.039761968
**Annotation Cluster 2**	**Enrichment Score: 4.56**	
Category	Term	P-value
SP_PIR_KEYWORDS	zinc	5.26E-08
GOTERM_MF_FAT	GO:0008270~zinc ion binding	6.71E-07
GOTERM_MF_FAT	GO:0046914~transition metal ion binding	1.53E-05
SP_PIR_KEYWORDS	metal-binding	3.38E-05
GOTERM_MF_FAT	GO:0046872~metal ion binding	7.15E-04
GOTERM_MF_FAT	GO:0043169~cation binding	8.29E-04
GOTERM_MF_FAT	GO:0043167~ion binding	0.00104236

Drug annotation:

**Annotation type**	**Enriched term**	**p-value (FDR)**
Drug target	SERPINE1; serpin peptidase inhibitor, clade E (nexin, plasminogen activator inhibitor type 1), member 1	3.47E-02

CODIM 14

91 genes, 52 chemicals

Functional enrichment of genes:

Annotation Cluster 1	Enrichment Score: 13.35	
Category	Term	P-value
SP_PIR_KEYWORDS	endoplasmic reticulum	1.43E-24
GOTERM_CC_FAT	GO:0005783~endoplasmic reticulum	6.25E-20
GOTERM_CC_FAT	GO:0044432~endoplasmic reticulum part	1.72E-16
GOTERM_CC_FAT	GO:0005789~endoplasmic reticulum membrane	3.84E-11
GOTERM_CC_FAT	GO:0042175~nuclear envelope-endoplasmic reticulum network	1.02E-10
GOTERM_CC_FAT	GO:0012505~endomembrane system	6.60E-10
GOTERM_CC_FAT	GO:0031090~organelle membrane	8.81E-06
**Annotation Cluster 2**	**Enrichment Score: 5.73**	
Category	Term	P-value
GOTERM_CC_FAT	GO:0012505~endomembrane system	6.60E-10
GOTERM_BP_FAT	GO:0015031~protein transport	1.87E-07
GOTERM_BP_FAT	GO:0045184~establishment of protein localization	2.18E-07
SP_PIR_KEYWORDS	er-golgi transport	4.26E-07
GOTERM_BP_FAT	GO:0006886~intracellular protein transport	6.53E-07
GOTERM_BP_FAT	GO:0046907~intracellular transport	1.28E-06
GOTERM_BP_FAT	GO:0008104~protein localization	1.34E-06
GOTERM_BP_FAT	GO:0034613~cellular protein localization	1.95E-06
GOTERM_BP_FAT	GO:0070727~cellular macromolecule localization	2.18E-06
GOTERM_CC_FAT	GO:0031090~organelle membrane	8.81E-06
SP_PIR_KEYWORDS	protein transport	5.74E-05
GOTERM_BP_FAT	GO:0016192~vesicle-mediated transport	9.64E-05
SP_PIR_KEYWORDS	transport	0.001130058
**Annotation Cluster 3**	**Enrichment Score: 5.42**	
Category	Term	P-value
UP_SEQ_FEATURE	topological domain:Lumenal	1.16E-12
SP_PIR_KEYWORDS	membrane	7.28E-06
UP_SEQ_FEATURE	transmembrane region	7.55E-06
SP_PIR_KEYWORDS	transmembrane	8.49E-06
UP_SEQ_FEATURE	topological domain:Cytoplasmic	2.37E-05
GOTERM_CC_FAT	GO:0031224~intrinsic to membrane	8.63E-04
GOTERM_CC_FAT	GO:0016021~integral to membrane	9.16E-04
**Annotation Cluster 4**	**Enrichment Score: 5.41**	
Category	Term	P-value
INTERPRO	IPR000348:emp24/gp25L/p24	3.97E-07
UP_SEQ_FEATURE	domain:GOLD	3.36E-06
INTERPRO	IPR009038:GOLD	3.62E-06
GOTERM_CC_FAT	GO:0005793~ER-Golgi intermediate compartment	4.61E-05
**Annotation Cluster 5**	**Enrichment Score: 3.61**	
Category	Term	P-value
UP_SEQ_FEATURE	short sequence motif:Prevents secretion from ER	2.02E-11
GOTERM_CC_FAT	GO:0005788~endoplasmic reticulum lumen	3.21E-07
INTERPRO	IPR000886:Endoplasmic reticulum, targeting sequence	2.96E-05
SP_PIR_KEYWORDS	Isomerase	1.27E-04
GOTERM_CC_FAT	GO:0048770~pigment granule	3.86E-04
GOTERM_CC_FAT	GO:0042470~melanosome	3.86E-04
GOTERM_CC_FAT	GO:0043233~organelle lumen	0.905523772
GOTERM_CC_FAT	GO:0031974~membrane-enclosed lumen	0.919959277
GOTERM_CC_FAT	GO:0070013~intracellular organelle lumen	0.937638533

Drug annotation:

Annotation type	Enriched term	p-value (FDR)

Chemical Fragment	c1c(cc(cc1I)I)I	7.11E-05
Chemical Fragment	Cc1c(cccc1Cl)Cl	4.31E-04

CODIM 15

55 genes, 48 chemicals

Functional enrichment of genes:

**Annotation Cluster 1**	**Enrichment Score: 2.54**	
Category	Term	P-value
GOTERM_BP_FAT	GO:0006563~L-serine metabolic process	9.28E-04
KEGG_PATHWAY	hsa00260:Glycine, serine and threonine metabolism	0.004319529
GOTERM_BP_FAT	GO:0009069~serine family amino acid metabolic process	0.005756289
**Annotation Cluster 2**	**Enrichment Score: 2.00**	
Category	Term	P-value
SP_PIR_KEYWORDS	Aminoacyl-tRNA synthetase	2.25E-05
KEGG_PATHWAY	hsa00970:Aminoacyl-tRNA biosynthesis	3.21E-05
GOTERM_MF_FAT	GO:0016875~ligase activity, forming carbon-oxygen bonds	4.25E-05
GOTERM_MF_FAT	GO:0016876~ligase activity, forming aminoacyl-tRNA and related compounds	4.25E-05
GOTERM_MF_FAT	GO:0004812~aminoacyl-tRNA ligase activity	4.25E-05
GOTERM_BP_FAT	GO:0043038~amino acid activation	5.85E-05
GOTERM_BP_FAT	GO:0043039~tRNA aminoacylation	5.85E-05
GOTERM_BP_FAT	GO:0006418~tRNA aminoacylation for protein translation	5.85E-05
GOTERM_BP_FAT	GO:0006399~tRNA metabolic process	0.002320177
GOTERM_MF_FAT	GO:0000049~tRNA binding	0.002875221
SP_PIR_KEYWORDS	ligase	0.006586294
INTERPRO	IPR014729:Rossmann-like alpha/beta/alpha sandwich fold	0.013913194
SP_PIR_KEYWORDS	protein biosynthesis	0.014425618
GOTERM_BP_FAT	GO:0034660~ncRNA metabolic process	0.027120096
GOTERM_BP_FAT	GO:0006412~translation	0.076127375
GOTERM_MF_FAT	GO:0003723~RNA binding	0.201133504
GOTERM_MF_FAT	GO:0032555~purine ribonucleotide binding	0.28648941
GOTERM_MF_FAT	GO:0032553~ribonucleotide binding	0.28648941
SP_PIR_KEYWORDS	nucleotide-binding	0.319792205
GOTERM_MF_FAT	GO:0017076~purine nucleotide binding	0.332015222
GOTERM_MF_FAT	GO:0005524~ATP binding	0.381221624
GOTERM_MF_FAT	GO:0032559~adenyl ribonucleotide binding	0.389970012
GOTERM_MF_FAT	GO:0000166~nucleotide binding	0.399117334
SP_PIR_KEYWORDS	atp-binding	0.43133076
GOTERM_MF_FAT	GO:0030554~adenyl nucleotide binding	0.441515712
GOTERM_MF_FAT	GO:0001883~purine nucleoside binding	0.454652627
GOTERM_MF_FAT	GO:0001882~nucleoside binding	0.462299836

Drug annotation:

**Annotation type**	**Enriched term**	**p-value**
Drug target	SLC6A2; solute carrier family 6 (neurotransmitter transporter, noradrenalin), member 2	3.87E-03
Drug target	HRH1; histamine receptor H1	3.87E-03
Drug target	HTR2A; 5-hydroxytryptamine (serotonin) receptor 2A	4.58E-03
Drug target	ADCY1; adenylate cyclase 1 (brain)	6.13E-03
Drug target	SLC6A4; solute carrier family 6 (neurotransmitter transporter, serotonin), member 4	8.45E-03
Drug target	DRD1; dopamine receptor D1	1.10E-02
Drug target	ADRA1A; adrenergic, alpha-1A-, receptor	1.45E-02
Drug target	DRD2; dopamine receptor D2	1.45E-02
Drug target	CHRM2; cholinergic receptor, muscarinic 2	2.22E-02
Drug target	CHRM5; cholinergic receptor, muscarinic 5	2.40E-02
Drug target	DRD3; dopamine receptor D3	2.72E-02
Drug target	CHRM4; cholinergic receptor, muscarinic 4	2.87E-02
Drug target	HTR2C; 5-hydroxytryptamine (serotonin) receptor 2C	6.47E-02
Drug target	CHRM1; cholinergic receptor, muscarinic 1	6.53E-02
Drug target	CYP1B1; cytochrome P450, family 1, subfamily B, polypeptide 1	8.95E-02

Side effect	paralytic ileus	3.18E-04
Side effect	breast enlargement	3.33E-04
Side effect	galactorrhea	4.41E-04
Side effect	weight gain	2.13E-03
Side effect	testicular swelling	6.19E-03
Side effect	gynecomastia	1.83E-02
Side effect	adenitis	7.94E-02
Side effect	agitation	9.23E-02
Chemical Fragment	c1ccc(cc1)CN2CCN(C)CC2	1.85E-03

**Drugs**	**# of cell lines**	**Drug annotation**	**Literature evidence**
methylbenzethonium chloride	2	induces apoptosis	(Yip *et al*, 2006)
astemizole	2	histamine H1-receptor antagonist	
ciclosporin	2	immunosuppressant drug (ATC: L04AD01)	
loperamide	2	opioid drug used against diarrhea (ATC: A07DA03)	
alexidine	2	disinfectant	
gossypol	2	antiproliferative activity	(Le Blanc *et al*, 2002)
mefloquine	2	quinine analogue; anti-malarial (ATC: P01BC02)	
niclosamide	2	teniacide (ATC: P02DA01)	
pyrvinium	2	inhibits proliferation in colon cancer; antiparasitic (ATC: P02CX01)	
15-delta prostaglandin J2	1	NSAID; inhibits cell proliferation	(Kim *et al*, 2007)
nifuroxazide	1	inhibits myeloma survival; antibiotic (ATC: A07AX03)	(Nelson *et al*, 2008)
econazole	1	antifungal (ATC: D01AC03)	
desipramine	1	N06 psychoanaleptic	
mometasone	1	D07 corticosteroid, dermatological preparation; glucocorticosteroid (ATC: D07AC13)	
metixene	1	N04 anti-parkinson drug	
chlorpromazine	1	N05 psycholeptic	
perphenazine	1	N05 psycholeptic	
pimozide	1	N05 psycholeptic	
prochlorperazine	1	N05 psycholeptic	
thioridazine	1	cytogenetic damage; inhibits cell proliferation; antipsychotic drug (ATC: N05AC02)	(Strobl *et al*, 1990)
maprotiline	1	N06 psychoanaleptic	
trimipramine	1	N06 psychoanaleptic	
norcyclobenzaprine	1	structurally similar to tricyclic antidepressants	(Van Hoey)
suloctidil	1	vasodilator (ATC: C04AX19)	
abamectin	1	insecticide (ATCvet: QP54AA02)	
amiodarone	1	antiarrhythmic agent (ATC: C01BD01)	
azacyclonol	1	ataractive	(Braun *et al*, 1956)
bepridil	1	calcium channel blocker (ATC: C08EA02)	
butoconazole	1	antifungal (ATC: G01AF15)	
clioquinol	1	cell cycle blocker; antifungal (ATC: G01AC02)	(Zhai *et al*, 2010)
cyclobenzaprine	1	muscle relaxant (ATC: M03BX08)	
deptropine	1	antihistamine with anticholinergic properties (ATC: R06AX16)	
dequalinium chloride	1	anticarcinogenic activity; antiseptic (ATC: D08AH01)	(Weiss *et al*, 1987)
hexetidine	1	antibacterial and antifungal (ATC: A01AB12)	
ivermectin	1	antiparasitic avermectin (ATC: P02CF01)	
lasalocid	1	antibacterial (ATCvet: QP51AH02)	
meclozine	1	antihistamine (ATC: R06AE05)	
miconazole	1	antifungal (ATC: D01AC02)	
naftifine	1	antifungal (ATC: D01AE22)	
pizotifen	1	prevents vascular headache (ATC: N02CX01)	

prenylamine	1	calcium channel blocker (ATC: C01DX02)	
quinisocaine	1	antipruritic (ATC: D04AB05)	
tamoxifen	1	estrogen receptor antagonist (ATC: L02BA01)	
thiethylperazine	1	antiemetic (ATC: R06AD03); potential antipsychotic activity	
thioguanosine	1	cell cycle blocker; guanine analog	(Lepage, 1963)
ticlopidine	1	antiplatelet drug (ATC: B01AC05)	
tonzonium bromide	1	mono-catioinc detergent	
tribenoside	1	vasoprotective (ATC: C05AX05)	

CODIM 16

66 genes, 31 chemicals

Functional enrichment of genes:

Annotation Cluster 1	Enrichment Score: 3.68	
Category	Term	P-value
GOTERM_CC_FAT	GO:0031981~nuclear lumen	9.84E-06
GOTERM_CC_FAT	GO:0005730~nucleolus	1.31E-05
GOTERM_CC_FAT	GO:0070013~intracellular organelle lumen	1.61E-05
GOTERM_CC_FAT	GO:0043233~organelle lumen	2.17E-05
GOTERM_CC_FAT	GO:0031974~membrane-enclosed lumen	3.05E-05
SP_PIR_KEYWORDS	nucleus	0.001569641
GOTERM_CC_FAT	GO:0043232~intracellular non-membrane-bounded organelle	0.037376461
GOTERM_CC_FAT	GO:0043228~non-membrane-bounded organelle	0.037376461
**Annotation Cluster 2**	**Enrichment Score: 1.72**	
Category	Term	P-value
GOTERM_BP_FAT	GO:0034660~ncRNA metabolic process	0.003014102
GOTERM_BP_FAT	GO:0034470~ncRNA processing	0.010516674
GOTERM_BP_FAT	GO:0006399~tRNA metabolic process	0.012784712
GOTERM_BP_FAT	GO:0009451~RNA modification	0.017050849
GOTERM_BP_FAT	GO:0006396~RNA processing	0.035325317
UP_SEQ_FEATURE	active site:Nucleophile	0.036743114
GOTERM_BP_FAT	GO:0008033~tRNA processing	0.040385229
SP_PIR_KEYWORDS	trna processing	0.047902076

Drug annotation:

Annotation type	Enriched term	p-value
Drug target	HRH1; histamine receptor H1	3.87E-03
Drug target	KCNH2; potassium voltage-gated channel, subfamily H (eag-related), member 2	7.12E-03
Drug target	CYP2D6; cytochrome P450, family 2, subfamily D, polypeptide 6	1.45E-02
Drug target	CALM1; calmodulin 1 (phosphorylase kinase, delta)	3.80E-02
Drug target	CYP3A4; cytochrome P450, family 3, subfamily A, polypeptide 4	4.67E-02
Drug target	HTR2A;5-hydroxytryptamine (serotonin) receptor 2A	4.77E-02
Drug target	ABCB1; ATP-binding cassette, sub-family B (MDR/TAP), member 1	6.47E-02
Side effect	paralytic ileus	3.12E-03
Side effect	galactorrhea	3.96E-02
Chemical Fragment	C=C(NC)NCC	2.34E-05
Chemical Fragment	C(=NCCCC)N	7.00E-05
Chemical Fragment	C(=NCCCCCC)N	1.13E-04
Chemical Fragment	C(=NCCCC)N	3.02E-04
Chemical Fragment	c1ccc(cc1)CC	4.40E-04
Chemical Fragment	C(=NCCCCC)N	4.63E-04
Chemical Fragment	C(=C(NCc1cnc(nc1(N))C)C)S	6.77E-04
Chemical Fragment	C(=NOC(C)C)c1nc(N)sc1	2.42E-03
Chemical Fragment	C(=NOC(C)C)c1nc(N)sc1	3.11E-03
Chemical Fragment	C=C1CCCC2(C)(C(CC)CCC12)	6.56E-03
Chemical Fragment	Cc1c(cccc1Cl)Cl	6.56E-03

Drugs	# of cell lines	Drug annotation	Literature evidence
diphenhydramine	2	antihistamine (ATC: D04AA32) possessing anticholinergic, antitussive, antiemetic, and sedative properties	

LY-294002	2	PI3K inhibitor	(Maira *et al*, 2009)
sirolimus	2	inhibits proliferation; immunosuppressant (ATC: L04AA10)	(Wang *et al*, 2007)
benzamil	1	ENaC channel blocker	(Chalfant *et al*, 1996)
viomycin	1	antibiotic, exhibits anti-tuberculosis properties	
amantadine	1	antiviral and antiparkinsonian (ATC: N04BB01)	
cefadroxil	1	bactericidal antibiotic (ATC: J01DB05)	
ciclopirox	1	cell cycle blocker; antifungal (ATC: D01AE14)	(Zhou *et al*, 2010)
cloxacillin	1	antibiotic (ATC: J01CF02)	
finasteride	1	5-alpha-reductase inhibitor (ATC: G04CB01)	
glimepiride	1	sulfonylurea antidiabetic (ATC: A10BB12)	
hydroflumethiazide	1	diuretic (ATC: C03AA02)	
latamoxef	1	oxacephem antibiotic (ATC: J01DD06)	
loperamide	1	opioid drug used against diarrhea (ATC: A07DA03)	
lovastatin	1	cholesterol-lowering drug (ATC: C10AA02)	
lycorine	1	toxic alkaloid	
maprotiline	1	N06 psychoanaleptic	
metitepine	1	antipsychotic	(Monachon *et al*, 1972)
methylbenzethonium chloride	1	induces apoptosis	(Yip *et al*, 2006)
metixene	1	N04 anti-parkinson drug	
naftidrofuryl	1	spasmolytic (ATC: C04AX21)	
perhexiline	1	antianginal agent (ATC: C08EX02)	
phenazopyridine	1	local analgesic effect (ATC: G04BX06)	
syrosingopine	1		
thiethylperazine	1	antiemetic (ATC: R06AD03); potential antipsychotic activity	(Rotrosen *et al*, 1978)
trimipramine	1	N06 psychoanaleptic	
triprolidine	1	antihistamine with anticholinergic properties (ATC: R06AX07)	
troglitazone	1	antidiabetic (ATC: A10BG01); peroxisome proliferator-activated receptor activator	
lactobionic acid	1	antioxidant	
rosiglitazone	1	antidiabetic drug (ATC: A10BG02); peroxisome proliferator-activated receptor activator	
norcyclobenzaprine	1	structurally similar to tricyclic antidepressants	(Van Hoey)

CODIM 17

37 genes, 9 chemicals

Functional enrichment of genes:

**Annotation Cluster 1**	**Enrichment Score: 1.84**	
Category	Term	P-value
UP_SEQ_FEATURE	repeat:WD 4	4.60E-03
UP_SEQ_FEATURE	repeat:WD 3	5.78E-03
UP_SEQ_FEATURE	repeat:WD 1	6.25E-03
UP_SEQ_FEATURE	repeat:WD 2	6.25E-03
SP_PIR_KEYWORDS	wd repeat	6.35E-03
INTERPRO	IPR019775:WD40 repeat, conserved site	6.78E-03
INTERPRO	IPR019782:WD40 repeat 2	2.45E-02
INTERPRO	IPR017986:WD40 repeat, region	2.71E-02
INTERPRO	IPR019781:WD40 repeat, subgroup	2.94E-02
INTERPRO	IPR001680:WD40 repeat	3.70E-02
SMART	SM00320:WD40	4.78E-02
INTERPRO	IPR015943:WD40/YVTN repeat-like	4.78E-02

Drug annotation:

**Annotation type**	**Enriched term**	**p-value (FDR)**

ATC code (2nd level)	C01; Cardiac therapy	8.01E-02

Drugs	# of cell lines	Drug annotation	Literature evidence
digoxigenin	2	Na+/K+-ATPase inhibitor	(Yoda, 1974)
helveticoside	2	Na+/K+-ATPase inhibitor	(Yoda, 1974)
digoxin	2	Na+/K+-ATPase inhibitor; cardiac glycoside (ATC: C01AA05)	
lanatoside C	2	Na+/K+-ATPase inhibitor; cardiac glycoside (ATC: C01AA06)	
ouabain	2	Na+/K+-ATPase inhibitor; cardiac glycoside (ATC: C01AC01)	
strophanthidin	2	Na+/K+-ATPase inhibitor	(Deitmer & Ellis, 1978)
digitoxigenin	2	Na+/K+-ATPase inhibitor	(Erlenkamp *et al*, 1998)
bisacodyl	1	Na+/K+-ATPase inhibitor; stimulant laxative drug (ATC: A06AB02)	(Schreiner *et al*, 1980)
ellipticine	1	antineoplastic agent, uncoupling agent	

CODIM 18

86 genes, 285 chemicals

Functional enrichment of genes:

Annotation Cluster 1	Enrichment Score: 3.41	
Category	Term	P-value
SP_PIR_KEYWORDS	membrane	4.04E-05
UP_SEQ_FEATURE	transmembrane region	1.03E-04
SP_PIR_KEYWORDS	transmembrane	1.13E-04
UP_SEQ_FEATURE	glycosylation site:N-linked (GlcNAc...)	1.35E-04
UP_SEQ_FEATURE	topological domain:Extracellular	3.06E-04
SP_PIR_KEYWORDS	glycoprotein	3.93E-04
UP_SEQ_FEATURE	topological domain:Cytoplasmic	0.001852536
GOTERM_CC_FAT	GO:0016021~integral to membrane	0.002694147
GOTERM_CC_FAT	GO:0031224~intrinsic to membrane	0.004878841
**Annotation Cluster 2**	**Enrichment Score: 2.90**	
Category	Term	P-value
SP_PIR_KEYWORDS	signal	4.48E-05
UP_SEQ_FEATURE	signal peptide	4.57E-05
UP_SEQ_FEATURE	glycosylation site:N-linked (GlcNAc...)	1.35E-04
SP_PIR_KEYWORDS	glycoprotein	3.93E-04
SP_PIR_KEYWORDS	disulfide bond	0.002047932
SP_PIR_KEYWORDS	Secreted	0.007258047
UP_SEQ_FEATURE	disulfide bond	0.034776624
GOTERM_CC_FAT	GO:0005576~extracellular region	0.109053733

Drug annotation:

No enriched terms found.

CODIM 19

162 genes, 27 chemicals

Functional enrichment of genes:

Annotation Cluster 1	Enrichment Score: 2.41	
Category	Term	P-value
GOTERM_CC_FAT	GO:0005783~endoplasmic reticulum	0.001041882
SP_PIR_KEYWORDS	endoplasmic reticulum	0.001620378
GOTERM_CC_FAT	GO:0044432~endoplasmic reticulum part	0.033999285

Drug annotation:

No enriched terms found.

CODIM 20

165 genes, 18 chemicals
Functional enrichment of genes:

Annotation Cluster 1	Enrichment Score: 2.09	
Category	Term	P-value
GOTERM_CC_FAT	GO:0043228~non-membrane-bounded organelle	0.004337048
GOTERM_CC_FAT	GO:0043232~intracellular non-membrane-bounded organelle	0.004337048
GOTERM_CC_FAT	GO:0005856~cytoskeleton	0.026841081
**Annotation Cluster 2**	**Enrichment Score: 2.06**	
Category	Term	P-value
GOTERM_BP_FAT	GO:0016192~vesicle-mediated transport	4.95E-04
GOTERM_BP_FAT	GO:0046907~intracellular transport	0.001693696
SP_PIR_KEYWORDS	protein transport	0.002182699
GOTERM_BP_FAT	GO:0008104~protein localization	0.003850693
GOTERM_BP_FAT	GO:0015031~protein transport	0.009329004
GOTERM_BP_FAT	GO:0045184~establishment of protein localization	0.01024802
SP_PIR_KEYWORDS	transport	0.029596983
GOTERM_BP_FAT	GO:0034613~cellular protein localization	0.042971308
GOTERM_BP_FAT	GO:0070727~cellular macromolecule localization	0.045534093
GOTERM_BP_FAT	GO:0006886~intracellular protein transport	0.053601216

Drug annotation:
No enriched terms found.

CODIM 21
619 genes, 24 chemicals
Functional enrichment of genes:

Annotation Cluster 1	Enrichment Score: 6.41	
Category	Term	PValue
UP_SEQ_FEATURE	glycosylation site:N-linked (GlcNAc...)	3.55E-10
SP_PIR_KEYWORDS	glycoprotein	4.87E-10
UP_SEQ_FEATURE	disulfide bond	1.70E-09
UP_SEQ_FEATURE	topological domain:Extracellular	2.48E-09
SP_PIR_KEYWORDS	disulfide bond	3.29E-09
SP_PIR_KEYWORDS	signal	2.43E-07
UP_SEQ_FEATURE	signal peptide	2.55E-07
UP_SEQ_FEATURE	topological domain:Cytoplasmic	6.66E-07
GOTERM_CC_FAT	GO:0005887~integral to plasma membrane	1.87E-06
GOTERM_CC_FAT	GO:0031226~intrinsic to plasma membrane	3.14E-06
SP_PIR_KEYWORDS	transmembrane	3.29E-06
UP_SEQ_FEATURE	transmembrane region	3.89E-06
GOTERM_CC_FAT	GO:0031224~intrinsic to membrane	5.28E-06
GOTERM_CC_FAT	GO:0016021~integral to membrane	1.97E-05
GOTERM_CC_FAT	GO:0005886~plasma membrane	4.06E-05
GOTERM_CC_FAT	GO:0044459~plasma membrane part	5.08E-05
SP_PIR_KEYWORDS	membrane	5.18E-05
**Annotation Cluster 2**	**Enrichment Score: 5.83**	
Category	Term	PValue
GOTERM_CC_FAT	GO:0005576~extracellular region	3.19E-08
SP_PIR_KEYWORDS	Secreted	4.11E-08
SP_PIR_KEYWORDS	signal	2.43E-07
UP_SEQ_FEATURE	signal peptide	2.55E-07
GOTERM_CC_FAT	GO:0044421~extracellular region part	1.97E-04
GOTERM_CC_FAT	GO:0005615~extracellular space	6.25E-04
**Annotation Cluster 3**	**Enrichment Score: 2.48**	
Category	Term	PValue
GOTERM_BP_FAT	GO:0009611~response to wounding	6.91E-04
GOTERM_BP_FAT	GO:0006952~defense response	8.49E-04
GOTERM_BP_FAT	GO:0006954~inflammatory response	0.05971829

Drug annotation:
No enriched terms found.

CODIM 22
836 genes, 14 chemicals
Functional enrichment of genes:

**Annotation Cluster 1**	**Enrichment Score: 5.03**	
Category	Term	P-value
UP_SEQ_FEATURE	topological domain:Extracellular	1.25E-08
UP_SEQ_FEATURE	topological domain:Cytoplasmic	5.29E-07
SP_PIR_KEYWORDS	disulfide bond	2.15E-06
UP_SEQ_FEATURE	glycosylation site:N-linked (GlcNAc...)	3.66E-06
UP_SEQ_FEATURE	disulfide bond	6.43E-06
SP_PIR_KEYWORDS	transmembrane	9.26E-06
UP_SEQ_FEATURE	transmembrane region	9.46E-06
SP_PIR_KEYWORDS	glycoprotein	2.47E-05
SP_PIR_KEYWORDS	signal	3.10E-05
UP_SEQ_FEATURE	signal peptide	3.36E-05
SP_PIR_KEYWORDS	membrane	4.05E-05
GOTERM_CC_FAT	GO:0016021~integral to membrane	3.19E-04
GOTERM_CC_FAT	GO:0031224~intrinsic to membrane	3.92E-04
**Annotation Cluster 2**	**Enrichment Score: 4.15**	
Category	Term	P-value
UP_SEQ_FEATURE	topological domain:Extracellular	1.25E-08
UP_SEQ_FEATURE	topological domain:Cytoplasmic	5.29E-07
SP_PIR_KEYWORDS	receptor	8.87E-06
GOTERM_CC_FAT	GO:0005886~plasma membrane	6.35E-04
GOTERM_CC_FAT	GO:0031226~intrinsic to plasma membrane	7.13E-04
GOTERM_CC_FAT	GO:0005887~integral to plasma membrane	7.38E-04
SP_PIR_KEYWORDS	cell membrane	0.003911328
GOTERM_CC_FAT	GO:0044459~plasma membrane part	0.007741016
**Annotation Cluster 3**	**Enrichment Score: 3.14**	
Category	Term	P-value
GOTERM_MF_FAT	GO:0005543~phospholipid binding	4.71E-04
GOTERM_MF_FAT	GO:0035091~phosphoinositide binding	5.71E-04
UP_SEQ_FEATURE	domain:PX	7.39E-04
SMART	SM00312:PX	8.21E-04
GOTERM_MF_FAT	GO:0008289~lipid binding	8.65E-04
INTERPRO	IPR001683:Phox-like	9.70E-04

Drug annotation:
No enriched terms found.

CODIM 23
102 genes, 58 chemicals
Functional enrichment of genes:

**Annotation Cluster 1**	**Enrichment Score: 3.98**	
Category	Term	P-value
INTERPRO	IPR001909:Krueppel-associated box	1.78E-08
UP_SEQ_FEATURE	domain:KRAB	2.02E-08
UP_SEQ_FEATURE	zinc finger region:C2H2-type 6	6.22E-08
SMART	SM00349:KRAB	6.96E-08
UP_SEQ_FEATURE	zinc finger region:C2H2-type 7	2.26E-07
UP_SEQ_FEATURE	zinc finger region:C2H2-type 5	3.24E-07
UP_SEQ_FEATURE	zinc finger region:C2H2-type 8	5.20E-07
INTERPRO	IPR013087:Zinc finger, C2H2-type/integrase, DNA-binding	5.45E-07
INTERPRO	IPR015880:Zinc finger, C2H2-like	7.43E-07
UP_SEQ_FEATURE	zinc finger region:C2H2-type 3	1.21E-06
UP_SEQ_FEATURE	zinc finger region:C2H2-type 11	1.25E-06

INTERPRO	IPR007087:Zinc finger, C2H2-type	3.30E-06
SMART	SM00355:ZnF_C2H2	3.57E-06
UP_SEQ_FEATURE	zinc finger region:C2H2-type 4	3.60E-06
UP_SEQ_FEATURE	zinc finger region:C2H2-type 10	3.64E-06
UP_SEQ_FEATURE	zinc finger region:C2H2-type 12	4.06E-06
UP_SEQ_FEATURE	zinc finger region:C2H2-type 2	5.18E-06
UP_SEQ_FEATURE	zinc finger region:C2H2-type 13	1.20E-05
UP_SEQ_FEATURE	zinc finger region:C2H2-type 17	2.24E-05
SP_PIR_KEYWORDS	zinc-finger	4.03E-05
UP_SEQ_FEATURE	zinc finger region:C2H2-type 16	7.44E-05
UP_SEQ_FEATURE	zinc finger region:C2H2-type 9	8.57E-05
UP_SEQ_FEATURE	zinc finger region:C2H2-type 15	1.73E-04
PIR_SUPERFAMILY	PIRSF005559:zinc finger protein ZFP-36	3.33E-04
UP_SEQ_FEATURE	zinc finger region:C2H2-type 1	4.16E-04
GOTERM_MF_FAT	GO:0008270~zinc ion binding	6.82E-04
GOTERM_MF_FAT	GO:0003677~DNA binding	7.01E-04
SP_PIR_KEYWORDS	zinc	7.02E-04
GOTERM_BP_FAT	GO:0006350~transcription	0.001896131
UP_SEQ_FEATURE	zinc finger region:C2H2-type 18	0.002231138
SP_PIR_KEYWORDS	dna-binding	0.002515738
SP_PIR_KEYWORDS	metal-binding	0.003244845
GOTERM_BP_FAT	GO:0006355~regulation of transcription, DNA-dependent	0.0033329
GOTERM_BP_FAT	GO:0051252~regulation of RNA metabolic process	0.00458636
GOTERM_MF_FAT	GO:0046914~transition metal ion binding	0.00532855
GOTERM_BP_FAT	GO:0045449~regulation of transcription	0.009425629
GOTERM_MF_FAT	GO:0046872~metal ion binding	0.011965016
GOTERM_MF_FAT	GO:0043169~cation binding	0.013365187
GOTERM_MF_FAT	GO:0043167~ion binding	0.016002071
SP_PIR_KEYWORDS	Transcription	0.018692939
UP_SEQ_FEATURE	zinc finger region:C2H2-type 14	0.022267624
SP_PIR_KEYWORDS	zinc finger	0.027265456
UP_SEQ_FEATURE	zinc finger region:C2H2-type 1; degenerate	0.042265807
SP_PIR_KEYWORDS	transcription regulation	0.048200293
SP_PIR_KEYWORDS	nucleus	0.078299633
SP_PIR_KEYWORDS	DNA binding	0.093466428

Drug annotation:

No enriched terms found.

MCF7 Dataset:

Drug-induced transcriptional module: MCF7-9

49 genes, 37 chemicals

Functional enrichment of genes:

**Annotation Cluster 1**	**Enrichment Score: 1.61**	
Category	Term	P-value
GOTERM_BP_FAT	GO:0007186~G-protein coupled receptor protein signaling pathway	3.33E-03
GOTERM_BP_FAT	GO:0032103~positive regulation of response to external stimulus	2.30E-02
GOTERM_BP_FAT	GO:0048584~positive regulation of response to stimulus	5.37E-02
GOTERM_BP_FAT	GO:0032101~regulation of response to external stimulus	8.80E-02
**Annotation Cluster 2**	**Enrichment Score: 1.56**	
Category	Term	P-value
GOTERM_BP_FAT	GO:0060429~epithelium development	5.43E-03
GOTERM_BP_FAT	GO:0035295~tube development	7.90E-03
GOTERM_BP_FAT	GO:0048729~tissue morphogenesis	2.44E-02
GOTERM_BP_FAT	GO:0016331~morphogenesis of embryonic epithelium	2.53E-02
GOTERM_BP_FAT	GO:0001822~kidney development	4.26E-02
GOTERM_BP_FAT	GO:0002009~morphogenesis of an epithelium	5.64E-02
GOTERM_BP_FAT	GO:0001655~urogenital system development	5.80E-02

GOTERM_BP_FAT	GO:0048598~embryonic morphogenesis	7.99E-02

Drug annotation:

Annotation type	Enriched term	p-value (FDR)
Drug target	ESR1; estrogen receptor 1	2.98E-04
ATC code (2nd level)	G03; Sex hormones and modulators of the genital system	3.33E-08
ATC code (2nd level)	L02; Endocrine therapy	7.76E-04
Side effect	vulvovaginitis	4.87E-03
Side effect	pelvic pain	7.53E-03
Side effect	endometriosis	9.59E-03
Side effect	pneumonia	9.76E-03
Side effect	varicose vein	1.53E-02
Side effect	Endocrine Disorders	2.10E-02
Side effect	uterine fibroids	2.53E-02
Side effect	breast neoplasm	5.98E-02
Side effect	breast pain	7.08E-02
Chemical Fragment	c1ccc(cc1)CC	5.49E-07
Chemical Fragment	C=C1CCCC2(C)(C(CC)CCC12)	7.95E-06
Chemical Fragment	Cc1c(cccc1Cl)Cl	7.95E-06
Chemical Fragment	C(C)CCCC(C)C	5.47E-05
Chemical Fragment	c1c(cc(cc1I)I)I	7.20E-05
Chemical Fragment	C(C)CCCC(C)C	1.21E-04
Chemical Fragment	C(=NCCCCCC)N	4.91E-04
Chemical Fragment	C(=NCCCCC)N	4.91E-04
Chemical Fragment	C(c1ccccc1)CC	5.16E-04
Chemical Fragment	C(=C(NCc1cnc(nc1(N))C)C)S	9.45E-04
Chemical Fragment	c1c(cc(cc1I)I)I	3.78E-03
Chemical Fragment	C(=NCCCCCCN=CN)N	3.78E-03

Drugs	Drug annotation	Literature evidence
pregnenolone	GABAA antagonist, increases neurogenesis in the hippocampus	(Mayo *et al*, 2005)
fulvestrant	estrogen receptor antagonist (ATC: L02BA03)	
butyl hydroxybenzoate	oestrogenic activity	(Charles & Darbre, 2009)
epiandrosterone	steroid hormone with weak androgenic activity	
bendroflumethiazide	thiazide diuretic used to treat hypertension (ATC: C03AA01)	
estropipate	a form of estrogen	
pentoxyverine	selective agonist at the sigma-1 receptor (ATC: R05DB05)	
diethylstilbestrol	inhibits proliferation of human prostate cancer cell lines; nonsteroidal estrogen (ATC: G03CB02)	(Koike *et al*, 2005)
dilazep	adenosine reuptake inhibitor (ATC: C01DX10)	
dimethadione	anticonvulsant	
epitiostanol	steroidal antiestrogen	(Konishi *et al*, 1988)
equilin	increases ESR1 activity	(Davis *et al*, 2011)
estriol	one of the three main estrogens	
ethisterone	progestogen hormone (ATC: G03DC04)	
etynodiol	hormonal contraceptive (ATC: G03DC06)	
hexestrol		
ketorolac	cyclooxygenase inhibitor (ATC: M01AB15)	
lorglumide	cholecystokinin antagonist	(Makovec *et al*, 1987)
mestranol	estrogen	
nitrendipine	calcium channel blocker (ATC: C08CA08)	
estradiol	estrogen receptor agonist (ATC: G03CA03)	PubChem ID:450
alpha-estradiol	estrogen receptor agonist (ATC: G03CA03)	PubChem ID:450
noretynodrel	synthetic progestogen (ATC: G03FA09)	
levonorgestrel	synthetic progestogen (ATC: G03AC03)	
raloxifene	selective estrogen receptor modulator (ATC: G03XC01)	
Ribavirin	anti-viral drug (ATC: J05AB04)	

solasodine	glycoalkaloid	
Genistein	estrogen receptor agonist	
tamoxifen	estrogen receptor antagonist (ATC: L02BA01)	
testosterone	steroid hormone from the androgen group	
theobromine	vasodilator and diuretic (ATC: C03BD01)	
corticosterone	corticosteroid (steroid hormone)	
lynestrenol	progestagen hormone (ATC: G03AC02)	
estrone	estrogenic hormone	
prasterone	can be converted to androgen testosterone and estrogens	(Mo *et al*, 2006)
naringenin	flavanon; antioxidant, anti-inflammatory	
aminophylline	nonselective phosphodiesterase inhibitor (ATC: R03DA05)	

PC3 Dataset:

Drug-induced transcriptional module: PC3-3

38 genes, 25 chemicals

Functional enrichment of genes:

**Annotation Cluster 1**	**Enrichment Score: 3.96**	
Category	Term	P-value
SP_PIR_KEYWORDS	signal	3.44E-06
UP_SEQ_FEATURE	signal peptide	3.49E-06
UP_SEQ_FEATURE	disulfide bond	3.79E-05
SP_PIR_KEYWORDS	disulfide bond	6.34E-05
SP_PIR_KEYWORDS	Secreted	4.68E-03
GOTERM_CC_FAT	GO:0005576~extracellular region	1.22E-02
**Annotation Cluster 2**	**Enrichment Score: 2.10**	
Category	Term	P-value
SP_PIR_KEYWORDS	unfolded protein response	1.55E-03
GOTERM_BP_FAT	GO:0051789~response to protein stimulus	4.08E-03
GOTERM_BP_FAT	GO:0006986~response to unfolded protein	1.70E-02
GOTERM_BP_FAT	GO:0010033~response to organic substance	3.67E-02
**Annotation Cluster 3**	**Enrichment Score: 1.68**	
Category	Term	P-value
UP_SEQ_FEATURE	short sequence motif:Prevents secretion from ER	8.92E-04
GOTERM_CC_FAT	GO:0005788~endoplasmic reticulum lumen	2.38E-03
SP_PIR_KEYWORDS	endoplasmic reticulum	2.61E-03
GOTERM_CC_FAT	GO:0044432~endoplasmic reticulum part	4.88E-03
GOTERM_CC_FAT	GO:0005783~endoplasmic reticulum	7.13E-03
INTERPRO	IPR000886:Endoplasmic reticulum, targeting sequence	8.92E-03
GOTERM_CC_FAT	GO:0043233~organelle lumen	6.97E-01
GOTERM_CC_FAT	GO:0031974~membrane-enclosed lumen	7.17E-01
GOTERM_CC_FAT	GO:0070013~intracellular organelle lumen	8.25E-01

Drug annotation:

**Annotation type**	**Enrichment term**	**p-value (FDR)**
Drug target	HRH1;histamine receptor H1	1.00E-05
Drug target	HTR2A;5-hydroxytryptamine (serotonin) receptor 2A	2.96E-05
Drug target	DRD2;dopamine receptor D2	3.78E-05
Drug target	DRD1;dopamine receptor D1	1.03E-04
Drug target	ADRA1A;adrenergic, alpha-1A-, receptor	2.60E-04
Drug target	DRD3;dopamine receptor D3	2.60E-04
Drug target	CHRM2;cholinergic receptor, muscarinic 2	4.15E-04
Drug target	CHRM4;cholinergic receptor, muscarinic 4	1.33E-03

Drug target	HTR2C;5-hydroxytryptamine (serotonin) receptor 2C	1.40E-03
Drug target	SLC6A2;solute carrier family 6 (neurotransmitter transporter, noradrenalin), member 2	1.45E-03
Drug target	CHRM5;cholinergic receptor, muscarinic 5	1.55E-03
Drug target	CHRM1;cholinergic receptor, muscarinic 1	1.77E-03
Drug target	ADCY1;adenylate cyclase 1 (brain)	8.41E-03
Drug target	CHRM3;cholinergic receptor, muscarinic 3	9.84E-03
Drug target	SLC6A4;solute carrier family 6 (neurotransmitter transporter, serotonin), member 4	1.08E-02
Drug target	HTR6;5-hydroxytryptamine (serotonin) receptor 6	1.28E-02
Drug target	HTR1A;5-hydroxytryptamine (serotonin) receptor 1A	1.81E-02
Drug target	HRH4;histamine receptor H4	3.71E-02
Drug target	DRD4;dopamine receptor D4	5.10E-02
ATC code (2nd level)	R06; Antihistamines for systemic use	1.89E-02
Side effect	paralytic ileus	3.85E-04
Side effect	galactorrhea	8.97E-03
Side effect	gynecomastia	2.77E-02
Side effect	breast enlargement	7.46E-02
Side effect	urinary retention	9.40E-02

**Drugs**	**Drug annotation**	**Literature evidence**
deptropine	antihistamine with anticholinergic properties (ATC: R06AX16)	
promazine	N05 psycholeptic	
homochlorcyclizine	antihistamine	(Haraguchi *et al*, 1997)
cyproheptadine	antihistamine (ATC: R06AX02)	
thioridazine	cytogenetic damage; inhibits cell proliferation; antipsychotic drug (ATC: N05AC02)	(Strobl *et al*, 1990)
pizotifen	prevents vascular headache (ATC: N02CX01)	
clomipramine	tricyclic antidepressant (ATC: N06AA04)	
chlorpromazine	N05 psycholeptic	
trimipramine	N06 psychoanaleptic	
quinisocaine	antipruritic (ATC: D04AB05)	
alimemazine	antihistamine (ATC: R06AD01)	
cyclobenzaprine	muscle relaxant (ATC: M03BX08)	
amitriptyline	tricyclic antidepressant (ATC: N06AA09)	
imipramine	tricyclic antidepressant (ATC: N06AA02)	
promethazine	antihistamine (ATC: R06AD02)	
profenamine	antiparkinsonian agent; antihistamine action (ATC: N04AA05)	
chloropyramine	antihistamine (ATC: R06AC03)	
triprolidine	antihistamine with anticholinergic properties (ATC: R06AX07)	
vinburnine	vasodilator (ATC: C04AX17)	
loxapine	used to treat schizophrenia (ATC: N05AH01)	
mianserin	psychoactive drug (ATC: N06AX03)	
nisoxetine	selective norepinephrine reuptake inhibitor	(Graham & Langer, 1992)
metixene	N04 anti-parkinson drug	
domperidone	antidopaminergic drug (ATC: A03FA03)	
dicycloverine	muscarinic receptor antagonist (ATC: A03AA07)	

Drug-induced transcriptional module: PC3-9

39 genes, 17 chemicals

Functional enrichment of genes:

No significant annotation clusters found.

Drug Annotation:

**Annotation type**	**Enrichment term**	**p-value (FDR)**
Drug target	SERPINE1;serpin peptidase inhibitor, clade E (nexin, plasminogen activator inhibitor type 1), member 1	1.08E-02
Drug target	LPL;lipoprotein lipase	1.08E-02
Drug target	PPARG;peroxisome proliferator-activated receptor gamma	1.08E-02

Drug target	SLCO1B1;solute carrier organic anion transporter family, member 1B1	1.81E-02
Drug target	ABCC8;ATP-binding cassette, sub-family C (CFTR/MRP), member 8	2.86E-02
ATC code (2nd level)	A10; Drugs used in diabetes	1.70E-04

**Drugs**	**Drug annotation**	**Literature evidence**
troglitazone	antidiabetic (ATC: A10BG01); peroxisome proliferator-activated receptor gamma activator	
rosiglitazone	antidiabetic drug (ATC: A10BG02); peroxisome proliferator-activated receptor gamma activator	
15−delta prostaglandin J2	NSAID; inhibits cell proliferation	(Kim *et al*, 2007; Forman *et al*, 1995)
gliquidone	sulfonylurea antidiabetic (ATC: A10BB08); peroxisome proliferator-activated receptor gamma activator	(Scarsi *et al*, 2007)
glibenclamide	sulfonylurea antidiabetic (ATC: A10BB01); peroxisome proliferator-activated receptor gamma activator	(Fukuen *et al*, 2005)
mifepristone	progesterone receptor antagonist	
glimepiride	sulfonylurea antidiabetic (ATC: A10BB12); peroxisome proliferator-activated receptor gamma activator	(Scarsi *et al*, 2007; Fukuen *et al*, 2005)
tiratricol	thyroid hormone analogue (ATC: H03AA04, D11AX08)	
tretinoin	acid form of vitamin A; used to treat acne vulgaris and keratosis pilaris; antineoplastic (ATC: L01XX14), all-trans retinoic acid, peroxisome proliferator-activated receptor gamma activator	
3−acetylcoumarin		
acemetacin	non-steroidal anti-inflammatory drug (ATC: M01AB11)	
clemizole	antihistamine	
rilmenidine	used to treat hypertension	
bezafibrate	fibrate drug against hyperlipidaemia; peroxisome proliferator-activated receptor gamma activator	
zaprinast	phosphodiesterase inhibitor	(Choi *et al*, 2002)
racecadotril	antidiarrheal (ATC: A07XA04); enkephalinase inhibitor	(Matheson & Noble, 2000)
raubasine	antihypertensive and antidiabetic	

Drug-induced transcriptional module: PC3-11
73 genes, 22 chemicals
Functional enrichment of genes:

**Annotation Cluster 1**	**Enrichment Score: 5.14**	
Category	Term	P-value
UP_SEQ_FEATURE	glycosylation site:N-linked (GlcNAc...)	3.03E-09
SP_PIR_KEYWORDS	glycoprotein	1.56E-08
SP_PIR_KEYWORDS	Secreted	8.60E-08
SP_PIR_KEYWORDS	signal	1.01E-06
UP_SEQ_FEATURE	signal peptide	1.04E-06
GOTERM_CC_FAT	GO:0005576~extracellular region	3.30E-06
GOTERM_CC_FAT	GO:0044421~extracellular region part	4.39E-04
SP_PIR_KEYWORDS	disulfide bond	1.16E-03
UP_SEQ_FEATURE	disulfide bond	4.98E-03
GOTERM_CC_FAT	GO:0005615~extracellular space	1.09E-02
**Annotation Cluster 2**	**Enrichment Score: 2.39**	
Category	Term	P-value
GOTERM_MF_FAT	GO:0030414~peptidase inhibitor activity	9.73E-05
GOTERM_MF_FAT	GO:0004866~endopeptidase inhibitor activity	9.73E-05
GOTERM_BP_FAT	GO:0042060~wound healing	1.44E-03
KEGG_PATHWAY	hsa04610:Complement and coagulation cascades	2.02E-03
GOTERM_BP_FAT	GO:0050878~regulation of body fluid levels	2.81E-03
GOTERM_MF_FAT	GO:0004867~serine-type endopeptidase inhibitor activity	2.82E-03
GOTERM_MF_FAT	GO:0004857~enzyme inhibitor activity	4.55E-03
SP_PIR_KEYWORDS	serine proteinase inhibitor	5.76E-03
INTERPRO	IPR000215:Protease inhibitor I4, serpin	6.31E-03

SMART	SM00093:SERPIN	7.02E-03
GOTERM_BP_FAT	GO:0050817~coagulation	8.81E-03
GOTERM_BP_FAT	GO:0007596~blood coagulation	8.81E-03
PIR_SUPERFAMILY	PIRSF001630:serpin	9.34E-03
GOTERM_BP_FAT	GO:0007599~hemostasis	1.08E-02
UP_SEQ_FEATURE	site:Reactive bond	1.17E-02
SP_PIR_KEYWORDS	Serine protease inhibitor	2.22E-02
SP_PIR_KEYWORDS	protease inhibitor	3.55E-02

Drug annotation:

Annotation type	Enriched term	p-value (FDR)
Drug target	ADRB2; adrenergic, beta-2-, receptor, surface	6.55E-08
Drug target	ADRB1; adrenergic, beta-1-, receptor	2.60E-04
Drug target	ADRB3; adrenergic, beta-3-, receptor	3.75E-03
Drug target	ADCY10; adenylate cyclase 10	1.81E-02
ATC code (2nd level)	R03; Drugs for obstructive airway diseases	3.19E-06
ATC code (2nd level)	C01; Cardiac therapy	7.91E-02
Side effect	tachycardia	7.88E-02
Chemical Fragment	C(=NCCCCC)N	2.35E-12
Chemical Fragment	C(C)c1ccccc1	1.02E-08
Chemical Fragment	C(=C(NCc1cnc(nc1(N))C)C)S	6.44E-08
Chemical Fragment	C(c1ccccc1)c2ccccc2	1.14E-07
Chemical Fragment	C(=NCCCCCCN=CN)N	1.14E-07
Chemical Fragment	C(C)(C)CC(C)(C)C	1.46E-07
Chemical Fragment	C(=NCCCCCCN=CN)N	5.86E-07
Chemical Fragment	C(C)=C(NCc1cnc(nc1(N))C)C	7.36E-07
Chemical Fragment	C(=NCCCCCCN=CN)N	7.36E-07
Chemical Fragment	C(=NCCCCC)N	1.92E-06
Chemical Fragment	C(=NCCCCC)N	3.12E-06
Chemical Fragment	C(=NCCCCCCN=CN)N	7.95E-06
Chemical Fragment	C(C)(C)CC(C)(C)C	1.34E-05
Chemical Fragment	C(=NCCCC)N	1.41E-05
Chemical Fragment	C(=NCCCC)N	2.00E-05
Chemical Fragment	C(=NCCCC)N	2.19E-05
Chemical Fragment	C(=NOC)c1nc(N)sc1	9.31E-05
Chemical Fragment	C(=NCCCC)N	1.32E-04
Chemical Fragment	C(=C(NCc1cnc(nc1(N))C)C)S	1.89E-04
Chemical Fragment	c1ccc(C)cc1	1.99E-04
Chemical Fragment	C=CNCCSC	1.99E-04
Chemical Fragment	C1=Nc3ccccc3(Oc2ccc(cc12)Cl)	3.87E-04
Chemical Fragment	C(=C(NCc1cnc(nc1(N))C)C)S	3.87E-04
Chemical Fragment	C(=NCCCCCCN=CN)N	7.64E-04
Chemical Fragment	C(=NOC(C)C)c1nc(N)sc1	8.41E-04
Chemical Fragment	C(C)CCCC(C)C	1.09E-03
Chemical Fragment	C(=NOC(C)C)c1nc(N)sc1	2.13E-03
Chemical Fragment	C(=NOC)c1nc(N)sc1	2.93E-03
Chemical Fragment	C(=NCCCCC)N	6.81E-03

**Drugs**	**Drug annotation**	**Literature evidence**
salbutamol	ADRB2 agonist (ATC: R03AC02) (ADRB2: adrenergic, beta-2-receptor)	
metanephrine	metabolite of epinephrine (that is ADRB2 agonist)	(Thomas & Friedland, 1998)
alprostadil	Prostaglandin; used to treat erectile dysfunction (ATC: G04BE01); increases phosphorylation of ADRB2 protein	(Davis *et al*, 2011)
adenosine phosphate	cyclic structure known as cyclic AMP	
butamben	local anesthetic	
clenbuterol	ADRB2 agonist (ATC: R03AC14)	
dipivefrine	ADRB2 agonist	
dipyridamole	inhibits thrombus formation (ATC: B01AC07)	
ritodrine	ADRB2 agonist	
ethaverine	vasodilator and antispasmodic	

etilefrine	ADRB2 agonist	(Nusser *et al*, 1965)
fenoterol	ADRB2 agonist	
dobutamine	ADRB2 agonist	
isoetarine	ADRB2 agonist	
(+)- isoprenaline	ADRB2 agonist	PubChem ID: 3779
(-)- isoprenaline	ADRB2 agonist	PubChem ID: 3779
isoxsuprine	ADRB2 agonist	(Falkay & Kovács, 1986)
corbadrine	metabolite of methyldopa (alpha-2-adrenergic receptor agonist)	
orciprenaline	ADRB2 agonist	
papaverine	antispasmodic (ATC: A03AD01); inhibits phosphodiesterase causing elevation of cyclic AMP	
terbutaline	ADRB2 agonist (ATC: R03AC03)	
zardaverine	phosphodiesterases (PDE III/IV) inhibitor	

HL60 Dataset:

Drug-induced transcriptional module: HL60-17

21 genes, 27 chemicals

Functional enrichment of genes:

**Annotation Cluster 1**	**Enrichment Score: 1.88**	
Category	Term	P-value
GOTERM_BP_FAT	GO:0051249~regulation of lymphocyte activation	1.15E-02
GOTERM_BP_FAT	GO:0002694~regulation of leukocyte activation	1.32E-02
GOTERM_BP_FAT	GO:0050865~regulation of cell activation	1.46E-02

Drug annotation:

**Annotation type**	**Enriched term**	**p-value (FDR)**
Drug target	ADRB2; adrenergic, beta-2-, receptor, surface	4.54E-03
ATC code (2nd level)	R03; Drugs for obstructive airway diseases	1.46E-03
ATC code (2nd level)	G02; Other gynecologicals	4.72E-02
Chemical Fragment	C(=C(NCc1cnc(nc1(N))C)C)S	7.92E-07
Chemical Fragment	C(=NCCCC)N	1.51E-05
Chemical Fragment	C(=NCCCC)N	1.71E-05
Chemical Fragment	C(=NCCCCC)N	1.72E-05
Chemical Fragment	C(=NCCCCC)N	2.10E-05
Chemical Fragment	C(=C(NCc1cnc(nc1(N))C)C)S	1.41E-04
Chemical Fragment	C(=NCCCCCCN=CN)N	2.39E-04
Chemical Fragment	C(=NCCCC)N	4.50E-04
Chemical Fragment	C(=NCCCCC)N	5.25E-04
Chemical Fragment	C(=NCCCC)N	7.79E-04
Chemical Fragment	C(=NCCCCCCN=CN)N	1.14E-03
Chemical Fragment	C(=NCCCC)N	1.24E-03
Chemical Fragment	C(C)c1ccccc1	1.24E-03
Chemical Fragment	C(=C(NCc1cnc(nc1(N))C)C)S	1.83E-03
Chemical Fragment	C(=NCCCCC)N	1.83E-03
Chemical Fragment	C1c3ccccc3(C=Cc2ccccc12)	1.83E-03
Chemical Fragment	C(c1ccccc1)c2ccccc2	1.83E-03
Chemical Fragment	C(=C(NCc1cnc(nc1(N))C)C)S	1.83E-03
Chemical Fragment	C(=NCCCCCCN=CN)N	1.83E-03
Chemical Fragment	C1=Nc3ccccc3(Oc2ccc(cc12)Cl)	2.61E-03
Chemical Fragment	C(=C(NCc1cnc(nc1(N))C)C)S	2.61E-03
Chemical Fragment	C(C)c1ccccc1	4.05E-03
Chemical Fragment	C(C)=C(NCc1cnc(nc1(N))C)C	6.83E-03
Chemical Fragment	C(=NCCCCCCN=CN)N	6.83E-03
Chemical Fragment	C(=NOC)c1nc(N)sc1	7.33E-03
Chemical Fragment	C(C)(C)CC(C)(C)C	9.17E-03
Chemical Fragment	C(C)(C)CC(C)(C)C	9.55E-03

Drugs	Drug annotation	Literature evidence
(+)-chelidonine	cell cycle blocker	(Panzer *et al*, 2001)
co-dergocrine mesilate	alpha-adrenergic receptor antagonist	(Kitamura, 1980)
dinoprost	abortifacient (ATC: G02AD01)	
salbutamol	ADRB2 agonist (ATC: R03AC02) (ADRB2 : adrenergic, beta-2-receptor)	
alprostadil	prostaglandin; used to treat erectile dysfunction (ATC: G04BE01); increases phosphorylation of ADRB2 protein	
bromocriptine	alpha-2 adrenergic agonist	(De Leeuw van Weenen *et al*, 2010)
clonidine	centrally acting alpha-2 adrenergic agonist (ATC: N02CX02)	
dihydroergocristine	used to treat dementia and age-related cognitive impairment (ATC: C04AE01)	
dihydroergotamine	alpha 2-adrenergic agonist and alpha 1-adrenergic antagonist	(Nusser *et al*, 1965)
dipivefrine	ADRB2 agonist	
ethaverine	vasodilator and antispasmodic	
etilefrine	ADRB2 agonist	(Ruffolo *et al*, 1987)
fenoterol	ADRB2 agonist	
hexetidine	anti-bacterial and anti-fungal (ATC: A01AB12)	
isoetarine	ADRB2 agonist	
(+)-isoprenaline	ADRB2 agonist	PubChem ID: 3779
(-)-isoprenaline	ADRB2 agonist	PubChem ID: 3779
corbadrine	metabolite of methyldopa (alpha-2-adrenergic receptor agonist)	
orciprenaline	ADRB2 agonist(ATC: R03AB03)	
methylergometrine	vasoconstrictor and smooth muscle agonist (ATC: G02AB01)	
nimodipine	dihydropyridine calcium channel blocker (ATC: C08CA06)	
papaverine	antispasmodic (ATC: A03AD01); inhibits phosphodiesterase causing elevation of cyclic AMP	
pergolide	alpha-2 adrenergic agonist	(Reid *et al*, 1997)
podophyllotoxin	purgative, vesicant, antirheumatic, antiviral, and antitumor agents (ATC: D06BB04)	
terbutaline	ADRB2 agonist (ATC: R03AC03)	
vigabatrin	inhibits GABA transaminase (ATC: N03AG04)	
zardaverine	phosphodiesterases (PDE III/IV) inhibitor	

Rat Liver Dataset:

Drug-induced transcriptional module: RatLiver-2

54 genes, 49 experiments

Functional enrichment of genes:

**Annotation Cluster 1**	**Enrichment Score: 2.19**	
Category	Term	P-value
KEGG_PATHWAY	rno04210:Apoptosis	3.54E-04
GOTERM_BP_FAT	GO:0009617~response to bacterium	4.67E-03
GOTERM_BP_FAT	GO:0032496~response to lipopolysaccharide	6.26E-03
GOTERM_BP_FAT	GO:0002237~response to molecule of bacterial origin	8.02E-03
GOTERM_BP_FAT	GO:0019221~cytokine-mediated signaling pathway	1.80E-02
KEGG_PATHWAY	rno04060:Cytokine-cytokine receptor interaction	4.55E-02
**Annotation Cluster 2**	**Enrichment Score: 1.99**	
Category	Term	P-value
GOTERM_BP_FAT	GO:0009611~response to wounding	5.06E-05
GOTERM_BP_FAT	GO:0006954~inflammatory response	6.20E-05
GOTERM_BP_FAT	GO:0006952~defense response	2.49E-04
GOTERM_BP_FAT	GO:0050900~leukocyte migration	8.05E-04

GOTERM_BP_FAT	GO:0002526~acute inflammatory response	3.00E-03
GOTERM_BP_FAT	GO:0030595~leukocyte chemotaxis	6.29E-03
GOTERM_BP_FAT	GO:0060326~cell chemotaxis	7.32E-03
GOTERM_BP_FAT	GO:0016477~cell migration	9.39E-03
SP_PIR_KEYWORDS	inflammatory response	1.04E-02
GOTERM_BP_FAT	GO:0042060~wound healing	1.83E-02
GOTERM_BP_FAT	GO:0051674~localization of cell	2.05E-02
GOTERM_BP_FAT	GO:0048870~cell motility	2.05E-02
GOTERM_BP_FAT	GO:0007626~locomotory behavior	2.91E-02
GOTERM_BP_FAT	GO:0042330~taxis	2.93E-02
GOTERM_BP_FAT	GO:0006935~chemotaxis	2.93E-02
GOTERM_CC_FAT	GO:0005615~extracellular space	4.12E-02
GOTERM_BP_FAT	GO:0006928~cell motion	4.40E-02
SP_PIR_KEYWORDS	Secreted	5.58E-02
GOTERM_CC_FAT	GO:0044421~extracellular region part	1.30E-01
GOTERM_BP_FAT	GO:0007610~behavior	1.56E-01
GOTERM_BP_FAT	GO:0006955~immune response	3.88E-01
**Annotation Cluster 3**	**Enrichment Score: 1.79**	
Category	Term	P-value
GOTERM_BP_FAT	GO:0042493~response to drug	3.80E-03
GOTERM_BP_FAT	GO:0042981~regulation of apoptosis	5.35E-03
GOTERM_BP_FAT	GO:0043067~regulation of programmed cell death	5.75E-03
GOTERM_BP_FAT	GO:0010941~regulation of cell death	5.89E-03
GOTERM_BP_FAT	GO:0006916~anti-apoptosis	1.13E-02
GOTERM_BP_FAT	GO:0043066~negative regulation of apoptosis	2.18E-02
GOTERM_BP_FAT	GO:0043069~negative regulation of programmed cell death	2.29E-02
GOTERM_BP_FAT	GO:0060548~negative regulation of cell death	2.31E-02
GOTERM_BP_FAT	GO:0045449~regulation of transcription	7.81E-01

Drug annotation:

**Drugs**	**Experiments**	**Drug annotation**	**Literature evidence**
benoxaprofen	3days; exp6406<br>5days; exp6408	NSAID (ATC: M01AE06)	
benzethonium chloride	1days; exp6427<br>3days; exp6431<br>5days; exp6435	surfactant, antiseptic, anti-infective properties	
benzothiazyl disulfide	5days; exp5588	vulcanizing accelerant	
bupropion	1days; exp6052	atypical antidepressant and smoking cessation aid (ATC: N06AX12)	
celecoxib	5days; exp6368	NSAID; COX-2 inhibitor (ATC: M01AH01)	
cholecalciferol	3days; exp5040	a form of vitamin D similar to steroids	
diclofenac	3days; exp3352<br>5days; exp3348	NSAID (ATC: M01AB05)	
etodolac	3days; exp5659<br>5days; exp5668	NSAID (ATC: M01AB08)	
ibuprofen	.25days; exp2691<br>3days; exp2703<br>3days; exp6535<br>5days; exp2697	NSAID (ATC: M01AE01)	
indomethacin	1days; exp6550<br>5days; exp3317<br>3days; exp3322<br>3days; exp3524<br>3days; exp6534<br>3days; exp6544<br>4days; exp7029<br>5days; exp3523	NSAID (ATC: M01AB01)	
interleukin-1 beta (rat)	3days; exp6494		
ketorolac	5days; exp6055	NSAID (ATC: M01AB15)	
Lipopolysaccharide, E. coli O55:B5	3days; exp4704<br>3days; exp4940<br>3days; exp5237<br>5days; exp4820<br>5days; exp5099	endotoxin, elicits strong immune responses	(Hertz *et al*, 1956)

meloxicam	1days; exp3370	NSAID (ATC: M01AC06)	
methotrexate	3days; exp3294	cell cycle blocker; antimetabolite (ATC: L01BA01)	(Moe *et al*, 2008; Zold *et al*, 2011)
naproxen	1days; exp3256	NSAID (ATC: M01AE02)	
nimesulide	3days; exp2804	NSAID (ATC: M01AX17)	
nystatin	1days; exp5550<br>5days; exp5543	antifungal (ATC: D01AA01)	
oxiconazole	3days; exp5270<br>5days; exp5253	antifungal (ATC: D01AC11)	
pramoxine	1days; exp5561	PDE-4 inhibitor (ATC: R03DX07)	
roflumilast	3days; exp7317<br>4days; exp7328	topical anesthetic; antipruritic (ATC: D04AB07)	
sildenafil	5days; exp3528	used to treat erectile dysfunction and pulmonary arterial hypertension (ATC: G04BE03)	
sulindac	1days; exp3055<br>3days; exp2735<br>5days; exp2747	NSAID (ATC: M01AB02)	
tnf alpha (rat)	.25days; exp7532		
trichloroacetic acid	5days; exp5568		

<u>Drug-induced transcriptional module: RatLiver-7</u>

52 genes, 18 experiments

Functional enrichment of genes:

No significant annotation clusters found.

Drug annotation:

Drugs	Experiments	Drug annotation	Literature evidence
alfacalcidol	3days; exp6300	analogue of vitamin D; affects parathyroid hormone levels and immune system, including regulatory T cells	(Reid *et al*, 1997)
allyl alcohol	1days; exp6029		
amiodarone	3days; exp6206	antiarrhythmic agent (ATC: C01BD01)	
betamethasone	1days; exp6208	glucocorticoid steroid with anti-inflammatory and immunosuppressive properties (ATC: D07AC01)	
cerivastatin	5days; exp5449	statin; HMG-CoA reductase inhibitor (ATC: C10AA06)	
chlorpromazine	1days; exp2860	typical antipsychotic (ATC: N05AA01)	
cholecalciferol	3days; exp5040	a form of vitamin D similar to steroids	
ethinylestradiol	.25days; exp2867	a derivative of estradiol (ATC: L02AA03)	
fludrocortisone acetate	1days; exp6265	synthetic corticosteroid (ATC: H02AA02)	
fluocinolone acetonide	1days; exp6589	corticosteroid (ATC: D07AC04)	
hydralazine	1days; exp4380	smooth muscle relaxant; vasodilator (ATC: C02DB02)	
hydrocortisone	1days; exp6434	glucocorticoid (ATC: D07AA02)	
ketoconazole	1days; exp3255	antifungal drug (ATC: D01AC08)	
Lipopolysaccharide, E. coli O55:B5	3days; exp4704<br>3days; exp4940<br>5days; exp4820	endotoxin, elicits strong immune responses	(Seifert *et al*, 1994; Liu *et al*, 1993)
prednisolone	1days; exp4938	corticosteroid (ATC: D07AA03)	
primidone	3days; exp5667	anticonvulsant (ATC: N03AA03)	

<u>Drug-induced transcriptional module: RatLiver-12</u>

59 genes, 24 experiments

Functional enrichment of genes:

Annotation Cluster 1	Enrichment Score: 9.92	
Category	Term	P-value
SP_PIR_KEYWORDS	lipid synthesis	2.04E-16
GOTERM_BP_FAT	GO:0008610~lipid biosynthetic process	2.80E-16
GOTERM_BP_FAT	GO:0016126~sterol biosynthetic process	3.12E-16
GOTERM_BP_FAT	GO:0006695~cholesterol biosynthetic process	8.72E-15
GOTERM_BP_FAT	GO:0016125~sterol metabolic process	6.55E-14
GOTERM_BP_FAT	GO:0008203~cholesterol metabolic process	1.69E-12
GOTERM_BP_FAT	GO:0006694~steroid biosynthetic process	2.18E-12
SP_PIR_KEYWORDS	Steroid biosynthesis	3.41E-12
GOTERM_BP_FAT	GO:0008202~steroid metabolic process	1.58E-10
KEGG_PATHWAY	rno00100:Steroid biosynthesis	3.10E-10
SP_PIR_KEYWORDS	sterol biosynthesis	4.12E-10
KEGG_PATHWAY	rno00900:Terpenoid backbone biosynthesis	1.01E-08

SP_PIR_KEYWORDS	Cholesterol biosynthesis	1.05E-08
GOTERM_BP_FAT	GO:0008299~isoprenoid biosynthetic process	2.20E-06
GOTERM_BP_FAT	GO:0006720~isoprenoid metabolic process	4.23E-05
SP_PIR_KEYWORDS	Isoprene biosynthesis	3.64E-04
SP_PIR_KEYWORDS	magnesium	2.28E-02
**Annotation Cluster 2**	**Enrichment Score: 3.62**	
Category	Term	P-value
KEGG_PATHWAY	rno00100:Steroid biosynthesis	3.10E-10
GOTERM_CC_FAT	GO:0005789~endoplasmic reticulum membrane	1.42E-06
GOTERM_CC_FAT	GO:0042175~nuclear envelope-endoplasmic reticulum network	2.23E-06
GOTERM_BP_FAT	GO:0055114~oxidation reduction	4.01E-06
GOTERM_CC_FAT	GO:0005783~endoplasmic reticulum	5.85E-06
GOTERM_CC_FAT	GO:0044432~endoplasmic reticulum part	7.61E-06
SP_PIR_KEYWORDS	oxidoreductase	1.17E-04
GOTERM_CC_FAT	GO:0012505~endomembrane system	1.61E-04
SP_PIR_KEYWORDS	endoplasmic reticulum	1.65E-04
GOTERM_CC_FAT	GO:0031090~organelle membrane	2.20E-04
SP_PIR_KEYWORDS	membrane	1.37E-02
SP_PIR_KEYWORDS	transmembrane	2.42E-02
UP_SEQ_FEATURE	transmembrane region	2.64E-02
GOTERM_CC_FAT	GO:0016021~integral to membrane	3.51E-01
GOTERM_CC_FAT	GO:0031224~intrinsic to membrane	4.14E-01
UP_SEQ_FEATURE	topological domain:Cytoplasmic	6.37E-01
**Annotation Cluster 3**	**Enrichment Score: 2.91**	
Category	Term	P-value
GOTERM_BP_FAT	GO:0006633~fatty acid biosynthetic process	1.22E-04
GOTERM_BP_FAT	GO:0006631~fatty acid metabolic process	4.10E-04
UP_SEQ_FEATURE	short sequence motif:Histidine box-1	6.32E-04
UP_SEQ_FEATURE	short sequence motif:Histidine box-3	6.32E-04
UP_SEQ_FEATURE	short sequence motif:Histidine box-2	6.32E-04
GOTERM_BP_FAT	GO:0046394~carboxylic acid biosynthetic process	1.61E-03
GOTERM_BP_FAT	GO:0016053~organic acid biosynthetic process	1.61E-03
GOTERM_MF_FAT	GO:0005506~iron ion binding	2.33E-03
KEGG_PATHWAY	rno01040:Biosynthesis of unsaturated fatty acids	8.60E-03
SP_PIR_KEYWORDS	Fatty acid biosynthesis	1.04E-02

Drug annotation:

Drugs	Experiments	Drug annotation	Literature evidence
atorvastatin	1days; exp2848<br>3days; exp2834<br>5days; exp2856	statin; HMG-CoA reductase inhibitor (ATC: C10AA05)	
cerivastatin	1days; exp4378<br>3days; exp4359<br>5days; exp5449	statin; HMG-CoA reductase inhibitor (ATC: C10AA06)	
doxycycline	1days; exp4388	tetracycline antibiotics (ATC: J01AA02)	
fluvastatin	1days; exp3382<br>3days; exp3415<br>3days; exp3470<br>5days; exp3408<br>5days; exp3409	statin; HMG-CoA reductase inhibitor (ATC: C10AA04)	
gemfibrozil	7days; exp2878	fibrates; used to lower lipid levels (ATC: C10AB04)	
lovastatin	1days; exp2966<br>1days; exp2977<br>3days; exp2971<br>3days; exp3000<br>5days; exp2967<br>5days; exp2992	statin; HMG-CoA reductase inhibitor (ATC: C10AA02)	
pravastatin	3days; exp5212<br>5days; exp5182	statin; HMG-CoA reductase inhibitor (ATC: C10AA03)	

simvastatin	1days; exp2824<br>3days; exp2813	statin; HMG-CoA reductase inhibitor (ATC: C10AA01)	
troglitazone	1days; exp6883	antidiabetic (ATC: A10BG01); peroxisome proliferator-activated receptor activator	

Drug-induced transcriptional module: RatLiver-22

88 genes, 16 experiments

Functional enrichment of genes:

**Annotation Cluster 1**	**Enrichment Score: 2.71**	
Category	Term	P-value
GOTERM_BP_FAT	GO:0042330~taxis	1.03E-05
GOTERM_BP_FAT	GO:0006935~chemotaxis	1.03E-05
GOTERM_BP_FAT	GO:0050900~leukocyte migration	3.79E-04
GOTERM_BP_FAT	GO:0030595~leukocyte chemotaxis	1.42E-03
GOTERM_BP_FAT	GO:0007626~locomotory behavior	1.57E-03
GOTERM_BP_FAT	GO:0060326~cell chemotaxis	1.78E-03
GOTERM_BP_FAT	GO:0007610~behavior	3.92E-03
GOTERM_BP_FAT	GO:0016477~cell migration	4.68E-03
GOTERM_BP_FAT	GO:0006954~inflammatory response	9.94E-03
GOTERM_BP_FAT	GO:0051674~localization of cell	1.39E-02
GOTERM_BP_FAT	GO:0048870~cell motility	1.39E-02
GOTERM_MF_FAT	GO:0005125~cytokine activity	2.19E-02
GOTERM_BP_FAT	GO:0006928~cell motion	3.94E-02
**Annotation Cluster 2**	**Enrichment Score: 2.53**	
Category	Term	P-value
GOTERM_BP_FAT	GO:0051240~positive regulation of multicellular organismal process	1.22E-04
KEGG_PATHWAY	rno04623:Cytosolic DNA-sensing pathway	3.58E-04
KEGG_PATHWAY	rno04621:NOD-like receptor signaling pathway	1.12E-03
GOTERM_BP_FAT	GO:0001817~regulation of cytokine production	2.42E-03
GOTERM_BP_FAT	GO:0001819~positive regulation of cytokine production	1.04E-02
GOTERM_BP_FAT	GO:0051046~regulation of secretion	1.61E-02
GOTERM_BP_FAT	GO:0060341~regulation of cellular localization	8.62E-02
**Annotation Cluster 3**	**Enrichment Score: 2.45**	
Category	Term	P-value
GOTERM_BP_FAT	GO:0006955~immune response	1.43E-07
GOTERM_BP_FAT	GO:0045321~leukocyte activation	5.50E-07
GOTERM_BP_FAT	GO:0002684~positive regulation of immune system process	1.20E-06
GOTERM_BP_FAT	GO:0001775~cell activation	1.76E-06
GOTERM_BP_FAT	GO:0050870~positive regulation of T cell activation	4.86E-06
GOTERM_BP_FAT	GO:0046649~lymphocyte activation	7.33E-06
GOTERM_BP_FAT	GO:0051094~positive regulation of developmental process	1.62E-05
GOTERM_BP_FAT	GO:0051251~positive regulation of lymphocyte activation	2.52E-05
GOTERM_BP_FAT	GO:0002696~positive regulation of leukocyte activation	4.16E-05
GOTERM_BP_FAT	GO:0050867~positive regulation of cell activation	5.13E-05
GOTERM_BP_FAT	GO:0042110~T cell activation	5.67E-05
GOTERM_BP_FAT	GO:0050863~regulation of T cell activation	5.67E-05
GOTERM_BP_FAT	GO:0051249~regulation of lymphocyte activation	2.27E-04
GOTERM_BP_FAT	GO:0002694~regulation of leukocyte activation	4.14E-04
GOTERM_BP_FAT	GO:0050865~regulation of cell activation	5.19E-04
GOTERM_CC_FAT	GO:0009897~external side of plasma membrane	5.33E-04
GOTERM_BP_FAT	GO:0045582~positive regulation of T cell differentiation	1.10E-03
GOTERM_BP_FAT	GO:0045621~positive regulation of lymphocyte differentiation	1.31E-03
GOTERM_BP_FAT	GO:0030098~lymphocyte differentiation	2.57E-03
GOTERM_BP_FAT	GO:0045580~regulation of T cell differentiation	3.01E-03
GOTERM_BP_FAT	GO:0045597~positive regulation of cell differentiation	4.10E-03

GOTERM_BP_FAT	GO:0045619~regulation of lymphocyte differentiation	4.89E-03
GOTERM_BP_FAT	GO:0045058~T cell selection	5.17E-03
GOTERM_BP_FAT	GO:0002521~leukocyte differentiation	6.65E-03
GOTERM_BP_FAT	GO:0030217~T cell differentiation	7.36E-03
KEGG_PATHWAY	rno05340:Primary immunodeficiency	2.66E-02
GOTERM_BP_FAT	GO:0030097~hemopoiesis	4.76E-02
GOTERM_BP_FAT	GO:0050671~positive regulation of lymphocyte proliferation	4.93E-02
GOTERM_BP_FAT	GO:0032946~positive regulation of mononuclear cell proliferation	5.08E-02
GOTERM_BP_FAT	GO:0070665~positive regulation of leukocyte proliferation	5.39E-02
GOTERM_CC_FAT	GO:0005768~endosome	5.57E-02
GOTERM_BP_FAT	GO:0048534~hemopoietic or lymphoid organ development	6.43E-02
GOTERM_BP_FAT	GO:0002520~immune system development	7.36E-02
GOTERM_BP_FAT	GO:0044093~positive regulation of molecular function	8.52E-02
GOTERM_BP_FAT	GO:0050670~regulation of lymphocyte proliferation	9.88E-02
GOTERM_BP_FAT	GO:0032944~regulation of mononuclear cell proliferation	1.01E-01
GOTERM_BP_FAT	GO:0070663~regulation of leukocyte proliferation	1.05E-01
GOTERM_BP_FAT	GO:0043085~positive regulation of catalytic activity	1.30E-01
SP_PIR_KEYWORDS	immune response	1.47E-01
GOTERM_BP_FAT	GO:0042325~regulation of phosphorylation	2.21E-01
GOTERM_BP_FAT	GO:0051174~regulation of phosphorus metabolic process	2.41E-01
GOTERM_BP_FAT	GO:0019220~regulation of phosphate metabolic process	2.41E-01
GOTERM_BP_FAT	GO:0045860~positive regulation of protein kinase activity	3.21E-01
GOTERM_BP_FAT	GO:0033674~positive regulation of kinase activity	3.39E-01
GOTERM_BP_FAT	GO:0051347~positive regulation of transferase activity	3.58E-01
GOTERM_BP_FAT	GO:0045859~regulation of protein kinase activity	4.90E-01
GOTERM_BP_FAT	GO:0043549~regulation of kinase activity	5.16E-01
GOTERM_BP_FAT	GO:0051338~regulation of transferase activity	5.42E-01

Drug annotation:

Drugs	Experiments	Drug annotation	Literature evidence
carbon tetrachloride	5days; exp7327	affects the central nervous system, degenerates liver and kidneys	(Reid *et al*, 1997)
doxapram	3days; exp5223<br>5days; exp5230	respiratory stimulant (ATC: R07AB01)	
lead(ii) acetate	1days; exp3323<br>5days; exp3440	causes lead poisoning	
Lipopolysaccharide, *E. coli* O55:B5	.25days; exp4446<br>1days; exp7363<br>1days; exp7617<br>3days; exp5237<br>3days; exp5239<br>3days; exp5347<br>5days; exp5099<br>5days; exp5182	endotoxin, elicits strong immune responses	(Moe *et al*, 2008; Zold *et al*, 2011)
n-nitrosodimethylamine	5days; exp6945	highly toxic, suspected carcinogen	
pralidoxime chloride	5days; exp6260	used against organophosphate poisoning (ATC: V03AB04)	
rofecoxib	1days; exp3088	NSAID (ATC: M01AH02)	

<u>Drug-induced transcriptional module: RatLiver-23</u>

45 genes, 25 experiments

Functional enrichment of genes:

**Annotation Cluster 1**	**Enrichment Score: 3.03**	
Category	Term	P-value
GOTERM_BP_FAT	GO:0010557~positive regulation of macromolecule biosynthetic process	3.72E-07
GOTERM_BP_FAT	GO:0031328~positive regulation of cellular biosynthetic process	6.65E-07
GOTERM_BP_FAT	GO:0009891~positive regulation of biosynthetic process	8.06E-07
GOTERM_BP_FAT	GO:0006350~transcription	9.27E-07
SP_PIR_KEYWORDS	dna-binding	1.38E-06
UP_SEQ_FEATURE	domain:Leucine-zipper	2.92E-06
GOTERM_BP_FAT	GO:0045449~regulation of transcription	3.28E-06
GOTERM_BP_FAT	GO:0010604~positive regulation of macromolecule metabolic process	6.59E-06

GOTERM_MF_FAT	GO:0030528~transcription regulator activity	8.53E-06
INTERPRO	IPR004827:Basic-leucine zipper (bZIP) transcription factor	1.64E-05
UP_SEQ_FEATURE	DNA-binding region:Basic motif	1.76E-05
GOTERM_MF_FAT	GO:0003677~DNA binding	1.97E-05
GOTERM_BP_FAT	GO:0010033~response to organic substance	2.18E-05
GOTERM_MF_FAT	GO:0003700~transcription factor activity	3.29E-05
GOTERM_BP_FAT	GO:0045941~positive regulation of transcription	3.86E-05
GOTERM_MF_FAT	GO:0043565~sequence-specific DNA binding	3.92E-05
GOTERM_BP_FAT	GO:0010628~positive regulation of gene expression	4.66E-05
SMART	SM00338:BRLZ	4.79E-05
SP_PIR_KEYWORDS	transcription regulation	6.55E-05
GOTERM_BP_FAT	GO:0006355~regulation of transcription, DNA-dependent	7.20E-05
GOTERM_BP_FAT	GO:0045893~positive regulation of transcription, DNA-dependent	8.44E-05
INTERPRO	IPR011616:bZIP transcription factor, bZIP-1	8.58E-05
KEGG_PATHWAY	rno04010:MAPK signaling pathway	8.71E-05
GOTERM_MF_FAT	GO:0016563~transcription activator activity	8.80E-05
GOTERM_BP_FAT	GO:0051254~positive regulation of RNA metabolic process	8.97E-05
GOTERM_BP_FAT	GO:0051252~regulation of RNA metabolic process	9.26E-05
SP_PIR_KEYWORDS	DNA binding	9.52E-05
GOTERM_BP_FAT	GO:0045935~positive regulation of nucleobase, nucleoside, nucleotide and nucleic acid metabolic process	1.06E-04
SP_PIR_KEYWORDS	Transcription	1.23E-04
GOTERM_MF_FAT	GO:0003690~double-stranded DNA binding	1.28E-04
GOTERM_BP_FAT	GO:0051173~positive regulation of nitrogen compound metabolic process	1.32E-04
SP_PIR_KEYWORDS	nucleus	1.74E-04
GOTERM_MF_FAT	GO:0043566~structure-specific DNA binding	5.57E-04
GOTERM_BP_FAT	GO:0006357~regulation of transcription from RNA polymerase II promoter	5.75E-04
GOTERM_BP_FAT	GO:0006351~transcription, DNA-dependent	1.06E-03
GOTERM_BP_FAT	GO:0032774~RNA biosynthetic process	1.15E-03
GOTERM_BP_FAT	GO:0009612~response to mechanical stimulus	1.92E-03
GOTERM_MF_FAT	GO:0046983~protein dimerization activity	2.44E-03
GOTERM_CC_FAT	GO:0031981~nuclear lumen	2.45E-03
GOTERM_BP_FAT	GO:0006366~transcription from RNA polymerase II promoter	3.60E-03
GOTERM_BP_FAT	GO:0045944~positive regulation of transcription from RNA polymerase II promoter	7.45E-03
GOTERM_BP_FAT	GO:0051726~regulation of cell cycle	7.98E-03
GOTERM_BP_FAT	GO:0009628~response to abiotic stimulus	8.72E-03
GOTERM_CC_FAT	GO:0070013~intracellular organelle lumen	9.29E-03
SP_PIR_KEYWORDS	Proto-oncogene	1.01E-02
GOTERM_CC_FAT	GO:0043233~organelle lumen	1.13E-02
GOTERM_CC_FAT	GO:0031974~membrane-enclosed lumen	1.30E-02
KEGG_PATHWAY	rno05200:Pathways in cancer	1.34E-02
GOTERM_CC_FAT	GO:0005654~nucleoplasm	1.40E-02
GOTERM_BP_FAT	GO:0051591~response to cAMP	1.80E-02
GOTERM_BP_FAT	GO:0031668~cellular response to extracellular stimulus	1.86E-02
GOTERM_MF_FAT	GO:0046982~protein heterodimerization activity	2.23E-02
GOTERM_BP_FAT	GO:0009314~response to radiation	2.34E-02
KEGG_PATHWAY	rno05210:Colorectal cancer	2.45E-02
GOTERM_BP_FAT	GO:0014070~response to organic cyclic substance	3.02E-02
GOTERM_BP_FAT	GO:0034097~response to cytokine stimulus	5.05E-02
GOTERM_CC_FAT	GO:0044451~nucleoplasm part	6.19E-02
GOTERM_BP_FAT	GO:0007611~learning or memory	6.74E-02
GOTERM_CC_FAT	GO:0043232~intracellular non-membrane-bounded organelle	7.98E-02
GOTERM_CC_FAT	GO:0043228~non-membrane-bounded organelle	7.98E-02
GOTERM_CC_FAT	GO:0005730~nucleolus	1.14E-01
GOTERM_CC_FAT	GO:0044427~chromosomal part	1.40E-01
GOTERM_CC_FAT	GO:0005694~chromosome	1.71E-01
GOTERM_BP_FAT	GO:0042493~response to drug	2.85E-01
GOTERM_CC_FAT	GO:0005856~cytoskeleton	3.25E-01
GOTERM_BP_FAT	GO:0007610~behavior	3.99E-01
GOTERM_CC_FAT	GO:0044430~cytoskeletal part	4.42E-01
GOTERM_BP_FAT	GO:0050877~neurological system process	8.68E-01
GOTERM_BP_FAT	GO:0050890~cognition	9.08E-01

**Annotation Cluster 2**	**Enrichment Score: 2.18**	
Category	Term	P-value
GOTERM_BP_FAT	GO:0042127~regulation of cell proliferation	1.08E-03
GOTERM_BP_FAT	GO:0009968~negative regulation of signal transduction	3.80E-03
GOTERM_BP_FAT	GO:0010648~negative regulation of cell communication	6.22E-03
GOTERM_BP_FAT	GO:0008285~negative regulation of cell proliferation	1.00E-02
GOTERM_MF_FAT	GO:0008134~transcription factor binding	4.88E-02
**Annotation Cluster 3**	**Enrichment Score: 2.00**	
Category	Term	P-value
GOTERM_BP_FAT	GO:0010962~regulation of glucan biosynthetic process	1.67E-03
GOTERM_BP_FAT	GO:0005979~regulation of glycogen biosynthetic process	1.67E-03
GOTERM_BP_FAT	GO:0032885~regulation of polysaccharide biosynthetic process	1.67E-03
GOTERM_BP_FAT	GO:0032881~regulation of polysaccharide metabolic process	2.25E-03
GOTERM_BP_FAT	GO:0043467~regulation of generation of precursor metabolites and energy	4.73E-03
GOTERM_BP_FAT	GO:0043255~regulation of carbohydrate biosynthetic process	4.73E-03
GOTERM_BP_FAT	GO:0010906~regulation of glucose metabolic process	8.42E-03
GOTERM_BP_FAT	GO:0010675~regulation of cellular carbohydrate metabolic process	1.04E-02
GOTERM_BP_FAT	GO:0006109~regulation of carbohydrate metabolic process	1.13E-02
GOTERM_BP_FAT	GO:0031329~regulation of cellular catabolic process	2.02E-02
GOTERM_BP_FAT	GO:0009894~regulation of catabolic process	4.12E-02
GOTERM_CC_FAT	GO:0000267~cell fraction	4.97E-02
KEGG_PATHWAY	rno04910:Insulin signaling pathway	5.97E-02
GOTERM_MF_FAT	GO:0019899~enzyme binding	1.43E-01

Drug annotation:

Drugs	Experiments	Drug annotation	Literature evidence
alfacalcidol	3days; exp6300<br>5days; exp6297	analogue of vitamin D; affects parathyroid hormone levels and immune system, including regulatory T cells	(Koike *et al*, 2005)
amoxicillin	3days; exp6032	beta-lactam antibiotic (ATC: J01CA04)	
cetraxate	5days; exp5732	has cytoprotective effects	
chloroxylenol	3days; exp5394	antimicrobial chemical compound	
clomipramine	3days; exp5101	tricyclic antidepressant (ATC: N06AA04)	
closantel	3days; exp5044<br>5days; exp5206	anthelmintic, insecticide (ATCvet: QP54AA51)	
daunorubicin	3days; exp6882	chemotherapeutic (ATC: L01DB02)	
epirubicin	5days; exp6491	anthracycline drug used for chemotherapy (ATC: L01DB03)	
ethanol	.25days; exp2686	psychoactive (ATC: D08AX08)	
fluocinolone acetonide	3days; exp6583	corticosteroid (ATC: D07AC04)	
geraniol	5days; exp5105	used in perfumery	
ipriflavone	3days; exp4950	synthetic isoflavone; prevents osteoporosis (ATC: M05BX01)	
isoeugenol	3days; exp5524		
methocarbamol	3days; exp4750	central muscle relaxant (ATC: M03BA03)	
mevastatin	3days; exp5187	statin; HMG-CoA reductase inhibitor (ATC: C10AA01)	
oxfendazole	5days; exp4943	benzimidazole anthelmintic	
prednisolone	5days; exp4945	corticosteroid (ATC: D07AA03)	
sertraline	5days; exp4988	antidepressant, selective serotonin reuptake inhibitor (ATC: N06AB06)	
streptozotocin	3days; exp5560	antineoplastic agent (ATC: L01AD04)	
tamoxifen	5days; exp6054	estrogen receptor antagonist (ATC: L02BA01)	
tretinoin	5days; exp5328	acid form of vitamin A; used to treat acne vulgaris and keratosis pilaris; antineoplastic (ATC: L01XX14)	
troglitazone	3days; exp6905	antidiabetic (ATC: A10BG01); peroxisome proliferator-activated receptor activator	
vecuronium bromide	3days; exp6278	muscle relexant; non-depolarizing blocking agent (ATC: M03AC03)	

<u>Drug-induced transcriptional module: RatLiver-36</u>
88 genes, 37 experiments

Functional enrichment of genes:

**Annotation Cluster 1**	**Enrichment Score: 10.92**	
Category	Term	P-value
GOTERM_CC_FAT	GO:0043232~intracellular non-membrane-bounded organelle	1.44E-14
GOTERM_CC_FAT	GO:0043228~non-membrane-bounded organelle	1.44E-14
GOTERM_CC_FAT	GO:0015630~microtubule cytoskeleton	4.30E-13
GOTERM_CC_FAT	GO:0044430~cytoskeletal part	1.17E-08
GOTERM_CC_FAT	GO:0005856~cytoskeleton	2.21E-07
**Annotation Cluster 2**	**Enrichment Score: 10.30**	
Category	Term	P-value
GOTERM_BP_FAT	GO:0007049~cell cycle	1.36E-19
GOTERM_BP_FAT	GO:0022402~cell cycle process	2.43E-17
GOTERM_BP_FAT	GO:0022403~cell cycle phase	3.14E-17
GOTERM_BP_FAT	GO:0000279~M phase	2.15E-15
GOTERM_BP_FAT	GO:0000278~mitotic cell cycle	3.06E-11
GOTERM_BP_FAT	GO:0000087~M phase of mitotic cell cycle	4.05E-10
GOTERM_BP_FAT	GO:0051301~cell division	1.10E-08
SP_PIR_KEYWORDS	cell division	1.93E-08
GOTERM_BP_FAT	GO:0000280~nuclear division	1.10E-07
GOTERM_BP_FAT	GO:0007067~mitosis	1.10E-07
SP_PIR_KEYWORDS	cell cycle	1.53E-07
GOTERM_BP_FAT	GO:0048285~organelle fission	1.73E-07
SP_PIR_KEYWORDS	mitosis	5.26E-05
**Annotation Cluster 3**	**Enrichment Score: 7.50**	
Category	Term	P-value
GOTERM_CC_FAT	GO:0005694~chromosome	1.44E-13
GOTERM_CC_FAT	GO:0044427~chromosomal part	2.64E-13
GOTERM_CC_FAT	GO:0031981~nuclear lumen	1.77E-07
GOTERM_CC_FAT	GO:0031974~membrane-enclosed lumen	4.26E-06
GOTERM_CC_FAT	GO:0070013~intracellular organelle lumen	6.84E-06
GOTERM_CC_FAT	GO:0043233~organelle lumen	1.15E-05
GOTERM_CC_FAT	GO:0005654~nucleoplasm	1.34E-05

Drug annotation:

**Drugs**	**Experiments**	**Drug annotation**	**Literature evidence**
1-naphthylisothiocyanate	3days; exp2675	an isothiocyanate derivative of naphthalene	
acetaminophen	3days; exp2915<br>3days; exp6042	analgesic and antipyretic (ATC: N02BE01)	
allyl alcohol	3days; exp6041		
Anastrozole	1days; exp5123	aromatase-inhibiting drug used against breast cancer (ATC: L02BG03)	
Artemisinin	1days; exp4789	antimalarial (ATC: P01BE01)	
benoxaprofen	1days; exp6369	NSAID (ATC: M01AE06)	
beta-Estradiol	1days; exp3134	estrogen receptor agonist (ATC: G03CA03)	PubChem ID:450
Clotrimazole	1days; exp2748<br>1days; exp3062	antifungal (ATC: D01AC01)	
crotamiton	1days; exp5195	antipruritic and scabicidal (ATCvet: QP53AX04)	
cyproterone acetate	1days; exp4628	antiandrogen (ATC: G03HA01)	
diethylstilbestrol	1days; exp2861	inhibits proliferation of human prostate cancer cell lines; nonsteroidal estrogen (ATC: G03CB02)	(Cvek & Dvorak, 2008)
dipyrone	1days; exp6092	analgesic and antipyretic (ATC: N02BB02)	
disulfiram	1days; exp4338	used to treat chronic alcoholism; proteasome inhibitor	(Cvek & Dvorak, 2008)
ethinylestradiol	1days; exp2779	derivative of estradiol (ATC: L02AA03)	
ethylestrenol	1days; exp6370	anabolic steroid with some progesterone-like activity (ATC: A14AB02)	
geraniol	3days; exp5139	used in perfumery	
granisetron	1days; exp4521	serotonin 5-HT3 receptor antagonist (ATC: A04AA02)	

ketoconazole	1days; exp2952	antifungal drug (ATC: D01AC08)	
methapyrilene	1days; exp6099<br>3days; exp6104	antihistamine and anticholinergic (ATC: R06AC05)	
methimazole	1days; exp4306	antithyroid drug (ATC: H03BB02)	
n,n-dimethylformamide	1days; exp3157<br>3days; exp3164	suspected to cause cancer and birth defects	
nafenopin	1days; exp6622	hypolipidemic agent	
norethindrone	1days; exp2827	progestogen (ATC: G03AC01)	
pantoprazole	1days; exp5043	proton pump inhibitor (ATC: A02BC02)	
pirinixic acid	1days; exp6031	hypolipidemic, peroxisome proliferator-activated receptor	
praziquantel	1days; exp5428	anthelmintic (ATC: P02BA01)	
pyrogallol	1days; exp5628	antiseptic properties	
rofecoxib	1days; exp6367	NSAID (ATC: M01AH02)	
sildenafil	1days; exp3482	used to treat erectile dysfunction and pulmonary arterial hypertension (ATC: G04BE03)	
testosterone	1days; exp6361	steroid hormone from the androgen group	
ticlopidine	1days; exp5469	antiplatelet drug (ATC: B01AC05)	
ticrynafen	1days; exp6130	uric acid-lowering (uricosuric) drug (ATC: C03CC02)	
vinblastine	3days; exp6136	antimicrotubule drug against certain types of cancer (ATC: L01CA01)	

Supplementary Table 6. Experimental results of RNAi-screen for selected genes from CODIM2. We found that siRNA mediated knock-down of 10 out of 24 poorly characterized genes (not previously linked to cellular cholesterol regulation) have a significant effect (labeled in orange) in the filipin and/or the DiI-LDL uptake assays with an inhibitory (yellow) or stimulatory (blue) role. An effect was deemed significant if 2 or more siRNAs had a consistent effect with absolute (average) Z-score >1 and FDR corrected p-value <0.01.

Gene Symbol	Gene Name	siRNA ID	Antisense siRNA	RNAi-Screen: filipin p-value (FDR)	RNAi-Screen: filipin Z-score	RNAi-Screen: DiI-LDL p-value (FDR)	RNAi-Screen: DiI-LDL Z-score
AGR2 (poorly characterized)	anterior gradient homolog 2 (Xenopus laevis)	HSS116219	AUAUGUCUGAGUCCAGAUGAGUUGG	3.9E-01	0.3	7.8E-03	-0.6
		HSS116220	UUUCUUUAAAGCUUGACUGUGUGGG	2.0E-01	-1.6	1.4E-04	-1.0
		s20693	UAACUGUCAGAGAUGGGUCaa	9.1E-01	0.1	3.1E-01	-0.2
		s20694	UUUCUUUAAAGCUUGACUGtg	3.9E-03	-3.6	1.7E-05	-1.0
ARMCX3 (poorly characterized)	armadillo repeat containing, X-linked 3	HSS122362	UAUACUCUUGCUCUCAUUGCUGUCA	1.5E-02	2.5	3.2E-01	-0.2
		HSS122363	AGACAUCUCAACCAAGCAAAGAACC	3.6E-02	-2.8	6.1E-04	-0.7
		s28338	UUCAUGUAUACUUUAAGCCtg	4.7E-03	4.2	3.2E-01	-0.2
		s28339	AUACUCAUUAGUAACAGUCat	9.8E-03	6.1	1.0E+0	0.0
DNAJB9 (poorly characterized)	DnaJ (Hsp40) homolog, subfamily B, member 9	HSS106408	UUGUCAGGGUGGUACUUCAUGGCCA	1.7E-04	6.0	2.4E-02	-0.8
		HSS106410	UGAACAGUCAGUGUAUGUAGUAACC	1.2E-03	3.7	6.6E-05	-1.1
		s8616	UCUUCGUUGAGUGACAGUCct	8.0E-01	-0.4	5.9E-01	-0.1
		s8618	UAGCAUCUGAGAGUGUUUCat	8.6E-01	-0.4	4.8E-01	0.2
DNAJC12 (poorly characterized)	DnaJ (Hsp40) homolog, subfamily C, member 12	HSS148264	UAGCGGGCUCGACUCUCUUCAUUGG	9.1E-01	-0.1	6.1E-03	-0.8
		HSS183512	AACCGAAGAUAGUUCAUCACAUCCC	5.4E-01	-0.6	2.7E-03	-0.9
		s32166	UCACUGAGUCAUUCAAAGCtt	6.5E-03	4.4	7.5E-01	0.0
		s32168	UUUGUUCAACCGAAGAUAGtt	1.5E-02	-2.8	5.5E-01	0.0
DUSP4 (poorly characterized)	dual specificity phosphatase 4	s4373	AAAGCUGAAGACGAACUGCga	2.2E-02	2.0	2.3E-01	0.4
		s4374	AUAGUGUCCUUCAAAGUGGtt	2.8E-01	-0.8	7.1E-14	-1.8
		VHS40973	UACUCUAUGGCUUCCAUGAACCAGG	3.7E-01	-0.8	6.5E-06	-1.2
		VHS40975	AGACAUUCAACAGAGCCGUGAUGCC	3.0E-01	1.1	3.7E-04	-1.1
EIF2AK3 (poorly characterized)	eukaryotic translation initiation factor 2-alpha kinase 3	HSS114059	UUUACUGUGAAGAAACUCCACUGCC	2.7E-04	5.7	1.8E-01	-0.3
		HSS190343	UUCAGAAGAAGAUUUGCUACUGGUG	7.9E-01	0.0	1.2E-06	-1.2
		s18102	UCUUGUUCCAUUUCGUCACta	1.6E-01	1.0	3.2E-06	0.9
		s18103	UUUGCGGAUAUUCUUGUUGta	1.9E-01	1.1	2.4E-01	0.4
FADS1	fatty acid desaturase 1	HSS106078	UGAUGUGGAAGGCCACAAAGGGAUC	3.2E-01	1.2	1.7E-01	-0.3
		HSS106079	UAAUCAUCCAGGCCAAGUCCACCCA	1.3E-03	4.3	9.9E-02	-0.5
		s8207	UUUAGUGAGUGGAUGAUGUcg	5.0E-01	-0.5	6.3E-01	0.1
FAM117A (poorly characterized)	family with sequence similarity 117, member A	s37630	UCACCUUCUAGCUCUUGCCag	2.4E-01	1.0	8.1E-02	0.5
		s37631	ACUUGGAAAUCUCUUUUCGgt	1.3E-01	1.5	2.3E-01	0.4
FASN	fatty acid synthase	s5031	UUGAUGAUCAGGUCCACGGcg	6.1E-01	-0.5	5.1E-07	-1.2
		s5032	UUUCUCACAGAUACGCUCCat	1.8E-02	3.5	1.2E-02	-0.6
FGFBP1 (poorly characterized)	fibroblast growth factor binding protein 1	HSS115142	AUUGAGUGCACUCAACCUUGAGAGA	2.9E-02	2.1	2.0E-03	-0.8
		HSS190756	AAUAGACUCUCUCAUCCUUGAGCUU	1.7E-03	3.4	1.6E-02	-0.6
		s19392	AAAAUUCAUGGUCCAAUUGag	3.5E-03	5.8	4.0E-09	1.5
		s19394	UUGAGCUUUAGGCAUGAGGtt	1.7E-02	4.3	3.3E-02	0.5
FKBP11 (poorly characterized)	FK506 binding protein 11, 19 kDa	s224198	UGUAUGUGAAGCGUGUCUCca	4.3E-02	1.9	4.9E-01	0.2
		s27896	UCUAUAACCAGAGGGUCUCtg	1.5E-01	1.1	6.3E-01	0.0
HMGCR	3-hydroxy-	HSS104864	UUUGCAUGCUCCUUGAACACCUAGC	1.8E-02	2.7	1.7E-01	0.4

	3-methylglutaryl-Coenzyme A reductase	HSS179267	AUAACAUUCUCACAACAAGCUCCCA	4.4E-03	2.9	7.2E-01	0.0
		s143	UCCUUUAUCACUGCGAACCct	2.5E-02	3.7	1.3E-08	1.5
HSPA5 (poorly characterized)	heat shock 70kDa protein 5 (glucose-regulated protein, 78kDa)	HSS105076	UUCAAAUUUGGCCCGAGUCAGGGUC	3.8E-03	4.5	3.0E-01	-0.1
		HSS105077	UACCCUUGUCUUCAGCUGUCACUCG	6.3E-02	1.7	6.0E-02	-0.4
		s6980	UCUAGUAUCAAUGCGCUCCtt	1.3E-02	2.8	9.4E-01	0.0
		s6981	UACGCUACAGCUUCAUCUGgg	1.6E-01	-1.1	4.4E-01	0.3
IDH1 (poorly characterized)	isocitrate dehydrogenase 1 (NADP+), soluble	HSS105181	AAAUGAUUCGUGUCAUUUCAUCUCC	6.3E-02	1.4	1.7E-01	-0.4
		HSS105182	AAAUCAGUUGCUCUGUAUUGAUCCC	6.8E-01	-0.9	7.0E-05	-1.2
		s7120	UCAAUUCCCAAAUGAUUCGtg	3.0E-01	-0.8	1.1E-06	-1.2
		s7121	UUGGUGACUUGGUCGUUGGtg	8.3E-02	1.7	2.6E-01	-0.3
INSIG1	insulin induced gene 1	s7465	AAGAGAGUGACAUUCCUCUgg	6.6E-01	0.2	2.9E-15	2.0
		s7466	UUUGGAUAAAUAUUAUUCCta	4.1E-01	0.6	1.5E-02	-0.6
ISG20 (poorly characterized)	interferon stimulated exonuclease gene 20kDa	HSS142681	UAAUCGGUGAUCUCUCCCUCAGGCC	2.2E-02	2.6	6.8E-04	-1.0
		HSS142682	AGCCGCUCAUGUCCUCUUUCAGUGC	1.8E-02	-2.1	9.0E-03	-0.7
		s7524	UCUGUAAUCGGUGAUCUCUcc	3.1E-02	3.1	8.3E-01	0.0
		s7525	AUUUGAUAGAGCUCCAUCGtt	2.9E-02	2.4	6.3E-01	0.2
LDLR	low density lipoprotein receptor	HSS106010	UUCUCAUUUCCUCUGCCAGCAACGU	4.4E-04	-4.7	1.0E-10	-1.7
		HSS106011	UGUCCUUGCAGUCAUAUUCCCGGUC	1.7E-04	-5.5	0.0E+0	-2.0
		s224006	UAUCUUCGCAUCUUCGCUGgg	4.3E-04	-5.0	0.0E+0	-2.8
LZTFL1 (poorly characterized)	leucine zipper transcription factor-like 1	HSS123079	AAAUUUCCUUGUUUAGGAGUUCUGC	5.7E-02	-1.5	6.1E-03	-0.7
		HSS123080	UUCAAUGGUCUUCAACCUUGACUUC	1.1E-02	-2.8	1.8E-05	-1.4
		s29234	UAAGUUACUUAAGUCUUGGgc	1.1E-01	-1.2	2.5E-03	-0.7
		s29235	UAAGAUACCACUUCUCAGCtt	8.6E-01	-0.3	8.2E-01	-0.1
NPC1	Niemann-Pick disease, type C1	HSS107272	AAAUGGUGCAGUUCGUGUUAUACGG	6.4E-08	15.1	2.3E-01	-0.3
		HSS107273	AAUAAUCGUCGAUCCAGGACGAGGG	1.8E-03	5.5	1.6E-01	-0.6
		s237198	AUAUUGCUAUCACAAUUGGtc	1.1E-04	13.9	3.5E-01	-0.1
NUCB2 (poorly characterized)	nucleobindin 2	s9773	UAUAUCUUGAAGGGAAUCCaa	6.7E-01	0.3	9.0E-03	0.6
		s9774	UUCUUAACCUUCCUACUUCtt	7.5E-01	0.0	7.5E-01	-0.1
PGM3 (poorly characterized)	phosphoglucomutase 3	HSS107909	UUCAUUGGACUUAAUUUCCAUUCCC	1.1E-04	7.2	5.4E-02	-0.5
		HSS107910	UAAGGAAACUGCUAAUUAACGUUGC	1.7E-04	6.0	6.1E-03	-0.7
		s10409	UGAUUGCCUCCUGUAAUCCtg	5.7E-02	1.5	9.9E-03	-0.6
		s10410	UCUUGUUGCAGAUUCACAGct	4.0E-01	-0.7	1.2E-02	0.6
RGS1 (poorly characterized)	regulator of G-protein signaling 1	HSS143614	UUUCAUUCCAGAUUCCAGAUGUGGG	1.7E-03	3.8	3.0E-01	-0.2
		HSS143615	UUAUAUAUCUCUUCUGCUUUACAGG	5.4E-01	-1.1	6.5E-06	-1.1
		s11986	UCAUAGAUCUCAGGUAUGCtt	9.2E-01	-0.1	1.5E-02	-0.6
		s11988	UAUUGAUUUGUUUAGCAGCat	6.2E-01	-0.3	1.4E-01	-0.3
SEC24D (poorly characterized)	SEC24 family, member D (S. cerevisiae)	s19116	UUGUUGUCUAUCCAAGUGCat	1.7E-01	1.1	4.1E-01	0.2
		s19117	UGAUUAACUAGAAUUUCCCgg	8.3E-02	1.4	4.8E-01	-0.2
SLC2A14 (poorly characterized)	solute carrier family 2 (facilitated glucose transporter), member 14	HSS152070	AAGCGGUUAACAAAGAGUCCGACGG	2.0E-01	0.7	4.3E-08	-1.5
		HSS152071	AUGAGUUCCAGACCAAAGAUCUGGG	3.8E-03	3.9	1.7E-02	-0.6
		s44617	AUCAUAUGCAGAGUCCUUCtt	3.0E-01	0.6	3.1E-02	-0.6
		s44619	AAGAUAAUAAAAACGUAGGct	5.4E-01	0.4	6.0E-02	0.4
SPP1 (poorly characterized)	secreted phosphoprotein 1	HSS144072	AUUGCUCUCAUCAUUGGCUUUCCGC	8.6E-01	-0.3	5.1E-02	-0.4
		HSS186069	AUCAGAAGGCGCGUUCAGGUCCUGG	3.8E-03	-3.4	3.0E-04	-1.1
		s13375	AGAACUUCCAGAAUCAGCCtg	1.1E-02	3.5	1.9E-03	0.8
		s13377	UUUCGUUGGACUUACUUGGaa	1.2E-01	1.1	2.5E-01	0.3

SQLE	squalene epoxidase	s198	UUAGUUGAUGCAGGGAAUCat	4.0E-01	-0.6	2.1E-06	-1.1
		s199	UUCCUUGAUAUCUCCAGUCtc	1.5E-02	4.4	2.6E-01	0.4
TMEM41B (poorly characterized)	transmembrane protein 41B	HSS162367	AUCGUUCGGCGACUCUGCCUUUCGC	8.6E-01	-1.1	1.7E-05	-1.0
		HSS162368	AAAUGGACACCAAUAUAAGGAGUGA	3.7E-01	-1.8	1.6E-01	-0.3
		s53992	UUGUAUACAACUGGUCUCCca	6.1E-02	-1.3	0.0E+0	-2.3
		s53993	UGCUGAUCCAGCUUCUACCca	7.5E-03	4.4	7.2E-01	0.1
TRIM2 (poorly characterized)	tripartite motif containing 2	HSS118585	UUUCCAGUGCUGUACAUGCUUGCGG	8.6E-01	-0.5	4.4E-01	0.2
		HSS118586	UUAUUUCUUCCUUUGGUACCCACUC	1.1E-03	-4.8	2.8E-07	-1.5
		s23555	UGGUGGUUAAGAUCGUCCCga	1.1E-02	3.3	2.9E-11	-1.5
		s23556	UACCGUUCCAGGCAUAUACtg	9.9E-01	-0.2	3.7E-04	-0.8
TUBA1A (poorly characterized)	tubulin, alpha 1a	HSS144577	UGGAUGGUACGCUUGGUCUUGAUGG	3.8E-02	1.7	1.8E-03	-1.0
		HSS144578	UAGUAUAGGUUGGACGCUCAAUAUC	2.6E-01	-2.1	3.9E-02	-0.6
		s15400	ACAAUUUGACCUAUUAACCta	4.5E-02	2.1	1.8E-07	1.4
		s15401	UAACUGAUCAGACCACAACtt	3.5E-01	0.6	3.9E-02	-0.5
WIPI1 (poorly characterized)	WD repeat domain, phosphoinositide interacting 1	HSS123809	AUAUCCAGGAGGGUCUUCAACAGCU	2.9E-02	-3.3	2.9E-01	-0.2
		HSS123810	AUAGAGAGAGCACAUAGACCUGUUG	1.7E-03	-4.1	3.0E-01	-0.3
		s30081	UCAUGGGCAGCAAUAGUGCag	5.1E-02	-1.6	2.8E-02	-0.5
		s30083	UUCUGGAUCGUUGAGAGGGta	6.3E-02	2.4	4.4E-01	-0.3
ZNF165 (poorly characterized)	zinc finger protein 165	HSS111629	UGACAUUAAGUGCUGGUCCAGGAGG	6.2E-01	0.0	4.7E-06	-1.3
		HSS111630	UUCCAGCUUUGGCAUGGAUCUACUG	2.4E-02	-2.6	2.6E-05	-1.2
		s15225	UGUUUAGCAAGUUUUGGGCtc	7.5E-03	3.2	1.1E-01	0.4
		s15226	UUUACCAAAACCUUCAUCCtg	8.9E-01	0.0	2.4E-02	0.6

Supplementary References

Akira T, Kaori S, Tomohiko A & Masamichi H (2000) Effects of intraperitoneally administered antibiotics on rat peritoneal mesothelial cell proliferation. *Boei Ika Daigakko Zasshi* **25:** 194–200

Baliga BS, Pronczuk AW & Munro HN (1969) Mechanism of cycloheximide inhibition of protein synthesis in a cell-free system prepared from rat liver. *J Biol Chem* **244:** 4480–9

Bantscheff M, Hopf C, Savitski MM, Dittmann A, Grandi P, Michon A-M, Schlegl J, Abraham Y, Becher I, Bergamini G, Boesche M, Delling M, Dümpelfeld B, Eberhard D, Huthmacher C, Mathieson T, Poeckel D, Reader V, Strunk K, Sweetman G, *et al* (2011) Chemoproteomics profiling of HDAC inhibitors reveals selective targeting of HDAC complexes. *Nat Biotechnol* **29:** 255–65

Barnes PJ (2006) How corticosteroid control inflammation: Quintiles Prize Lecture 2005. *Br J Pharmacol* **148:** 245–54

Beutler JA, Karbon EW, Brubaker AN, Malik R, Curtis DR & Enna SJ (1985) Securinine alkaloids: a new class of GABA receptor antagonist. *Brain Res* **330:** 135–40

Le Blanc M, Russo J, Kudelka AP & Smith JA (2002) An in vitro study of inhibitory activity of gossypol, a cottonseed extract, in human carcinoma cell lines. *Pharmacol Res* **46:** 551–5

Braun DL, Brown BB & Feldman RG (1956) The pharmacologic activity of alpha-(4-piperidyl)-benzhydrol hydrochloride (azacyclonol hydrochloride); an ataractive agent. *J Pharmacol Exp Ther* **118:** 153–61

Brink JJ & Lepage GA (1965) 9-Beta-D-Arabinofuranosyladenine as an inhibitor of metabolism in normal and neoplastic cells. *Can J Biochem Physiol* **43:** 1–15

Błaszczyk A & Skolimowski J (2005) Apoptosis and cytotoxicity caused by ethoxyquin and two of its salts. *Cell Mol Biol Lett* **10:** 15–21

Canfran-Duque A, Casado ME, Pastor O, Sanchez-Wandelmer J, De la Pena G, Lerma M, Mariscal P, Bracher F, Lasuncion MA & Busto R (2012) Atypical antipsychotics alter cholesterol and fatty acid metabolism in vitro. *J Lipid Res*

Chalfant ML, Peterson-Yantorno K, O'Brien TG & Civan MM (1996) Regulation of epithelial Na+ channels from M-1 cortical collecting duct cells. *Am J Physiol* **271:** F861–70

Chan J (2004) Eukaryotic protein synthesis inhibitors identified by comparison of cytotoxicity profiles. *RNA* **10:** 528–543

Charles AK & Darbre PD (2009) Oestrogenic activity of benzyl salicylate, benzyl benzoate and butylphenylmethylpropional (Lilial) in MCF7 human breast cancer cells in vitro. *J Appl Toxicol* **29:** 422–34

Chen Y, Tsai Y-H & Tseng S-H (2011) The potential of tetrandrine as a protective agent for ischemic stroke. *Molecules* **16:** 8020–32

Cho RJ, Huang M, Campbell MJ, Dong H, Steinmetz L, Sapinoso L, Hampton G, Elledge SJ, Davis RW & Lockhart DJ (2001) Transcriptional regulation and function during the human cell cycle. *Nat Genet* **27:** 48–54

Choi S-H, Choi D-H, Song K-S, Shin K-H & Chun B-G (2002) Zaprinast, an inhibitor of cGMP-selective phosphodiesterases, enhances the secretion of TNF-alpha and IL-1beta and the expression of iNOS and MHC class II molecules in rat microglial cells. *J Neurosci Res* **67:** 411–21

Colsky J, Meiselas LE, Rosen SJ & Schulman I (1955) Response of patients with leukemia to 8-azaguanine. *Blood* **10:** 482–92

Crawford DF & Piwnica-Worms H (2001) The G(2) DNA damage checkpoint delays expression of genes encoding mitotic regulators. *J Biol Chem* **276:** 37166–77

Cvek B & Dvorak Z (2008) The value of proteasome inhibition in cancer. Can the old drug, disulfiram, have a bright new future as a novel proteasome inhibitor? *Drug Discov Today* **13:** 716–22

Davis AP, King BL, Mockus S, Murphy CG, Saraceni-Richards C, Rosenstein M, Wiegers T & Mattingly CJ (2011) The Comparative Toxicogenomics Database: update 2011. *Nucleic Acids Res* **39:** D1067–72

Deitmer JW & Ellis D (1978) Changes in the intracellular sodium activity of sheep heart Purkinje fibres produced by calcium and other divalent cations. *J Physiol* **277:** 437–53

Eisenreich WJ, Höfner G & Bracher F (2003) Alkaloids from Croton flavens L. and their affinities to GABA-receptors. *Nat Prod Res* **17:** 437–40

Erlenkamp S, Gretzer B, Zillikens S, Glitsch HG, Pusch H, Staroske T & Welzel P (1998) Na+/K+ pump inhibition and positive inotropic effect of digitoxigenin and some C-22-substituted derivatives in sheep cardiac preparations. *Naunyn Schmiedebergs Arch Pharmacol* **357:** 54–62

Falkay G & Kovács L (1986) Affinity of tocolytic agents on human placental and myometrial beta-adrenergic receptors. *J Perinat Med* **14:** 109–13

Fernø J, Raeder MB, Vik-Mo AO, Skrede S, Glambek M, Tronstad K-J, Breilid H, Løvlie R, Berge RK, Stansberg C & Steen VM (2005) Antipsychotic drugs activate SREBP-regulated expression of lipid biosynthetic genes in cultured human glioma cells: a novel mechanism of action? *Pharmacogenomics J* **5:** 298–304

Forman BM, Tontonoz P, Chen J, Brun RP, Spiegelman BM & Evans RM (1995) 15-Deoxy-delta 12, 14-prostaglandin J2 is a ligand for the adipocyte determination factor PPAR gamma. *Cell* **83:** 803–12

Fukuen S, Iwaki M, Yasui A, Makishima M, Matsuda M & Shimomura I (2005) Sulfonylurea agents exhibit peroxisome proliferator-activated receptor gamma agonistic activity. *J Biol Chem* **280:** 23653–9

Fulda S (2008) Betulinic Acid for cancer treatment and prevention. *Int J Mol Sci* **9:** 1096–107

Giammarioli AM, Maselli A, Casagrande A, Gambardella L, Gallina A, Spada M, Giovannetti A, Proietti E, Malorni W & Pierdominici M (2008) Pyrimethamine induces apoptosis of melanoma cells via a caspase and cathepsin double-edged mechanism. *Cancer Res* **68:** 5291–300

Gonzalo-Garijo MA, Cordobés-Duran C, Lamilla-Yerga AM & Moreno-Gastón I (2007) Severe immediate reaction to nabumetone. *J Investig Allergol Clin Immunol* **17:** 274–6

Gopal YNV, Arora TS & Van Dyke MW (2007) Parthenolide specifically depletes histone deacetylase 1 protein and induces cell death through ataxia telangiectasia mutated. *Chem Biol* **14:** 813–23

Gordaliza M, García PA, Del Corral JMM, Castro MA & Gómez-Zurita MA (2004) Podophyllotoxin: distribution, sources, applications and new cytotoxic derivatives. *Toxicon* **44:** 441–59

Graham D & Langer SZ (1992) Advances in sodium-ion coupled biogenic amine transporters. *Life Sci* **51:** 631–45

Griffin DS & Segall HJ (1986) Genotoxicity and cytotoxicity of selected pyrrolizidine alkaloids, a possible alkenal metabolite of the alkaloids, and related alkenals. *Toxicol Appl Pharmacol* **86:** 227–34

Hamon M, Mallat M, Herbet A, Nelson DL, Audinot M, Pichat L & Glowinski J (1981) [3H]Metergoline: a new ligand of serotonin receptors in the rat brain. *J Neurochem* **36:** 613–26

Haraguchi K, Ito K, Kotaki H, Sawada Y & Iga T (1997) Prediction of drug-induced catalepsy based on dopamine D1, D2, and muscarinic acetylcholine receptor occupancies. *Drug Metab Dispos* **25:** 675–84

Harris DM, Li L, Chen M, Lagunero FT, Go VLW & Boros LG (2012) Diverse mechanisms of growth inhibition by luteolin, resveratrol, and quercetin in MIA PaCa-2 cells: a comparative glucose tracer study with the fatty acid synthase inhibitor C75. *Metabolomics* **8:** 201–210

Hertz R, Li MC & Spencer DB (1956) Effect of methotrexate therapy upon choriocarcinoma and chorioadenoma. *Proc Soc Exp Biol Med* **93:** 361–6

Van Hoey NM Effect of cyclobenzaprine on tricyclic antidepressant assays. *Ann Pharmacother* **39:** 1314–7

Hsu S-P, Ho P-Y, Juan S-H, Liang Y-C & Lee W-S (2008) Progesterone inhibits human endothelial cell proliferation through a p53-dependent pathway. *Cell Mol Life Sci* **65:** 3839–50

Ittarat I, Asawamahasakda W, Bartlett MS, Smith JW & Meshnick SR (1995) Effects of atovaquone and other inhibitors on Pneumocystis carinii dihydroorotate dehydrogenase. *Antimicrob Agents Chemother (Bethesda)* **39:** 325–8

Izaguirre Roncal LB, Gonzalvo Ibáñez FJ, Pinilla Lozano I, Pablo Júlvez L, Abecia Martínez E & Honrubia López FM (2001) [Trifluoperazine's action on fibroblast proliferation in cell cultures. Comparison of different doses]. *Archivos de la Sociedad Española de Oftalmología* **76:** 471–6

Jiménez A, Carrasco L & Vázquez D (1977) Enzymic and nonenzymic translocation by yeast polysomes. Site of action of a number of inhibitors. *Biochemistry* **16:** 4727–30

Jinwal UK, Miyata Y, Koren J, Jones JR, Trotter JH, Chang L, O'Leary J, Morgan D, Lee DC, Shults CL, Rousaki A, Weeber EJ, Zuiderweg ERP, Gestwicki JE & Dickey CA (2009) Chemical manipulation of hsp70 ATPase activity regulates tau stability. *J Neurosci* **29:** 12079–88

Jordan MA, Toso RJ, Thrower D & Wilson L (1993) Mechanism of mitotic block and inhibition of cell proliferation by taxol at low concentrations. *Proc Natl Acad Sci U S A* **90:** 9552–6

Kanakis CD, Tarantilis PA, Polissiou MG, Diamantoglou S & Tajmir-Riahi HA (2007) An overview of DNA and RNA bindings to antioxidant flavonoids. *Cell Biochem Biophys* **49:** 29–36

Kanakis CD, Tarantilis PA, Polissiou MG & Tajmir-Riahi H-A (2006) Interaction of antioxidant flavonoids with tRNA: intercalation or external binding and comparison with flavonoid-DNA adducts. *DNA Cell Biol* **25:** 116–23

Kelder T, Van Iersel MP, Hanspers K, Kutmon M, Conklin BR, Evelo CT & Pico AR (2012) WikiPathways: building research communities on biological pathways. *Nucleic Acids Res* **40:** D1301–7

Kim H-J, Kim J-Y, Meng Z, Wang LH, Liu F, Conrads TP, Burke TR, Veenstra TD & Farrar WL (2007) 15-deoxy-Delta12,14-prostaglandin J2 inhibits transcriptional activity of estrogen receptor-alpha via covalent modification of DNA-binding domain. *Cancer Res* **67:** 2595–602

Kitamura S (1980) The used to treat asthmatic patients using an alpha-adrenergic receptor blocking agent, co-dergocrine mesylate ('Hydergine'). *Pharmatherapeutica* **2:** 330–6

Koike H, Ito K, Takezawa Y, Oyama T, Yamanaka H & Suzuki K (2005) Insulin-like growth factor binding protein-6 inhibits prostate cancer cell proliferation: implication for anticancer effect of diethylstilbestrol in hormone refractory prostate cancer. *Br J Cancer* **92:** 1538–44

Konishi Y, Morimoto T, Komaki K, Yamakawa T, Mituyama N, Tanaka T, Oomine Y & Monden Y (1988) [A case of advanced breast cancer successfully treated with combined tamoxifen and epitiostanol]. *Gan To Kagaku Ryoho* **15:** 2163–7

Kristiana I, Sharpe LJ, Catts VS, Lutze-Mann LH & Brown AJ (2010) Antipsychotic drugs upregulate lipogenic gene expression by disrupting intracellular trafficking of lipoprotein-derived cholesterol. *Pharmacogenomics J* **10:** 396–407

Lee A-H, Iwakoshi NN & Glimcher LH (2003) XBP-1 regulates a subset of endoplasmic reticulum resident chaperone genes in the unfolded protein response. *Mol Cell Biol* **23:** 7448–59

De Leeuw van Weenen JE, Parlevliet ET, Maechler P, Havekes LM, Romijn JA, Ouwens DM, Pijl H & Guigas B (2010) The dopamine receptor D2 agonist bromocriptine inhibits glucose-stimulated insulin secretion by direct activation of the alpha2-adrenergic receptors in beta cells. *Biochem Pharmacol* **79:** 1827–36

Lepage GA (1963) Basic biochemical effects and mechanism of action of 6-thioguanine. *Cancer Res* **23:** 1202–6

Lessner H, Jonsson U, Loeb V & Larsen W (1963) Preliminary clinical experience with trimethylcolchicinic acid methyl ether d-tartrate (TMCA) in various malignancies. *Cancer Chemother Rep* **27:** 33–8

Lijnen P, Fagard R & Petrov V (1998) Proliferation of human peripheral blood mononuclear cells during calcium channel blockade. *Am J Hypertens* **11:** 1461–8

Liu KX, Kato Y, Yamazaki M, Higuchi O, Nakamura T & Sugiyama Y (1993) Decrease in the hepatic clearance of hepatocyte growth factor in carbon tetrachloride-intoxicated rats. *Hepatology* **17:** 651–60

López-Franco O, Hernández-Vargas P, Ortiz-Muñoz G, Sanjuán G, Suzuki Y, Ortega L, Blanco J, Egido J & Gómez-Guerrero C (2006) Parthenolide modulates the NF-kappaB-mediated inflammatory responses in experimental atherosclerosis. *Arterioscler Thromb Vasc Biol* **26:** 1864–70

Maira S-M, Stauffer F, Schnell C & García-Echeverría C (2009) PI3K inhibitors for cancer treatment: where do we stand? *Biochem Soc Trans* **37:** 265–72

Makovec F, Bani M, Cereda R, Chisté R, Pacini MA, Revel L, Rovati LA, Rovati LC & Setnikar I (1987) Pharmacological properties of lorglumide as a member of a new class of cholecystokinin antagonists. *Arzneimittelforschung* **37:** 1265–8

Marcu MG, Doyle M, Bertolotti A, Ron D, Hendershot L & Neckers L (2002) Heat shock protein 90 modulates the unfolded protein response by stabilizing IRE1alpha. *Mol Cell Biol* **22:** 8506–13

Marshall FN & Williamson HE (1964) Natruretic response during infusion of beta-diethylaminoethyl-diphenylpropyl acetate hydrocloride (SKF 525-a). *J Pharmacol Exp Ther* **143:** 395–400

Matheson AJ & Noble S (2000) Racecadotril. *Drugs* **59:** 829–35; discussion 836–7

Mayo W, Lemaire V, Malaterre J, Rodriguez JJ, Cayre M, Stewart MG, Kharouby M, Rougon G, Le Moal M, Piazza P V & Abrous DN (2005) Pregnenolone sulfate enhances neurogenesis and PSA-NCAM in young and aged hippocampus. *Neurobiol Aging* **26:** 103–14

McLean HM, Khalil MA, Heiman AS & Lee HJ (1994) Novel fluorinated antiinflammatory steroid with reduced side effects: methyl 9 alpha-fluoroprednisolone-16-carboxylate. *J Pharm Sci* **83:** 476–80

McMahon D & Langstroth P (1972) The effects of canavanine and of arginine starvation on macromolecular synthesis in Chlamydomonas reinhardi. *J Gen Microbiol* **73:** 239–250

Miyata Y (2005a) Hsp90 inhibitor geldanamycin and its derivatives as novel cancer chemotherapeutic agents. *Curr Pharm Des* **11:** 1131–8

Miyata Y (2005b) Hsp90 inhibitor geldanamycin and its derivatives as novel cancer chemotherapeutic agents. *Curr Pharm Des* **11:** 1131–8

Mo Q, Lu S & Simon NG (2006) Dehydroepiandrosterone and its metabolites: differential effects on androgen receptor trafficking and transcriptional activity. *J Steroid Biochem Mol Biol* **99:** 50–8

Moe S, Wazny LD & Martin JE (2008) Oral calcitriol versus oral alfacalcidol for the used to treat secondary hyperparathyroidism in patients receiving hemodialysis: a randomized, crossover trial. *Can J Clin Pharmacol* **15:** e36–43

Monachon MA, Burkard WP, Jalfre M & Haefely W (1972) Blockade of central 5-hydroxytryptamine receptors by methiothepin. *Naunyn Schmiedebergs Arch Pharmacol* **274:** 192–7

Nafisi S, Malekabady ZM & Khalilzadeh MA (2010) Interaction of β-carboline alkaloids with RNA. *DNA Cell Biol* **29:** 753–61

Nelson EA, Walker SR, Kepich A, Gashin LB, Hideshima T, Ikeda H, Chauhan D, Anderson KC & Frank DA (2008) Nifuroxazide inhibits survival of multiple myeloma cells by directly inhibiting STAT3. *Blood* **112:** 5095–102

Nose K (1984) Inhibition by flavonoids of RNA synthesis in permeable WI-38 cells and of transcription by RNA polymerase II. *Biochem Pharmacol* **33:** 3823–7

Nusser E, Donath H & Russ W (1965) [On the circulatory action of depot-Effortil in patients with hypotonic regulation circulator disorders]. *Med Welt* **32:** 1824–7

Panzer A, Joubert AM, Bianchi PC, Hamel E & Seegers JC (2001) The effects of chelidonine on tubulin polymerisation, cell cycle progression and selected signal transmission pathways. *Eur J Cell Biol* **80:** 111–8

Patel JB & Malick JB (1982) Pharmacological properties of tracazolate: a new non-benzodiazepine anxiolytic agent. *Eur J Pharmacol* **78:** 323–33

Rada RT & Donlon PT (1974) Piperacetazine in ambulatory chronic schizophrenic patients. *Curr Ther Res Clin Exp* **16:** 124–9

Reid RR, Prodeus AP, Khan W, Hsu T, Rosen FS & Carroll MC (1997) Endotoxin shock in antibody-deficient mice: unraveling the role of natural antibody and complement in the clearance of lipopolysaccharide. *J Immunol* **159:** 970–5

Rotrosen J, Angrist BM, Gershon S, Aronson M, Gruen P, Sachar EJ, Denning RK, Matthysse S, Stanley M & Wilk S (1978) Thiethylperazine; clinical antipsychotic efficacy and correlation with potency in predictive systems. *Arch Gen Psychiatry* **35:** 1112–8

Ruffolo RR, Cohen ML, Messick K & Horng JS (1987) Alpha-2-adrenoceptor-mediated effects of pergolide. *Pharmacology* **35:** 148–54

Sandberg K & Coyle JT (1985) Characterization of [3H]hemicholinium-3 binding associated with neuronal choline uptake sites in rat brain membranes. *Brain Res* **348:** 321–30

Santos Cerqueira G, Dos Santos E Silva G, Rios Vasconcelos E, Fragoso de Freitas AP, Arcanjo Moura B, Silveira Macedo D, Lopes Souto A, Barbosa Filho JM, De Almeida Leal LK, De Castro Brito GA, Souccar C & De Barros Viana GS

(2012) Effects of hecogenin and its possible mechanism of action on experimental models of gastric ulcer in mice. *Eur J Pharmacol*

Scarsi M, Podvinec M, Roth A, Hug H, Kersten S, Albrecht H, Schwede T, Meyer UA & Rücker C (2007) Sulfonylureas and glinides exhibit peroxisome proliferator-activated receptor gamma activity: a combined virtual screening and biological assay approach. *Mol Pharmacol* **71:** 398–406

Schewe T (1995) Molecular actions of ebselen--an antiinflammatory antioxidant. *Gen Pharmacol* **26:** 1153–69

Schreiner J, Nell G & Loeschke K (1980) Effect of diphenolic laxatives on Na+-K+-activated ATPase and cyclic nucleotide content of rat colon mucosa in vivo. *Naunyn Schmiedebergs Arch Pharmacol* **313:** 249–55

Seifert WF, Bosma A, Brouwer A, Hendriks HF, Roholl PJ, Van Leeuwen RE, Van Thiel-de Ruiter GC, Seifert-Bock I & Knook DL (1994) Vitamin A deficiency potentiates carbon tetrachloride-induced liver fibrosis in rats. *Hepatology* **19:** 193–201

Sharma S V, Agatsuma T & Nakano H (1998) Targeting of the protein chaperone, HSP90, by the transformation suppressing agent, radicicol. *Oncogene* **16:** 2639–45

Sieber SM, Whang-Peng J, Johns DG & Adamson RH (1973) Effects of hycanthone on rapidly proliferating cells. *Biochem Pharmacol* **22:** 1253–62

Spies CM, Gaber T, Hahne M, Naumann L, Tripmacher R, Schellmann S, Stahn C, Burmester G-R, Radbruch A & Buttgereit F (2010) Rimexolone inhibits proliferation, cytokine expression and signal transduction of human CD4+ T-cells. *Immunol Lett* **131:** 24–32

Strobl JS, Kirkwood KL, Lantz TK, Lewine MA, Peterson VA & Worley JF (1990) Inhibition of human breast cancer cell proliferation in tissue culture by the neuroleptic agents pimozide and thioridazine. *Cancer Res* **50:** 5399–405

Szegezdi E, Logue SE, Gorman AM & Samali A (2006) Mediators of endoplasmic reticulum stress-induced apoptosis. *EMBO Rep* **7:** 880–5

Taldone T, Gozman A, Maharaj R & Chiosis G (2008) Targeting Hsp90: small-molecule inhibitors and their clinical development. *Curr Opin Pharmacol* **8:** 370–4

Temmink OH, Comijn EM, Fukushima M & Peters GJ (2004) Intracellular thymidylate synthase inhibition by trifluorothymidine in FM3A cells. *Nucleosides Nucleotides Nucleic Acids* **23:** 1491–4

Thomas LP & Friedland LR (1998) The cost-effective use of nebulized racemic epinephrine in the used to treat croup. *Am J Emerg Med* **16:** 87–9

Thorne CA, Hanson AJ, Schneider J, Tahinci E, Orton D, Cselenyi CS, Jernigan KK, Meyers KC, Hang BI, Waterson AG, Kim K, Melancon B, Ghidu VP, Sulikowski GA, LaFleur B, Salic A, Lee LA, Miller DM & Lee E (2010) Small-molecule inhibition of Wnt signaling through activation of casein kinase 1α. *Nat Chem Biol* **6:** 829–36

Vanhaecke T, Papeleu P, Elaut G & Rogiers V (2004) Trichostatin A-like hydroxamate histone deacetylase inhibitors as therapeutic agents: toxicological point of view. *Curr Med Chem* **11:** 1629–43

Wagner CR, Ballato G, Akanni AO, McIntee EJ, Larson RS, Chang S & Abul-Hajj YJ (1997) Potent growth inhibitory activity of zidovudine on cultured human breast cancer cells and rat mammary tumors. *Cancer Res* **57:** 2341–2345

Walker UA, Venhoff N, Koch EC, Olschewski M, Schneider J & Setzer B (2003) Uridine abrogates mitochondrial toxicity related to nucleoside analogue reverse transcriptase inhibitors in HepG2 cells. *Antivir Ther* **8:** 463–70

Wang Y, Zhao Q, Ma S, Yang F, Gong Y & Ke C (2007) Sirolimus inhibits human pancreatic carcinoma cell proliferation by a mechanism linked to the targeting of mTOR/HIF-1 alpha/VEGF signaling. *IUBMB Life* **59:** 717–21

Weiss MJ, Wong JR, Ha CS, Bleday R, Salem RR, Steele GD & Chen LB (1987) Dequalinium, a topical antimicrobial agent, displays anticarcinoma activity based on selective mitochondrial accumulation. *Proc Natl Acad Sci U S A* **84:** 5444–8

Weston BJ, Spackman VM & Dewdney JM (1986) Effect of beta-lactam antibiotics on a human myeloid cell line: investigation of potential in vivo correlates in the mouse. *Cell Biol Toxicol* **2:** 549–57

Whitfield ML, Sherlock G, Saldanha AJ, Murray JI, Ball CA, Alexander KE, Matese JC, Perou CM, Hurt MM, Brown PO & Botstein D (2002) Identification of genes periodically expressed in the human cell cycle and their expression in tumors. *Mol Biol Cell* **13:** 1977–2000

Wong LJ, Tsao GC, Bruce JI & Wong SS (1990) Inhibition of in vitro RNA synthesis by hycanthone, oxamniquine and praziquantel. *Experientia* **46:** 461–4

Yip KW, Mao X, Au PYB, Hedley DW, Chow S, Dalili S, Mocanu JD, Bastianutto C, Schimmer A & Liu F-F (2006) Benzethonium chloride: a novel anticancer agent identified by using a cell-based small-molecule screen. *Clin Cancer Res* **12:** 5557–69

Yoda A (1974) Association and dissociation rate constants of the complexes between various cardiac monoglycosides and Na, K-ATPase. *Ann N Y Acad Sci* **242:** 598–616

Yuksel M, Okajima K, Uchiba M & Okabe H (2003) Gabexate mesilate, a synthetic protease inhibitor, inhibits lipopolysaccharide-induced tumor necrosis factor-

alpha production by inhibiting activation of both nuclear factor-kappaB and activator protein-1 in human monocytes. *J Pharmacol Exp Ther* **305:** 298–305

Zhai S, Yang L, Cui QC, Sun Y, Dou QP & Yan B (2010) Tumor cellular proteasome inhibition and growth suppression by 8-hydroxyquinoline and clioquinol requires their capabilities to bind copper and transport copper into cells. *J Biol Inorg Chem* **15:** 259–69

Zhou H, Shen T, Luo Y, Liu L, Chen W, Xu B, Han X, Pang J, Rivera CA & Huang S (2010) The antitumor activity of the fungicide ciclopirox. *Int J Cancer* **127:** 2467–77

Zhou J, Duan L, Chen H, Ren X, Zhang Z, Zhou F, Liu J, Pei D & Ding K (2009) Atovaquone derivatives as potent cytotoxic and apoptosis inducing agents. *Bioorg Med Chem Lett* **19:** 5091–4

Zold E, Szodoray P, Nakken B, Barath S, Kappelmayer J, Csathy L, Hajas A, Sipka S, Gyimesi E, Gaal J, Barta Z, Hallay J, Szegedi G & Bodolay E (2011) Alfacalcidol treatment restores derailed immune-regulation in patients with undifferentiated connective tissue disease. *Autoimmun Rev* **10:** 155–62
