# Supplementary material for: Characterization of drug-induced transcriptional modules: towards drug repositioning and functional understanding
Source: Mol Syst Biol. 2013 Apr 30;9:662. doi: 10.1038/msb.2013.20 (PMC3658274; doi:10.1038/msb.2013.20)
Supplement: Supplementary Data set 1 — Characterization of gene and drug members of drug-induced modules [file msb201320-s3.zip › Supplementary_Dataset_1/CODIM/heatmaps/CODI-module14.pdf]

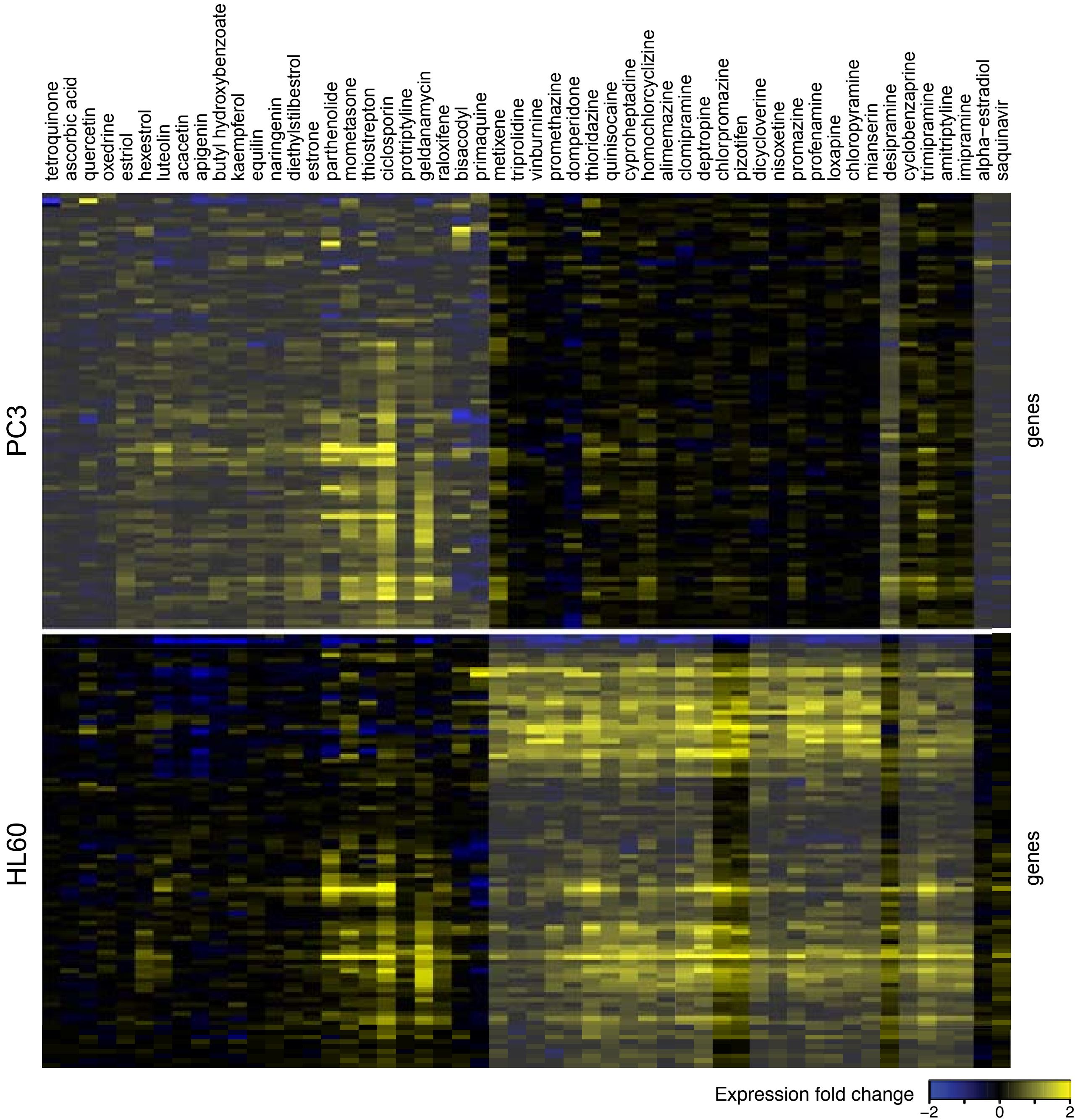
tetroquinone
ascorbic acid
quercetin
oxedrine
estriol
hexestrol
luteolin
acacetin
apigenin
butyl hydroxybenzoate
kaempferol
equilin
naringenin
diethylstilbestrol
estrone
parthenolide
mometasone
thiostrepton
ciclosporin
protriptyline
geldanamycin
raloxifene
bisacodyl
primaquine
metixene
triprolidine
vinburnine
promethazine
domperidone
thioridazine
quinisocaine
cyproheptadine
homochlorcyclizine
alimemazine
clomipramine
deptropine
chlorpromazine
pizotifen
dicycloverine
nisoxetine
promazine
profenamine
loxapine
chloropyramine
mianserin
desipramine
cyclobenzaprine
trimipramine
amitriptyline
imipramine
alpha−estradiol
saquinavir
PC3
genes
HL60
genes
Expression fold change
−2
0
2
