# Supplementary material for: Characterization of drug-induced transcriptional modules: towards drug repositioning and functional understanding
Source: Mol Syst Biol. 2013 Apr 30;9:662. doi: 10.1038/msb.2013.20 (PMC3658274; doi:10.1038/msb.2013.20)
Supplement: Supplementary Data set 1 — Characterization of gene and drug members of drug-induced modules [file msb201320-s3.zip › Supplementary_Dataset_1/CODIM/heatmaps/CODI-module15.pdf]

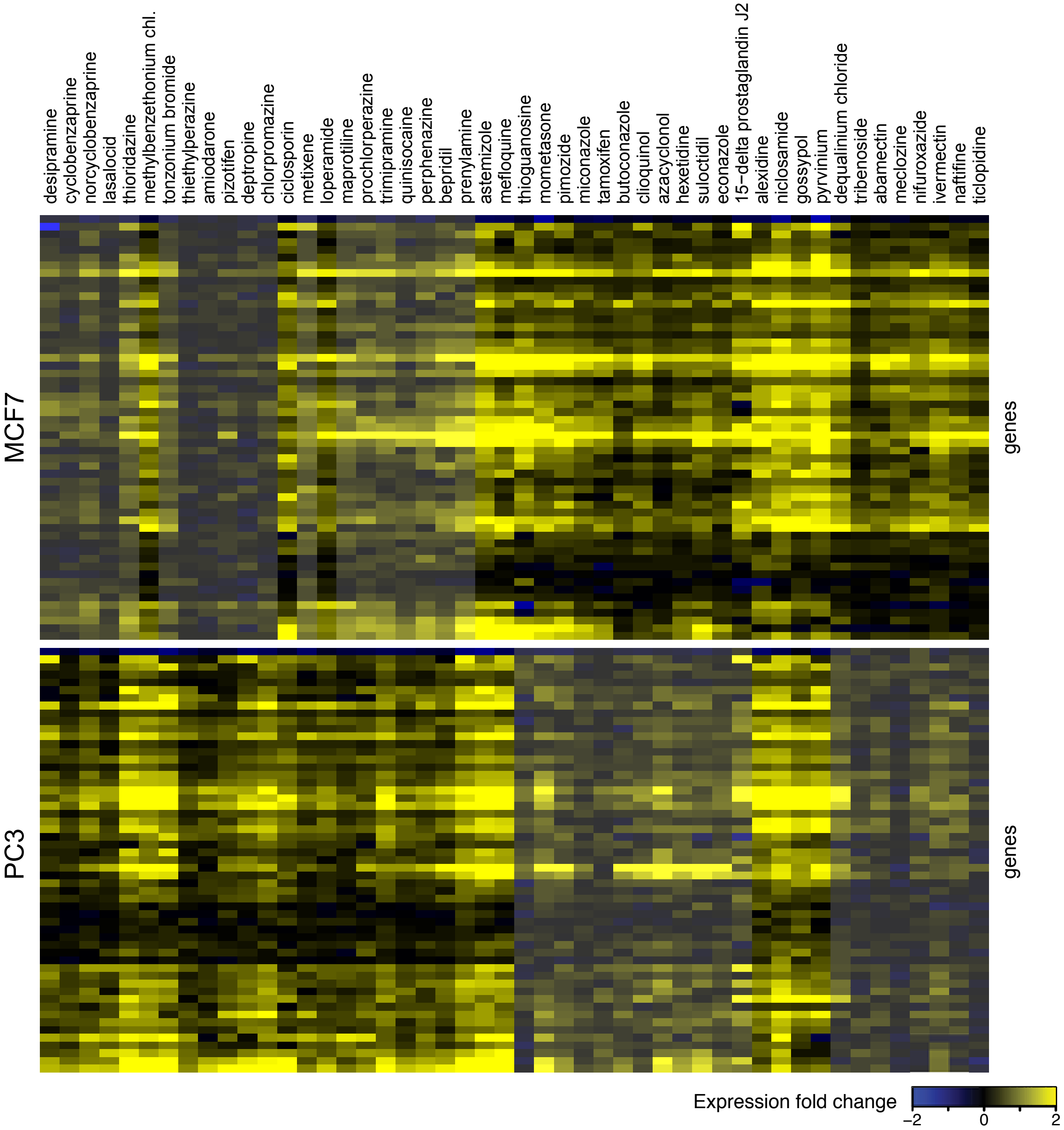

desipramine
cyclobenzaprine
norcyclobenzaprine
lasalocid
thioridazine
methylbenzethonium chl.
tonzonium bromide
thiethylperazine
amiodarone
pizotifen
deptropine
chlorpromazine
ciclosporin
metixene
loperamide
maprotiline
prochlorperazine
trimipramine
quinisocaine
perphenazine
bepridil
prenylamine
astemizole
mefloquine
thioguanosine
mometasone
pimozide
miconazole
tamoxifen
butoconazole
clioquinol
azacyclonol
hexetidine
suloctidil
econazole
15−delta prostaglandin J2
alexidine
niclosamide
gossypol
pyrvinium
dequalinium chloride
tribenoside
abamectin
meclozine
nifuroxazide
ivermectin
naftifine
ticlopidine
MCF7
genes
PC3
genes
Expression fold change
−2
0
2
