# Supplementary material for: Characterization of drug-induced transcriptional modules: towards drug repositioning and functional understanding
Source: Mol Syst Biol. 2013 Apr 30;9:662. doi: 10.1038/msb.2013.20 (PMC3658274; doi:10.1038/msb.2013.20)
Supplement: Supplementary Data set 1 — Characterization of gene and drug members of drug-induced modules [file msb201320-s3.zip › Supplementary_Dataset_1/CODIM/heatmaps/CODI-module17.pdf]

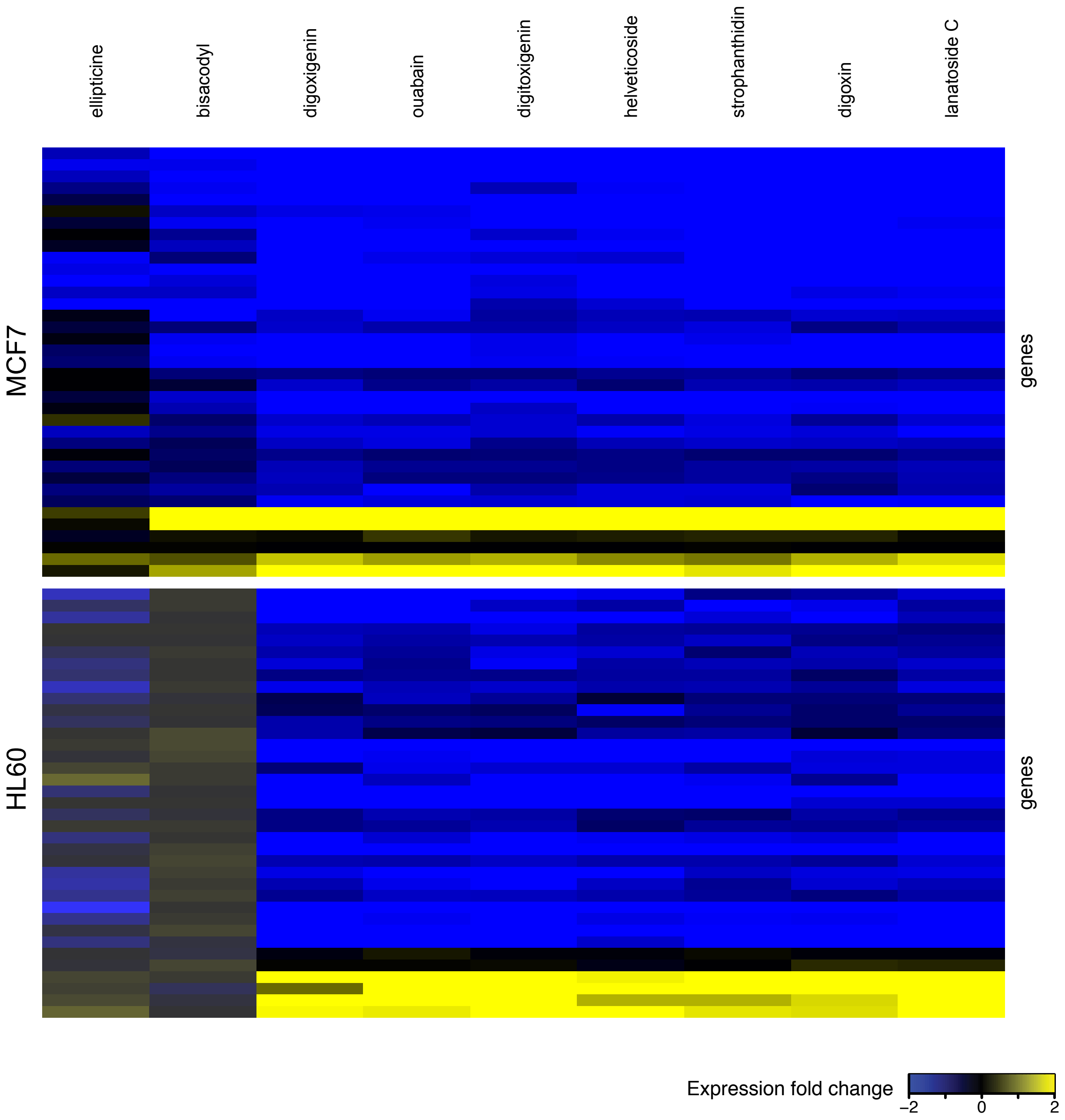
ellipticine
bisacodyl
digoxigenin
ouabain
digitoxigenin
helveticoside
strophanthidin
digoxin
lanatoside C
MCF7
HL60
genes
genes
Expression fold change
−2
0
2
