# Supplementary material for: Characterization of drug-induced transcriptional modules: towards drug repositioning and functional understanding
Source: Mol Syst Biol. 2013 Apr 30;9:662. doi: 10.1038/msb.2013.20 (PMC3658274; doi:10.1038/msb.2013.20)
Supplement: Supplementary Data set 1 — Characterization of gene and drug members of drug-induced modules [file msb201320-s3.zip › Supplementary_Dataset_1/CODIM/heatmaps/CODI-module2.pdf]

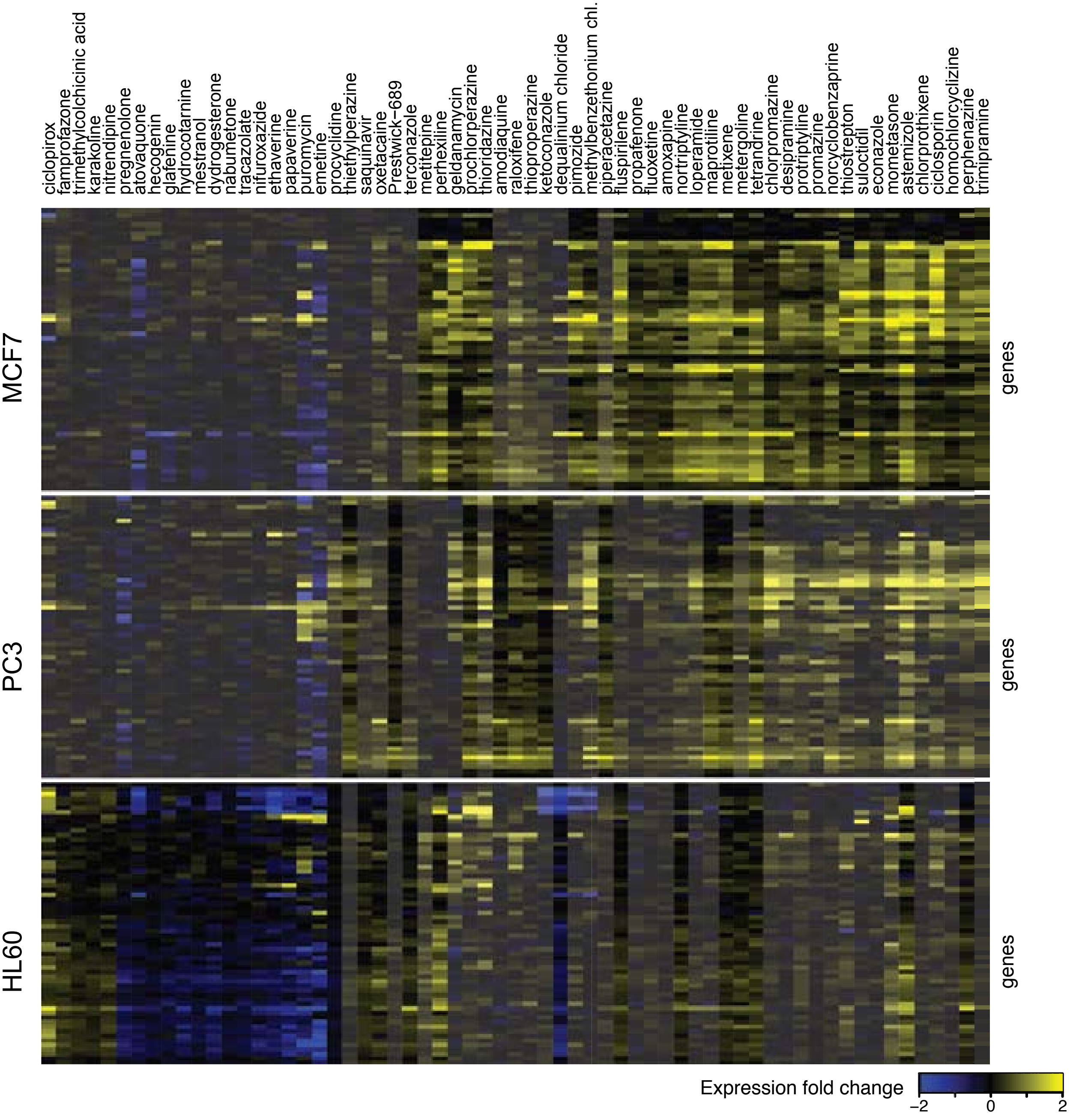
ciclopirox
famprofazone
trimethylcolchicinic acid
karakoline
nitrendipine
pregnenolone
atovaquone
hecogenin
glafenine
hydrocotarnine
mestranol
dydrogesterone
nabumetone
tracazolate
nifuroxazide
ethaverine
papaverine
puromycin
emetine
procyclidine
thiethylperazine
saquinavir
oxetacaine
Prestwick−689
terconazole
metitepine
perhexiline
geldanamycin
prochlorperazine
thioridazine
amodiaquine
raloxifene
thioproperazine
ketoconazole
dequalinium chloride
pimozide
methylbenzethonium chl.
piperacetazine
fluspirilene
propafenone
fluoxetine
amoxapine
nortriptyline
loperamide
maprotiline
metixene
metergoline
tetrandrine
chlorpromazine
desipramine
protriptyline
promazine
norcyclobenzaprine
thiostrepton
suloctidil
econazole
mometasone
astemizole
chlorprothixene
ciclosporin
homochlorcyclizine
perphenazine
trimipramine
MCF7
PC3
HL60
genes
genes
genes
Expression fold change
−2
0
2
