# Supplementary figures and images for: Characterization of drug-induced transcriptional modules: towards drug repositioning and functional understanding
Source: Mol Syst Biol. 2013 Apr 30;9:662. doi: 10.1038/msb.2013.20 (PMC3658274; doi:10.1038/msb.2013.20)

HL60

PC3

MCF7

Expression fold change

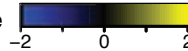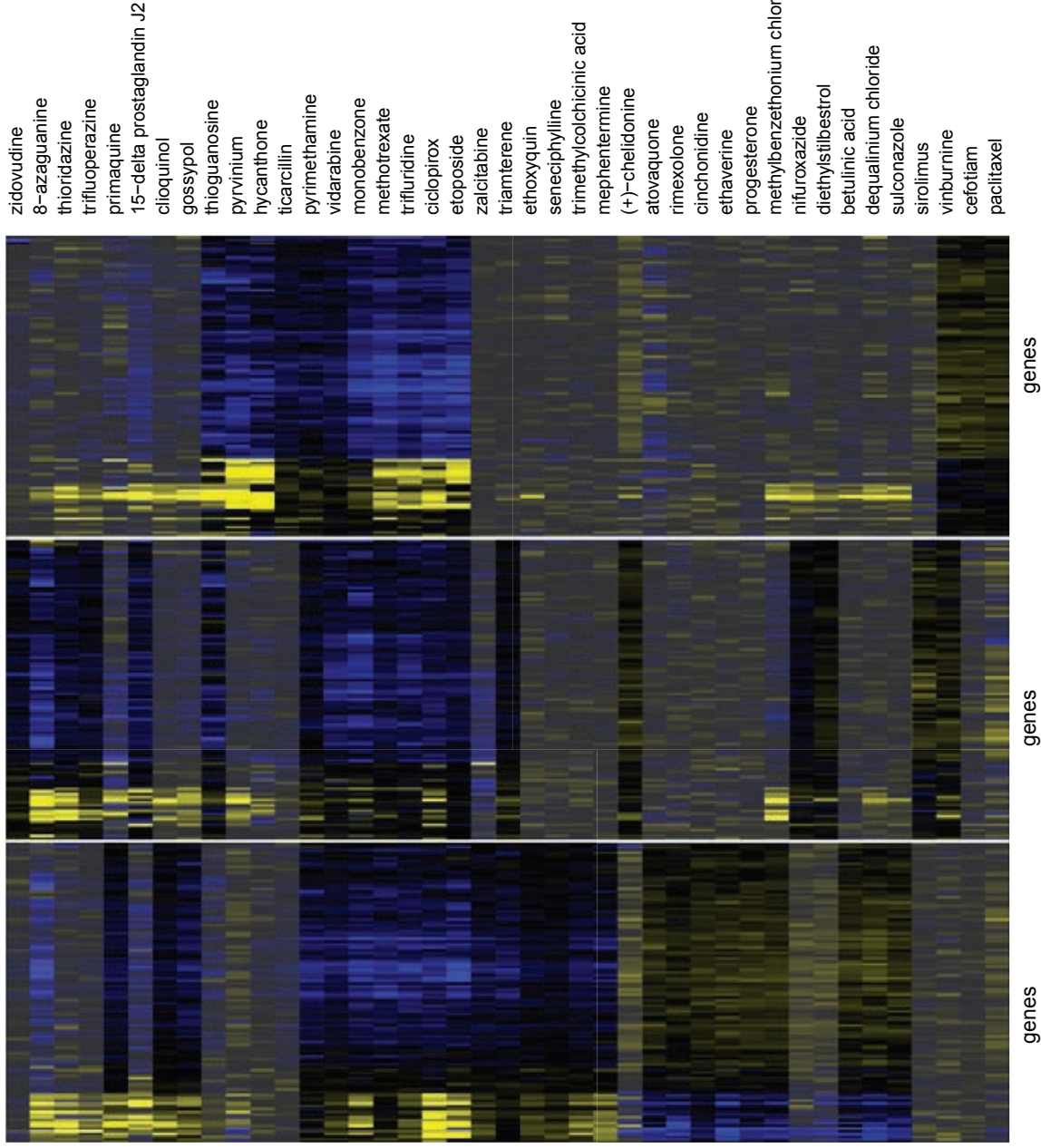

Supplement: Supplementary Data set 1 — Characterization of gene and drug members of drug-induced modules [file msb201320-s3.zip › Supplementary_Dataset_1/CODIM/heatmaps/CODI-module1.pdf]

HL60

PC3

MCF7

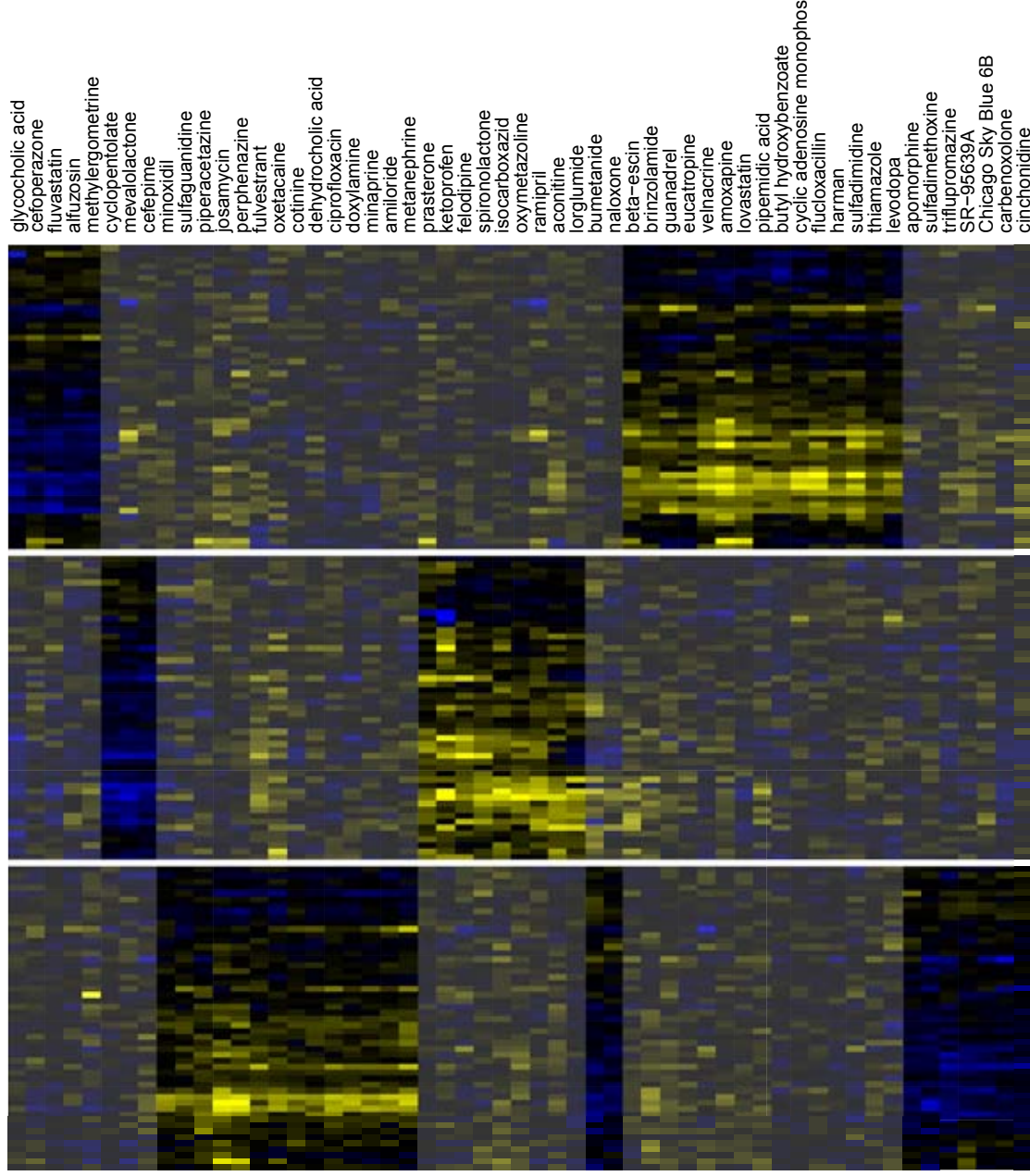

Expression fold change

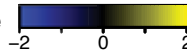

Supplement: Supplementary Data set 1 — Characterization of gene and drug members of drug-induced modules [file msb201320-s3.zip › Supplementary_Dataset_1/CODIM/heatmaps/CODI-module10.pdf]

HL60

PC3

MCF7

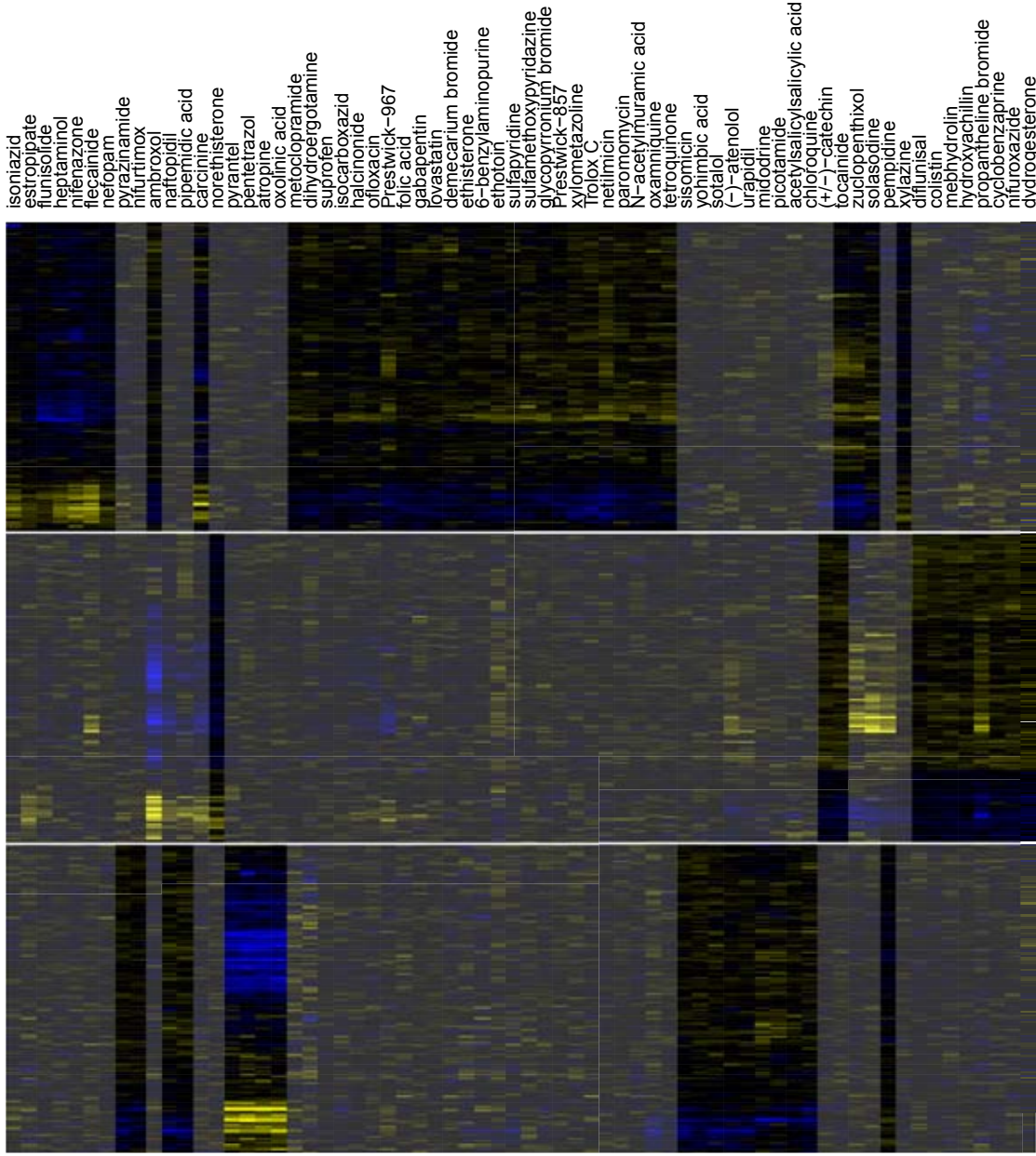

Expression fold change

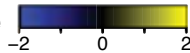

Supplement: Supplementary Data set 1 — Characterization of gene and drug members of drug-induced modules [file msb201320-s3.zip › Supplementary_Dataset_1/CODIM/heatmaps/CODI-module11.pdf]

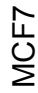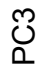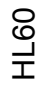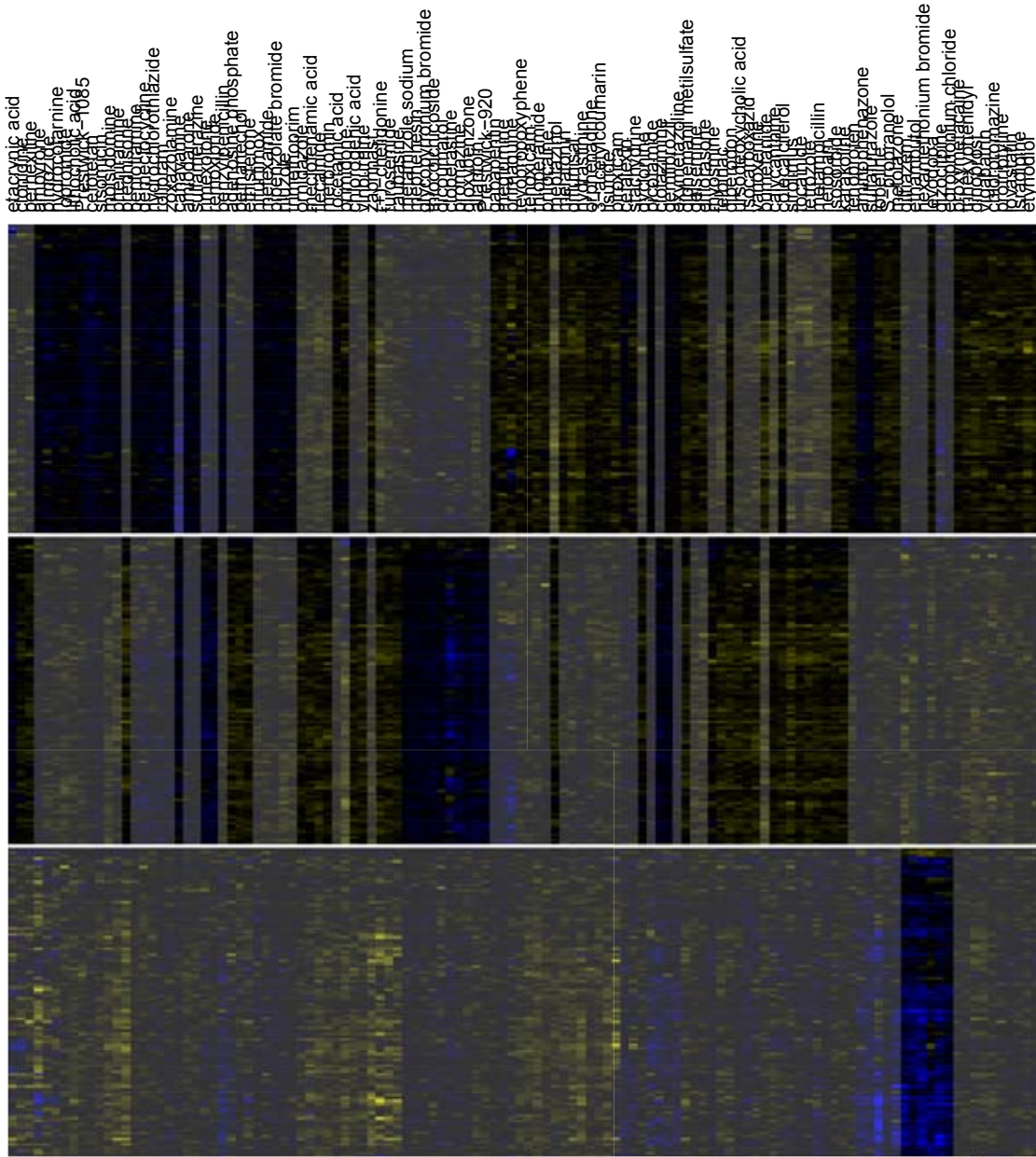

genes

genes

genes

Expression fold change

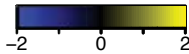

Supplement: Supplementary Data set 1 — Characterization of gene and drug members of drug-induced modules [file msb201320-s3.zip › Supplementary_Dataset_1/CODIM/heatmaps/CODI-module12.pdf]

HL60

MCF7

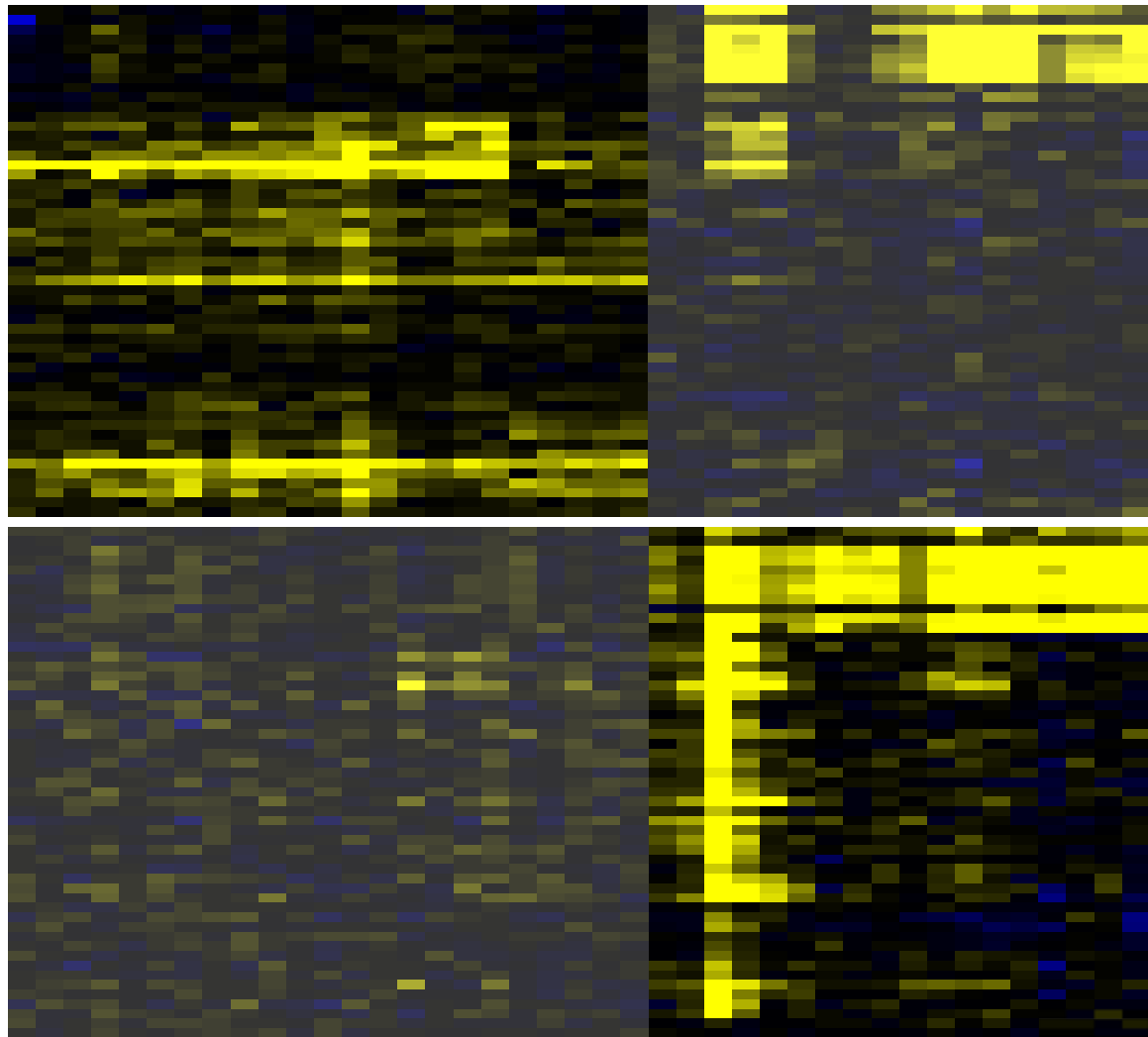

Expression fold change

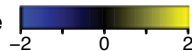

Supplement: Supplementary Data set 1 — Characterization of gene and drug members of drug-induced modules [file msb201320-s3.zip › Supplementary_Dataset_1/CODIM/heatmaps/CODI-module13.pdf]

PC3

MCF7

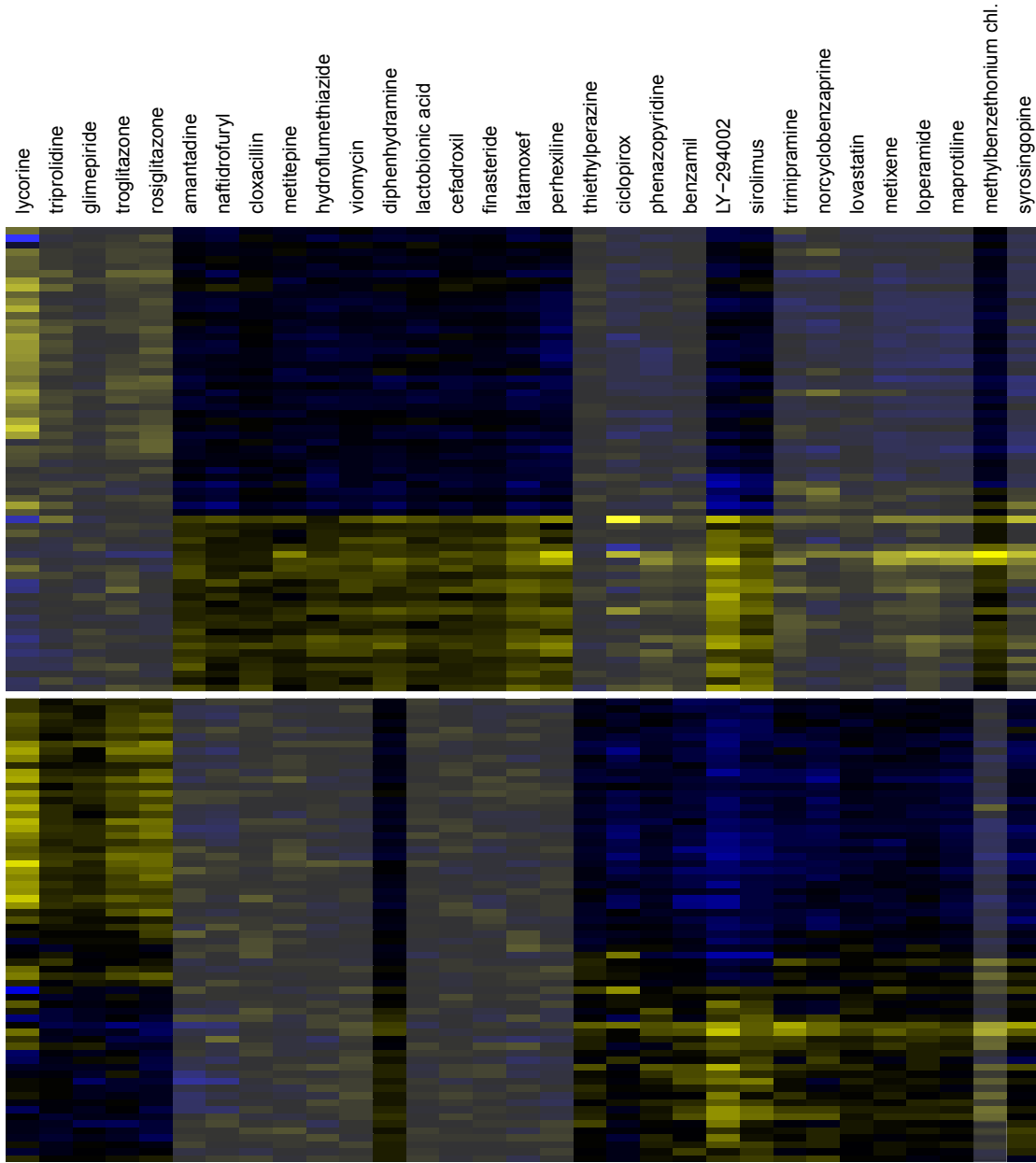

genes

genes

Expression fold change

-2

0

2

Supplement: Supplementary Data set 1 — Characterization of gene and drug members of drug-induced modules [file msb201320-s3.zip › Supplementary_Dataset_1/CODIM/heatmaps/CODI-module16.pdf]

PC3

MCF7

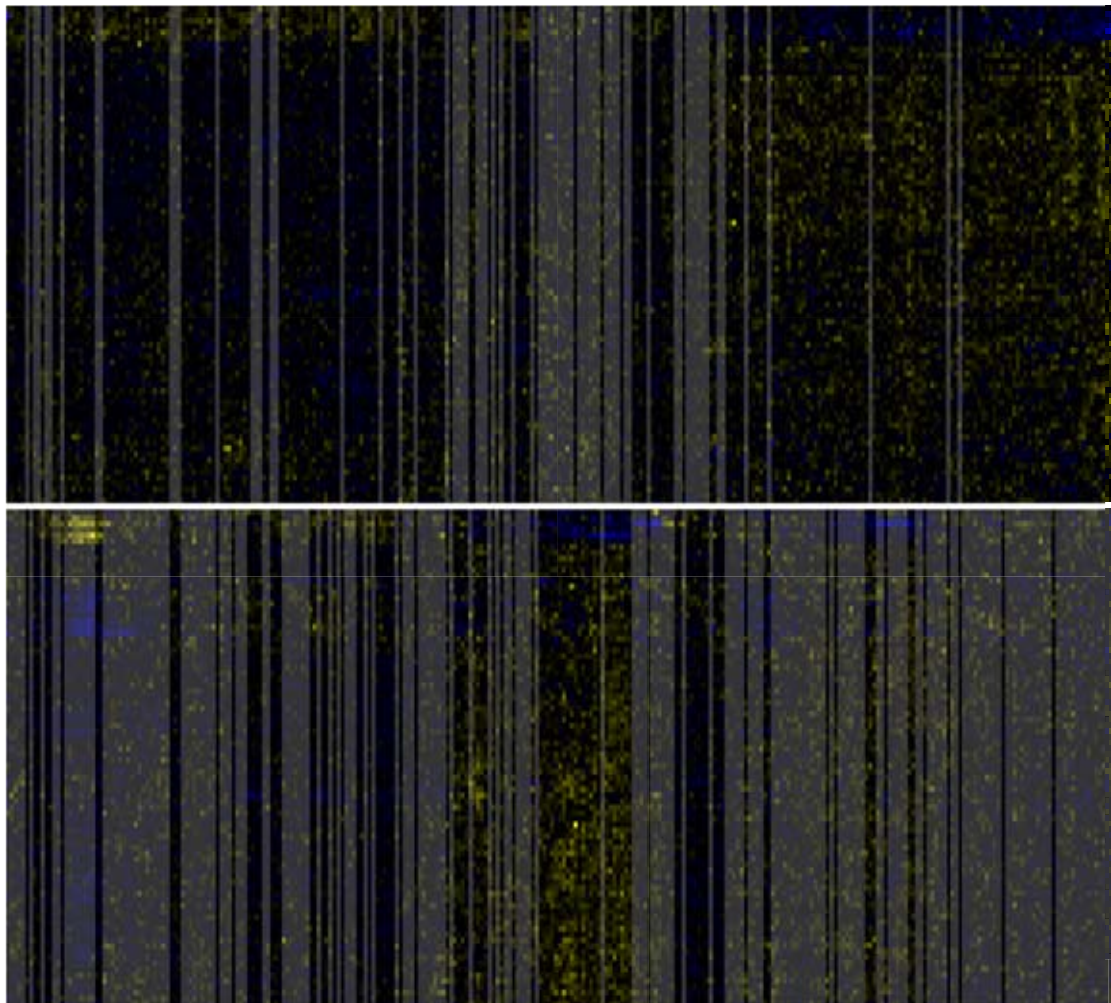

genes

genes

Expression fold change

Supplement: Supplementary Data set 1 — Characterization of gene and drug members of drug-induced modules [file msb201320-s3.zip › Supplementary_Dataset_1/CODIM/heatmaps/CODI-module18.pdf]

PC3

MCF7

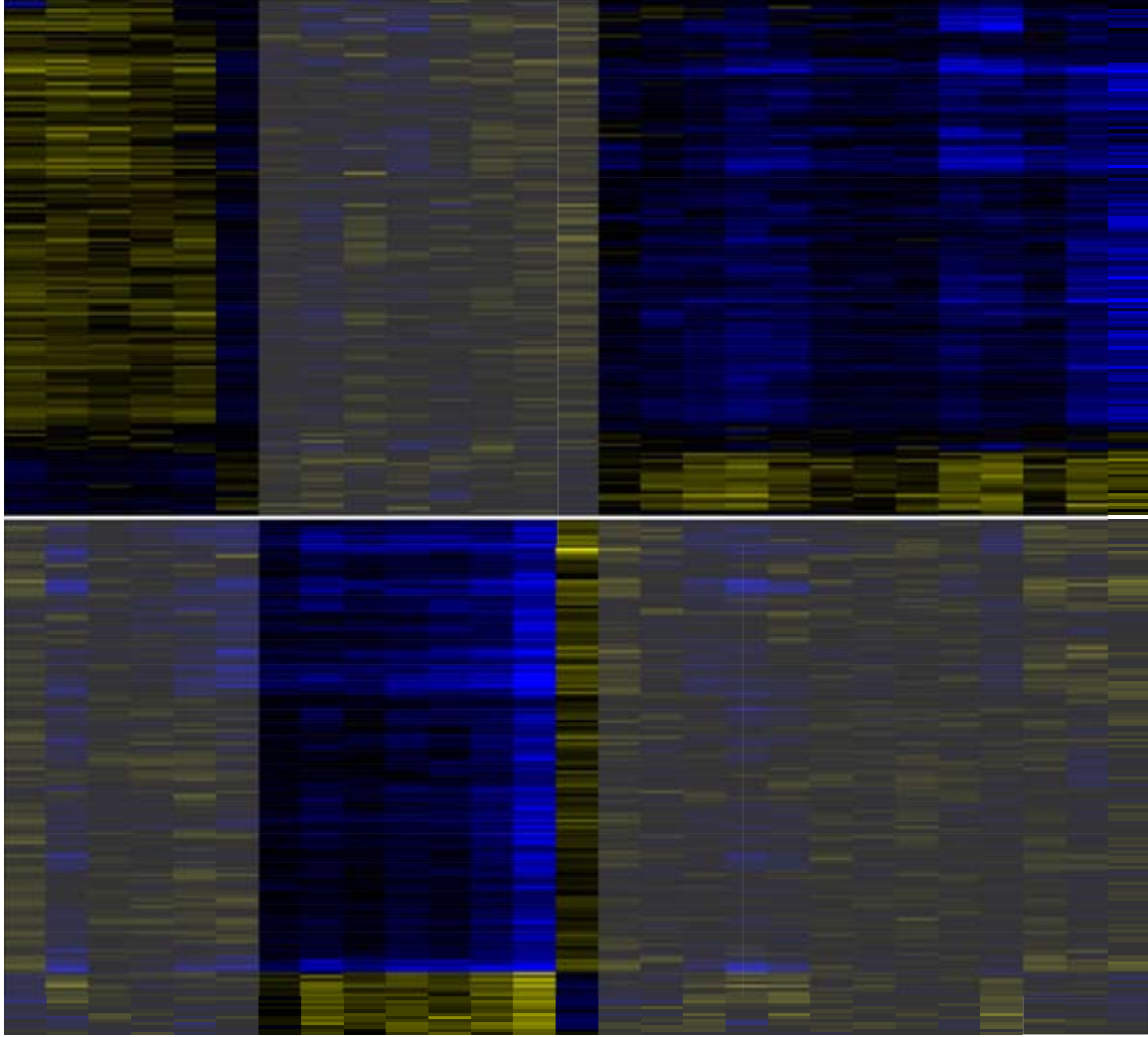

Expression fold change

-2 0 2

genes

genes

Supplement: Supplementary Data set 1 — Characterization of gene and drug members of drug-induced modules [file msb201320-s3.zip › Supplementary_Dataset_1/CODIM/heatmaps/CODI-module19.pdf]

MCF7

PC3

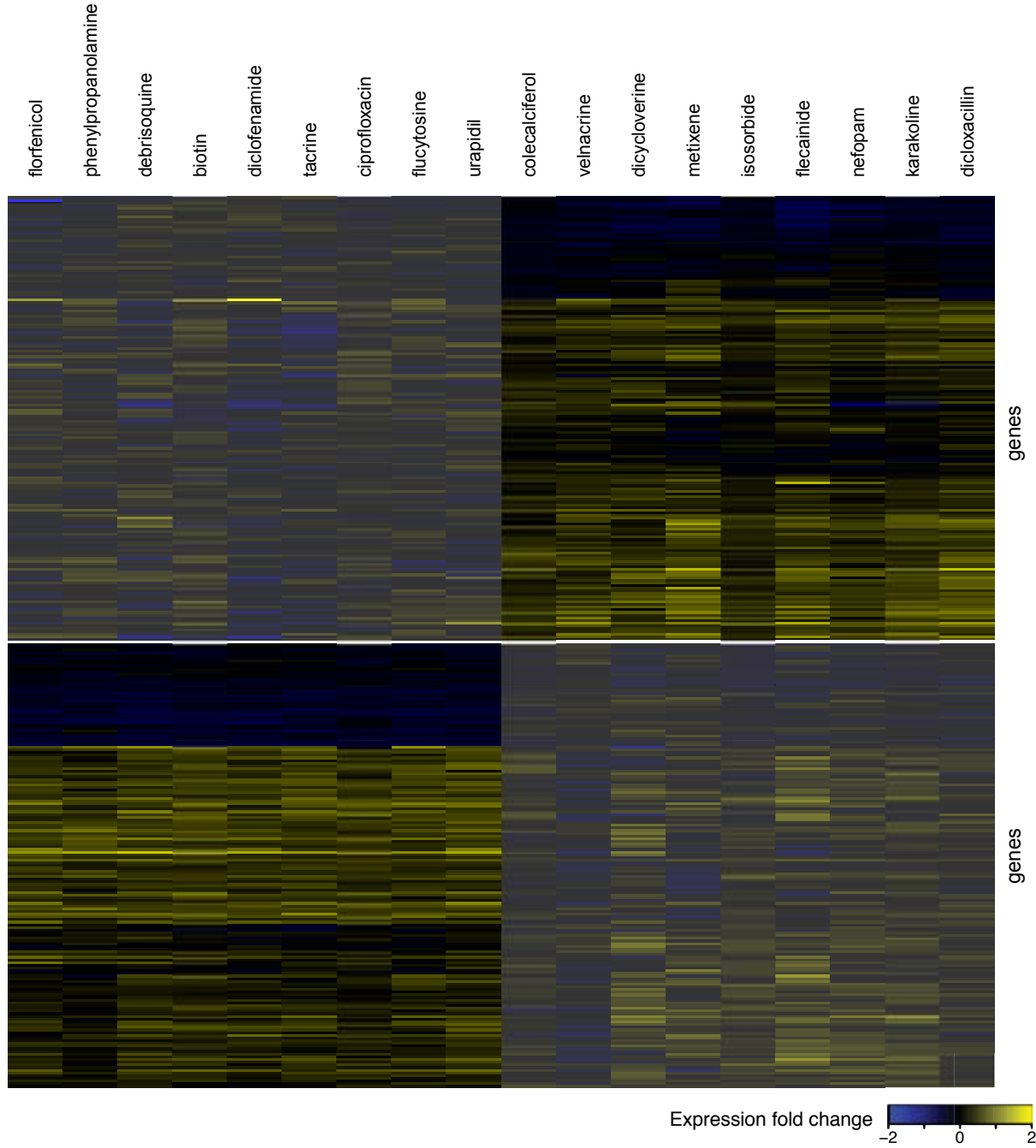

Supplement: Supplementary Data set 1 — Characterization of gene and drug members of drug-induced modules [file msb201320-s3.zip › Supplementary_Dataset_1/CODIM/heatmaps/CODI-module20.pdf]

PC3

MCF7

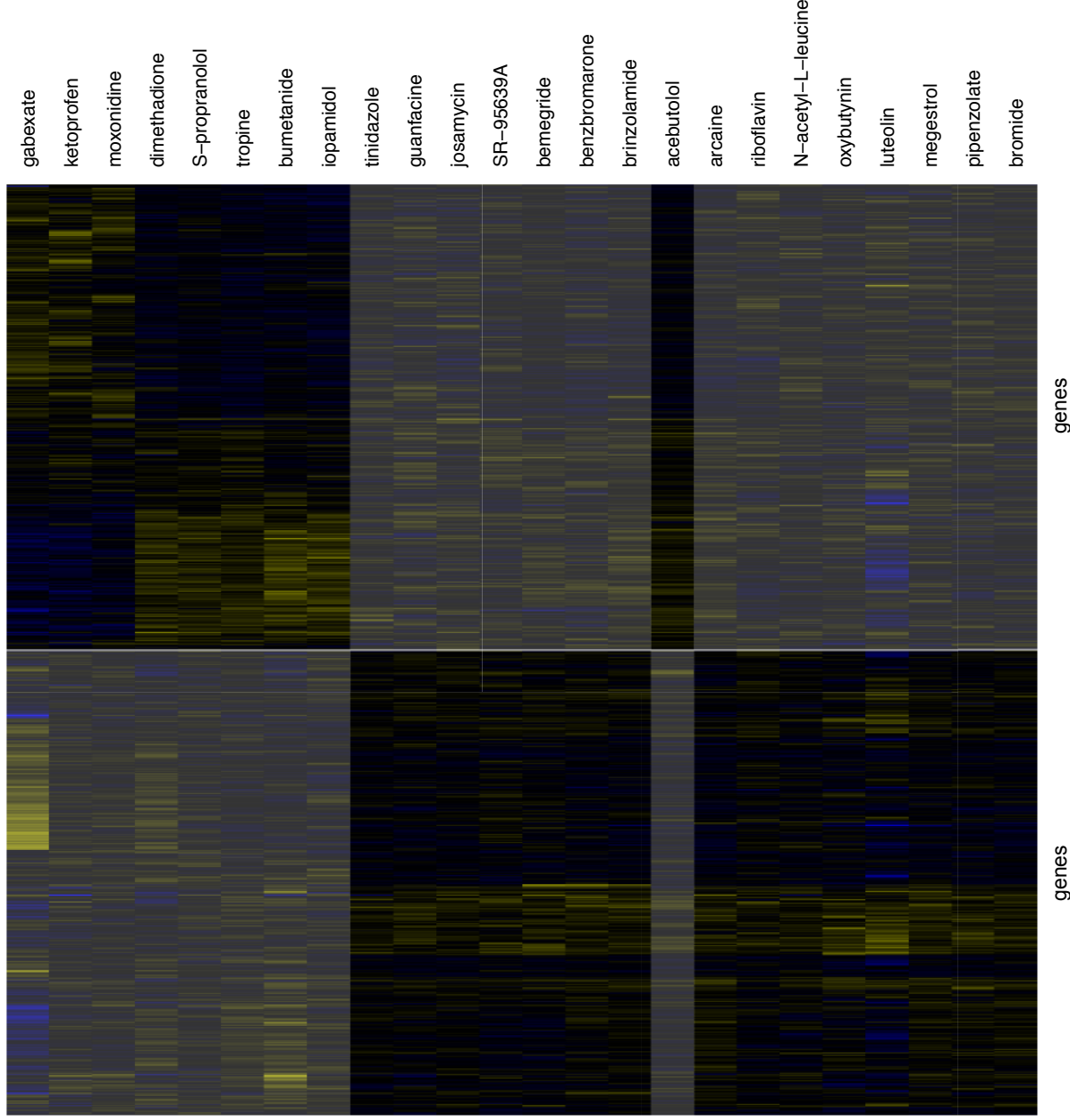

Supplement: Supplementary Data set 1 — Characterization of gene and drug members of drug-induced modules [file msb201320-s3.zip › Supplementary_Dataset_1/CODIM/heatmaps/CODI-module21.pdf]

MCF7

PC3

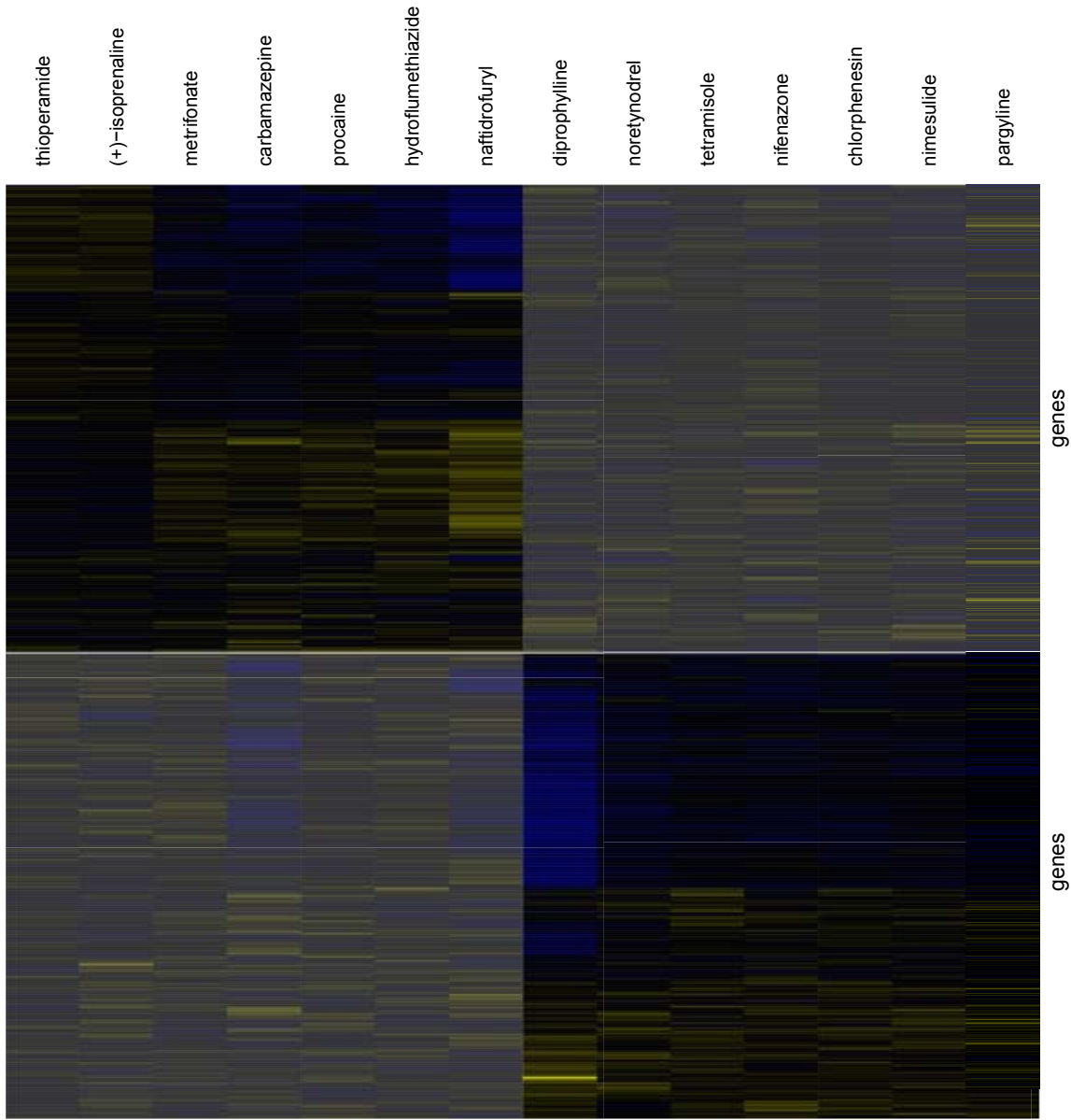

Expression fold change

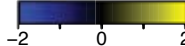

Supplement: Supplementary Data set 1 — Characterization of gene and drug members of drug-induced modules [file msb201320-s3.zip › Supplementary_Dataset_1/CODIM/heatmaps/CODI-module22.pdf]

HL60

PC3

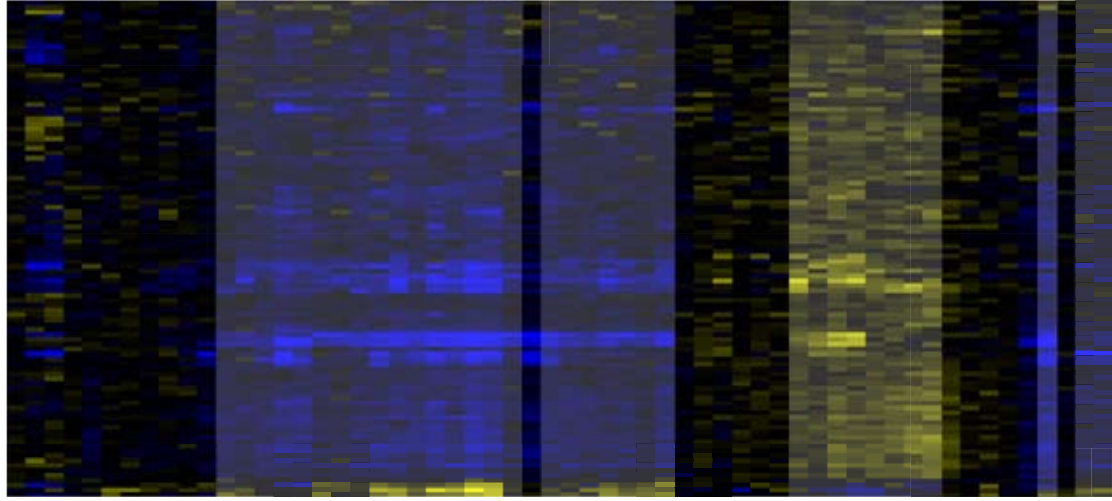

genes

genes

Expression fold change

-2 0 2

Supplement: Supplementary Data set 1 — Characterization of gene and drug members of drug-induced modules [file msb201320-s3.zip › Supplementary_Dataset_1/CODIM/heatmaps/CODI-module23.pdf]

HL60

PC3

MCF7

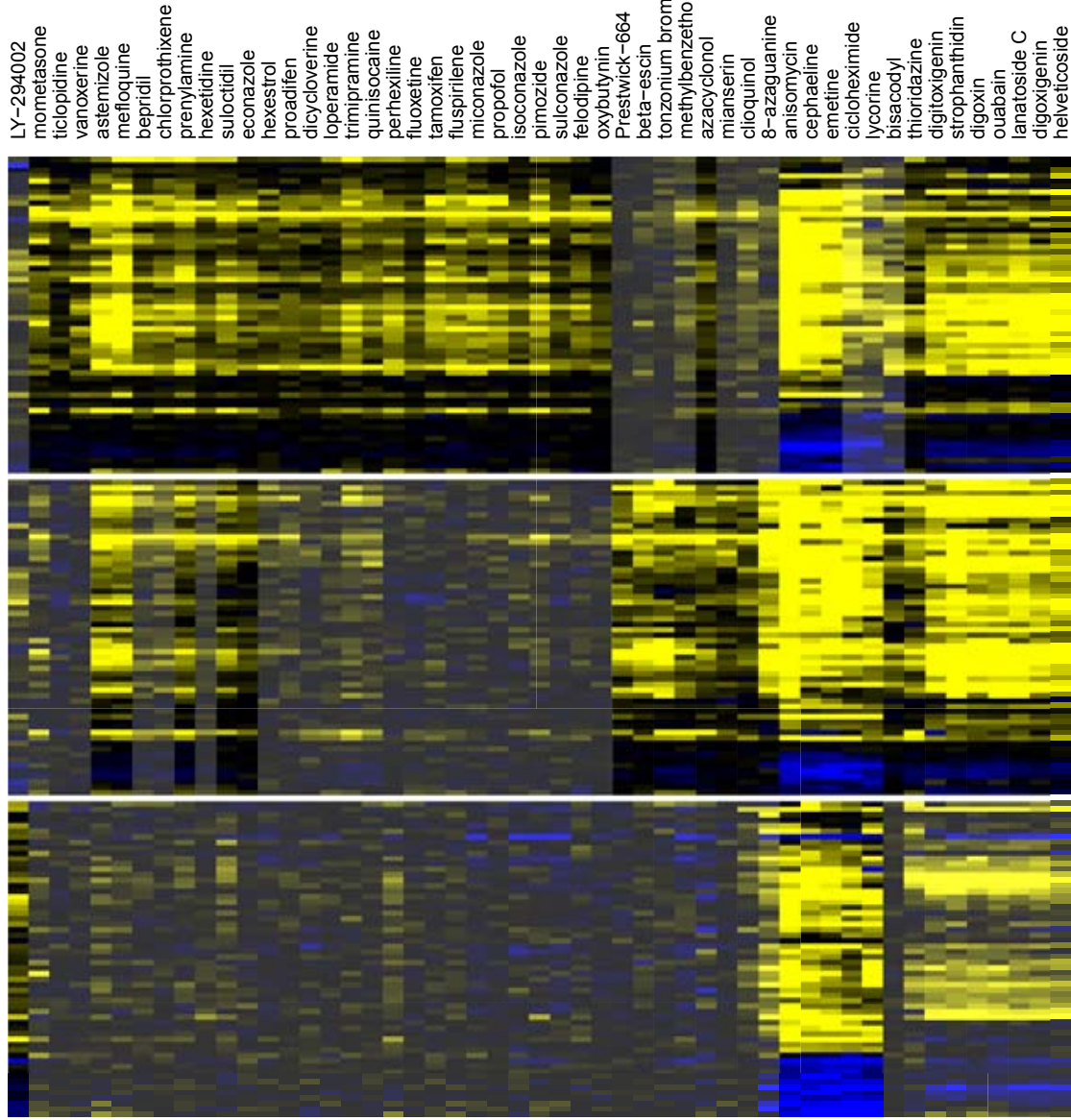

genes

genes

genes

Expression fold change

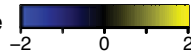

Supplement: Supplementary Data set 1 — Characterization of gene and drug members of drug-induced modules [file msb201320-s3.zip › Supplementary_Dataset_1/CODIM/heatmaps/CODI-module3.pdf]

HL60

PC3

MCF7

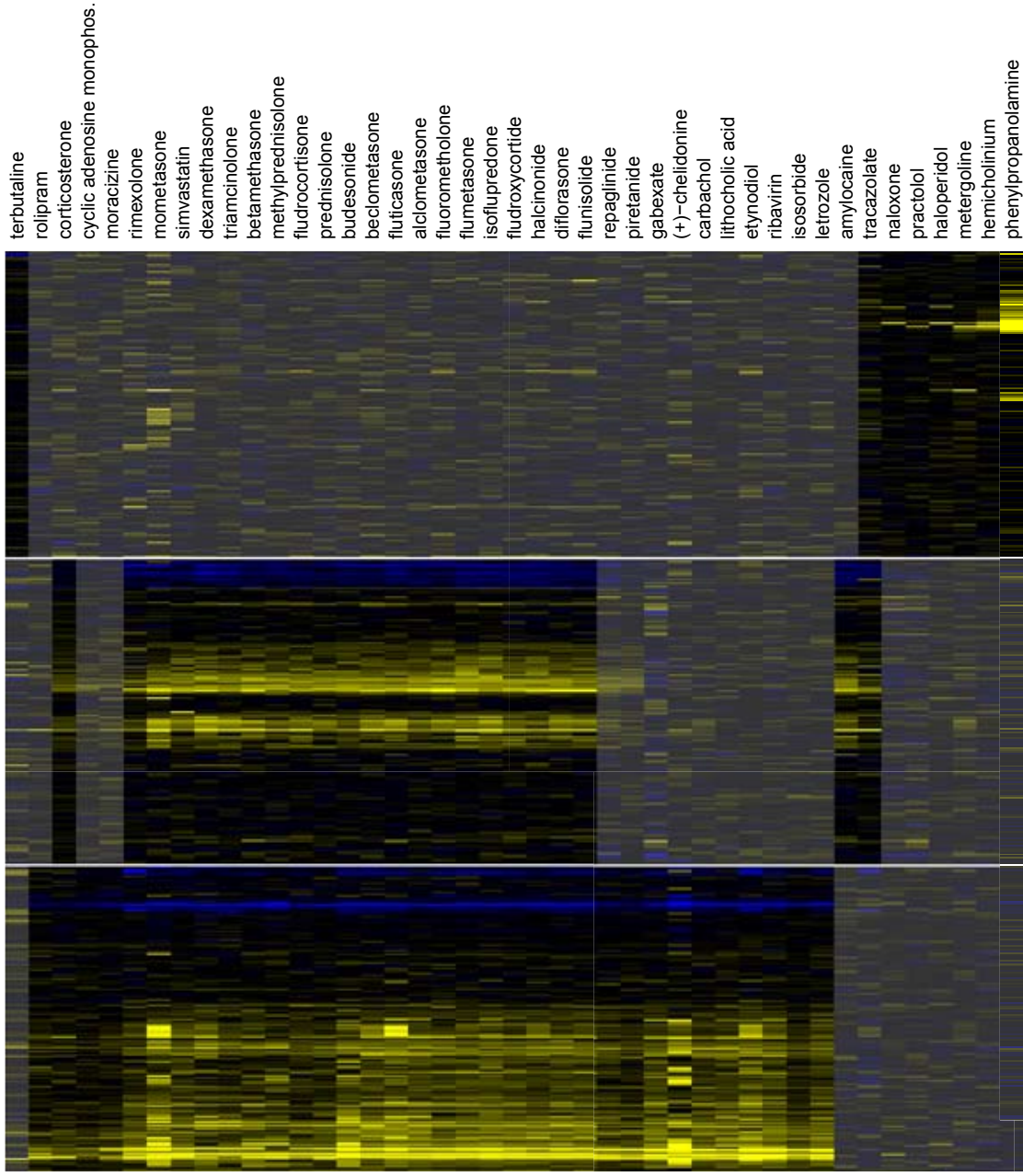

Expression fold change

-2

0

2

Supplement: Supplementary Data set 1 — Characterization of gene and drug members of drug-induced modules [file msb201320-s3.zip › Supplementary_Dataset_1/CODIM/heatmaps/CODI-module4.pdf]

HL60

PC3

MCF7

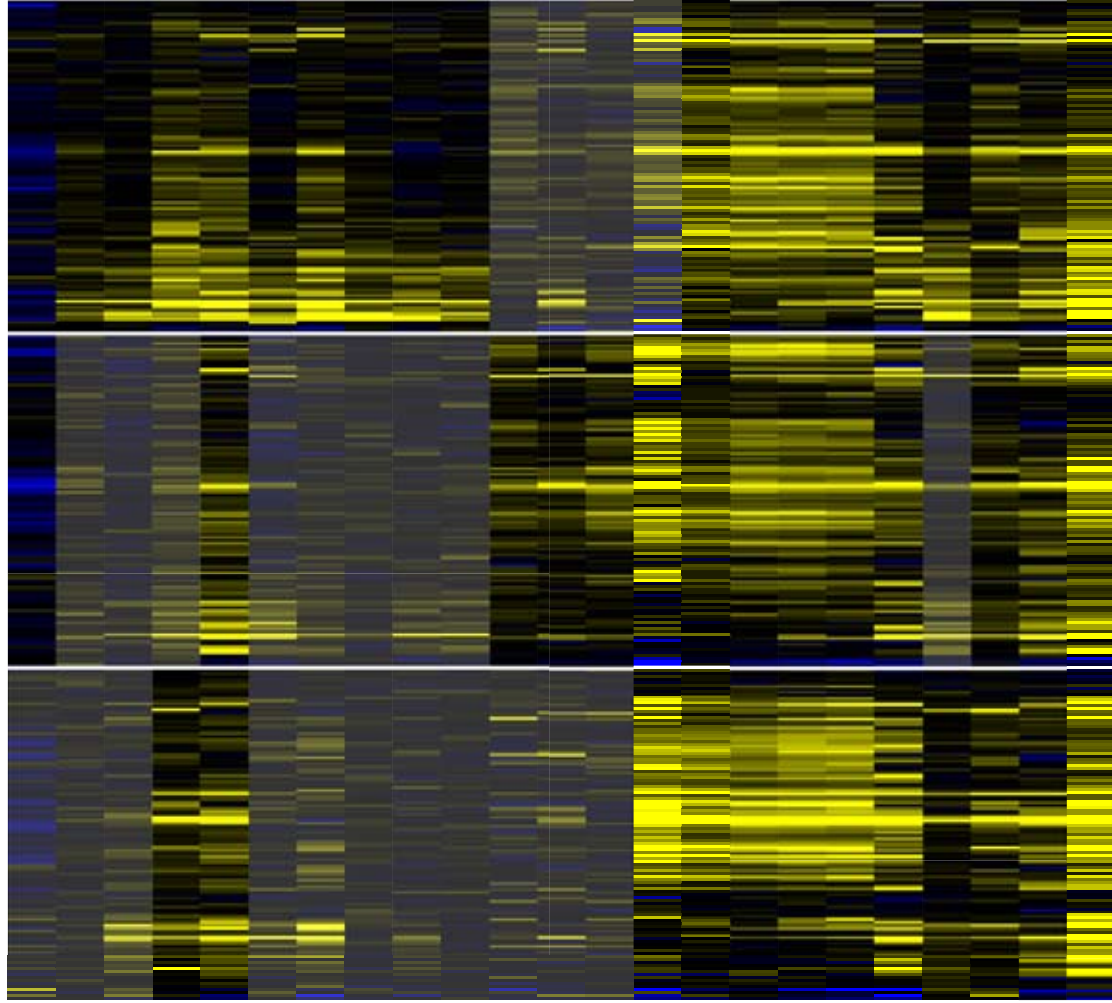

genes

genes

genes

Expression fold change

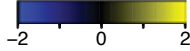

Supplement: Supplementary Data set 1 — Characterization of gene and drug members of drug-induced modules [file msb201320-s3.zip › Supplementary_Dataset_1/CODIM/heatmaps/CODI-module5.pdf]

HL60

PC3

MCF7

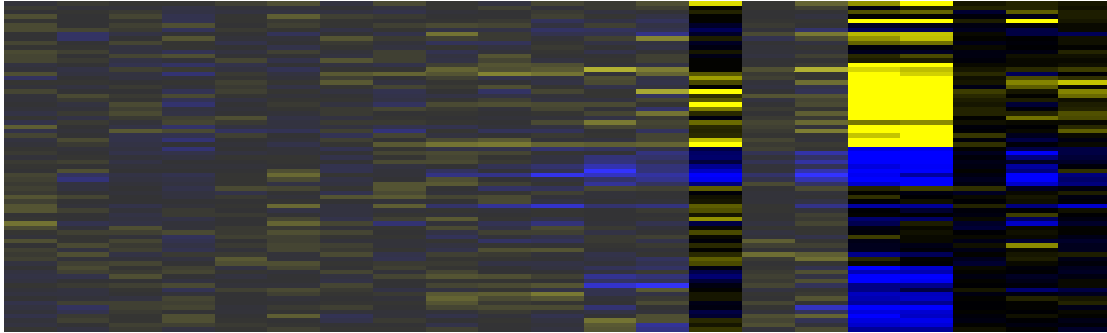

genes

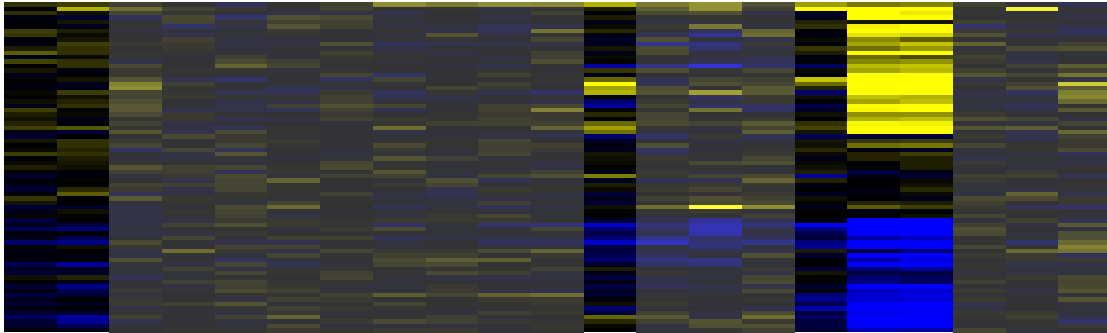

genes

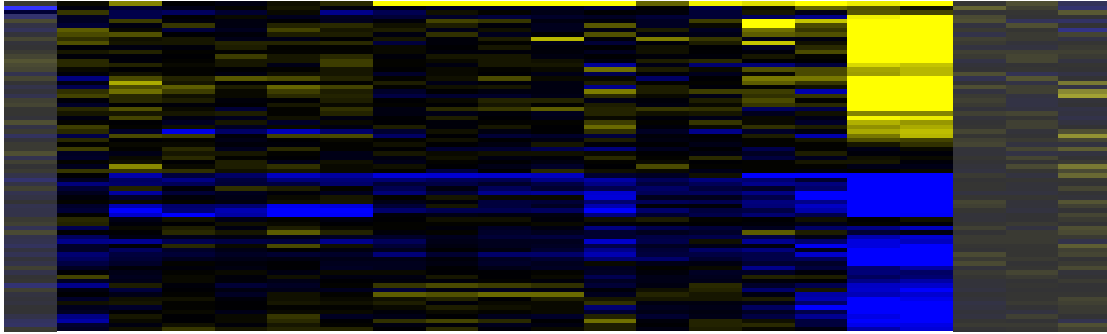

genes

Expression fold change

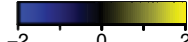

Supplement: Supplementary Data set 1 — Characterization of gene and drug members of drug-induced modules [file msb201320-s3.zip › Supplementary_Dataset_1/CODIM/heatmaps/CODI-module6.pdf]

MCF7

PC3

HL60

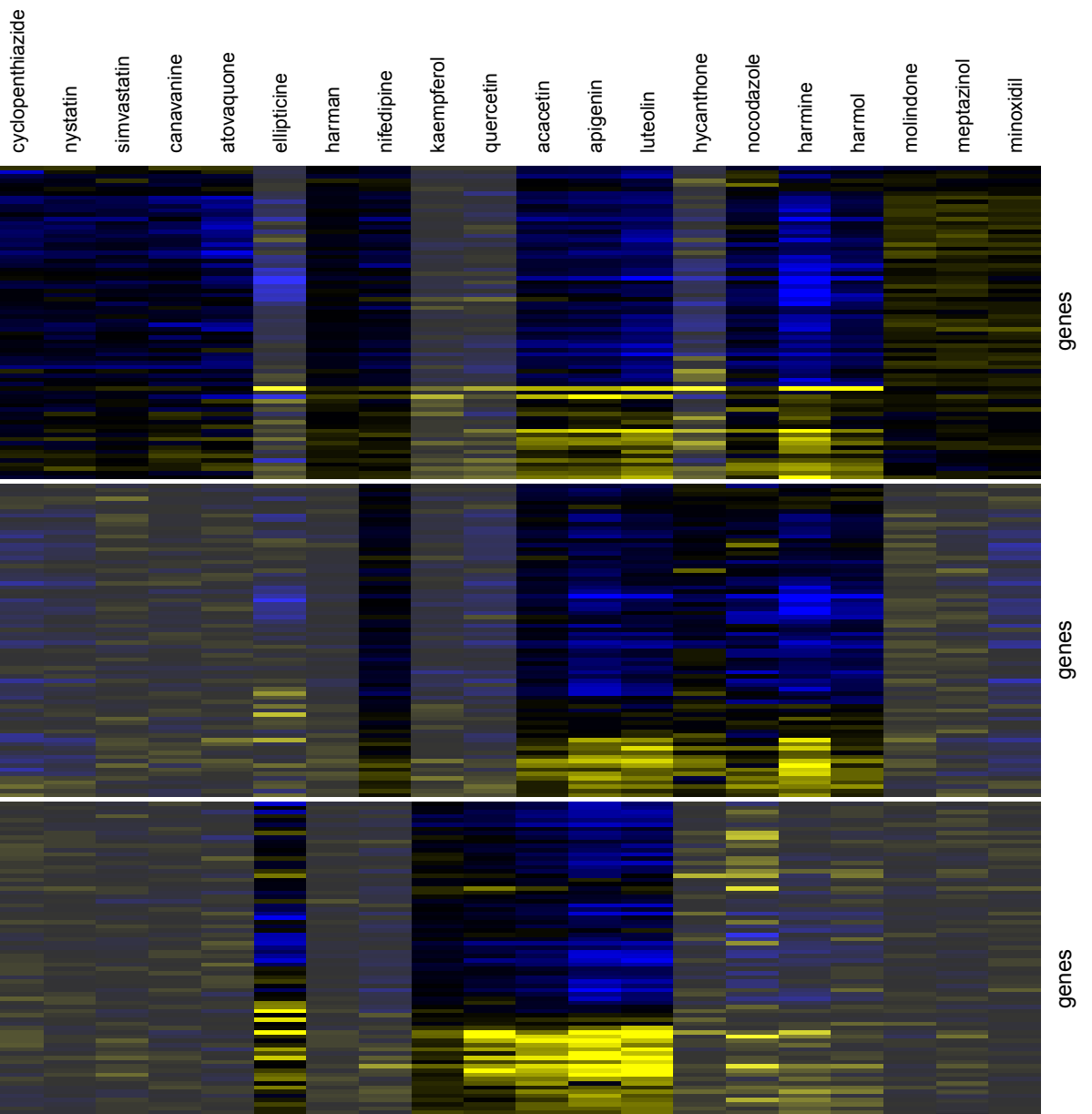

Supplement: Supplementary Data set 1 — Characterization of gene and drug members of drug-induced modules [file msb201320-s3.zip › Supplementary_Dataset_1/CODIM/heatmaps/CODI-module7.pdf]

HL60

PC3

MCF7

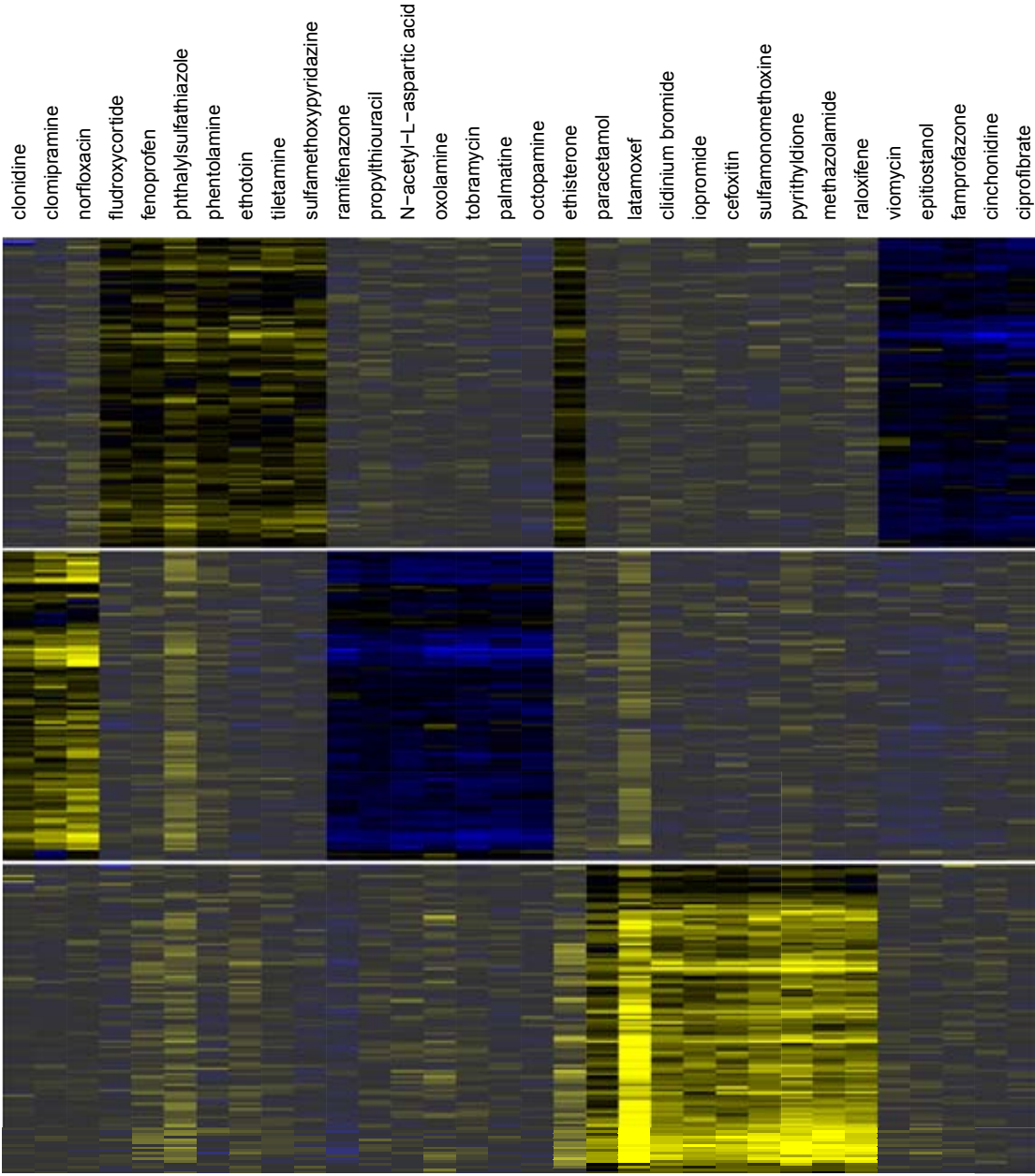

genes

genes

genes

Expression fold change  
-2 0 2

Supplement: Supplementary Data set 1 — Characterization of gene and drug members of drug-induced modules [file msb201320-s3.zip › Supplementary_Dataset_1/CODIM/heatmaps/CODI-module8.pdf]

HL60

PC3

MCF7

genes

genes

genes

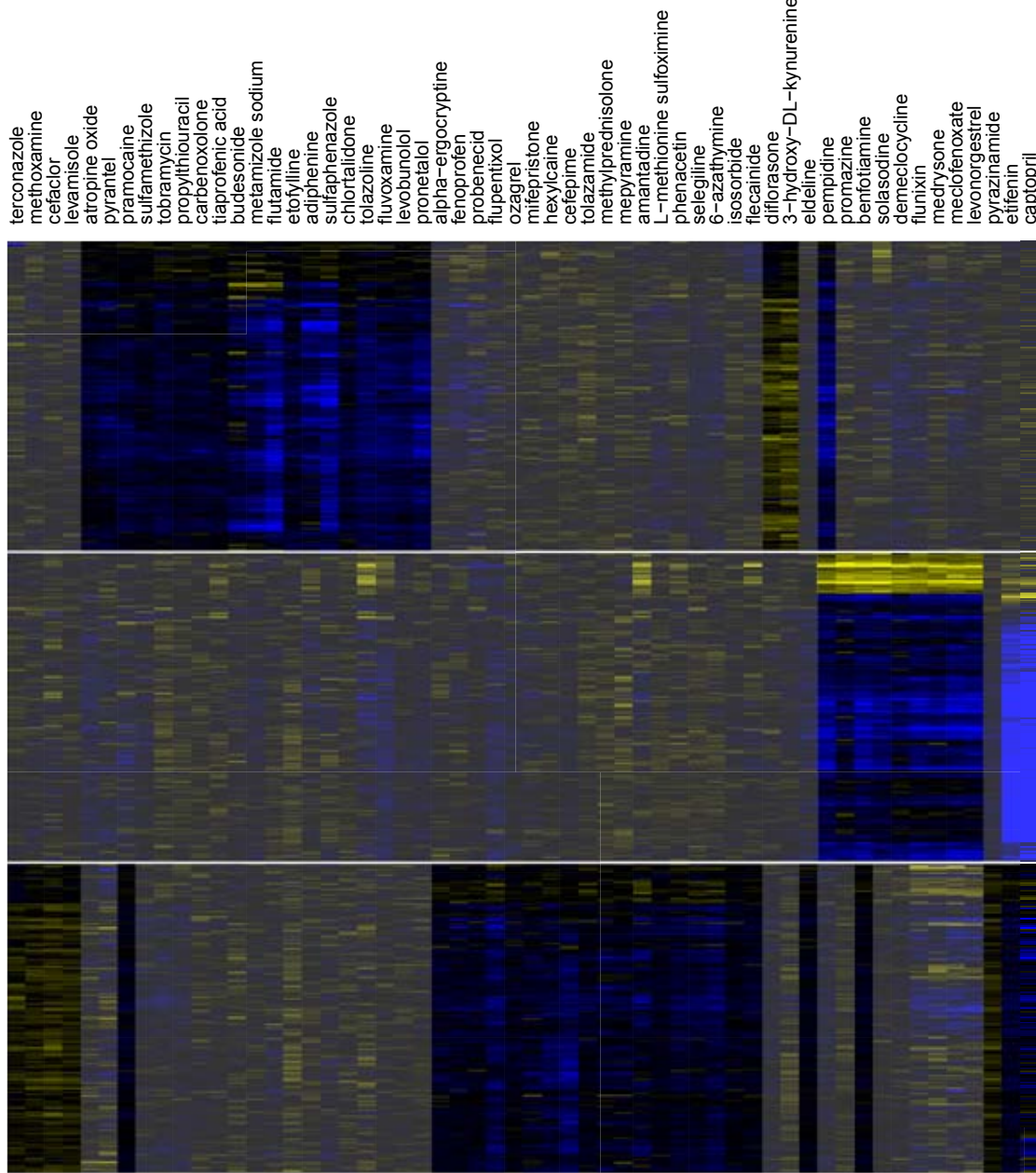

Supplement: Supplementary Data set 1 — Characterization of gene and drug members of drug-induced modules [file msb201320-s3.zip › Supplementary_Dataset_1/CODIM/heatmaps/CODI-module9.pdf]
