# Supplementary material for: Characterization of drug-induced transcriptional modules: towards drug repositioning and functional understanding
Source: Mol Syst Biol. 2013 Apr 30;9:662. doi: 10.1038/msb.2013.20 (PMC3658274; doi:10.1038/msb.2013.20)
Supplement: Supplementary Data set 1 — Characterization of gene and drug members of drug-induced modules [file msb201320-s3.zip › Supplementary_Dataset_1/Supplementary-dataset-1-all-modules-characterization.pdf]

# Supplementary Data Set 1. Characterization of drug-induced transcriptional modules.

## MCF7 Dataset:

### Drug-induced transcriptional module: MCF7-1

### 39 genes, 29 chemicals

### Functional enrichment of genes:

| **Annotation Cluster 1** | **Enrichment Score: 2.8282753257819455** | |
|---|---|---|
| | | |
| Category | Term | PValue |
| GOTERM_BP_FAT | GO:0006563~L-serine metabolic process | 4.29E-04 |
| GOTERM_BP_FAT | GO:0009069~serine family amino acid metabolic process | 2.70E-03 |
| KEGG_PATHWAY | hsa00260:Glycine, serine and threonine metabolism | 2.83E-03 |
| | | |
| **Annotation Cluster 2** | **Enrichment Score: 1.7516252603880789** | |
| | | |
| Category | Term | PValue |
| GOTERM_BP_FAT | GO:0006094~gluconeogenesis | 1.82E-03 |
| GOTERM_BP_FAT | GO:0019319~hexose biosynthetic process | 3.74E-03 |
| GOTERM_BP_FAT | GO:0046364~monosaccharide biosynthetic process | 5.82E-03 |
| GOTERM_BP_FAT | GO:0006090~pyruvate metabolic process | 7.27E-03 |
| GOTERM_BP_FAT | GO:0046165~alcohol biosynthetic process | 8.32E-03 |
| GOTERM_BP_FAT | GO:0034637~cellular carbohydrate biosynthetic process | 2.13E-02 |
| GOTERM_BP_FAT | GO:0016051~carbohydrate biosynthetic process | 4.55E-02 |
| GOTERM_BP_FAT | GO:0006006~glucose metabolic process | 7.75E-02 |
| GOTERM_BP_FAT | GO:0019318~hexose metabolic process | 1.16E-01 |
| GOTERM_BP_FAT | GO:0005996~monosaccharide metabolic process | 1.46E-01 |
| | | |
| | | |
| **Annotation Cluster 3** | **Enrichment Score: 1.608437532304507** | |
| | | |
| Category | Term | PValue |
| SP_PIR_KEYWORDS | Aminoacyl-tRNA synthetase | 2.34E-04 |
| GOTERM_BP_FAT | GO:0006418~tRNA aminoacylation for protein translation | 4.07E-04 |
| GOTERM_BP_FAT | GO:0043038~amino acid activation | 4.07E-04 |
| GOTERM_BP_FAT | GO:0043039~tRNA aminoacylation | 4.07E-04 |
| GOTERM_MF_FAT | GO:0016876~ligase activity, forming aminoacyl-tRNA and related compounds | 4.39E-04 |
| GOTERM_MF_FAT | GO:0016875~ligase activity, forming carbon-oxygen bonds | 4.39E-04 |
| GOTERM_MF_FAT | GO:0004812~aminoacyl-tRNA ligase activity | 4.39E-04 |
| KEGG_PATHWAY | hsa00970:Aminoacyl-tRNA biosynthesis | 4.50E-04 |
| GOTERM_BP_FAT | GO:0006399~tRNA metabolic process | 6.43E-03 |
| INTERPRO | IPR014729:Rossmann-like alpha/beta/alpha sandwich fold | 7.60E-03 |
| SP_PIR_KEYWORDS | ligase | 1.01E-02 |
| SP_PIR_KEYWORDS | protein biosynthesis | 2.97E-02 |
| GOTERM_BP_FAT | GO:0034660~ncRNA metabolic process | 4.23E-02 |
| GOTERM_BP_FAT | GO:0006412~translation | 9.52E-02 |
| GOTERM_MF_FAT | GO:0032553~ribonucleotide binding | 1.64E-01 |
| GOTERM_MF_FAT | GO:0032555~purine ribonucleotide binding | 1.64E-01 |
| SP_PIR_KEYWORDS | nucleotide-binding | 1.76E-01 |
| GOTERM_MF_FAT | GO:0017076~purine nucleotide binding | 1.94E-01 |
| GOTERM_MF_FAT | GO:0000166~nucleotide binding | 2.16E-01 |
| GOTERM_MF_FAT | GO:0005524~ATP binding | 2.87E-01 |
| GOTERM_MF_FAT | GO:0032559~adenyl ribonucleotide binding | 2.93E-01 |
| SP_PIR_KEYWORDS | atp-binding | 3.23E-01 |

| | | |
|---|---|---|
| GOTERM_MF_FAT | GO:0030554~adenyl nucleotide binding | 3.35E-01 |
| GOTERM_MF_FAT | GO:0001883~purine nucleoside binding | 3.46E-01 |
| GOTERM_MF_FAT | GO:0001882~nucleoside binding | 3.52E-01 |
| GOTERM_MF_FAT | GO:0003723~RNA binding | 4.27E-01 |
| GOTERM_CC_FAT | GO:0005829~cytosol | 4.63E-01 |
| SP_PIR_KEYWORDS | cytoplasm | 5.94E-01 |

## Enriched annotation of drugs:

| **Annotation type** | **Enrichment term** | **q-value** |
|---|---|---|
| ATC code (2nd level) | G01; Gynecological antiinfectives and antiseptics | 3.45E-02 |

## Drug-induced transcriptional module: MCF7-2

## 40 genes, 32 chemicals

## Functional enrichment of genes:

| **Annotation Cluster 1** | **Enrichment Score: 7.444863684200159** | |
|---|---|---|
| | | |
| Category | Term | PValue |
| GOTERM_BP_FAT | GO:0016126~sterol biosynthetic process | 3.76E-15 |
| SP_PIR_KEYWORDS | Steroid biosynthesis | 7.11E-15 |
| GOTERM_BP_FAT | GO:0006694~steroid biosynthetic process | 1.97E-14 |
| GOTERM_BP_FAT | GO:0008202~steroid metabolic process | 3.81E-14 |
| GOTERM_BP_FAT | GO:0016125~sterol metabolic process | 8.66E-14 |
| SP_PIR_KEYWORDS | lipid synthesis | 3.08E-13 |
| SP_PIR_KEYWORDS | sterol biosynthesis | 3.34E-12 |
| GOTERM_BP_FAT | GO:0008203~cholesterol metabolic process | 8.53E-11 |
| KEGG_PATHWAY | hsa00100:Steroid biosynthesis | 1.92E-10 |
| GOTERM_BP_FAT | GO:0006695~cholesterol biosynthetic process | 5.69E-10 |
| GOTERM_BP_FAT | GO:0008610~lipid biosynthetic process | 6.43E-10 |
| SP_PIR_KEYWORDS | Cholesterol biosynthesis | 1.14E-08 |
| GOTERM_BP_FAT | GO:0008299~isoprenoid biosynthetic process | 3.65E-07 |
| GOTERM_BP_FAT | GO:0006720~isoprenoid metabolic process | 5.25E-06 |
| KEGG_PATHWAY | hsa00900:Terpenoid backbone biosynthesis | 1.13E-04 |
| SP_PIR_KEYWORDS | Isoprene biosynthesis | 4.05E-04 |
| SP_PIR_KEYWORDS | oxidoreductase | 6.14E-04 |
| SP_PIR_KEYWORDS | nadp | 7.87E-04 |
| GOTERM_BP_FAT | GO:0055114~oxidation reduction | 1.52E-03 |
| GOTERM_CC_FAT | GO:0005777~peroxisome | 3.45E-02 |
| GOTERM_CC_FAT | GO:0042579~microbody | 3.45E-02 |
| SP_PIR_KEYWORDS | magnesium | 1.17E-01 |
| SP_PIR_KEYWORDS | transferase | 5.33E-01 |
| | | |
| | | |
| **Annotation Cluster 2** | **Enrichment Score: 5.110151575395163** | |
| | | |
| Category | Term | PValue |
| GOTERM_CC_FAT | GO:0005783~endoplasmic reticulum | 1.78E-12 |
| SP_PIR_KEYWORDS | endoplasmic reticulum | 3.53E-10 |
| GOTERM_CC_FAT | GO:0042175~nuclear envelope-endoplasmic reticulum network | 3.92E-09 |
| GOTERM_CC_FAT | GO:0044432~endoplasmic reticulum part | 2.58E-08 |
| GOTERM_CC_FAT | GO:0005789~endoplasmic reticulum membrane | 4.13E-08 |
| GOTERM_CC_FAT | GO:0012505~endomembrane system | 1.16E-06 |
| GOTERM_CC_FAT | GO:0031090~organelle membrane | 3.78E-05 |
| SP_PIR_KEYWORDS | oxidoreductase | 6.14E-04 |
| GOTERM_BP_FAT | GO:0055114~oxidation reduction | 1.52E-03 |
| GOTERM_CC_FAT | GO:0016021~integral to membrane | 5.14E-03 |
| GOTERM_CC_FAT | GO:0031224~intrinsic to membrane | 7.36E-03 |

| | | |
|---|---|---|
| UP_SEQ_FEATURE | transmembrane region | 8.14E-03 |
| SP_PIR_KEYWORDS | transmembrane | 8.52E-03 |
| SP_PIR_KEYWORDS | membrane | 1.02E-02 |
| | | |
| | | |
| **Annotation Cluster 3** | **Enrichment Score: 4.115762939193044** | |
| | | |
| Category | Term | PValue |
| GOTERM_BP_FAT | GO:0016126~sterol biosynthetic process | 3.76E-15 |
| GOTERM_BP_FAT | GO:0008203~cholesterol metabolic process | 8.53E-11 |
| GOTERM_CC_FAT | GO:0005792~microsome | 2.37E-02 |
| GOTERM_CC_FAT | GO:0042598~vesicular fraction | 2.51E-02 |
| GOTERM_CC_FAT | GO:0005624~membrane fraction | 3.61E-01 |
| GOTERM_CC_FAT | GO:0005626~insoluble fraction | 3.89E-01 |
| GOTERM_CC_FAT | GO:0000267~cell fraction | 5.79E-01 |

## Enriched annotation of drugs:

| Annotation type | Enrichment term | q-value |
|---|---|---|
| Drug target | HTR2A;5-hydroxytryptamine (serotonin) receptor 2A | 2.58E-06 |
| Drug target | HRH1;histamine receptor H1 | 6.88E-04 |
| Drug target | CYP2D6;cytochrome P450, family 2, subfamily D, polypeptide 6 | 4.18E-03 |
| Drug target | HTR1A;5-hydroxytryptamine (serotonin) receptor 1A | 4.18E-03 |
| Drug target | SLC6A4;solute carrier family 6 (neurotransmitter transporter, serotonin), member 4 | 5.62E-03 |
| Drug target | SLC6A2;solute carrier family 6 (neurotransmitter transporter, noradrenalin), member 2 | 7.19E-03 |
| Drug target | DRD4;dopamine receptor D4 | 7.69E-03 |
| Drug target | ADRA1A;adrenergic, alpha-1A-, receptor | 8.98E-03 |
| Drug target | DRD3;dopamine receptor D3 | 8.98E-03 |
| Drug target | DRD2;dopamine receptor D2 | 8.98E-03 |
| Drug target | DRD1;dopamine receptor D1 | 2.36E-02 |
| Drug target | HTR2C;5-hydroxytryptamine (serotonin) receptor 2C | 3.20E-02 |
| Drug target | CHRM3;cholinergic receptor, muscarinic 3 | 3.33E-02 |
| Drug target | CHRM5;cholinergic receptor, muscarinic 5 | 3.34E-02 |
| Drug target | HTR6;5-hydroxytryptamine (serotonin) receptor 6 | 3.34E-02 |
| Drug target | CHRM1;cholinergic receptor, muscarinic 1 | 3.87E-02 |
| Drug target | HTR7;5-hydroxytryptamine (serotonin) receptor 7 (adenylate cyclase-coupled) | 3.87E-02 |
| Drug target | CYP3A4;cytochrome P450, family 3, subfamily A, polypeptide 4 | 3.87E-02 |
| ATC code (2nd level) | N05; Psycholeptics | 7.76E-04 |
| ATC code (2nd level) | N06; Psychoanaleptics | 1.25E-02 |
| Side effect | paralytic ileus | 6.93E-06 |
| Side effect | galactorrhea | 8.54E-06 |
| Side effect | adenitis | 1.12E-04 |
| Side effect | gynecomastia | 4.10E-04 |
| Side effect | SIADH | 1.88E-03 |
| Side effect | testicular swelling | 5.55E-03 |
| Side effect | breast enlargement | 9.76E-03 |
| Side effect | urinary retention | 1.34E-02 |

| | | |
|---|---|---|
| Side effect | purpura | 2.53E-02 |
| Side effect | torticollis | 5.47E-02 |
| Side effect | petechiae | 7.08E-02 |

## Drug-induced transcriptional module: MCF7-3

**36 genes, 23 chemicals**

**Functional enrichment of genes:**

No significant annotation clusters found.

**Enriched annotation of drugs:**

No significant annotation terms found.

## Drug-induced transcriptional module: MCF7-4

**41 genes, 8 chemicals**

**Functional enrichment of genes:**

| **Annotation Cluster 1** | **Enrichment Score: 2.4971477268288993** | |
|---|---|---|
| | | |
| Category | Term | PValue |
| GOTERM_BP_FAT | GO:0001523~retinoid metabolic process | 9.69E-04 |
| GOTERM_BP_FAT | GO:0016101~diterpenoid metabolic process | 9.69E-04 |
| GOTERM_BP_FAT | GO:0006776~vitamin A metabolic process | 9.69E-04 |
| GOTERM_BP_FAT | GO:0006721~terpenoid metabolic process | 1.18E-03 |
| GOTERM_BP_FAT | GO:0042445~hormone metabolic process | 1.76E-03 |
| GOTERM_BP_FAT | GO:0006775~fat-soluble vitamin metabolic process | 1.94E-03 |
| SP_PIR_KEYWORDS | oxidoreductase | 3.42E-03 |
| GOTERM_BP_FAT | GO:0010817~regulation of hormone levels | 4.71E-03 |
| GOTERM_BP_FAT | GO:0006720~isoprenoid metabolic process | 6.67E-03 |
| GOTERM_BP_FAT | GO:0034754~cellular hormone metabolic process | 8.83E-03 |
| GOTERM_BP_FAT | GO:0006766~vitamin metabolic process | 1.69E-02 |
| GOTERM_BP_FAT | GO:0019748~secondary metabolic process | 1.84E-02 |
| | | |
| | | |
| **Annotation Cluster 2** | **Enrichment Score: 2.027363417394413** | |
| | | |
| Category | Term | PValue |
| GOTERM_BP_FAT | GO:0055114~oxidation reduction | 1.84E-03 |
| SP_PIR_KEYWORDS | oxidoreductase | 3.42E-03 |
| GOTERM_MF_FAT | GO:0009055~electron carrier activity | 1.87E-02 |
| SP_PIR_KEYWORDS | nadp | 6.61E-02 |
| | | |
| | | |
| **Annotation Cluster 3** | **Enrichment Score: 1.9845802687827583** | |
| | | |
| Category | Term | PValue |
| GOTERM_BP_FAT | GO:0060429~epithelium development | 2.04E-03 |
| GOTERM_BP_FAT | GO:0048732~gland development | 4.71E-03 |
| GOTERM_BP_FAT | GO:0048598~embryonic morphogenesis | 7.13E-03 |
| GOTERM_BP_FAT | GO:0035270~endocrine system development | 8.83E-03 |
| GOTERM_BP_FAT | GO:0048729~tissue morphogenesis | 1.20E-02 |
| GOTERM_BP_FAT | GO:0002009~morphogenesis of an epithelium | 3.49E-02 |
| GOTERM_BP_FAT | GO:0048568~embryonic organ development | 5.05E-02 |

**Enriched annotation of drugs:**

No significant annotation terms found.

## Drug-induced transcriptional module: MCF7-5

**30 genes, 13 chemicals**

**Functional enrichment of genes:**

| **Annotation Cluster 1** | **Enrichment Score: 1.6959442105562526** | |
|---|---|---|
| | | |
| Category | Term | PValue |
| SP_PIR_KEYWORDS | signal | 4.32E-03 |
| UP_SEQ_FEATURE | signal peptide | 4.36E-03 |
| UP_SEQ_FEATURE | disulfide bond | 1.55E-02 |
| SP_PIR_KEYWORDS | Secreted | 1.77E-02 |
| SP_PIR_KEYWORDS | disulfide bond | 1.99E-02 |
| SP_PIR_KEYWORDS | glycoprotein | 2.29E-02 |
| GOTERM_CC_FAT | GO:0005576~extracellular region | 3.32E-02 |
| GOTERM_CC_FAT | GO:0005615~extracellular space | 3.87E-02 |
| UP_SEQ_FEATURE | glycosylation site:N-linked (GlcNAc...) | 4.34E-02 |
| GOTERM_CC_FAT | GO:0044421~extracellular region part | 8.40E-02 |
| | | |
| | | |
| **Annotation Cluster 2** | **Enrichment Score: 1.437971770485521** | |
| | | |
| Category | Term | PValue |
| SP_PIR_KEYWORDS | Secreted | 1.77E-02 |
| GOTERM_BP_FAT | GO:0006935~chemotaxis | 2.24E-02 |
| GOTERM_BP_FAT | GO:0042330~taxis | 2.24E-02 |
| GOTERM_CC_FAT | GO:0005576~extracellular region | 3.32E-02 |
| GOTERM_BP_FAT | GO:0007626~locomotory behavior | 5.79E-02 |
| GOTERM_BP_FAT | GO:0007610~behavior | 1.38E-01 |

**Enriched annotation of drugs:**

No significant annotation terms found.

## Drug-induced transcriptional module: MCF7-6

**117 genes, 22 chemicals**

**Functional enrichment of genes:**

| **Annotation Cluster 1** | **Enrichment Score: 5.3086227488376165** | |
|---|---|---|
| | | |
| Category | Term | PValue |
| GOTERM_CC_FAT | GO:0031981~nuclear lumen | 5.21E-07 |
| GOTERM_CC_FAT | GO:0005654~nucleoplasm | 5.00E-06 |
| GOTERM_CC_FAT | GO:0070013~intracellular organelle lumen | 6.66E-06 |
| GOTERM_CC_FAT | GO:0043233~organelle lumen | 1.01E-05 |
| GOTERM_CC_FAT | GO:0031974~membrane-enclosed lumen | 1.63E-05 |
| | | |
| | | |
| **Annotation Cluster 2** | **Enrichment Score: 1.8457065285679297** | |
| | | |
| Category | Term | PValue |
| SP_PIR_KEYWORDS | transcription regulation | 4.40E-03 |
| GOTERM_BP_FAT | GO:0006350~transcription | 5.82E-03 |
| SP_PIR_KEYWORDS | dna-binding | 6.38E-03 |

| | | |
|---|---|---|
| SP_PIR_KEYWORDS | Transcription | 7.02E-03 |
| GOTERM_MF_FAT | GO:0003677~DNA binding | 8.19E-03 |
| GOTERM_BP_FAT | GO:0051252~regulation of RNA metabolic process | 1.17E-02 |
| GOTERM_BP_FAT | GO:0045449~regulation of transcription | 1.32E-02 |
| GOTERM_MF_FAT | GO:0030528~transcription regulator activity | 2.93E-02 |
| GOTERM_BP_FAT | GO:0006355~regulation of transcription, DNA-dependent | 3.17E-02 |
| GOTERM_MF_FAT | GO:0003700~transcription factor activity | 2.58E-01 |

## Enriched annotation of drugs:

No significant annotation terms found.

## Drug-induced transcriptional module: MCF7-7

## 26 genes, 40 chemicals

## Functional enrichment of genes:

| **Annotation Cluster 1** | **Enrichment Score: 3.7175243432526637** | |
|---|---|---|
| | | |
| Category | Term | PValue |
| SP_PIR_KEYWORDS | dna-binding | 5.17E-09 |
| GOTERM_MF_FAT | GO:0003677~DNA binding | 2.27E-07 |
| GOTERM_MF_FAT | GO:0003700~transcription factor activity | 3.19E-06 |
| SP_PIR_KEYWORDS | nucleus | 1.45E-05 |
| SP_PIR_KEYWORDS | transcription regulation | 2.62E-05 |
| GOTERM_BP_FAT | GO:0045944~positive regulation of transcription from RNA polymerase II promoter | 3.08E-05 |
| GOTERM_BP_FAT | GO:0045941~positive regulation of transcription | 4.00E-05 |
| GOTERM_BP_FAT | GO:0010628~positive regulation of gene expression | 4.57E-05 |
| GOTERM_BP_FAT | GO:0045935~positive regulation of nucleobase, nucleoside, nucleotide and nucleic acid metabolic process | 8.33E-05 |
| GOTERM_BP_FAT | GO:0010557~positive regulation of macromolecule biosynthetic process | 1.05E-04 |
| GOTERM_BP_FAT | GO:0051173~positive regulation of nitrogen compound metabolic process | 1.12E-04 |
| GOTERM_MF_FAT | GO:0043565~sequence-specific DNA binding | 1.21E-04 |
| GOTERM_BP_FAT | GO:0031328~positive regulation of cellular biosynthetic process | 1.39E-04 |
| GOTERM_BP_FAT | GO:0009891~positive regulation of biosynthetic process | 1.55E-04 |
| GOTERM_BP_FAT | GO:0051252~regulation of RNA metabolic process | 1.57E-04 |
| GOTERM_BP_FAT | GO:0045893~positive regulation of transcription, DNA-dependent | 1.70E-04 |
| GOTERM_BP_FAT | GO:0051254~positive regulation of RNA metabolic process | 1.80E-04 |
| GOTERM_BP_FAT | GO:0006357~regulation of transcription from RNA polymerase II promoter | 2.26E-04 |
| GOTERM_MF_FAT | GO:0030528~transcription regulator activity | 2.84E-04 |
| GOTERM_BP_FAT | GO:0006355~regulation of transcription, DNA-dependent | 6.96E-04 |
| GOTERM_BP_FAT | GO:0010604~positive regulation of macromolecule metabolic process | 7.66E-04 |
| GOTERM_BP_FAT | GO:0045449~regulation of transcription | 8.82E-04 |
| GOTERM_MF_FAT | GO:0003690~double-stranded DNA binding | 9.71E-04 |
| SP_PIR_KEYWORDS | Transcription | 1.19E-03 |
| GOTERM_BP_FAT | GO:0006350~transcription | 3.18E-03 |
| GOTERM_MF_FAT | GO:0043566~structure-specific DNA binding | 3.52E-03 |
| GOTERM_BP_FAT | GO:0031668~cellular response to extracellular stimulus | 7.35E-03 |
| GOTERM_MF_FAT | GO:0046983~protein dimerization activity | 1.58E-02 |
| GOTERM_MF_FAT | GO:0016563~transcription activator activity | 4.47E-02 |
| GOTERM_BP_FAT | GO:0007610~behavior | 1.49E-01 |
| | | |
| | | |
| **Annotation Cluster 2** | **Enrichment Score: 2.5205424604466624** | |
| | | |
| Category | Term | PValue |
| GOTERM_BP_FAT | GO:0045637~regulation of myeloid cell differentiation | 1.55E-05 |
| GOTERM_BP_FAT | GO:0045638~negative regulation of myeloid cell differentiation | 1.30E-03 |
| GOTERM_BP_FAT | GO:0045596~negative regulation of cell differentiation | 5.83E-02 |

| | | |
|---|---|---|
| GOTERM_BP_FAT | GO:0007242~intracellular signaling cascade | 7.05E-02 |
| | | |
| | | |
| **Annotation Cluster 3** | **Enrichment Score: 2.428289227575506** | |
| | | |
| Category | Term | PValue |
| INTERPRO | IPR004827:Basic-leucine zipper (bZIP) transcription factor | 3.15E-06 |
| SMART | SM00338:BRLZ | 1.16E-05 |
| GOTERM_BP_FAT | GO:0045944~positive regulation of transcription from RNA polymerase II promoter | 3.08E-05 |
| UP_SEQ_FEATURE | domain:Leucine-zipper | 4.00E-05 |
| GOTERM_BP_FAT | GO:0051591~response to cAMP | 4.10E-05 |
| UP_SEQ_FEATURE | DNA-binding region:Basic motif | 6.86E-05 |
| GOTERM_MF_FAT | GO:0043565~sequence-specific DNA binding | 1.21E-04 |
| GOTERM_BP_FAT | GO:0045893~positive regulation of transcription, DNA-dependent | 1.70E-04 |
| GOTERM_BP_FAT | GO:0051254~positive regulation of RNA metabolic process | 1.80E-04 |
| GOTERM_MF_FAT | GO:0003690~double-stranded DNA binding | 9.71E-04 |
| INTERPRO | IPR011616:bZIP transcription factor, bZIP-1 | 1.59E-03 |
| SP_PIR_KEYWORDS | leucine zipper | 2.42E-03 |
| BIOCARTA | h_arenrf2Pathway:Oxidative Stress Induced Gene Expression Via Nrf2 | 2.68E-03 |
| GOTERM_MF_FAT | GO:0043566~structure-specific DNA binding | 3.52E-03 |
| GOTERM_BP_FAT | GO:0009612~response to mechanical stimulus | 4.64E-03 |
| GOTERM_BP_FAT | GO:0034097~response to cytokine stimulus | 1.22E-02 |
| GOTERM_CC_FAT | GO:0043232~intracellular non-membrane-bounded organelle | 1.38E-02 |
| GOTERM_CC_FAT | GO:0043228~non-membrane-bounded organelle | 1.38E-02 |
| GOTERM_MF_FAT | GO:0046983~protein dimerization activity | 1.58E-02 |
| GOTERM_CC_FAT | GO:0031981~nuclear lumen | 2.48E-02 |
| GOTERM_BP_FAT | GO:0014070~response to organic cyclic substance | 2.58E-02 |
| GOTERM_BP_FAT | GO:0009628~response to abiotic stimulus | 2.64E-02 |
| GOTERM_CC_FAT | GO:0070013~intracellular organelle lumen | 5.88E-02 |
| GOTERM_CC_FAT | GO:0043233~organelle lumen | 6.33E-02 |
| GOTERM_BP_FAT | GO:0042493~response to drug | 6.73E-02 |
| GOTERM_CC_FAT | GO:0031974~membrane-enclosed lumen | 6.89E-02 |
| SP_PIR_KEYWORDS | Proto-oncogene | 7.05E-02 |
| GOTERM_MF_FAT | GO:0003702~RNA polymerase II transcription factor activity | 7.36E-02 |
| GOTERM_MF_FAT | GO:0008134~transcription factor binding | 9.21E-02 |
| GOTERM_CC_FAT | GO:0005730~nucleolus | 1.06E-01 |
| GOTERM_CC_FAT | GO:0005654~nucleoplasm | 1.99E-01 |
| GOTERM_CC_FAT | GO:0044451~nucleoplasm part | 2.36E-01 |

## Enriched annotation of drugs:

| **Annotation type** | **Enrichment term** | **q-value** |
|---|---|---|
| Drug target | CYP2D6;cytochrome P450, family 2, subfamily D, polypeptide 6 | 1.76E-02 |
| Drug target | CYP3A4;cytochrome P450, family 3, subfamily A, polypeptide 4 | 1.76E-02 |
| Drug target | KCNH2;potassium voltage-gated channel, subfamily H (eag-related), member 2 | 1.96E-02 |
| Drug target | CALM1;calmodulin 1 (phosphorylase kinase, delta) | 2.71E-02 |

## Drug-induced transcriptional module: MCF7-8

## 52 genes, 19 chemicals

## Functional enrichment of genes:

| **Annotation Cluster 1** | **Enrichment Score: 7.2823806719898885** | |
|---|---|---|
| | | |
| Category | Term | PValue |

| | | |
|---|---|---|
| SP_PIR_KEYWORDS | stress response | 4.01E-14 |
| GOTERM_BP_FAT | GO:0006986~response to unfolded protein | 1.54E-13 |
| GOTERM_BP_FAT | GO:0051789~response to protein stimulus | 2.60E-11 |
| SP_PIR_KEYWORDS | Chaperone | 2.00E-08 |
| GOTERM_BP_FAT | GO:0010033~response to organic substance | 3.39E-08 |
| GOTERM_MF_FAT | GO:0051082~unfolded protein binding | 9.28E-07 |
| GOTERM_BP_FAT | GO:0006457~protein folding | 8.49E-06 |
| GOTERM_MF_FAT | GO:0032559~adenyl ribonucleotide binding | 4.05E-02 |
| GOTERM_MF_FAT | GO:0005524~ATP binding | 8.25E-02 |
| | | |
| | | |
| **Annotation Cluster 2** | **Enrichment Score: 2.816076240251051** | |
| | | |
| Category | Term | PValue |
| GOTERM_MF_FAT | GO:0051082~unfolded protein binding | 9.28E-07 |
| GOTERM_BP_FAT | GO:0006457~protein folding | 8.49E-06 |
| INTERPRO | IPR002939:Chaperone DnaJ, C-terminal | 3.16E-04 |
| INTERPRO | IPR003095:Heat shock protein DnaJ | 2.79E-03 |
| SMART | SM00271:DnaJ | 6.43E-03 |
| INTERPRO | IPR015609:Molecular chaperone, heat shock protein, Hsp40, DnaJ | 9.53E-03 |
| INTERPRO | IPR001623:Heat shock protein DnaJ, N-terminal | 1.42E-02 |
| UP_SEQ_FEATURE | domain:J | 1.50E-02 |
| INTERPRO | IPR018253:Heat shock protein DnaJ, conserved site | 1.51E-02 |
| GOTERM_MF_FAT | GO:0031072~heat shock protein binding | 5.05E-02 |
| | | |
| | | |
| **Annotation Cluster 3** | **Enrichment Score: 2.7462153256165704** | |
| | | |
| Category | Term | PValue |
| INTERPRO | IPR001395:Aldo/keto reductase | 1.43E-05 |
| GOTERM_BP_FAT | GO:0055114~oxidation reduction | 8.88E-05 |
| GOTERM_MF_FAT | GO:0047115~trans-1,2-dihydrobenzene-1,2-diol dehydrogenase activity | 1.27E-04 |
| INTERPRO | IPR018170:Aldo/keto reductase, conserved site | 4.73E-04 |
| SP_PIR_KEYWORDS | oxidoreductase | 6.52E-04 |
| UP_SEQ_FEATURE | site:Lowers pKa of active site Tyr | 6.96E-04 |
| UP_SEQ_FEATURE | nucleotide phosphate-binding region:NADP | 1.86E-03 |
| SP_PIR_KEYWORDS | nadp | 2.17E-03 |
| GOTERM_MF_FAT | GO:0016628~oxidoreductase activity, acting on the CH-CH group of donors, NAD or NADP as acceptor | 4.22E-03 |
| GOTERM_MF_FAT | GO:0033764~steroid dehydrogenase activity, acting on the CH-OH group of donors, NAD or NADP as acceptor | 4.22E-03 |
| GOTERM_MF_FAT | GO:0016229~steroid dehydrogenase activity | 5.42E-03 |
| KEGG_PATHWAY | hsa00140:Steroid hormone biosynthesis | 5.85E-03 |
| KEGG_PATHWAY | hsa00980:Metabolism of xenobiotics by cytochrome P450 | 1.62E-02 |
| UP_SEQ_FEATURE | active site:Proton donor | 1.96E-02 |
| UP_SEQ_FEATURE | binding site:Substrate | 2.57E-01 |

## Enriched annotation of drugs:

No significant annotation terms found.

## Drug-induced transcriptional module: MCF7-9

## 49 genes, 37 chemicals

## Functional enrichment of genes:

| **Annotation Cluster 1** | **Enrichment Score: 1.6103071866224787** | |
|---|---|---|
| | | |
| Category | Term | PValue |
| GOTERM_BP_FAT | GO:0007186~G-protein coupled receptor protein signaling pathway | 3.33E-03 |
| GOTERM_BP_FAT | GO:0032103~positive regulation of response to external stimulus | 2.30E-02 |

| | | |
|---|---|---|
| GOTERM_BP_FAT | GO:0048584~positive regulation of response to stimulus | 5.37E-02 |
| GOTERM_BP_FAT | GO:0032101~regulation of response to external stimulus | 8.80E-02 |
| | | |
| | | |
| **Annotation Cluster 2** | **Enrichment Score: 1.5661474893139846** | |
| | | |
| Category | Term | PValue |
| GOTERM_BP_FAT | GO:0060429~epithelium development | 5.43E-03 |
| GOTERM_BP_FAT | GO:0035295~tube development | 7.90E-03 |
| GOTERM_BP_FAT | GO:0048729~tissue morphogenesis | 2.44E-02 |
| GOTERM_BP_FAT | GO:0016331~morphogenesis of embryonic epithelium | 2.53E-02 |
| GOTERM_BP_FAT | GO:0001822~kidney development | 4.26E-02 |
| GOTERM_BP_FAT | GO:0002009~morphogenesis of an epithelium | 5.64E-02 |
| GOTERM_BP_FAT | GO:0001655~urogenital system development | 5.80E-02 |
| GOTERM_BP_FAT | GO:0048598~embryonic morphogenesis | 7.99E-02 |

## Enriched annotation of drugs:

| Annotation type | Enrichment term | q-value |
|---|---|---|
| Drug target | ESR1;estrogen receptor 1 | 2.98E-04 |
| ATC code (2nd level) | G03; Sex hormones and modulators of the genital system | 3.33E-08 |
| ATC code (2nd level) | L02; Endocrine therapy | 7.76E-04 |
| Side effect | vulvovaginitis | 4.87E-03 |
| Side effect | pelvic pain | 7.53E-03 |
| Side effect | endometriosis | 9.59E-03 |
| Side effect | pneumonia | 9.76E-03 |
| Side effect | varicose vein | 1.53E-02 |
| Side effect | Endocrine Disorders | 2.10E-02 |
| Side effect | uterine fibroids | 2.53E-02 |
| Side effect | breast neoplasm | 5.98E-02 |
| Side effect | breast pain | 7.08E-02 |

## Drug-induced transcriptional module: MCF7-10

## 25 genes, 20 chemicals

## Functional enrichment of genes:

| **Annotation Cluster 1** | **Enrichment Score: 1.9807471628902773** | |
|---|---|---|
| | | |
| Category | Term | PValue |
| UP_SEQ_FEATURE | repeat:WD 6 | 4.36E-03 |
| UP_SEQ_FEATURE | repeat:WD 5 | 7.19E-03 |
| INTERPRO | IPR019782:WD40 repeat 2 | 8.40E-03 |
| UP_SEQ_FEATURE | repeat:WD 4 | 9.01E-03 |
| INTERPRO | IPR017986:WD40 repeat, region | 9.36E-03 |
| INTERPRO | IPR019781:WD40 repeat, subgroup | 1.02E-02 |
| UP_SEQ_FEATURE | repeat:WD 3 | 1.07E-02 |
| UP_SEQ_FEATURE | repeat:WD 2 | 1.14E-02 |
| UP_SEQ_FEATURE | repeat:WD 1 | 1.14E-02 |
| SP_PIR_KEYWORDS | wd repeat | 1.15E-02 |
| INTERPRO | IPR001680:WD40 repeat | 1.30E-02 |
| INTERPRO | IPR019775:WD40 repeat, conserved site | 1.42E-02 |
| SMART | SM00320:WD40 | 1.60E-02 |
| INTERPRO | IPR015943:WD40/YVTN repeat-like | 1.71E-02 |

## Enriched annotation of drugs:

No significant annotation terms found.

## Drug-induced transcriptional module: MCF7-11

### 28 genes, 17 chemicals

### Functional enrichment of genes:

| **Annotation Cluster 1** | **Enrichment Score: 1.599734111168645** | |
|---|---|---|
| | | |
| Category | Term | PValue |
| GOTERM_MF_FAT | GO:0003700~transcription factor activity | 7.93E-04 |
| SP_PIR_KEYWORDS | zinc-finger | 7.41E-03 |
| GOTERM_MF_FAT | GO:0008270~zinc ion binding | 7.69E-03 |
| SP_PIR_KEYWORDS | zinc | 8.69E-03 |
| SP_PIR_KEYWORDS | differentiation | 1.68E-02 |
| GOTERM_MF_FAT | GO:0046914~transition metal ion binding | 2.94E-02 |
| SP_PIR_KEYWORDS | metal-binding | 5.63E-02 |
| GOTERM_MF_FAT | GO:0046872~metal ion binding | 2.01E-01 |
| GOTERM_MF_FAT | GO:0043169~cation binding | 2.08E-01 |
| GOTERM_MF_FAT | GO:0043167~ion binding | 2.20E-01 |

### Enriched annotation of drugs:

No significant annotation terms found.

## Drug-induced transcriptional module: MCF7-12

### 32 genes, 38 chemicals

### Functional enrichment of genes:

No significant annotation clusters found.

### Enriched annotation of drugs:

No significant annotation terms found.

## Drug-induced transcriptional module: MCF7-13

No of genes: 37 No of Drugs: 6

### Functional enrichment of genes:

| **Annotation Cluster 1** | **Enrichment Score: 4.614567942552974** | |
|---|---|---|
| | | |
| Category | Term | PValue |
| GOTERM_CC_FAT | GO:0005856~cytoskeleton | 5.31E-07 |
| GOTERM_CC_FAT | GO:0043228~non-membrane-bounded organelle | 3.83E-06 |
| GOTERM_CC_FAT | GO:0043232~intracellular non-membrane-bounded organelle | 3.83E-06 |
| SP_PIR_KEYWORDS | phosphoprotein | 4.48E-02 |
| | | |
| | | |
| **Annotation Cluster 2** | **Enrichment Score: 3.883602303161378** | |
| | | |
| Category | Term | PValue |
| PIR_SUPERFAMILY | PIRSF002306:tubulin | 8.19E-10 |
| INTERPRO | IPR018316:Tubulin/FtsZ, 2-layer sandwich domain | 8.97E-10 |
| INTERPRO | IPR017975:Tubulin, conserved site | 1.45E-09 |
| INTERPRO | IPR003008:Tubulin/FtsZ, GTPase domain | 1.45E-09 |
| INTERPRO | IPR000217:Tubulin | 1.45E-09 |

| | | |
|---|---|---|
| GOTERM_BP_FAT | GO:0051258~protein polymerization | 5.40E-08 |
| KEGG_PATHWAY | hsa05130:Pathogenic Escherichia coli infection | 3.46E-06 |
| UP_SEQ_FEATURE | nucleotide phosphate-binding region:GTP | 4.61E-06 |
| GOTERM_BP_FAT | GO:0007018~microtubule-based movement | 5.88E-06 |
| SP_PIR_KEYWORDS | gtp-binding | 9.10E-06 |
| KEGG_PATHWAY | hsa04540:Gap junction | 2.09E-05 |
| GOTERM_BP_FAT | GO:0007017~microtubule-based process | 5.79E-05 |
| GOTERM_MF_FAT | GO:0003924~GTPase activity | 6.36E-05 |
| GOTERM_BP_FAT | GO:0043623~cellular protein complex assembly | 1.09E-04 |
| INTERPRO | IPR002453:Beta tubulin | 1.10E-04 |
| GOTERM_MF_FAT | GO:0005525~GTP binding | 1.14E-04 |
| GOTERM_MF_FAT | GO:0019001~guanyl nucleotide binding | 1.18E-04 |
| GOTERM_MF_FAT | GO:0032561~guanyl ribonucleotide binding | 1.18E-04 |
| SP_PIR_KEYWORDS | microtubule | 2.07E-04 |
| INTERPRO | IPR013838:Beta tubulin, autoregulation binding site | 2.73E-04 |
| INTERPRO | IPR002452:Alpha tubulin | 2.73E-04 |
| GOTERM_CC_FAT | GO:0044430~cytoskeletal part | 4.67E-04 |
| GOTERM_MF_FAT | GO:0005198~structural molecule activity | 1.05E-03 |
| GOTERM_CC_FAT | GO:0005874~microtubule | 1.15E-03 |
| GOTERM_BP_FAT | GO:0034622~cellular macromolecular complex assembly | 2.36E-03 |
| GOTERM_BP_FAT | GO:0034621~cellular macromolecular complex subunit organization | 3.82E-03 |
| GOTERM_CC_FAT | GO:0015630~microtubule cytoskeleton | 6.74E-03 |
| GOTERM_BP_FAT | GO:0006461~protein complex assembly | 1.88E-02 |
| GOTERM_BP_FAT | GO:0070271~protein complex biogenesis | 1.88E-02 |
| GOTERM_BP_FAT | GO:0065003~macromolecular complex assembly | 4.89E-02 |
| SP_PIR_KEYWORDS | nucleotide-binding | 5.67E-02 |
| GOTERM_BP_FAT | GO:0043933~macromolecular complex subunit organization | 5.88E-02 |
| GOTERM_CC_FAT | GO:0005829~cytosol | 9.01E-02 |
| GOTERM_MF_FAT | GO:0032555~purine ribonucleotide binding | 2.38E-01 |
| GOTERM_MF_FAT | GO:0032553~ribonucleotide binding | 2.38E-01 |
| GOTERM_MF_FAT | GO:0017076~purine nucleotide binding | 2.76E-01 |
| GOTERM_MF_FAT | GO:0000166~nucleotide binding | 3.12E-01 |
| | | |
| | | |
| **Annotation Cluster 3** | **Enrichment Score: 2.41559463369049** | |
| | | |
| Category | Term | PValue |
| GOTERM_CC_FAT | GO:0001725~stress fiber | 2.96E-03 |
| GOTERM_CC_FAT | GO:0032432~actin filament bundle | 3.32E-03 |
| GOTERM_CC_FAT | GO:0042641~actomyosin | 3.70E-03 |
| GOTERM_CC_FAT | GO:0015629~actin cytoskeleton | 5.99E-03 |

## Enriched annotation of drugs:

No significant annotation terms found.

## <u>Drug-induced transcriptional module: MCF7-14</u>

## 78 genes, 14 chemicals

## Functional enrichment of genes:

| **Annotation Cluster 1** | **Enrichment Score: 36.99530232122559** | |
|---|---|---|
| | | |
| Category | Term | PValue |
| GOTERM_BP_FAT | GO:0022403~cell cycle phase | 4.45E-44 |
| GOTERM_BP_FAT | GO:0022402~cell cycle process | 2.34E-42 |
| GOTERM_BP_FAT | GO:0000278~mitotic cell cycle | 2.97E-41 |
| GOTERM_BP_FAT | GO:0000279~M phase | 4.29E-41 |
| GOTERM_BP_FAT | GO:0007049~cell cycle | 3.42E-40 |
| SP_PIR_KEYWORDS | cell cycle | 5.42E-38 |
| GOTERM_BP_FAT | GO:0000280~nuclear division | 5.90E-37 |

| | | |
|---|---|---|
| GOTERM_BP_FAT | GO:0007067~mitosis | 5.90E-37 |
| GOTERM_BP_FAT | GO:0000087~M phase of mitotic cell cycle | 1.12E-36 |
| GOTERM_BP_FAT | GO:0048285~organelle fission | 3.20E-36 |
| SP_PIR_KEYWORDS | cell division | 8.65E-35 |
| SP_PIR_KEYWORDS | mitosis | 3.22E-33 |
| GOTERM_BP_FAT | GO:0051301~cell division | 1.35E-28 |
| | | |
| | | |
| **Annotation Cluster 2** | **Enrichment Score: 18.741037758071844** | |
| | | |
| Category | Term | PValue |
| GOTERM_CC_FAT | GO:0005819~spindle | 7.11E-29 |
| GOTERM_CC_FAT | GO:0015630~microtubule cytoskeleton | 1.65E-27 |
| GOTERM_CC_FAT | GO:0044430~cytoskeletal part | 3.09E-21 |
| GOTERM_CC_FAT | GO:0005856~cytoskeleton | 1.49E-18 |
| GOTERM_CC_FAT | GO:0043228~non-membrane-bounded organelle | 6.21E-14 |
| GOTERM_CC_FAT | GO:0043232~intracellular non-membrane-bounded organelle | 6.21E-14 |
| SP_PIR_KEYWORDS | cytoskeleton | 3.12E-12 |
| | | |
| | | |
| **Annotation Cluster 3** | **Enrichment Score: 15.894019730507372** | |
| | | |
| Category | Term | PValue |
| GOTERM_BP_FAT | GO:0007017~microtubule-based process | 1.18E-23 |
| GOTERM_BP_FAT | GO:0000226~microtubule cytoskeleton organization | 7.51E-18 |
| GOTERM_BP_FAT | GO:0007051~spindle organization | 1.09E-13 |
| GOTERM_BP_FAT | GO:0007010~cytoskeleton organization | 2.75E-11 |

## Enriched annotation of drugs:

No significant annotation terms found.

## <u>Drug-induced transcriptional module: MCF7-15</u>

## 30 genes, 8 chemicals

## Functional enrichment of genes:

| | | |
|---|---|---|
| **Annotation Cluster 1** | **Enrichment Score: 2.48709088347047** | |
| | | |
| Category | Term | PValue |
| GOTERM_BP_FAT | GO:0050900~leukocyte migration | 2.23E-04 |
| GOTERM_BP_FAT | GO:0006928~cell motion | 3.08E-04 |
| GOTERM_BP_FAT | GO:0016477~cell migration | 2.44E-03 |
| GOTERM_BP_FAT | GO:0048870~cell motility | 2.95E-03 |
| GOTERM_BP_FAT | GO:0051674~localization of cell | 2.95E-03 |
| GOTERM_BP_FAT | GO:0032101~regulation of response to external stimulus | 3.09E-02 |
| GOTERM_BP_FAT | GO:0042127~regulation of cell proliferation | 8.65E-02 |
| | | |
| | | |
| **Annotation Cluster 2** | **Enrichment Score: 2.2717777889572592** | |
| | | |
| Category | Term | PValue |
| GOTERM_BP_FAT | GO:0006952~defense response | 3.78E-05 |
| UP_SEQ_FEATURE | disulfide bond | 1.26E-03 |
| SP_PIR_KEYWORDS | disulfide bond | 1.78E-03 |
| SP_PIR_KEYWORDS | signal | 5.67E-03 |
| UP_SEQ_FEATURE | signal peptide | 5.72E-03 |
| GOTERM_CC_FAT | GO:0005576~extracellular region | 2.72E-02 |
| SP_PIR_KEYWORDS | glycoprotein | 7.29E-02 |
| UP_SEQ_FEATURE | glycosylation site:N-linked (GlcNAc...) | 1.23E-01 |
| | | |

| | | |
|---|---|---|
| **Annotation Cluster 3** | **Enrichment Score: 1.423046800910826** | |
| | | |
| Category | Term | PValue |
| SP_PIR_KEYWORDS | immune response | 1.52E-05 |
| SP_PIR_KEYWORDS | immunoglobulin receptor | 2.76E-04 |
| SP_PIR_KEYWORDS | transmembrane protein | 2.82E-03 |
| UP_SEQ_FEATURE | topological domain:Extracellular | 3.60E-02 |
| UP_SEQ_FEATURE | topological domain:Cytoplasmic | 6.78E-02 |
| INTERPRO | IPR007110:Immunoglobulin-like | 8.78E-02 |
| UP_SEQ_FEATURE | transmembrane region | 1.06E-01 |
| SP_PIR_KEYWORDS | transmembrane | 1.08E-01 |
| INTERPRO | IPR013783:Immunoglobulin-like fold | 1.12E-01 |
| SP_PIR_KEYWORDS | cell membrane | 1.80E-01 |
| SP_PIR_KEYWORDS | membrane | 1.88E-01 |
| SP_PIR_KEYWORDS | receptor | 1.91E-01 |
| GOTERM_CC_FAT | GO:0005886~plasma membrane | 2.13E-01 |
| GOTERM_CC_FAT | GO:0016021~integral to membrane | 2.98E-01 |
| GOTERM_CC_FAT | GO:0031224~intrinsic to membrane | 3.38E-01 |

## Enriched annotation of drugs:

No significant annotation terms found.

## <u>Drug-induced transcriptional module: MCF7-16</u>

## 33 genes, 70 chemicals

## Functional enrichment of genes:

| | | |
|---|---|---|
| **Annotation Cluster 1** | **Enrichment Score: 2.2117809708907226** | |
| | | |
| Category | Term | PValue |
| SP_PIR_KEYWORDS | rna-binding | 1.89E-03 |
| GOTERM_MF_FAT | GO:0043021~ribonucleoprotein binding | 4.30E-03 |
| SP_PIR_KEYWORDS | ribonucleoprotein | 6.02E-03 |
| GOTERM_MF_FAT | GO:0003723~RNA binding | 7.95E-03 |
| GOTERM_CC_FAT | GO:0030529~ribonucleoprotein complex | 2.25E-02 |
| | | |
| | | |
| **Annotation Cluster 2** | **Enrichment Score: 1.6257104219159297** | |
| | | |
| Category | Term | PValue |
| SP_PIR_KEYWORDS | ribonucleoprotein | 6.02E-03 |
| GOTERM_CC_FAT | GO:0033279~ribosomal subunit | 7.87E-03 |
| GOTERM_CC_FAT | GO:0044445~cytosolic part | 1.39E-02 |
| GOTERM_BP_FAT | GO:0006412~translation | 1.44E-02 |
| SP_PIR_KEYWORDS | protein biosynthesis | 1.73E-02 |
| GOTERM_CC_FAT | GO:0015935~small ribosomal subunit | 1.90E-02 |
| GOTERM_CC_FAT | GO:0030529~ribonucleoprotein complex | 2.25E-02 |
| SP_PIR_KEYWORDS | ribosome | 2.29E-02 |
| GOTERM_CC_FAT | GO:0005840~ribosome | 2.63E-02 |
| GOTERM_CC_FAT | GO:0022626~cytosolic ribosome | 3.70E-02 |
| GOTERM_BP_FAT | GO:0006414~translational elongation | 4.98E-02 |
| GOTERM_CC_FAT | GO:0005829~cytosol | 5.24E-02 |
| KEGG_PATHWAY | hsa03010:Ribosome | 5.28E-02 |
| SP_PIR_KEYWORDS | ribosomal protein | 8.09E-02 |

## Enriched annotation of drugs:

No significant annotation terms found.

## Drug-induced transcriptional module: MCF7-17

### 27 genes, 25 chemicals

### Functional enrichment of genes:

| Annotation Cluster 1 | Enrichment Score: 1.9436344829645589 | |
|---|---|---|
| | | |
| Category | Term | PValue |
| GOTERM_CC_FAT | GO:0044449~contractile fiber part | 1.01E-03 |
| GOTERM_CC_FAT | GO:0043292~contractile fiber | 1.20E-03 |
| GOTERM_CC_FAT | GO:0030018~Z disc | 3.08E-03 |
| GOTERM_CC_FAT | GO:0031674~I band | 3.73E-03 |
| GOTERM_CC_FAT | GO:0005856~cytoskeleton | 1.06E-02 |
| GOTERM_CC_FAT | GO:0030017~sarcomere | 1.09E-02 |
| GOTERM_CC_FAT | GO:0030016~myofibril | 1.51E-02 |
| GOTERM_CC_FAT | GO:0044430~cytoskeletal part | 1.60E-01 |
| GOTERM_CC_FAT | GO:0005886~plasma membrane | 8.31E-01 |

### Enriched annotation of drugs:

| Annotation type | Enrichment term | q-value |
|---|---|---|
| Side effect | polymyalgia rheumatica | 5.37E-02 |

## Drug-induced transcriptional module: MCF7-18

### 38 genes, 15 chemicals

### Functional enrichment of genes:

No significant annotation clusters found.

### Enriched annotation of drugs:

No significant annotation terms found.

## Drug-induced transcriptional module: MCF7-19

### 22 genes, 9 chemicals

### Functional enrichment of genes:

| Annotation Cluster 1 | Enrichment Score: 1.6215761917459408 | |
|---|---|---|
| | | |
| Category | Term | PValue |
| UP_SEQ_FEATURE | repeat:WD 4 | 6.95E-03 |
| UP_SEQ_FEATURE | repeat:WD 3 | 8.31E-03 |
| UP_SEQ_FEATURE | repeat:WD 1 | 8.83E-03 |
| UP_SEQ_FEATURE | repeat:WD 2 | 8.83E-03 |
| SP_PIR_KEYWORDS | wd repeat | 8.94E-03 |
| INTERPRO | IPR019775:WD40 repeat, conserved site | 9.63E-03 |
| INTERPRO | IPR019782:WD40 repeat 2 | 5.21E-02 |
| INTERPRO | IPR017986:WD40 repeat, region | 5.59E-02 |
| INTERPRO | IPR019781:WD40 repeat, subgroup | 5.91E-02 |
| INTERPRO | IPR001680:WD40 repeat | 6.92E-02 |
| INTERPRO | IPR015943:WD40/YVTN repeat-like | 8.28E-02 |
| SMART | SM00320:WD40 | 9.10E-02 |
| | | |
| | | |

| Annotation Cluster 2 | Enrichment Score: 1.5767426174909447 | |
|---|---|---|
| | | |
| Category | Term | PValue |
| GOTERM_BP_FAT | GO:0035239~tube morphogenesis | 1.43E-02 |
| GOTERM_BP_FAT | GO:0035295~tube development | 3.44E-02 |
| GOTERM_BP_FAT | GO:0007389~pattern specification process | 3.79E-02 |

## Enriched annotation of drugs:

| Annotation type | Enrichment term | q-value |
|---|---|---|
| ATC code (2nd level) | C01; Cardiac therapy | 9.32E-02 |

## Drug-induced transcriptional module: MCF7-20

### 30 genes, 215 chemicals

### Functional enrichment of genes:

No significant annotation clusters found.

### Enriched annotation of drugs:

No significant annotation terms found.

## Drug-induced transcriptional module: MCF7-21

### 24 genes, 19 chemicals

### Functional enrichment of genes:

| Annotation Cluster 1 | Enrichment Score: 1.4292195057910047 | |
|---|---|---|
| | | |
| Category | Term | PValue |
| GOTERM_BP_FAT | GO:0006511~ubiquitin-dependent protein catabolic process | 9.97E-03 |
| GOTERM_BP_FAT | GO:0019941~modification-dependent protein catabolic process | 1.39E-02 |
| GOTERM_BP_FAT | GO:0043632~modification-dependent macromolecule catabolic process | 1.39E-02 |
| GOTERM_BP_FAT | GO:0051603~proteolysis involved in cellular protein catabolic process | 1.68E-02 |
| GOTERM_BP_FAT | GO:0044257~cellular protein catabolic process | 1.69E-02 |
| GOTERM_BP_FAT | GO:0030163~protein catabolic process | 1.93E-02 |
| GOTERM_BP_FAT | GO:0010498~proteasomal protein catabolic process | 2.10E-02 |
| GOTERM_BP_FAT | GO:0043161~proteasomal ubiquitin-dependent protein catabolic process | 2.10E-02 |
| GOTERM_BP_FAT | GO:0044265~cellular macromolecule catabolic process | 3.30E-02 |
| GOTERM_BP_FAT | GO:0009057~macromolecule catabolic process | 4.14E-02 |
| GOTERM_BP_FAT | GO:0006508~proteolysis | 5.66E-02 |
| SP_PIR_KEYWORDS | hydrolase | 6.63E-02 |
| GOTERM_BP_FAT | GO:0010604~positive regulation of macromolecule metabolic process | 4.17E-01 |
| SP_PIR_KEYWORDS | nucleus | 9.83E-01 |

### Enriched annotation of drugs:

| Annotation type | Enrichment term | q-value |
|---|---|---|
| Drug target | TTR;transthyretin | 7.75E-02 |

## Drug-induced transcriptional module: MCF7-22

### 40 genes, 16 chemicals

## Functional enrichment of genes:

| **Annotation Cluster 1** | **Enrichment Score: 1.429693197747981** | |
|---|---|---|
| | | |
| Category | Term | PValue |
| GOTERM_BP_FAT | GO:0006396~RNA processing | 1.71E-03 |
| SP_PIR_KEYWORDS | mrna splicing | 3.61E-02 |
| SP_PIR_KEYWORDS | mrna processing | 5.69E-02 |
| GOTERM_BP_FAT | GO:0008380~RNA splicing | 7.24E-02 |
| GOTERM_BP_FAT | GO:0006397~mRNA processing | 8.84E-02 |
| GOTERM_BP_FAT | GO:0016071~mRNA metabolic process | 1.18E-01 |
| | | |
| | | |
| **Annotation Cluster 2** | **Enrichment Score: 1.373263850884335** | |
| | | |
| Category | Term | PValue |
| SP_PIR_KEYWORDS | rna-binding | 6.25E-03 |
| GOTERM_MF_FAT | GO:0003723~RNA binding | 1.72E-02 |
| SMART | SM00360:RRM | 8.26E-02 |
| INTERPRO | IPR000504:RNA recognition motif, RNP-1 | 1.22E-01 |
| INTERPRO | IPR012677:Nucleotide-binding, alpha-beta plait | 1.26E-01 |

## Enriched annotation of drugs:

No significant annotation terms found.

## Drug-induced transcriptional module: MCF7-23

## 90 genes, 9 chemicals

## Functional enrichment of genes:

| **Annotation Cluster 1** | **Enrichment Score: 2.2530696360797933** | |
|---|---|---|
| | | |
| Category | Term | PValue |
| GOTERM_CC_FAT | GO:0000267~cell fraction | 6.18E-04 |
| GOTERM_CC_FAT | GO:0005624~membrane fraction | 1.44E-02 |
| GOTERM_CC_FAT | GO:0005626~insoluble fraction | 1.96E-02 |
| | | |
| | | |
| **Annotation Cluster 2** | **Enrichment Score: 1.918217017829944** | |
| | | |
| Category | Term | PValue |
| GOTERM_BP_FAT | GO:0046822~regulation of nucleocytoplasmic transport | 6.76E-04 |
| GOTERM_BP_FAT | GO:0032386~regulation of intracellular transport | 1.09E-03 |
| GOTERM_BP_FAT | GO:0033157~regulation of intracellular protein transport | 4.66E-03 |
| GOTERM_BP_FAT | GO:0046824~positive regulation of nucleocytoplasmic transport | 5.83E-03 |
| GOTERM_BP_FAT | GO:0032880~regulation of protein localization | 1.03E-02 |
| GOTERM_BP_FAT | GO:0042990~regulation of transcription factor import into nucleus | 1.23E-02 |
| GOTERM_BP_FAT | GO:0032388~positive regulation of intracellular transport | 1.38E-02 |
| GOTERM_BP_FAT | GO:0042306~regulation of protein import into nucleus | 3.11E-02 |
| GOTERM_BP_FAT | GO:0051223~regulation of protein transport | 3.20E-02 |
| GOTERM_BP_FAT | GO:0070201~regulation of establishment of protein localization | 4.00E-02 |
| GOTERM_BP_FAT | GO:0060341~regulation of cellular localization | 4.77E-02 |
| GOTERM_BP_FAT | GO:0051050~positive regulation of transport | 1.44E-01 |
| | | |
| | | |
| **Annotation Cluster 3** | **Enrichment Score: 1.471528083498598** | |
| | | |
| Category | Term | PValue |
| GOTERM_BP_FAT | GO:0016192~vesicle-mediated transport | 1.74E-04 |
| GOTERM_BP_FAT | GO:0046907~intracellular transport | 5.93E-03 |

| | | |
|---|---|---|
| GOTERM_BP_FAT | GO:0008104~protein localization | 1.15E-02 |
| SP_PIR_KEYWORDS | protein transport | 2.48E-02 |
| SP_PIR_KEYWORDS | er-golgi transport | 3.13E-02 |
| GOTERM_BP_FAT | GO:0015031~protein transport | 5.91E-02 |
| GOTERM_BP_FAT | GO:0045184~establishment of protein localization | 6.22E-02 |
| SP_PIR_KEYWORDS | transport | 9.36E-02 |
| GOTERM_BP_FAT | GO:0034613~cellular protein localization | 2.37E-01 |
| GOTERM_BP_FAT | GO:0070727~cellular macromolecule localization | 2.43E-01 |
| GOTERM_BP_FAT | GO:0006886~intracellular protein transport | 3.59E-01 |

## Enriched annotation of drugs:

No significant annotation terms found.

## Drug-induced transcriptional module: MCF7-24

## 425 genes, 9 chemicals

## Functional enrichment of genes:

| **Annotation Cluster 1** | **Enrichment Score: 4.113962366347981** | |
|---|---|---|
| | | |
| Category | Term | PValue |
| UP_SEQ_FEATURE | glycosylation site:N-linked (GlcNAc...) | 1.54E-06 |
| SP_PIR_KEYWORDS | glycoprotein | 1.63E-06 |
| UP_SEQ_FEATURE | disulfide bond | 3.29E-04 |
| SP_PIR_KEYWORDS | signal | 5.79E-04 |
| UP_SEQ_FEATURE | signal peptide | 5.89E-04 |
| SP_PIR_KEYWORDS | disulfide bond | 7.31E-04 |
| | | |
| **Annotation Cluster 2** | **Enrichment Score: 3.355247450452249** | |
| | | |
| Category | Term | PValue |
| UP_SEQ_FEATURE | glycosylation site:N-linked (GlcNAc...) | 1.54E-06 |
| SP_PIR_KEYWORDS | glycoprotein | 1.63E-06 |
| UP_SEQ_FEATURE | topological domain:Extracellular | 7.67E-05 |
| UP_SEQ_FEATURE | topological domain:Cytoplasmic | 3.92E-04 |
| UP_SEQ_FEATURE | transmembrane region | 0.001857655 |
| SP_PIR_KEYWORDS | membrane | 0.001948228 |
| SP_PIR_KEYWORDS | transmembrane | 0.002200752 |
| GOTERM_CC_FAT | GO:0031224~intrinsic to membrane | 0.003563762 |
| GOTERM_CC_FAT | GO:0005886~plasma membrane | 0.010999768 |
| GOTERM_CC_FAT | GO:0016021~integral to membrane | 0.011830181 |

## Enriched annotation of drugs:

No significant annotation terms found.

## Drug-induced transcriptional module: MCF7-25

## 451 genes, 7 chemicals

## Functional enrichment of genes:

| **Annotation Cluster 1** | **Enrichment Score: 3.5218683141227958** | |
|---|---|---|
| | | |
| Category | Term | PValue |
| UP_SEQ_FEATURE | domain:PX | 4.97E-05 |
| INTERPRO | IPR001683:Phox-like | 6.04E-05 |

| | | |
|---|---|---|
| SMART | SM00312:PX | 7.77E-05 |
| GOTERM_MF_FAT | GO:0035091~phosphoinositide binding | 8.19E-04 |
| GOTERM_MF_FAT | GO:0005543~phospholipid binding | 1.31E-03 |
| GOTERM_MF_FAT | GO:0008289~lipid binding | 2.94E-03 |
| | | |
| | | |
| **Annotation Cluster 2** | **Enrichment Score: 1.7885916384018243** | |
| | | |
| Category | Term | PValue |
| SP_PIR_KEYWORDS | receptor | 2.42E-03 |
| UP_SEQ_FEATURE | topological domain:Extracellular | 1.08E-02 |
| SP_PIR_KEYWORDS | cell membrane | 1.40E-02 |
| UP_SEQ_FEATURE | topological domain:Cytoplasmic | 1.45E-02 |
| GOTERM_CC_FAT | GO:0005887~integral to plasma membrane | 1.90E-02 |
| GOTERM_CC_FAT | GO:0031226~intrinsic to plasma membrane | 2.32E-02 |
| GOTERM_CC_FAT | GO:0005886~plasma membrane | 2.99E-02 |
| GOTERM_CC_FAT | GO:0044459~plasma membrane part | 7.03E-02 |

### Enriched annotation of drugs:

No significant annotation terms found.

# PC3 Dataset:

## Drug-induced transcriptional module: PC3-1

### 25 genes, 27 chemicals

### Functional enrichment of genes:

| **Annotation Cluster 1** | **Enrichment Score: 3.248551065259976** | |
|---|---|---|
| | | |
| Category | Term | PValue |
| GOTERM_BP_FAT | GO:0006915~apoptosis | 4.66E-05 |
| GOTERM_BP_FAT | GO:0012501~programmed cell death | 5.26E-05 |
| GOTERM_BP_FAT | GO:0008219~cell death | 1.57E-04 |
| GOTERM_BP_FAT | GO:0016265~death | 1.64E-04 |
| SP_PIR_KEYWORDS | Apoptosis | 5.24E-03 |
| GOTERM_BP_FAT | GO:0032268~regulation of cellular protein metabolic process | 9.76E-02 |
| | | |
| | | |
| **Annotation Cluster 2** | **Enrichment Score: 2.1116054159437305** | |
| | | |
| Category | Term | PValue |
| GOTERM_BP_FAT | GO:0051384~response to glucocorticoid stimulus | 9.89E-09 |
| GOTERM_BP_FAT | GO:0031960~response to corticosteroid stimulus | 1.44E-08 |
| GOTERM_BP_FAT | GO:0002237~response to molecule of bacterial origin | 9.99E-07 |
| GOTERM_BP_FAT | GO:0010033~response to organic substance | 1.03E-06 |
| UP_SEQ_FEATURE | domain:Leucine-zipper | 1.56E-06 |
| GOTERM_BP_FAT | GO:0048545~response to steroid hormone stimulus | 1.95E-06 |
| INTERPRO | IPR004827:Basic-leucine zipper (bZIP) transcription factor | 3.77E-06 |
| SMART | SM00338:BRLZ | 9.08E-06 |
| GOTERM_BP_FAT | GO:0009617~response to bacterium | 1.16E-05 |
| GOTERM_BP_FAT | GO:0032496~response to lipopolysaccharide | 1.80E-05 |
| SP_PIR_KEYWORDS | DNA binding | 2.75E-05 |
| SP_PIR_KEYWORDS | leucine zipper | 5.77E-05 |
| GOTERM_BP_FAT | GO:0051591~response to cAMP | 6.21E-05 |
| UP_SEQ_FEATURE | DNA-binding region:Basic motif | 8.17E-05 |
| GOTERM_BP_FAT | GO:0009991~response to extracellular stimulus | 8.88E-05 |
| GOTERM_BP_FAT | GO:0045768~positive regulation of anti-apoptosis | 9.05E-05 |
| GOTERM_BP_FAT | GO:0009725~response to hormone stimulus | 9.56E-05 |

| | | |
|---|---|---|
| GOTERM_BP_FAT | GO:0009719~response to endogenous stimulus | 1.63E-04 |
| GOTERM_BP_FAT | GO:0014070~response to organic cyclic substance | 1.70E-04 |
| GOTERM_BP_FAT | GO:0045767~regulation of anti-apoptosis | 1.87E-04 |
| GOTERM_BP_FAT | GO:0009628~response to abiotic stimulus | 7.41E-04 |
| GOTERM_BP_FAT | GO:0051412~response to corticosterone stimulus | 8.79E-04 |
| GOTERM_BP_FAT | GO:0034097~response to cytokine stimulus | 9.01E-04 |
| GOTERM_BP_FAT | GO:0010035~response to inorganic substance | 1.12E-03 |
| GOTERM_BP_FAT | GO:0007565~female pregnancy | 1.16E-03 |
| GOTERM_BP_FAT | GO:0051385~response to mineralocorticoid stimulus | 1.18E-03 |
| GOTERM_BP_FAT | GO:0042493~response to drug | 1.28E-03 |
| GOTERM_BP_FAT | GO:0008284~positive regulation of cell proliferation | 1.38E-03 |
| INTERPRO | IPR011616:bZIP transcription factor, bZIP-1 | 1.73E-03 |
| KEGG_PATHWAY | hsa05200:Pathways in cancer | 1.75E-03 |
| SP_PIR_KEYWORDS | dna-binding | 2.12E-03 |
| KEGG_PATHWAY | hsa04620:Toll-like receptor signaling pathway | 2.34E-03 |
| GOTERM_BP_FAT | GO:0009416~response to light stimulus | 2.35E-03 |
| GOTERM_BP_FAT | GO:0048661~positive regulation of smooth muscle cell proliferation | 3.04E-03 |
| GOTERM_MF_FAT | GO:0003677~DNA binding | 3.74E-03 |
| KEGG_PATHWAY | hsa04010:MAPK signaling pathway | 4.43E-03 |
| GOTERM_BP_FAT | GO:0042127~regulation of cell proliferation | 5.60E-03 |
| BIOCARTA | h_cdMacPathway:Cadmium induces DNA synthesis and proliferation in macrophages | 5.71E-03 |
| GOTERM_BP_FAT | GO:0045944~positive regulation of transcription from RNA polymerase II promoter | 5.91E-03 |
| GOTERM_BP_FAT | GO:0009612~response to mechanical stimulus | 6.05E-03 |
| GOTERM_BP_FAT | GO:0048660~regulation of smooth muscle cell proliferation | 6.05E-03 |
| BBID | 114.Genomic_reformatting_Brain_Ischemia | 6.38E-03 |
| GOTERM_CC_FAT | GO:0000785~chromatin | 6.67E-03 |
| GOTERM_BP_FAT | GO:0006979~response to oxidative stress | 6.69E-03 |
| GOTERM_BP_FAT | GO:0008285~negative regulation of cell proliferation | 7.72E-03 |
| BIOCARTA | h_arenrf2Pathway:Oxidative Stress Induced Gene Expression Via Nrf2 | 7.79E-03 |
| GOTERM_BP_FAT | GO:0009314~response to radiation | 8.26E-03 |
| GOTERM_BP_FAT | GO:0031667~response to nutrient levels | 8.26E-03 |
| GOTERM_MF_FAT | GO:0043565~sequence-specific DNA binding | 8.72E-03 |
| GOTERM_BP_FAT | GO:0031668~cellular response to extracellular stimulus | 9.55E-03 |
| SP_PIR_KEYWORDS | nucleus | 1.02E-02 |
| GOTERM_BP_FAT | GO:0051252~regulation of RNA metabolic process | 1.04E-02 |
| GOTERM_MF_FAT | GO:0003700~transcription factor activity | 1.11E-02 |
| BIOCARTA | h_il6Pathway:IL 6 signaling pathway | 1.28E-02 |
| GOTERM_BP_FAT | GO:0000302~response to reactive oxygen species | 1.33E-02 |
| GOTERM_MF_FAT | GO:0003690~double-stranded DNA binding | 1.68E-02 |
| GOTERM_BP_FAT | GO:0045893~positive regulation of transcription, DNA-dependent | 1.69E-02 |
| GOTERM_BP_FAT | GO:0007611~learning or memory | 1.75E-02 |
| GOTERM_BP_FAT | GO:0051254~positive regulation of RNA metabolic process | 1.75E-02 |
| GOTERM_BP_FAT | GO:0043065~positive regulation of apoptosis | 1.85E-02 |
| GOTERM_BP_FAT | GO:0043068~positive regulation of programmed cell death | 1.87E-02 |
| GOTERM_BP_FAT | GO:0010942~positive regulation of cell death | 1.89E-02 |
| SP_PIR_KEYWORDS | transcription regulation | 2.01E-02 |
| GOTERM_BP_FAT | GO:0051094~positive regulation of developmental process | 2.08E-02 |
| KEGG_PATHWAY | hsa04662:B cell receptor signaling pathway | 2.21E-02 |
| BIOCARTA | h_tcrPathway:T Cell Receptor Signaling Pathway | 2.24E-02 |
| BIOCARTA | h_tollPathway:Toll-Like Receptor Pathway | 2.24E-02 |
| BIOCARTA | h_il1rPathway:Signal transduction through IL1R | 2.42E-02 |
| GOTERM_BP_FAT | GO:0045941~positive regulation of transcription | 2.74E-02 |
| GOTERM_BP_FAT | GO:0006355~regulation of transcription, DNA-dependent | 2.88E-02 |
| GOTERM_BP_FAT | GO:0010628~positive regulation of gene expression | 2.93E-02 |
| KEGG_PATHWAY | hsa04660:T cell receptor signaling pathway | 3.25E-02 |
| GOTERM_BP_FAT | GO:0007568~aging | 3.31E-02 |
| GOTERM_BP_FAT | GO:0051241~negative regulation of multicellular organismal process | 3.38E-02 |
| GOTERM_BP_FAT | GO:0032870~cellular response to hormone stimulus | 3.61E-02 |
| GOTERM_MF_FAT | GO:0043566~structure-specific DNA binding | 3.86E-02 |
| GOTERM_BP_FAT | GO:0045935~positive regulation of nucleobase, nucleoside, nucleotide | 3.95E-02 |

| | | |
|---|---|---|
| | and nucleic acid metabolic process | |
| GOTERM_BP_FAT | GO:0007610~behavior | 4.20E-02 |
| GOTERM_CC_FAT | GO:0044427~chromosomal part | 4.37E-02 |
| GOTERM_BP_FAT | GO:0010557~positive regulation of macromolecule biosynthetic process | 4.42E-02 |
| GOTERM_BP_FAT | GO:0051173~positive regulation of nitrogen compound metabolic process | 4.56E-02 |
| GOTERM_BP_FAT | GO:0031328~positive regulation of cellular biosynthetic process | 5.07E-02 |
| GOTERM_BP_FAT | GO:0009891~positive regulation of biosynthetic process | 5.34E-02 |
| GOTERM_BP_FAT | GO:0006357~regulation of transcription from RNA polymerase II promoter | 6.40E-02 |
| GOTERM_CC_FAT | GO:0005694~chromosome | 6.54E-02 |
| GOTERM_MF_FAT | GO:0003702~RNA polymerase II transcription factor activity | 7.36E-02 |
| GOTERM_MF_FAT | GO:0046983~protein dimerization activity | 7.57E-02 |
| SP_PIR_KEYWORDS | Proto-oncogene | 7.59E-02 |
| GOTERM_BP_FAT | GO:0007166~cell surface receptor linked signal transduction | 7.67E-02 |
| GOTERM_BP_FAT | GO:0051240~positive regulation of multicellular organismal process | 7.99E-02 |
| GOTERM_BP_FAT | GO:0045449~regulation of transcription | 8.52E-02 |
| GOTERM_BP_FAT | GO:0045597~positive regulation of cell differentiation | 8.61E-02 |
| GOTERM_MF_FAT | GO:0030528~transcription regulator activity | 9.11E-02 |
| GOTERM_MF_FAT | GO:0008134~transcription factor binding | 9.21E-02 |
| GOTERM_BP_FAT | GO:0032268~regulation of cellular protein metabolic process | 9.76E-02 |
| GOTERM_BP_FAT | GO:0010604~positive regulation of macromolecule metabolic process | 1.14E-01 |
| BIOCARTA | h_mapkPathway:MAPKinase Signaling Pathway | 1.42E-01 |
| GOTERM_BP_FAT | GO:0050890~cognition | 1.52E-01 |
| GOTERM_CC_FAT | GO:0070013~intracellular organelle lumen | 1.70E-01 |
| SP_PIR_KEYWORDS | repressor | 1.72E-01 |
| SP_PIR_KEYWORDS | Transcription | 1.75E-01 |
| GOTERM_CC_FAT | GO:0043233~organelle lumen | 1.80E-01 |
| GOTERM_CC_FAT | GO:0031974~membrane-enclosed lumen | 1.93E-01 |
| GOTERM_CC_FAT | GO:0031981~nuclear lumen | 1.99E-01 |
| GOTERM_BP_FAT | GO:0009611~response to wounding | 2.46E-01 |
| GOTERM_CC_FAT | GO:0000267~cell fraction | 2.94E-01 |
| GOTERM_BP_FAT | GO:0050877~neurological system process | 3.15E-01 |
| GOTERM_CC_FAT | GO:0005654~nucleoplasm | 3.25E-01 |
| GOTERM_CC_FAT | GO:0043232~intracellular non-membrane-bounded organelle | 3.25E-01 |
| GOTERM_CC_FAT | GO:0043228~non-membrane-bounded organelle | 3.25E-01 |
| GOTERM_CC_FAT | GO:0044451~nucleoplasm part | 3.36E-01 |
| GOTERM_BP_FAT | GO:0006350~transcription | 3.38E-01 |
| GOTERM_BP_FAT | GO:0042592~homeostatic process | 4.31E-01 |
| GOTERM_CC_FAT | GO:0005730~nucleolus | 4.51E-01 |
| UP_SEQ_FEATURE | disulfide bond | 7.15E-01 |
| SP_PIR_KEYWORDS | disulfide bond | 7.41E-01 |
| SP_PIR_KEYWORDS | signal | 8.24E-01 |
| UP_SEQ_FEATURE | signal peptide | 8.24E-01 |
| UP_SEQ_FEATURE | splice variant | 1.00E+00 |
| SP_PIR_KEYWORDS | alternative splicing | 1.00E+00 |
| | | |
| | | |
| **Annotation Cluster 3** | **Enrichment Score: 2.087399373933161** | |
| | | |
| Category | Term | PValue |
| BIOCARTA | h_atmPathway:ATM Signaling Pathway | 5.56E-04 |
| KEGG_PATHWAY | hsa05200:Pathways in cancer | 1.75E-03 |
| GOTERM_CC_FAT | GO:0005829~cytosol | 5.61E-01 |

## Enriched annotation of drugs:

No significant annotation terms found.

## <u>Drug-induced transcriptional module: PC3-2</u>

### 34 genes,  28 chemicals

## Functional enrichment of genes:

| **Annotation Cluster 1** | **Enrichment Score: 1.6853315330704641** | |
|---|---|---|
| | | |
| Category | Term | PValue |
| SP_PIR_KEYWORDS | Aminoacyl-tRNA synthetase | 1.39E-04 |
| KEGG_PATHWAY | hsa00970:Aminoacyl-tRNA biosynthesis | 1.50E-04 |
| GOTERM_MF_FAT | GO:0016875~ligase activity, forming carbon-oxygen bonds | 1.77E-04 |
| GOTERM_MF_FAT | GO:0016876~ligase activity, forming aminoacyl-tRNA and related compounds | 1.77E-04 |
| GOTERM_MF_FAT | GO:0004812~aminoacyl-tRNA ligase activity | 1.77E-04 |
| GOTERM_BP_FAT | GO:0006418~tRNA aminoacylation for protein translation | 3.70E-04 |
| GOTERM_BP_FAT | GO:0043039~tRNA aminoacylation | 3.70E-04 |
| GOTERM_BP_FAT | GO:0043038~amino acid activation | 3.70E-04 |
| GOTERM_MF_FAT | GO:0000049~tRNA binding | 9.05E-04 |
| INTERPRO | IPR014729:Rossmann-like alpha/beta/alpha sandwich fold | 5.10E-03 |
| SP_PIR_KEYWORDS | ligase | 5.48E-03 |
| GOTERM_BP_FAT | GO:0006399~tRNA metabolic process | 5.87E-03 |
| SP_PIR_KEYWORDS | protein biosynthesis | 1.88E-02 |
| GOTERM_BP_FAT | GO:0034660~ncRNA metabolic process | 3.90E-02 |
| GOTERM_BP_FAT | GO:0006412~translation | 8.84E-02 |
| GOTERM_MF_FAT | GO:0003723~RNA binding | 9.39E-02 |
| GOTERM_CC_FAT | GO:0005625~soluble fraction | 1.75E-01 |
| GOTERM_MF_FAT | GO:0032555~purine ribonucleotide binding | 2.05E-01 |
| GOTERM_MF_FAT | GO:0032553~ribonucleotide binding | 2.05E-01 |
| GOTERM_MF_FAT | GO:0000166~nucleotide binding | 2.18E-01 |
| GOTERM_MF_FAT | GO:0005524~ATP binding | 2.32E-01 |
| GOTERM_MF_FAT | GO:0017076~purine nucleotide binding | 2.35E-01 |
| GOTERM_MF_FAT | GO:0032559~adenyl ribonucleotide binding | 2.37E-01 |
| GOTERM_MF_FAT | GO:0030554~adenyl nucleotide binding | 2.70E-01 |
| GOTERM_MF_FAT | GO:0001883~purine nucleoside binding | 2.79E-01 |
| GOTERM_MF_FAT | GO:0001882~nucleoside binding | 2.84E-01 |
| SP_PIR_KEYWORDS | nucleotide-binding | 3.53E-01 |
| SP_PIR_KEYWORDS | atp-binding | 3.94E-01 |
| SP_PIR_KEYWORDS | acetylation | 4.77E-01 |
| GOTERM_CC_FAT | GO:0005829~cytosol | 5.07E-01 |
| GOTERM_CC_FAT | GO:0000267~cell fraction | 7.15E-01 |
| | | |
| | | |
| **Annotation Cluster 2** | **Enrichment Score: 1.3190782261768748** | |
| | | |
| Category | Term | PValue |
| GOTERM_BP_FAT | GO:0042981~regulation of apoptosis | 8.31E-03 |
| GOTERM_BP_FAT | GO:0043067~regulation of programmed cell death | 8.73E-03 |
| GOTERM_BP_FAT | GO:0010941~regulation of cell death | 8.90E-03 |
| GOTERM_BP_FAT | GO:0043066~negative regulation of apoptosis | 2.16E-02 |
| GOTERM_BP_FAT | GO:0043069~negative regulation of programmed cell death | 2.25E-02 |
| GOTERM_BP_FAT | GO:0060548~negative regulation of cell death | 2.28E-02 |
| GOTERM_BP_FAT | GO:0006917~induction of apoptosis | 7.51E-02 |
| GOTERM_BP_FAT | GO:0012502~induction of programmed cell death | 7.51E-02 |
| GOTERM_BP_FAT | GO:0006916~anti-apoptosis | 1.40E-01 |
| GOTERM_BP_FAT | GO:0043065~positive regulation of apoptosis | 1.49E-01 |
| GOTERM_BP_FAT | GO:0043068~positive regulation of programmed cell death | 1.50E-01 |
| GOTERM_BP_FAT | GO:0010942~positive regulation of cell death | 1.51E-01 |
| GOTERM_BP_FAT | GO:0006952~defense response | 3.72E-01 |

## Enriched annotation of drugs:

| **Annotation type** | **Enrichment term** | **q-value** |
|---|---|---|
| Drug target | HRH1;histamine receptor H1 | 1.23E-03 |
| Drug target | CYP2D6;cytochrome P450, family 2, subfamily D, polypeptide 6 | 9.84E-03 |

| | | |
|---|---|---|
| Drug target | HTR2A;5-hydroxytryptamine (serotonin) receptor 2A | 1.81E-02 |
| Drug target | ABCB1;ATP-binding cassette, sub-family B (MDR/TAP), member 1 | 5.50E-02 |
| Drug target | KCNH2;potassium voltage-gated channel, subfamily H (eag-related), member 2 | 7.53E-02 |
| Drug target | CALM1;calmodulin 1 (phosphorylase kinase, delta) | 9.65E-02 |
| Side effect | paralytic ileus | 2.33E-05 |
| Side effect | galactorrhea | 8.97E-03 |
| Side effect | gynecomastia | 2.77E-02 |
| **Annotation type** | **Enrichment term** | **q-value** |
| Side effect | paralytic ileus | 7.29E-03 |
| **Annotation type** | **Enrichment term** | **q-value** |
| Drug target | DRD4;dopamine receptor D4 | 3.95E-02 |
| Drug target | DRD2;dopamine receptor D2 | 7.15E-02 |
| Drug target | DRD3;dopamine receptor D3 | 7.53E-02 |

## Drug-induced transcriptional module: PC3-3

## 38 genes, 25 chemicals

## Functional enrichment of genes:

| **Annotation Cluster 1** | **Enrichment Score: 3.964121128847142** | |
|---|---|---|
| | | |
| Category | Term | PValue |
| SP_PIR_KEYWORDS | signal | 3.44E-06 |
| UP_SEQ_FEATURE | signal peptide | 3.49E-06 |
| UP_SEQ_FEATURE | disulfide bond | 3.79E-05 |
| SP_PIR_KEYWORDS | disulfide bond | 6.34E-05 |
| SP_PIR_KEYWORDS | Secreted | 4.68E-03 |
| GOTERM_CC_FAT | GO:0005576~extracellular region | 1.22E-02 |
| | | |
| | | |
| **Annotation Cluster 2** | **Enrichment Score: 2.101131649765943** | |
| | | |
| Category | Term | PValue |
| SP_PIR_KEYWORDS | unfolded protein response | 1.55E-03 |
| GOTERM_BP_FAT | GO:0051789~response to protein stimulus | 4.08E-03 |
| GOTERM_BP_FAT | GO:0006986~response to unfolded protein | 1.70E-02 |
| GOTERM_BP_FAT | GO:0010033~response to organic substance | 3.67E-02 |
| | | |
| | | |
| **Annotation Cluster 3** | **Enrichment Score: 1.683165060154669** | |
| | | |
| Category | Term | PValue |
| UP_SEQ_FEATURE | short sequence motif:Prevents secretion from ER | 8.92E-04 |
| GOTERM_CC_FAT | GO:0005788~endoplasmic reticulum lumen | 2.38E-03 |
| SP_PIR_KEYWORDS | endoplasmic reticulum | 2.61E-03 |
| GOTERM_CC_FAT | GO:0044432~endoplasmic reticulum part | 4.88E-03 |
| GOTERM_CC_FAT | GO:0005783~endoplasmic reticulum | 7.13E-03 |
| INTERPRO | IPR000886:Endoplasmic reticulum, targeting sequence | 8.92E-03 |
| GOTERM_CC_FAT | GO:0043233~organelle lumen | 6.97E-01 |
| GOTERM_CC_FAT | GO:0031974~membrane-enclosed lumen | 7.17E-01 |
| GOTERM_CC_FAT | GO:0070013~intracellular organelle lumen | 8.25E-01 |

## Enriched annotation of drugs:

| Annotation type | Enrichment term | q-value |
|---|---|---|
| Drug target | HRH1;histamine receptor H1 | 1.00E-05 |
| Drug target | HTR2A;5-hydroxytryptamine (serotonin) receptor 2A | 2.96E-05 |
| Drug target | DRD2;dopamine receptor D2 | 3.78E-05 |
| Drug target | DRD1;dopamine receptor D1 | 1.03E-04 |
| Drug target | ADRA1A;adrenergic, alpha-1A-, receptor | 2.60E-04 |
| Drug target | DRD3;dopamine receptor D3 | 2.60E-04 |
| Drug target | CHRM2;cholinergic receptor, muscarinic 2 | 4.15E-04 |
| Drug target | CHRM4;cholinergic receptor, muscarinic 4 | 1.33E-03 |
| Drug target | HTR2C;5-hydroxytryptamine (serotonin) receptor 2C | 1.40E-03 |
| Drug target | SLC6A2;solute carrier family 6 (neurotransmitter transporter, noradrenalin), member 2 | 1.45E-03 |
| Drug target | CHRM5;cholinergic receptor, muscarinic 5 | 1.55E-03 |
| Drug target | CHRM1;cholinergic receptor, muscarinic 1 | 1.77E-03 |
| Drug target | ADCY1;adenylate cyclase 1 (brain) | 8.41E-03 |
| Drug target | CHRM3;cholinergic receptor, muscarinic 3 | 9.84E-03 |
| Drug target | SLC6A4;solute carrier family 6 (neurotransmitter transporter, serotonin), member 4 | 1.08E-02 |
| Drug target | HTR6;5-hydroxytryptamine (serotonin) receptor 6 | 1.28E-02 |
| Drug target | HTR1A;5-hydroxytryptamine (serotonin) receptor 1A | 1.81E-02 |
| Drug target | HRH4;histamine receptor H4 | 3.71E-02 |
| Drug target | DRD4;dopamine receptor D4 | 5.10E-02 |
| ATC code (2nd level) | R06; Antihistamines for systemic use | 1.89E-02 |
| Side effect | paralytic ileus | 3.85E-04 |
| Side effect | galactorrhea | 8.97E-03 |
| Side effect | gynecomastia | 2.77E-02 |
| Side effect | breast enlargement | 7.46E-02 |
| Side effect | urinary retention | 9.40E-02 |

## Drug-induced transcriptional module: PC3-4

## 37 genes, 10 chemicals

## Functional enrichment of genes:

| Annotation Cluster 1 | Enrichment Score: 1.5889998419871902 | |
|---|---|---|
| | | |
| Category | Term | PValue |
| SP_PIR_KEYWORDS | chemotaxis | 2.29E-04 |
| GOTERM_BP_FAT | GO:0007626~locomotory behavior | 1.53E-03 |
| INTERPRO | IPR001811:Small chemokine, interleukin-8-like | 1.65E-03 |
| SMART | SM00199:SCY | 2.05E-03 |
| GOTERM_MF_FAT | GO:0008009~chemokine activity | 2.32E-03 |
| GOTERM_MF_FAT | GO:0042379~chemokine receptor binding | 3.11E-03 |
| GOTERM_BP_FAT | GO:0006935~chemotaxis | 3.31E-03 |
| GOTERM_BP_FAT | GO:0042330~taxis | 3.31E-03 |
| GOTERM_BP_FAT | GO:0006874~cellular calcium ion homeostasis | 5.19E-03 |
| GOTERM_CC_FAT | GO:0005576~extracellular region | 5.52E-03 |
| GOTERM_BP_FAT | GO:0055074~calcium ion homeostasis | 5.66E-03 |
| GOTERM_BP_FAT | GO:0006875~cellular metal ion homeostasis | 6.32E-03 |
| GOTERM_BP_FAT | GO:0055065~metal ion homeostasis | 6.85E-03 |

| | | |
|---|---|---|
| GOTERM_BP_FAT | GO:0007610~behavior | 9.41E-03 |
| GOTERM_BP_FAT | GO:0030005~cellular di-, tri-valent inorganic cation homeostasis | 9.67E-03 |
| GOTERM_BP_FAT | GO:0055066~di-, tri-valent inorganic cation homeostasis | 1.15E-02 |
| GOTERM_BP_FAT | GO:0030003~cellular cation homeostasis | 1.39E-02 |
| GOTERM_BP_FAT | GO:0055080~cation homeostasis | 1.74E-02 |
| SP_PIR_KEYWORDS | cytokine | 1.83E-02 |
| GOTERM_MF_FAT | GO:0005125~cytokine activity | 2.39E-02 |
| GOTERM_BP_FAT | GO:0006873~cellular ion homeostasis | 3.61E-02 |
| GOTERM_CC_FAT | GO:0044421~extracellular region part | 3.63E-02 |
| GOTERM_BP_FAT | GO:0055082~cellular chemical homeostasis | 3.70E-02 |
| SP_PIR_KEYWORDS | Secreted | 3.93E-02 |
| GOTERM_BP_FAT | GO:0050801~ion homeostasis | 4.14E-02 |
| KEGG_PATHWAY | hsa04060:Cytokine-cytokine receptor interaction | 4.46E-02 |
| KEGG_PATHWAY | hsa04062:Chemokine signaling pathway | 4.54E-02 |
| SP_PIR_KEYWORDS | signal | 4.79E-02 |
| UP_SEQ_FEATURE | signal peptide | 4.82E-02 |
| GOTERM_BP_FAT | GO:0019725~cellular homeostasis | 6.96E-02 |
| GOTERM_BP_FAT | GO:0048878~chemical homeostasis | 7.29E-02 |
| GOTERM_BP_FAT | GO:0006955~immune response | 1.17E-01 |
| SP_PIR_KEYWORDS | glycoprotein | 1.57E-01 |
| GOTERM_BP_FAT | GO:0042592~homeostatic process | 2.06E-01 |
| GOTERM_BP_FAT | GO:0007186~G-protein coupled receptor protein signaling pathway | 2.26E-01 |
| UP_SEQ_FEATURE | glycosylation site:N-linked (GlcNAc...) | 2.31E-01 |
| GOTERM_BP_FAT | GO:0007267~cell-cell signaling | 2.37E-01 |
| UP_SEQ_FEATURE | disulfide bond | 2.38E-01 |
| GOTERM_CC_FAT | GO:0005615~extracellular space | 2.40E-01 |
| SP_PIR_KEYWORDS | disulfide bond | 2.70E-01 |
| GOTERM_BP_FAT | GO:0007155~cell adhesion | 3.22E-01 |
| GOTERM_BP_FAT | GO:0022610~biological adhesion | 3.22E-01 |
| GOTERM_BP_FAT | GO:0007166~cell surface receptor linked signal transduction | 4.70E-01 |

## Enriched annotation of drugs:

No significant annotation terms found.

## Drug-induced transcriptional module: PC3-5

## 47 genes, 41 chemicals

## Functional enrichment of genes:

| **Annotation Cluster 1** | **Enrichment Score: 3.074546789718209** | |
|---|---|---|
| | | |
| Category | Term | PValue |
| SMART | SM00702:P4Hc | 1.86E-05 |
| INTERPRO | IPR006620:Prolyl 4-hydroxylase, alpha subunit | 4.20E-05 |
| INTERPRO | IPR005123:Oxoglutarate and iron-dependent oxygenase | 4.20E-05 |
| SP_PIR_KEYWORDS | dioxygenase | 4.91E-05 |
| GOTERM_MF_FAT | GO:0016702~oxidoreductase activity, acting on single donors with incorporation of molecular oxygen, incorporation of two atoms of oxygen | 5.04E-05 |
| GOTERM_MF_FAT | GO:0016701~oxidoreductase activity, acting on single donors with incorporation of molecular oxygen | 5.04E-05 |
| SP_PIR_KEYWORDS | vitamin c | 6.02E-05 |
| UP_SEQ_FEATURE | domain:Fe2OG dioxygenase | 6.04E-05 |
| GOTERM_MF_FAT | GO:0031418~L-ascorbic acid binding | 8.22E-05 |
| GOTERM_MF_FAT | GO:0016706~oxidoreductase activity, acting on paired donors, with incorporation or reduction of molecular oxygen, 2-oxoglutarate as one donor, and incorporation of one atom each of oxygen into both donors | 8.22E-05 |
| GOTERM_MF_FAT | GO:0031543~peptidyl-proline dioxygenase activity | 4.38E-04 |
| UP_SEQ_FEATURE | metal ion-binding site:Iron | 5.62E-04 |
| UP_SEQ_FEATURE | binding site:2-oxoglutarate | 9.64E-04 |
| GOTERM_MF_FAT | GO:0019842~vitamin binding | 7.08E-03 |
| GOTERM_MF_FAT | GO:0031406~carboxylic acid binding | 9.47E-03 |

| | | |
|---|---|---|
| SP_PIR_KEYWORDS | iron | 9.86E-03 |
| GOTERM_MF_FAT | GO:0005506~iron ion binding | 1.42E-02 |
| GOTERM_BP_FAT | GO:0055114~oxidation reduction | 2.15E-02 |
| SP_PIR_KEYWORDS | oxidoreductase | 3.06E-02 |
| SP_PIR_KEYWORDS | metal-binding | 2.74E-01 |
| GOTERM_MF_FAT | GO:0046914~transition metal ion binding | 6.72E-01 |
| | | |
| | | |
| **Annotation Cluster 2** | **Enrichment Score: 2.93750678585954** | |
| | | |
| Category | Term | PValue |
| GOTERM_BP_FAT | GO:0001666~response to hypoxia | 6.41E-11 |
| GOTERM_BP_FAT | GO:0070482~response to oxygen levels | 1.10E-10 |
| GOTERM_BP_FAT | GO:0048878~chemical homeostasis | 1.64E-02 |
| GOTERM_BP_FAT | GO:0042592~homeostatic process | 3.17E-02 |
| GOTERM_BP_FAT | GO:0019725~cellular homeostasis | 5.91E-02 |
| GOTERM_BP_FAT | GO:0006873~cellular ion homeostasis | 3.14E-01 |
| GOTERM_BP_FAT | GO:0055082~cellular chemical homeostasis | 3.18E-01 |
| GOTERM_BP_FAT | GO:0050801~ion homeostasis | 3.37E-01 |
| GOTERM_BP_FAT | GO:0006952~defense response | 5.01E-01 |
| | | |
| | | |
| **Annotation Cluster 3** | **Enrichment Score: 2.501759814474319** | |
| | | |
| Category | Term | PValue |
| SP_PIR_KEYWORDS | glycolysis | 2.96E-07 |
| GOTERM_BP_FAT | GO:0019318~hexose metabolic process | 4.85E-07 |
| GOTERM_BP_FAT | GO:0006096~glycolysis | 6.39E-07 |
| GOTERM_BP_FAT | GO:0006006~glucose metabolic process | 1.35E-06 |
| GOTERM_BP_FAT | GO:0005996~monosaccharide metabolic process | 1.41E-06 |
| GOTERM_BP_FAT | GO:0006007~glucose catabolic process | 2.18E-06 |
| GOTERM_BP_FAT | GO:0019320~hexose catabolic process | 4.59E-06 |
| GOTERM_BP_FAT | GO:0046365~monosaccharide catabolic process | 5.14E-06 |
| GOTERM_BP_FAT | GO:0046164~alcohol catabolic process | 8.74E-06 |
| GOTERM_BP_FAT | GO:0044275~cellular carbohydrate catabolic process | 1.06E-05 |
| KEGG_PATHWAY | hsa00010:Glycolysis / Gluconeogenesis | 1.30E-05 |
| GOTERM_BP_FAT | GO:0016052~carbohydrate catabolic process | 2.54E-05 |
| GOTERM_BP_FAT | GO:0006091~generation of precursor metabolites and energy | 3.57E-05 |
| KEGG_PATHWAY | hsa00051:Fructose and mannose metabolism | 4.83E-05 |
| GOTERM_MF_FAT | GO:0019200~carbohydrate kinase activity | 5.38E-05 |
| SP_PIR_KEYWORDS | phosphotransferase | 1.31E-04 |
| COG_ONTOLOGY | Carbohydrate transport and metabolism | 7.91E-04 |
| SP_PIR_KEYWORDS | kinase | 2.15E-03 |
| KEGG_PATHWAY | hsa00052:Galactose metabolism | 6.10E-03 |
| SP_PIR_KEYWORDS | allosteric enzyme | 7.01E-03 |
| KEGG_PATHWAY | hsa00500:Starch and sucrose metabolism | 1.00E-02 |
| SP_PIR_KEYWORDS | ATP | 1.39E-02 |
| SP_PIR_KEYWORDS | transferase | 3.05E-02 |
| UP_SEQ_FEATURE | binding site:Substrate | 5.80E-02 |
| GOTERM_MF_FAT | GO:0030554~adenyl nucleotide binding | 8.81E-02 |
| GOTERM_MF_FAT | GO:0001883~purine nucleoside binding | 9.35E-02 |
| GOTERM_MF_FAT | GO:0001882~nucleoside binding | 9.67E-02 |
| GOTERM_CC_FAT | GO:0044429~mitochondrial part | 1.18E-01 |
| SP_PIR_KEYWORDS | mitochondrion | 1.64E-01 |
| SP_PIR_KEYWORDS | atp-binding | 1.66E-01 |
| GOTERM_MF_FAT | GO:0017076~purine nucleotide binding | 2.01E-01 |
| UP_SEQ_FEATURE | nucleotide phosphate-binding region:ATP | 2.11E-01 |
| GOTERM_MF_FAT | GO:0005524~ATP binding | 2.58E-01 |
| GOTERM_MF_FAT | GO:0032559~adenyl ribonucleotide binding | 2.65E-01 |
| UP_SEQ_FEATURE | binding site:ATP | 2.74E-01 |
| SP_PIR_KEYWORDS | nucleotide-binding | 3.30E-01 |

| | | |
|---|---|---|
| GOTERM_BP_FAT | GO:0016310~phosphorylation | 3.34E-01 |
| UP_SEQ_FEATURE | active site:Proton acceptor | 3.72E-01 |
| GOTERM_MF_FAT | GO:0000166~nucleotide binding | 3.79E-01 |
| GOTERM_CC_FAT | GO:0005829~cytosol | 4.25E-01 |
| GOTERM_MF_FAT | GO:0032555~purine ribonucleotide binding | 4.51E-01 |
| GOTERM_MF_FAT | GO:0032553~ribonucleotide binding | 4.51E-01 |
| GOTERM_BP_FAT | GO:0006793~phosphorus metabolic process | 4.84E-01 |
| GOTERM_BP_FAT | GO:0006796~phosphate metabolic process | 4.84E-01 |
| GOTERM_BP_FAT | GO:0006468~protein amino acid phosphorylation | 6.90E-01 |

## Enriched annotation of drugs:

No significant annotation terms found.

## Drug-induced transcriptional module: PC3-6

## 31 genes, 19 chemicals

## Functional enrichment of genes:

| **Annotation Cluster 1** | **Enrichment Score: 6.414849867963569** | |
|---|---|---|
| | | |
| Category | Term | PValue |
| GOTERM_CC_FAT | GO:0005730~nucleolus | 4.53E-11 |
| GOTERM_CC_FAT | GO:0070013~intracellular organelle lumen | 1.22E-07 |
| GOTERM_CC_FAT | GO:0043233~organelle lumen | 1.58E-07 |
| GOTERM_CC_FAT | GO:0031974~membrane-enclosed lumen | 2.11E-07 |
| GOTERM_CC_FAT | GO:0031981~nuclear lumen | 8.91E-07 |
| GOTERM_CC_FAT | GO:0043232~intracellular non-membrane-bounded organelle | 2.37E-06 |
| GOTERM_CC_FAT | GO:0043228~non-membrane-bounded organelle | 2.37E-06 |
| SP_PIR_KEYWORDS | nucleus | 5.21E-04 |
| | | |
| | | |
| **Annotation Cluster 2** | **Enrichment Score: 2.428816658226607** | |
| | | |
| Category | Term | PValue |
| GOTERM_BP_FAT | GO:0034470~ncRNA processing | 4.62E-04 |
| GOTERM_BP_FAT | GO:0034660~ncRNA metabolic process | 8.04E-04 |
| GOTERM_BP_FAT | GO:0009451~RNA modification | 3.41E-03 |
| GOTERM_BP_FAT | GO:0006396~RNA processing | 3.53E-03 |
| UP_SEQ_FEATURE | active site:Nucleophile | 4.53E-03 |
| GOTERM_BP_FAT | GO:0008033~tRNA processing | 8.44E-03 |
| SP_PIR_KEYWORDS | trna processing | 1.13E-02 |
| GOTERM_BP_FAT | GO:0006399~tRNA metabolic process | 1.92E-02 |

## Enriched annotation of drugs:

| **Annotation type** | **Enrichment term** | **q-value** |
|---|---|---|
| Drug target | SERPINE1;serpin peptidase inhibitor, clade E (nexin, plasminogen activator inhibitor type 1), member 1 | 1.81E-02 |

## Drug-induced transcriptional module: PC3-7

## 64 genes, 23 chemicals

## Functional enrichment of genes:

| **Annotation Cluster 1** | **Enrichment Score: 5.447774442527262** | |
|---|---|---|
| | | |
| Category | Term | PValue |
| SP_PIR_KEYWORDS | chelation | 3.47E-08 |

| | | |
|---|---|---|
| SP_PIR_KEYWORDS | metal-thiolate cluster | 1.60E-07 |
| UP_SEQ_FEATURE | metal ion-binding site:Divalent metal cation; cluster B | 1.61E-07 |
| UP_SEQ_FEATURE | metal ion-binding site:Divalent metal cation; cluster A | 1.61E-07 |
| UP_SEQ_FEATURE | region of interest:Alpha | 1.61E-07 |
| UP_SEQ_FEATURE | region of interest:Beta | 1.61E-07 |
| INTERPRO | IPR000006:Metallothionein, vertebrate | 2.05E-07 |
| INTERPRO | IPR003019:Metallothionein superfamily, eukaryotic | 2.05E-07 |
| INTERPRO | IPR018064:Metallothionein, vertebrate, metal binding site | 2.05E-07 |
| PIR_SUPERFAMILY | PIRSF002564:metallothionein | 2.13E-07 |
| SP_PIR_KEYWORDS | metal binding | 7.43E-07 |
| SP_PIR_KEYWORDS | cadmium | 6.79E-06 |
| GOTERM_MF_FAT | GO:0046870~cadmium ion binding | 1.83E-05 |
| SP_PIR_KEYWORDS | acetylated amino end | 1.08E-03 |
| SP_PIR_KEYWORDS | copper | 1.22E-03 |
| GOTERM_MF_FAT | GO:0005507~copper ion binding | 3.15E-03 |
| SP_PIR_KEYWORDS | acetylation | 9.37E-01 |
| | | |
| | | |
| **Annotation Cluster 2** | **Enrichment Score: 2.300260262849464** | |
| | | |
| Category | Term | PValue |
| GOTERM_BP_FAT | GO:0009611~response to wounding | 6.45E-06 |
| GOTERM_BP_FAT | GO:0042060~wound healing | 1.29E-04 |
| GOTERM_BP_FAT | GO:0001944~vasculature development | 8.62E-04 |
| GOTERM_BP_FAT | GO:0006928~cell motion | 1.37E-03 |
| GOTERM_BP_FAT | GO:0016477~cell migration | 1.84E-03 |
| GOTERM_BP_FAT | GO:0048514~blood vessel morphogenesis | 2.23E-03 |
| GOTERM_BP_FAT | GO:0051674~localization of cell | 2.40E-03 |
| GOTERM_BP_FAT | GO:0048870~cell motility | 2.40E-03 |
| GOTERM_BP_FAT | GO:0001568~blood vessel development | 4.79E-03 |
| GOTERM_BP_FAT | GO:0001525~angiogenesis | 2.83E-02 |
| GOTERM_BP_FAT | GO:0008284~positive regulation of cell proliferation | 4.32E-02 |
| GOTERM_BP_FAT | GO:0051094~positive regulation of developmental process | 4.46E-02 |
| GOTERM_BP_FAT | GO:0001666~response to hypoxia | 1.61E-01 |
| GOTERM_BP_FAT | GO:0070482~response to oxygen levels | 1.75E-01 |
| GOTERM_BP_FAT | GO:0042127~regulation of cell proliferation | 1.83E-01 |

## **Enriched annotation of drugs**:

| **Annotation type** | **Enrichment term** | **q-value** |
|---|---|---|
| Drug target | NR3C1;nuclear receptor subfamily 3, group C, member 1 (glucocorticoid receptor) | 1.03E-04 |
| Drug target | NR3C2;nuclear receptor subfamily 3, group C, member 2 | 8.98E-02 |
| ATC code (2nd level) | D07; Corticosteroids, dermatological preparations | 1.21E-22 |
| ATC code (2nd level) | H02; Corticosteroids for systemic use | 7.46E-08 |
| ATC code (2nd level) | R01; Nasal preparations | 2.70E-07 |
| ATC code (2nd level) | R03; Drugs for obstructive airway diseases | 1.92E-04 |
| ATC code (2nd level) | A07; Antidiarrheals, intestinal anti-inflammatory/anti-infective agents | 1.89E-02 |
| ATC code (2nd level) | C05; Vasoprotectives | 1.93E-02 |
| ATC code (2nd level) | D10; Anti-acne preparations | 2.56E-02 |
| Side effect | striae | 3.90E-16 |
| Side effect | skin atrophy | 2.75E-11 |
| Side effect | hypopigmentation | 1.69E-08 |
| Side effect | miliaria | 2.92E-08 |
| Side effect | hypertrichosis | 7.52E-06 |
| Side effect | folliculitis | 7.52E-06 |

| | | |
|---|---|---|
| Side effect | cataract | 1.58E-05 |
| Side effect | Aseptic necrosis | 6.25E-05 |
| Side effect | ocular infection | 1.80E-04 |
| Side effect | alkalosis | 1.80E-04 |
| Side effect | paraparesis | 1.80E-04 |
| Side effect | nasal septum perforation | 1.80E-04 |
| Side effect | glaucoma | 3.13E-04 |
| Side effect | exophthalmos | 3.98E-04 |
| Side effect | paraplegia | 8.01E-04 |
| Side effect | infection | 2.06E-03 |
| Side effect | pseudotumor cerebri | 3.37E-03 |
| Side effect | latent diabetes | 8.97E-03 |
| Side effect | abscess | 8.97E-03 |
| Side effect | arachnoiditis | 1.05E-02 |
| Side effect | osteoporosis | 1.14E-02 |
| Side effect | pathological fracture | 1.14E-02 |
| Side effect | papilledema | 1.14E-02 |
| Side effect | hypernatremia | 2.57E-02 |
| Side effect | hyperpigmentation | 7.40E-02 |
| Side effect | myopathy | 8.40E-02 |
| Side effect | ulcer | 9.40E-02 |
| Side effect | cardiomegaly | 9.40E-02 |

## Drug-induced transcriptional module: PC3-8

## 66 genes, 19 chemicals

## Functional enrichment of genes:

| **Annotation Cluster 1** | **Enrichment Score: 42.14862318163935** | |
|---|---|---|
| | | |
| Category | Term | PValue |
| GOTERM_BP_FAT | GO:0000279~M phase | 3.52E-48 |
| GOTERM_BP_FAT | GO:0007067~mitosis | 4.03E-45 |
| GOTERM_BP_FAT | GO:0000280~nuclear division | 4.03E-45 |
| GOTERM_BP_FAT | GO:0000087~M phase of mitotic cell cycle | 8.24E-45 |
| GOTERM_BP_FAT | GO:0022403~cell cycle phase | 9.49E-45 |
| GOTERM_BP_FAT | GO:0048285~organelle fission | 2.63E-44 |
| GOTERM_BP_FAT | GO:0000278~mitotic cell cycle | 2.12E-43 |
| SP_PIR_KEYWORDS | cell cycle | 2.82E-42 |
| GOTERM_BP_FAT | GO:0022402~cell cycle process | 5.58E-42 |
| SP_PIR_KEYWORDS | cell division | 1.94E-41 |
| SP_PIR_KEYWORDS | mitosis | 2.20E-41 |
| GOTERM_BP_FAT | GO:0007049~cell cycle | 1.60E-40 |
| GOTERM_BP_FAT | GO:0051301~cell division | 4.38E-34 |
| | | |
| | | |
| **Annotation Cluster 2** | **Enrichment Score: 22.905409533028248** | |
| | | |
| Category | Term | PValue |
| GOTERM_CC_FAT | GO:0005819~spindle | 1.61E-33 |
| GOTERM_CC_FAT | GO:0015630~microtubule cytoskeleton | 3.61E-32 |

| | | |
|---|---|---|
| GOTERM_CC_FAT | GO:0044430~cytoskeletal part | 1.18E-26 |
| GOTERM_CC_FAT | GO:0005856~cytoskeleton | 1.95E-21 |
| GOTERM_CC_FAT | GO:0043228~non-membrane-bounded organelle | 2.51E-18 |
| GOTERM_CC_FAT | GO:0043232~intracellular non-membrane-bounded organelle | 2.51E-18 |
| SP_PIR_KEYWORDS | cytoskeleton | 5.45E-15 |
| | | |
| | | |
| **Annotation Cluster 3** | **Enrichment Score: 15.63184347583849** | |
| | | |
| Category | Term | PValue |
| GOTERM_BP_FAT | GO:0007017~microtubule-based process | 2.10E-25 |
| GOTERM_BP_FAT | GO:0000226~microtubule cytoskeleton organization | 1.53E-17 |
| GOTERM_BP_FAT | GO:0007010~cytoskeleton organization | 2.06E-11 |
| GOTERM_BP_FAT | GO:0007051~spindle organization | 4.49E-11 |

## Enriched annotation of drugs:

No significant annotation terms found.

## Drug-induced transcriptional module: PC3-9

## 39 genes, 17 chemicals

## Functional enrichment of genes:

No significant annotation clusters found.

## Enriched annotation of drugs:

| Annotation type | Enrichment term | q-value |
|---|---|---|
| Drug target | SERPINE1;serpin peptidase inhibitor, clade E (nexin, plasminogen activator inhibitor type 1), member 1 | 1.08E-02 |
| Drug target | LPL;lipoprotein lipase | 1.08E-02 |
| Drug target | PPARG;peroxisome proliferator-activated receptor gamma | 1.08E-02 |
| Drug target | SLCO1B1;solute carrier organic anion transporter family, member 1B1 | 1.81E-02 |
| Drug target | ABCC8;ATP-binding cassette, sub-family C (CFTR/MRP), member 8 | 2.86E-02 |
| ATC code (2nd level) | A10; Drugs used in diabetes | 1.70E-04 |

## Drug-induced transcriptional module: PC3-10

## 38 genes, 12 chemicals

## Functional enrichment of genes:

| **Annotation Cluster 1** | **Enrichment Score: 2.038362156655783** | |
|---|---|---|
| | | |
| Category | Term | PValue |
| INTERPRO | IPR019782:WD40 repeat 2 | 4.20E-03 |
| INTERPRO | IPR017986:WD40 repeat, region | 4.83E-03 |
| INTERPRO | IPR019781:WD40 repeat, subgroup | 5.41E-03 |
| UP_SEQ_FEATURE | repeat:WD 3 | 6.38E-03 |
| UP_SEQ_FEATURE | repeat:WD 1 | 6.90E-03 |
| UP_SEQ_FEATURE | repeat:WD 2 | 6.90E-03 |
| SP_PIR_KEYWORDS | wd repeat | 7.00E-03 |
| INTERPRO | IPR001680:WD40 repeat | 7.40E-03 |
| INTERPRO | IPR019775:WD40 repeat, conserved site | 8.32E-03 |

| | | |
|---|---|---|
| INTERPRO | IPR015943:WD40/YVTN repeat-like | 1.06E-02 |
| SMART | SM00320:WD40 | 1.35E-02 |
| UP_SEQ_FEATURE | repeat:WD 6 | 1.65E-02 |
| UP_SEQ_FEATURE | repeat:WD 5 | 2.64E-02 |
| UP_SEQ_FEATURE | repeat:WD 4 | 3.26E-02 |

## Enriched annotation of drugs:

No significant annotation terms found.

## Drug-induced transcriptional module: PC3-11

### 73 genes, 22 chemicals

Functional enrichment of genes:

| **Annotation Cluster 1** | **Enrichment Score: 5.140678110697808** | |
|---|---|---|
| | | |
| Category | Term | PValue |
| UP_SEQ_FEATURE | glycosylation site:N-linked (GlcNAc...) | 3.03E-09 |
| SP_PIR_KEYWORDS | glycoprotein | 1.56E-08 |
| SP_PIR_KEYWORDS | Secreted | 8.60E-08 |
| SP_PIR_KEYWORDS | signal | 1.01E-06 |
| UP_SEQ_FEATURE | signal peptide | 1.04E-06 |
| GOTERM_CC_FAT | GO:0005576~extracellular region | 3.30E-06 |
| GOTERM_CC_FAT | GO:0044421~extracellular region part | 4.39E-04 |
| SP_PIR_KEYWORDS | disulfide bond | 1.16E-03 |
| UP_SEQ_FEATURE | disulfide bond | 4.98E-03 |
| GOTERM_CC_FAT | GO:0005615~extracellular space | 1.09E-02 |
| | | |
| | | |
| **Annotation Cluster 2** | **Enrichment Score: 2.396383755569229** | |
| | | |
| Category | Term | PValue |
| GOTERM_MF_FAT | GO:0030414~peptidase inhibitor activity | 9.73E-05 |
| GOTERM_MF_FAT | GO:0004866~endopeptidase inhibitor activity | 9.73E-05 |
| GOTERM_BP_FAT | GO:0042060~wound healing | 1.44E-03 |
| KEGG_PATHWAY | hsa04610:Complement and coagulation cascades | 2.02E-03 |
| GOTERM_BP_FAT | GO:0050878~regulation of body fluid levels | 2.81E-03 |
| GOTERM_MF_FAT | GO:0004867~serine-type endopeptidase inhibitor activity | 2.82E-03 |
| GOTERM_MF_FAT | GO:0004857~enzyme inhibitor activity | 4.55E-03 |
| SP_PIR_KEYWORDS | serine proteinase inhibitor | 5.76E-03 |
| INTERPRO | IPR000215:Protease inhibitor I4, serpin | 6.31E-03 |
| SMART | SM00093:SERPIN | 7.02E-03 |
| GOTERM_BP_FAT | GO:0050817~coagulation | 8.81E-03 |
| GOTERM_BP_FAT | GO:0007596~blood coagulation | 8.81E-03 |
| PIR_SUPERFAMILY | PIRSF001630:serpin | 9.34E-03 |
| GOTERM_BP_FAT | GO:0007599~hemostasis | 1.08E-02 |
| UP_SEQ_FEATURE | site:Reactive bond | 1.17E-02 |
| SP_PIR_KEYWORDS | Serine protease inhibitor | 2.22E-02 |
| SP_PIR_KEYWORDS | protease inhibitor | 3.55E-02 |

## Enriched annotation of drugs:

| Annotation type | Enrichment term | q-value |
|---|---|---|
| Drug target | ADRB2;adrenergic, beta-2-, receptor, surface | 6.55E-08 |
| Drug target | ADRB1;adrenergic, beta-1-, receptor | 2.60E-04 |
| Drug target | ADRB3;adrenergic, beta-3-, receptor | 3.75E-03 |
| Drug target | ADCY10;adenylate cyclase 10 (soluble) | 1.81E-02 |
| ATC code (2nd level) | R03; Drugs for obstructive airway diseases | 3.19E-06 |
| ATC code (2nd level) | C01; Cardiac therapy | 7.91E-02 |
| Side effect | tachycardia | 7.88E-02 |

## Drug-induced transcriptional module: PC3-12

### 89 genes, 35 chemicals

Functional enrichment of genes:

| **Annotation Cluster 1** | **Enrichment Score: 4.061203059670271** | |
|---|---|---|
| | | |
| Category | Term | PValue |
| INTERPRO | IPR001909:Krueppel-associated box | 5.92E-08 |
| UP_SEQ_FEATURE | domain:KRAB | 5.99E-08 |
| UP_SEQ_FEATURE | zinc finger region:C2H2-type 6 | 1.03E-07 |
| SMART | SM00349:KRAB | 2.27E-07 |
| UP_SEQ_FEATURE | zinc finger region:C2H2-type 11 | 3.74E-07 |
| UP_SEQ_FEATURE | zinc finger region:C2H2-type 7 | 4.26E-07 |
| UP_SEQ_FEATURE | zinc finger region:C2H2-type 5 | 4.80E-07 |
| INTERPRO | IPR015880:Zinc finger, C2H2-like | 8.24E-07 |
| INTERPRO | IPR013087:Zinc finger, C2H2-type/integrase, DNA-binding | 8.80E-07 |
| UP_SEQ_FEATURE | zinc finger region:C2H2-type 10 | 1.11E-06 |
| UP_SEQ_FEATURE | zinc finger region:C2H2-type 8 | 1.15E-06 |
| UP_SEQ_FEATURE | zinc finger region:C2H2-type 12 | 1.40E-06 |
| UP_SEQ_FEATURE | zinc finger region:C2H2-type 3 | 1.63E-06 |
| INTERPRO | IPR007087:Zinc finger, C2H2-type | 3.97E-06 |
| SMART | SM00355:ZnF_C2H2 | 4.03E-06 |
| UP_SEQ_FEATURE | zinc finger region:C2H2-type 13 | 4.71E-06 |
| UP_SEQ_FEATURE | zinc finger region:C2H2-type 4 | 5.49E-06 |
| UP_SEQ_FEATURE | zinc finger region:C2H2-type 2 | 7.69E-06 |
| UP_SEQ_FEATURE | zinc finger region:C2H2-type 17 | 1.13E-05 |
| UP_SEQ_FEATURE | zinc finger region:C2H2-type 9 | 3.12E-05 |
| UP_SEQ_FEATURE | zinc finger region:C2H2-type 16 | 3.80E-05 |
| SP_PIR_KEYWORDS | zinc-finger | 4.54E-05 |
| UP_SEQ_FEATURE | zinc finger region:C2H2-type 15 | 8.93E-05 |
| GOTERM_MF_FAT | GO:0003677~DNA binding | 1.70E-04 |
| PIR_SUPERFAMILY | PIRSF005559:zinc finger protein ZFP-36 | 2.11E-04 |
| GOTERM_MF_FAT | GO:0008270~zinc ion binding | 5.35E-04 |
| SP_PIR_KEYWORDS | zinc | 5.93E-04 |
| UP_SEQ_FEATURE | zinc finger region:C2H2-type 1 | 7.50E-04 |
| SP_PIR_KEYWORDS | dna-binding | 1.17E-03 |
| GOTERM_BP_FAT | GO:0006350~transcription | 1.29E-03 |
| UP_SEQ_FEATURE | zinc finger region:C2H2-type 18 | 1.49E-03 |
| SP_PIR_KEYWORDS | metal-binding | 1.77E-03 |
| GOTERM_BP_FAT | GO:0006355~regulation of transcription, DNA-dependent | 2.88E-03 |
| GOTERM_MF_FAT | GO:0046914~transition metal ion binding | 3.50E-03 |
| GOTERM_BP_FAT | GO:0051252~regulation of RNA metabolic process | 3.93E-03 |
| GOTERM_BP_FAT | GO:0045449~regulation of transcription | 5.90E-03 |
| SP_PIR_KEYWORDS | Transcription | 9.16E-03 |
| GOTERM_MF_FAT | GO:0046872~metal ion binding | 9.48E-03 |
| GOTERM_MF_FAT | GO:0043169~cation binding | 1.05E-02 |

| | | |
|---|---|---|
| GOTERM_MF_FAT | GO:0043167~ion binding | 1.25E-02 |
| UP_SEQ_FEATURE | zinc finger region:C2H2-type 14 | 1.54E-02 |
| SP_PIR_KEYWORDS | zinc finger | 1.73E-02 |
| SP_PIR_KEYWORDS | transcription regulation | 2.84E-02 |
| UP_SEQ_FEATURE | zinc finger region:C2H2-type 1; degenerate | 3.28E-02 |
| SP_PIR_KEYWORDS | nucleus | 5.00E-02 |
| SP_PIR_KEYWORDS | DNA binding | 5.84E-02 |
| | | |
| | | |
| **Annotation Cluster 2** | **Enrichment Score: 1.3941460558573309** | |
| | | |
| Category | Term | PValue |
| GOTERM_BP_FAT | GO:0006310~DNA recombination | 3.29E-04 |
| GOTERM_BP_FAT | GO:0006259~DNA metabolic process | 6.97E-03 |
| GOTERM_BP_FAT | GO:0006281~DNA repair | 2.41E-02 |
| SP_PIR_KEYWORDS | dna repair | 4.15E-02 |
| GOTERM_BP_FAT | GO:0010212~response to ionizing radiation | 4.59E-02 |
| SP_PIR_KEYWORDS | DNA damage | 5.41E-02 |
| GOTERM_BP_FAT | GO:0006974~response to DNA damage stimulus | 7.36E-02 |
| GOTERM_BP_FAT | GO:0033554~cellular response to stress | 2.19E-01 |
| GOTERM_BP_FAT | GO:0009314~response to radiation | 2.46E-01 |
| GOTERM_BP_FAT | GO:0009628~response to abiotic stimulus | 5.07E-01 |

## Enriched annotation of drugs:

No significant annotation terms found.

## Drug-induced transcriptional module: PC3-13

## 26 genes, 8 chemicals

Functional enrichment of genes:

| **Annotation Cluster 1** | **Enrichment Score: 1.8199048607534811** | |
|---|---|---|
| | | |
| Category | Term | PValue |
| INTERPRO | IPR000504:RNA recognition motif, RNP-1 | 5.78E-03 |
| INTERPRO | IPR012677:Nucleotide-binding, alpha-beta plait | 6.13E-03 |
| GOTERM_MF_FAT | GO:0003723~RNA binding | 7.32E-03 |
| GOTERM_BP_FAT | GO:0006396~RNA processing | 8.88E-03 |
| SMART | SM00360:RRM | 9.24E-03 |
| UP_SEQ_FEATURE | domain:RRM | 1.99E-02 |
| SP_PIR_KEYWORDS | rna-binding | 2.15E-02 |
| GOTERM_MF_FAT | GO:0000166~nucleotide binding | 3.03E-01 |
| | | |
| | | |
| **Annotation Cluster 2** | **Enrichment Score: 1.431277081641486** | |
| | | |
| Category | Term | PValue |
| GOTERM_MF_FAT | GO:0003723~RNA binding | 7.32E-03 |
| GOTERM_BP_FAT | GO:0006396~RNA processing | 8.88E-03 |
| GOTERM_BP_FAT | GO:0006397~mRNA processing | 4.11E-02 |
| GOTERM_BP_FAT | GO:0016071~mRNA metabolic process | 5.62E-02 |
| SP_PIR_KEYWORDS | mrna processing | 1.08E-01 |
| GOTERM_BP_FAT | GO:0008380~RNA splicing | 1.59E-01 |

## Enriched annotation of drugs:

| **Annotation type** | **Enrichment term** | **q-value** |
|---|---|---|

| | | |
|---|---|---|
| Drug target | CYP1B1;cytochrome P450, family 1, subfamily B, polypeptide 1 | 8.60E-02 |
| Annotation type | Enrichment term | q-value |
| Drug target | CYP1B1;cytochrome P450, family 1, subfamily B, polypeptide 1 | 8.60E-02 |

## Drug-induced transcriptional module: PC3-14

## 24 genes, 11 chemicals

## Functional enrichment of genes:

| **Annotation Cluster 1** | **Enrichment Score: 9.955952566887282** | |
|---|---|---|
| | | |
| Category | Term | PValue |
| SP_PIR_KEYWORDS | lipid synthesis | 1.95E-22 |
| SP_PIR_KEYWORDS | Steroid biosynthesis | 3.15E-22 |
| GOTERM_BP_FAT | GO:0008202~steroid metabolic process | 1.70E-21 |
| GOTERM_BP_FAT | GO:0016125~sterol metabolic process | 1.03E-20 |
| GOTERM_BP_FAT | GO:0016126~sterol biosynthetic process | 7.83E-20 |
| GOTERM_BP_FAT | GO:0006694~steroid biosynthetic process | 3.52E-19 |
| GOTERM_BP_FAT | GO:0008610~lipid biosynthetic process | 5.54E-18 |
| GOTERM_BP_FAT | GO:0008203~cholesterol metabolic process | 7.35E-17 |
| SP_PIR_KEYWORDS | sterol biosynthesis | 1.88E-16 |
| KEGG_PATHWAY | hsa00100:Steroid biosynthesis | 1.05E-15 |
| GOTERM_BP_FAT | GO:0006695~cholesterol biosynthetic process | 1.23E-13 |
| SP_PIR_KEYWORDS | Cholesterol biosynthesis | 3.05E-12 |
| SP_PIR_KEYWORDS | oxidoreductase | 7.79E-12 |
| GOTERM_BP_FAT | GO:0055114~oxidation reduction | 5.89E-11 |
| SP_PIR_KEYWORDS | nadp | 2.92E-09 |
| GOTERM_CC_FAT | GO:0005783~endoplasmic reticulum | 3.15E-08 |
| GOTERM_CC_FAT | GO:0005789~endoplasmic reticulum membrane | 7.34E-07 |
| SP_PIR_KEYWORDS | endoplasmic reticulum | 1.10E-06 |
| GOTERM_CC_FAT | GO:0042175~nuclear envelope-endoplasmic reticulum network | 1.11E-06 |
| GOTERM_CC_FAT | GO:0012505~endomembrane system | 1.63E-06 |
| GOTERM_CC_FAT | GO:0044432~endoplasmic reticulum part | 4.11E-06 |
| GOTERM_BP_FAT | GO:0008299~isoprenoid biosynthetic process | 7.37E-06 |
| UP_SEQ_FEATURE | transmembrane region | 3.03E-05 |
| SP_PIR_KEYWORDS | transmembrane | 3.20E-05 |
| GOTERM_BP_FAT | GO:0006720~isoprenoid metabolic process | 5.15E-05 |
| KEGG_PATHWAY | hsa00900:Terpenoid backbone biosynthesis | 7.02E-05 |
| SP_PIR_KEYWORDS | membrane | 9.87E-05 |
| SP_PIR_KEYWORDS | Isoprene biosynthesis | 1.39E-04 |
| GOTERM_CC_FAT | GO:0031090~organelle membrane | 2.32E-04 |
| GOTERM_CC_FAT | GO:0016021~integral to membrane | 5.40E-04 |
| GOTERM_CC_FAT | GO:0031224~intrinsic to membrane | 7.87E-04 |
| SP_PIR_KEYWORDS | magnesium | 3.22E-02 |
| | | |
| | | |
| **Annotation Cluster 2** | **Enrichment Score: 3.378651257033207** | |
| | | |
| Category | Term | PValue |
| UP_SEQ_FEATURE | short sequence motif:Histidine box-2 | 5.63E-07 |
| UP_SEQ_FEATURE | short sequence motif:Histidine box-1 | 5.63E-07 |
| UP_SEQ_FEATURE | short sequence motif:Histidine box-3 | 5.63E-07 |
| GOTERM_BP_FAT | GO:0006633~fatty acid biosynthetic process | 1.07E-05 |
| GOTERM_MF_FAT | GO:0016717~oxidoreductase activity, acting on paired donors, with oxidation of a pair of donors resulting in the reduction of molecular oxygen to two molecules of water | 1.20E-04 |

| | | |
|---|---|---|
| GOTERM_BP_FAT | GO:0016053~organic acid biosynthetic process | 2.01E-04 |
| GOTERM_BP_FAT | GO:0046394~carboxylic acid biosynthetic process | 2.01E-04 |
| GOTERM_BP_FAT | GO:0006631~fatty acid metabolic process | 3.15E-04 |
| SP_PIR_KEYWORDS | iron | 7.42E-04 |
| GOTERM_MF_FAT | GO:0005506~iron ion binding | 1.32E-03 |
| SP_PIR_KEYWORDS | Fatty acid biosynthesis | 3.99E-03 |
| GOTERM_MF_FAT | GO:0046872~metal ion binding | 9.19E-02 |
| GOTERM_MF_FAT | GO:0043169~cation binding | 9.58E-02 |
| GOTERM_MF_FAT | GO:0043167~ion binding | 1.02E-01 |
| GOTERM_MF_FAT | GO:0046914~transition metal ion binding | 2.02E-01 |
| | | |
| | | |
| **Annotation Cluster 3** | **Enrichment Score: 2.3001587099994376** | |
| | | |
| Category | Term | PValue |
| GOTERM_CC_FAT | GO:0005792~microsome | 8.72E-04 |
| GOTERM_CC_FAT | GO:0042598~vesicular fraction | 9.45E-04 |
| GOTERM_CC_FAT | GO:0000267~cell fraction | 1.43E-02 |
| GOTERM_CC_FAT | GO:0005624~membrane fraction | 1.49E-02 |
| GOTERM_CC_FAT | GO:0005626~insoluble fraction | 1.79E-02 |

## Enriched annotation of drugs:

No significant annotation terms found.

## Drug-induced transcriptional module: PC3-15

## 56 genes, 14 chemicals

## Functional enrichment of genes:

| | | |
|---|---|---|
| **Annotation Cluster 1** | **Enrichment Score: 15.612089171497864** | |
| | | |
| Category | Term | PValue |
| GOTERM_BP_FAT | GO:0006986~response to unfolded protein | 4.20E-20 |
| SP_PIR_KEYWORDS | stress response | 2.49E-19 |
| GOTERM_BP_FAT | GO:0051789~response to protein stimulus | 7.64E-17 |
| GOTERM_BP_FAT | GO:0010033~response to organic substance | 4.47E-09 |
| | | |
| | | |
| **Annotation Cluster 2** | **Enrichment Score: 15.384829269004648** | |
| | | |
| Category | Term | PValue |
| SP_PIR_KEYWORDS | Chaperone | 2.02E-19 |
| GOTERM_BP_FAT | GO:0006457~protein folding | 1.94E-18 |
| GOTERM_MF_FAT | GO:0051082~unfolded protein binding | 1.79E-10 |
| | | |
| | | |
| **Annotation Cluster 3** | **Enrichment Score: 7.148353129480604** | |
| | | |
| Category | Term | PValue |
| SP_PIR_KEYWORDS | molecular chaperone | 7.99E-11 |
| SP_PIR_KEYWORDS | heat shock | 9.52E-07 |
| SP_PIR_KEYWORDS | stress-induced protein | 4.72E-06 |
| | | |
| | | |
| **Annotation Cluster 4** | **Enrichment Score: 3.9541477903260938** | |
| | | |
| Category | Term | PValue |
| INTERPRO | IPR013126:Heat shock protein 70 | 3.60E-05 |
| INTERPRO | IPR001023:Heat shock protein Hsp70 | 3.60E-05 |
| INTERPRO | IPR018181:Heat shock protein 70, conserved site | 4.78E-05 |

| | | |
|---|---|---|
| KEGG_PATHWAY | hsa04612:Antigen processing and presentation | 2.46E-03 |

**Enriched annotation of drugs:**

No significant annotation terms found.

**Drug-induced transcriptional module: PC3-16**

**30 genes, 6 chemicals**

**Functional enrichment of genes:**

No significant annotation clusters found.

**Enriched annotation of drugs:**

No significant annotation terms found.

**Drug-induced transcriptional module: PC3-17**

**55 genes, 9 chemicals**

**Functional enrichment of genes:**

| **Annotation Cluster 1** | **Enrichment Score: 1.511512598128385** | |
|---|---|---|
| | | |
| Category | Term | PValue |
| SP_PIR_KEYWORDS | dna-binding | 8.23E-03 |
| SP_PIR_KEYWORDS | transcription regulation | 2.55E-02 |
| GOTERM_MF_FAT | GO:0003677~DNA binding | 2.78E-02 |
| SP_PIR_KEYWORDS | Transcription | 3.30E-02 |
| GOTERM_BP_FAT | GO:0006350~transcription | 4.67E-02 |
| GOTERM_BP_FAT | GO:0045449~regulation of transcription | 4.83E-02 |
| GOTERM_MF_FAT | GO:0030528~transcription regulator activity | 6.06E-02 |
| | | |
| | | |
| **Annotation Cluster 2** | **Enrichment Score: 1.3096019272909456** | |
| | | |
| Category | Term | PValue |
| GOTERM_BP_FAT | GO:0051169~nuclear transport | 9.27E-03 |
| GOTERM_BP_FAT | GO:0006913~nucleocytoplasmic transport | 9.27E-03 |
| GOTERM_BP_FAT | GO:0006406~mRNA export from nucleus | 1.31E-02 |
| GOTERM_BP_FAT | GO:0006405~RNA export from nucleus | 2.03E-02 |
| GOTERM_BP_FAT | GO:0051168~nuclear export | 4.16E-02 |
| GOTERM_BP_FAT | GO:0051028~mRNA transport | 8.13E-02 |
| GOTERM_BP_FAT | GO:0050658~RNA transport | 9.91E-02 |
| GOTERM_BP_FAT | GO:0051236~establishment of RNA localization | 9.91E-02 |
| GOTERM_BP_FAT | GO:0050657~nucleic acid transport | 9.91E-02 |
| GOTERM_BP_FAT | GO:0006403~RNA localization | 1.03E-01 |
| GOTERM_BP_FAT | GO:0015931~nucleobase, nucleoside, nucleotide and nucleic acid transport | 1.14E-01 |
| GOTERM_BP_FAT | GO:0046907~intracellular transport | 2.18E-01 |

**Enriched annotation of drugs:**

No significant annotation terms found.

**Drug-induced transcriptional module: PC3-18**

**45 genes, 49 chemicals**

**Functional enrichment of genes:**

No significant annotation clusters found.

**Enriched annotation of drugs:**

No significant annotation terms found.

## Drug-induced transcriptional module: PC3-19

**62 genes, 100 chemicals**

**Functional enrichment of genes:**

| Annotation Cluster 1 | Enrichment Score: 3.3522062191313164 | |
|---|---|---|
| | | |
| Category | Term | PValue |
| UP_SEQ_FEATURE | glycosylation site:N-linked (GlcNAc...) | 9.35E-05 |
| UP_SEQ_FEATURE | transmembrane region | 1.24E-04 |
| SP_PIR_KEYWORDS | transmembrane | 1.35E-04 |
| SP_PIR_KEYWORDS | membrane | 2.22E-04 |
| SP_PIR_KEYWORDS | glycoprotein | 2.41E-04 |
| UP_SEQ_FEATURE | topological domain:Extracellular | 6.13E-04 |
| UP_SEQ_FEATURE | topological domain:Cytoplasmic | 1.23E-03 |
| GOTERM_CC_FAT | GO:0016021~integral to membrane | 2.53E-03 |
| GOTERM_CC_FAT | GO:0031224~intrinsic to membrane | 4.21E-03 |
| | | |
| | | |
| **Annotation Cluster 2** | **Enrichment Score: 2.689741599432085** | |
| | | |
| Category | Term | PValue |
| UP_SEQ_FEATURE | glycosylation site:N-linked (GlcNAc...) | 9.35E-05 |
| SP_PIR_KEYWORDS | signal | 2.21E-04 |
| UP_SEQ_FEATURE | signal peptide | 2.25E-04 |
| SP_PIR_KEYWORDS | glycoprotein | 2.41E-04 |
| SP_PIR_KEYWORDS | disulfide bond | 2.93E-03 |
| SP_PIR_KEYWORDS | Secreted | 1.94E-02 |
| UP_SEQ_FEATURE | disulfide bond | 3.27E-02 |
| GOTERM_CC_FAT | GO:0005576~extracellular region | 1.46E-01 |
| | | |
| | | |
| **Annotation Cluster 3** | **Enrichment Score: 2.2084950363959837** | |
| | | |
| Category | Term | PValue |
| UP_SEQ_FEATURE | topological domain:Extracellular | 6.13E-04 |
| UP_SEQ_FEATURE | topological domain:Cytoplasmic | 1.23E-03 |
| GOTERM_CC_FAT | GO:0005886~plasma membrane | 7.77E-03 |
| GOTERM_CC_FAT | GO:0005887~integral to plasma membrane | 1.06E-02 |
| GOTERM_CC_FAT | GO:0031226~intrinsic to plasma membrane | 1.17E-02 |
| GOTERM_CC_FAT | GO:0044459~plasma membrane part | 7.69E-02 |

**Enriched annotation of drugs:**

| Annotation type | Enrichment term | q-value |
|---|---|---|
| Side effect | leukemia | 9.90E-02 |

## Drug-induced transcriptional module: PC3-20

**27 genes, 326 chemicals**

## Functional enrichment of genes:

| **Annotation Cluster 1** | **Enrichment Score: 1.581926594998705** | |
|---|---|---|
| | | |
| Category | Term | PValue |
| GOTERM_BP_FAT | GO:0015031~protein transport | 3.24E-03 |
| GOTERM_BP_FAT | GO:0045184~establishment of protein localization | 3.40E-03 |
| GOTERM_BP_FAT | GO:0008104~protein localization | 5.96E-03 |
| GOTERM_CC_FAT | GO:0019898~extrinsic to membrane | 1.15E-02 |
| SP_PIR_KEYWORDS | er-golgi transport | 1.61E-02 |
| SP_PIR_KEYWORDS | protein transport | 1.67E-02 |
| GOTERM_CC_FAT | GO:0044431~Golgi apparatus part | 2.03E-02 |
| GOTERM_BP_FAT | GO:0006886~intracellular protein transport | 4.61E-02 |
| GOTERM_BP_FAT | GO:0046907~intracellular transport | 5.05E-02 |
| GOTERM_BP_FAT | GO:0034613~cellular protein localization | 5.77E-02 |
| GOTERM_BP_FAT | GO:0070727~cellular macromolecule localization | 5.91E-02 |
| GOTERM_CC_FAT | GO:0005794~Golgi apparatus | 7.30E-02 |
| SP_PIR_KEYWORDS | golgi apparatus | 8.68E-02 |
| GOTERM_BP_FAT | GO:0016192~vesicle-mediated transport | 9.49E-02 |
| SP_PIR_KEYWORDS | transport | 9.54E-02 |
| | | |
| | | |
| **Annotation Cluster 2** | **Enrichment Score: 1.3257383877783788** | |
| | | |
| Category | Term | PValue |
| GOTERM_CC_FAT | GO:0005783~endoplasmic reticulum | 5.02E-03 |
| SP_PIR_KEYWORDS | endoplasmic reticulum | 8.36E-03 |
| GOTERM_CC_FAT | GO:0012505~endomembrane system | 6.19E-02 |
| GOTERM_CC_FAT | GO:0005789~endoplasmic reticulum membrane | 9.07E-02 |
| GOTERM_CC_FAT | GO:0042175~nuclear envelope-endoplasmic reticulum network | 1.01E-01 |
| GOTERM_CC_FAT | GO:0044432~endoplasmic reticulum part | 1.39E-01 |
| GOTERM_CC_FAT | GO:0031090~organelle membrane | 1.59E-01 |

## Enriched annotation of drugs:

| **Annotation type** | **Enrichment term** | **q-value** |
|---|---|---|
| Side effect | paralytic ileus | 7.29E-03 |

## Drug-induced transcriptional module: PC3-21

## 21 genes, 16 chemicals

## Functional enrichment of genes:

No significant annotation clusters found.

## Enriched annotation of drugs:

No significant annotation terms found.

## Drug-induced transcriptional module: PC3-22

## 110 genes, 11 chemicals

## Functional enrichment of genes:

| **Annotation Cluster 1** | **Enrichment Score: 1.7763333918367747** | |
|---|---|---|
| | | |
| Category | Term | PValue |

| | | |
|---|---|---|
| GOTERM_BP_FAT | GO:0034613~cellular protein localization | 9.93E-04 |
| GOTERM_BP_FAT | GO:0070727~cellular macromolecule localization | 1.07E-03 |
| GOTERM_BP_FAT | GO:0006886~intracellular protein transport | 1.76E-03 |
| GOTERM_BP_FAT | GO:0046907~intracellular transport | 5.19E-03 |
| GOTERM_BP_FAT | GO:0015031~protein transport | 2.38E-02 |
| GOTERM_BP_FAT | GO:0045184~establishment of protein localization | 2.54E-02 |
| GOTERM_BP_FAT | GO:0008104~protein localization | 2.57E-02 |
| GOTERM_BP_FAT | GO:0006605~protein targeting | 4.36E-02 |
| SP_PIR_KEYWORDS | protein transport | 4.24E-01 |
| SP_PIR_KEYWORDS | transport | 6.16E-01 |
| | | |
| | | |
| **Annotation Cluster 2** | **Enrichment Score: 1.641869601677667** | |
| | | |
| Category | Term | PValue |
| INTERPRO | IPR003081:Glutathione S-transferase, Mu class | 8.85E-04 |
| PIR_SUPERFAMILY | PIRSF000503:glutathione transferase | 6.40E-03 |
| UP_SEQ_FEATURE | domain:GST N-terminal | 1.05E-02 |
| GOTERM_MF_FAT | GO:0004364~glutathione transferase activity | 1.11E-02 |
| INTERPRO | IPR004045:Glutathione S-transferase, N-terminal | 1.24E-02 |
| INTERPRO | IPR004046:Glutathione S-transferase, C-terminal | 1.61E-02 |
| INTERPRO | IPR010987:Glutathione S-transferase, C-terminal-like | 2.02E-02 |
| INTERPRO | IPR017933:Glutathione S-transferase/chloride channel, C-terminal | 2.47E-02 |
| UP_SEQ_FEATURE | domain:GST C-terminal | 2.90E-02 |
| KEGG_PATHWAY | hsa00982:Drug metabolism | 6.42E-02 |
| KEGG_PATHWAY | hsa00980:Metabolism of xenobiotics by cytochrome P450 | 6.80E-02 |
| KEGG_PATHWAY | hsa00480:Glutathione metabolism | 7.95E-02 |
| GOTERM_MF_FAT | GO:0016765~transferase activity, transferring alkyl or aryl (other than methyl) groups | 8.57E-02 |
| INTERPRO | IPR012335:Thioredoxin fold | 1.82E-01 |

## Enriched annotation of drugs:

No significant annotation terms found.

## <u>Drug-induced transcriptional module: PC3-23</u>

## 158 genes, 8 chemicals

## Functional enrichment of genes:

| **Annotation Cluster 1** | **Enrichment Score: 2.2696257602797214** | |
|---|---|---|
| | | |
| Category | Term | PValue |
| GOTERM_CC_FAT | GO:0005783~endoplasmic reticulum | 1.73E-03 |
| SP_PIR_KEYWORDS | endoplasmic reticulum | 3.03E-03 |
| GOTERM_CC_FAT | GO:0044432~endoplasmic reticulum part | 2.96E-02 |
| | | |
| | | |
| Annotation Cluster 2 | Enrichment Score: 1.544776839339515 | |
| | | |
| Category | Term | PValue |
| GOTERM_MF_FAT | GO:0000287~magnesium ion binding | 1.19E-02 |
| UP_SEQ_FEATURE | metal ion-binding site:Magnesium | 3.84E-02 |
| SP_PIR_KEYWORDS | magnesium | 5.06E-02 |

## Enriched annotation of drugs:

No significant annotation terms found.

## Drug-induced transcriptional module: PC3-24

### 81 genes, 11 chemicals

### Functional enrichment of genes:

| **Annotation Cluster 1** | **Enrichment Score: 1.5499361777406624** | |
|---|---|---|
| | | |
| Category | Term | PValue |
| GOTERM_BP_FAT | GO:0005976~polysaccharide metabolic process | 3.12E-03 |
| GOTERM_BP_FAT | GO:0000271~polysaccharide biosynthetic process | 4.48E-02 |
| GOTERM_BP_FAT | GO:0016051~carbohydrate biosynthetic process | 1.61E-01 |
| | | |
| | | |
| **Annotation Cluster 2** | **Enrichment Score: 1.5166736240094236** | |
| | | |
| Category | Term | PValue |
| GOTERM_BP_FAT | GO:0001568~blood vessel development | 1.18E-02 |
| GOTERM_BP_FAT | GO:0001944~vasculature development | 1.29E-02 |
| GOTERM_BP_FAT | GO:0001570~vasculogenesis | 2.05E-02 |
| GOTERM_BP_FAT | GO:0040007~growth | 7.75E-02 |
| GOTERM_BP_FAT | GO:0048514~blood vessel morphogenesis | 1.08E-01 |
| | | |
| | | |
| **Annotation Cluster 3** | **Enrichment Score: 1.5155585236409688** | |
| | | |
| Category | Term | PValue |
| GOTERM_BP_FAT | GO:0005976~polysaccharide metabolic process | 3.12E-03 |
| GOTERM_BP_FAT | GO:0044275~cellular carbohydrate catabolic process | 1.37E-02 |
| GOTERM_BP_FAT | GO:0006006~glucose metabolic process | 1.57E-02 |
| GOTERM_BP_FAT | GO:0016052~carbohydrate catabolic process | 2.19E-02 |
| GOTERM_BP_FAT | GO:0044042~glucan metabolic process | 2.22E-02 |
| GOTERM_BP_FAT | GO:0005977~glycogen metabolic process | 2.22E-02 |
| GOTERM_BP_FAT | GO:0006073~cellular glucan metabolic process | 2.22E-02 |
| GOTERM_BP_FAT | GO:0006112~energy reserve metabolic process | 2.97E-02 |
| GOTERM_BP_FAT | GO:0019318~hexose metabolic process | 3.42E-02 |
| GOTERM_BP_FAT | GO:0044264~cellular polysaccharide metabolic process | 4.71E-02 |
| GOTERM_BP_FAT | GO:0005996~monosaccharide metabolic process | 5.25E-02 |
| GOTERM_BP_FAT | GO:0006091~generation of precursor metabolites and energy | 1.75E-01 |
| GOTERM_BP_FAT | GO:0015980~energy derivation by oxidation of organic compounds | 2.81E-01 |

### Enriched annotation of drugs:

No significant annotation terms found.

## Drug-induced transcriptional module: PC3-25

### 81 genes, 9 chemicals

### Functional enrichment of genes:

| **Annotation Cluster 1** | **Enrichment Score: 1.5011907623187715** | |
|---|---|---|
| | | |
| Category | Term | PValue |
| SMART | SM00382:AAA | 5.80E-03 |
| INTERPRO | IPR003593:ATPase, AAA+ type, core | 1.19E-02 |
| INTERPRO | IPR003959:ATPase, AAA-type, core | 4.85E-02 |
| UP_SEQ_FEATURE | nucleotide phosphate-binding region:ATP | 2.95E-01 |

### Enriched annotation of drugs:

No significant annotation terms found.

## Drug-induced transcriptional module: PC3-26

### 22 genes, 197 chemicals

### Functional enrichment of genes:

No significant annotation clusters found.

### Enriched annotation of drugs:

| Annotation type | Enrichment term | q-value |
|---|---|---|
| Drug target | DRD4;dopamine receptor D4 | 3.95E-02 |
| Drug target | DRD2;dopamine receptor D2 | 7.15E-02 |
| Drug target | DRD3;dopamine receptor D3 | 7.53E-02 |

## Drug-induced transcriptional module: PC3-27

### 224 genes, 15 chemicals

### Functional enrichment of genes:

| **Annotation Cluster 1** | **Enrichment Score: 4.962121750628933** | |
|---|---|---|
| | | |
| Category | Term | PValue |
| SP_PIR_KEYWORDS | disulfide bond | 2.33E-08 |
| UP_SEQ_FEATURE | disulfide bond | 3.62E-08 |
| UP_SEQ_FEATURE | topological domain:Extracellular | 6.65E-08 |
| UP_SEQ_FEATURE | glycosylation site:N-linked (GlcNAc...) | 5.94E-07 |
| SP_PIR_KEYWORDS | glycoprotein | 1.16E-06 |
| GOTERM_CC_FAT | GO:0031224~intrinsic to membrane | 1.81E-06 |
| GOTERM_CC_FAT | GO:0016021~integral to membrane | 2.02E-06 |
| GOTERM_CC_FAT | GO:0005887~integral to plasma membrane | 6.06E-06 |
| GOTERM_CC_FAT | GO:0031226~intrinsic to plasma membrane | 8.23E-06 |
| UP_SEQ_FEATURE | topological domain:Cytoplasmic | 8.96E-06 |
| SP_PIR_KEYWORDS | transmembrane | 9.01E-06 |
| GOTERM_CC_FAT | GO:0005576~extracellular region | 1.23E-05 |
| UP_SEQ_FEATURE | transmembrane region | 1.50E-05 |
| SP_PIR_KEYWORDS | signal | 2.20E-05 |
| UP_SEQ_FEATURE | signal peptide | 2.27E-05 |
| GOTERM_CC_FAT | GO:0005886~plasma membrane | 2.61E-05 |
| SP_PIR_KEYWORDS | Secreted | 4.60E-05 |
| SP_PIR_KEYWORDS | membrane | 1.06E-03 |
| GOTERM_CC_FAT | GO:0044459~plasma membrane part | 1.14E-03 |
| GOTERM_CC_FAT | GO:0005615~extracellular space | 7.50E-03 |
| GOTERM_CC_FAT | GO:0044421~extracellular region part | 1.10E-02 |
| | | |
| | | |
| **Annotation Cluster 2** | **Enrichment Score: 3.1245908962135593** | |
| | | |
| Category | Term | PValue |
| GOTERM_BP_FAT | GO:0007155~cell adhesion | 1.62E-04 |
| GOTERM_BP_FAT | GO:0022610~biological adhesion | 1.62E-04 |
| SP_PIR_KEYWORDS | cell adhesion | 2.70E-04 |
| GOTERM_BP_FAT | GO:0016337~cell-cell adhesion | 4.47E-02 |
| | | |
| | | |
| **Annotation Cluster 3** | **Enrichment Score: 2.769067857679384** | |
| | | |

| Category | Term | PValue |
|---|---|---|
| SP_PIR_KEYWORDS | Lectin | 3.08E-04 |
| UP_SEQ_FEATURE | domain:C-type lectin | 6.85E-04 |
| INTERPRO | IPR018378:C-type lectin, conserved site | 7.84E-04 |
| INTERPRO | IPR001304:C-type lectin | 7.84E-04 |
| SMART | SM00034:CLECT | 9.64E-04 |
| INTERPRO | IPR016186:C-type lectin-like | 1.74E-03 |
| GOTERM_MF_FAT | GO:0005529~sugar binding | 3.67E-03 |
| GOTERM_MF_FAT | GO:0030246~carbohydrate binding | 8.82E-02 |

### Enriched annotation of drugs:

No significant annotation terms found.

## Drug-induced transcriptional module: PC3-28

### 442 genes, 7 chemicals

### Functional enrichment of genes:

| **Annotation Cluster 1** | **Enrichment Score: 5.9294426787356675** | |
|---|---|---|
| | | |
| Category | Term | PValue |
| UP_SEQ_FEATURE | topological domain:Extracellular | 5.03E-08 |
| SP_PIR_KEYWORDS | signal | 7.94E-07 |
| UP_SEQ_FEATURE | signal peptide | 8.38E-07 |
| UP_SEQ_FEATURE | glycosylation site:N-linked (GlcNAc...) | 1.72E-06 |
| SP_PIR_KEYWORDS | disulfide bond | 1.92E-06 |
| UP_SEQ_FEATURE | disulfide bond | 4.32E-06 |
| SP_PIR_KEYWORDS | glycoprotein | 6.51E-06 |
| | | |
| | | |
| **Annotation Cluster 2** | **Enrichment Score: 4.2262813390808756** | |
| | | |
| Category | Term | PValue |
| UP_SEQ_FEATURE | topological domain:Extracellular | 5.03E-08 |
| UP_SEQ_FEATURE | topological domain:Cytoplasmic | 1.11E-06 |
| UP_SEQ_FEATURE | glycosylation site:N-linked (GlcNAc...) | 1.72E-06 |
| SP_PIR_KEYWORDS | glycoprotein | 6.51E-06 |
| UP_SEQ_FEATURE | transmembrane region | 1.14E-04 |
| SP_PIR_KEYWORDS | transmembrane | 1.41E-04 |
| GOTERM_CC_FAT | GO:0016021~integral to membrane | 4.82E-03 |
| GOTERM_CC_FAT | GO:0031224~intrinsic to membrane | 1.15E-02 |
| SP_PIR_KEYWORDS | membrane | 1.65E-02 |
| | | |
| | | |
| **Annotation Cluster 3** | **Enrichment Score: 3.6566102583076736** | |
| | | |
| Category | Term | PValue |
| UP_SEQ_FEATURE | topological domain:Extracellular | 5.03E-08 |
| UP_SEQ_FEATURE | topological domain:Cytoplasmic | 1.11E-06 |
| SP_PIR_KEYWORDS | receptor | 8.13E-05 |
| GOTERM_CC_FAT | GO:0005886~plasma membrane | 5.64E-03 |
| GOTERM_CC_FAT | GO:0031226~intrinsic to plasma membrane | 6.22E-03 |
| GOTERM_CC_FAT | GO:0005887~integral to plasma membrane | 8.01E-03 |
| GOTERM_CC_FAT | GO:0044459~plasma membrane part | 1.98E-02 |

### Enriched annotation of drugs:

No significant annotation terms found.

## HL60 Dataset:

## Drug-induced transcriptional module: HL60-1

### 38 genes, 18 chemicals

### Functional enrichment of genes:

| | | |
|---|---|---|
| **Annotation Cluster 1** | **Enrichment Score: 10.524184198955725** | |
| | | |
| Category | Term | PValue |
| PIR_SUPERFAMILY | PIRSF002564:metallothionein | 8.06E-14 |
| SP_PIR_KEYWORDS | metal-thiolate cluster | 9.01E-14 |
| UP_SEQ_FEATURE | region of interest:Beta | 9.08E-14 |
| UP_SEQ_FEATURE | region of interest:Alpha | 9.08E-14 |
| UP_SEQ_FEATURE | metal ion-binding site:Divalent metal cation; cluster B | 9.08E-14 |
| UP_SEQ_FEATURE | metal ion-binding site:Divalent metal cation; cluster A | 9.08E-14 |
| INTERPRO | IPR000006:Metallothionein, vertebrate | 9.96E-14 |
| INTERPRO | IPR003019:Metallothionein superfamily, eukaryotic | 9.96E-14 |
| INTERPRO | IPR018064:Metallothionein, vertebrate, metal binding site | 9.96E-14 |
| SP_PIR_KEYWORDS | chelation | 5.43E-12 |
| SP_PIR_KEYWORDS | cadmium | 5.43E-12 |
| GOTERM_MF_FAT | GO:0046870~cadmium ion binding | 3.02E-11 |
| SP_PIR_KEYWORDS | metal binding | 4.12E-10 |
| SP_PIR_KEYWORDS | copper | 1.20E-07 |
| GOTERM_MF_FAT | GO:0005507~copper ion binding | 4.94E-07 |
| SP_PIR_KEYWORDS | acetylated amino end | 5.81E-06 |
| SP_PIR_KEYWORDS | acetylation | 1.98E-01 |
| | | |
| | | |
| **Annotation Cluster 2** | **Enrichment Score: 4.972093002513408** | |
| | | |
| Category | Term | PValue |
| SP_PIR_KEYWORDS | zinc | 4.55E-08 |
| GOTERM_MF_FAT | GO:0008270~zinc ion binding | 1.75E-07 |
| GOTERM_MF_FAT | GO:0046914~transition metal ion binding | 4.50E-06 |
| SP_PIR_KEYWORDS | metal-binding | 3.54E-05 |
| GOTERM_MF_FAT | GO:0046872~metal ion binding | 2.02E-04 |
| GOTERM_MF_FAT | GO:0043169~cation binding | 2.26E-04 |
| GOTERM_MF_FAT | GO:0043167~ion binding | 2.70E-04 |
| | | |
| | | |
| **Annotation Cluster 3** | **Enrichment Score: 1.9502216176763925** | |
| | | |
| Category | Term | PValue |
| GOTERM_BP_FAT | GO:0009615~response to virus | 1.04E-14 |
| SP_PIR_KEYWORDS | Antiviral defense | 2.74E-11 |
| KEGG_PATHWAY | hsa04622:RIG-I-like receptor signaling pathway | 2.24E-04 |
| SP_PIR_KEYWORDS | host-virus interaction | 2.92E-03 |
| SP_PIR_KEYWORDS | cytoplasm | 3.58E-02 |
| GOTERM_BP_FAT | GO:0006952~defense response | 9.17E-02 |
| SP_PIR_KEYWORDS | activator | 1.32E-01 |
| GOTERM_BP_FAT | GO:0006366~transcription from RNA polymerase II promoter | 1.37E-01 |
| GOTERM_MF_FAT | GO:0003677~DNA binding | 1.75E-01 |
| GOTERM_BP_FAT | GO:0006351~transcription, DNA-dependent | 1.89E-01 |
| GOTERM_BP_FAT | GO:0032774~RNA biosynthetic process | 1.91E-01 |
| SP_PIR_KEYWORDS | transcription regulation | 2.65E-01 |
| SP_PIR_KEYWORDS | Transcription | 2.95E-01 |
| SP_PIR_KEYWORDS | dna-binding | 3.17E-01 |
| SP_PIR_KEYWORDS | nucleus | 3.72E-01 |
| GOTERM_MF_FAT | GO:0003700~transcription factor activity | 3.91E-01 |
| GOTERM_BP_FAT | GO:0006355~regulation of transcription, DNA-dependent | 4.44E-01 |
| GOTERM_BP_FAT | GO:0051252~regulation of RNA metabolic process | 4.69E-01 |

| | | |
|---|---|---|
| GOTERM_MF_FAT | GO:0030528~transcription regulator activity | 5.62E-01 |
| GOTERM_BP_FAT | GO:0045449~regulation of transcription | 6.09E-01 |
| GOTERM_BP_FAT | GO:0006350~transcription | 6.18E-01 |

## Enriched annotation of drugs:

No significant annotation terms found.

## Drug-induced transcriptional module: HL60-2

## 56 genes, 12 chemicals

## Functional enrichment of genes:

| **Annotation Cluster 1** | **Enrichment Score: 6.058749490842719** | |
|---|---|---|
| | | |
| Category | Term | PValue |
| GOTERM_BP_FAT | GO:0006986~response to unfolded protein | 3.19E-18 |
| SP_PIR_KEYWORDS | stress response | 2.02E-17 |
| GOTERM_BP_FAT | GO:0051789~response to protein stimulus | 7.64E-17 |
| SP_PIR_KEYWORDS | Chaperone | 2.50E-16 |
| GOTERM_BP_FAT | GO:0006457~protein folding | 2.01E-15 |
| GOTERM_BP_FAT | GO:0010033~response to organic substance | 5.65E-12 |
| SP_PIR_KEYWORDS | molecular chaperone | 7.99E-11 |
| GOTERM_MF_FAT | GO:0051082~unfolded protein binding | 1.41E-10 |
| KEGG_PATHWAY | hsa04612:Antigen processing and presentation | 3.23E-04 |
| SP_PIR_KEYWORDS | ATP | 2.52E-02 |
| GOTERM_MF_FAT | GO:0005524~ATP binding | 5.18E-02 |
| GOTERM_MF_FAT | GO:0032559~adenyl ribonucleotide binding | 5.45E-02 |
| GOTERM_MF_FAT | GO:0030554~adenyl nucleotide binding | 7.27E-02 |
| GOTERM_MF_FAT | GO:0001883~purine nucleoside binding | 7.79E-02 |
| GOTERM_MF_FAT | GO:0001882~nucleoside binding | 8.10E-02 |
| SP_PIR_KEYWORDS | atp-binding | 9.01E-02 |
| GOTERM_MF_FAT | GO:0032553~ribonucleotide binding | 1.54E-01 |
| GOTERM_MF_FAT | GO:0032555~purine ribonucleotide binding | 1.54E-01 |
| GOTERM_MF_FAT | GO:0017076~purine nucleotide binding | 1.89E-01 |
| SP_PIR_KEYWORDS | nucleotide-binding | 2.25E-01 |
| GOTERM_MF_FAT | GO:0000166~nucleotide binding | 3.86E-01 |
| | | |
| | | |
| **Annotation Cluster 2** | **Enrichment Score: 3.5026336402165468** | |
| | | |
| Category | Term | PValue |
| SMART | SM00028:TPR | 3.08E-05 |
| INTERPRO | IPR019734:Tetratricopeptide repeat | 6.30E-05 |
| INTERPRO | IPR013026:Tetratricopeptide region | 8.26E-05 |
| INTERPRO | IPR011990:Tetratricopeptide-like helical | 2.74E-04 |
| SP_PIR_KEYWORDS | tpr repeat | 3.00E-04 |
| INTERPRO | IPR001440:Tetratricopeptide TPR-1 | 3.82E-04 |
| UP_SEQ_FEATURE | repeat:TPR 3 | 1.38E-03 |
| UP_SEQ_FEATURE | repeat:TPR 1 | 2.08E-03 |
| UP_SEQ_FEATURE | repeat:TPR 2 | 2.08E-03 |
| | | |
| | | |
| **Annotation Cluster 3** | **Enrichment Score: 3.220578465853062** | |
| | | |
| Category | Term | PValue |
| GOTERM_MF_FAT | GO:0031072~heat shock protein binding | 3.34E-05 |
| INTERPRO | IPR002939:Chaperone DnaJ, C-terminal | 3.74E-04 |
| INTERPRO | IPR015609:Molecular chaperone, heat shock protein, Hsp40, DnaJ | 5.31E-04 |
| SMART | SM00271:DnaJ | 7.08E-04 |
| INTERPRO | IPR001623:Heat shock protein DnaJ, N-terminal | 9.87E-04 |

| | | |
|---|---|---|
| UP_SEQ_FEATURE | domain:J | 1.04E-03 |
| INTERPRO | IPR018253:Heat shock protein DnaJ, conserved site | 1.08E-03 |
| INTERPRO | IPR003095:Heat shock protein DnaJ | 3.28E-03 |

## Enriched annotation of drugs:

No significant annotation terms found.

## Drug-induced transcriptional module: HL60-3

## 65 genes, 25 chemicals

## Functional enrichment of genes:

| **Annotation Cluster 1** | **Enrichment Score: 23.97768309927233** | |
|---|---|---|
| | | |
| Category | Term | PValue |
| GOTERM_BP_FAT | GO:0022403~cell cycle phase | 1.05E-33 |
| GOTERM_BP_FAT | GO:0000279~M phase | 3.80E-33 |
| GOTERM_BP_FAT | GO:0022402~cell cycle process | 1.26E-31 |
| SP_PIR_KEYWORDS | cell cycle | 1.42E-31 |
| GOTERM_BP_FAT | GO:0000278~mitotic cell cycle | 1.62E-29 |
| GOTERM_BP_FAT | GO:0007049~cell cycle | 3.07E-29 |
| SP_PIR_KEYWORDS | cell division | 2.50E-28 |
| GOTERM_BP_FAT | GO:0007067~mitosis | 3.19E-28 |
| GOTERM_BP_FAT | GO:0000280~nuclear division | 3.19E-28 |
| GOTERM_BP_FAT | GO:0000087~M phase of mitotic cell cycle | 5.24E-28 |
| GOTERM_BP_FAT | GO:0048285~organelle fission | 1.18E-27 |
| SP_PIR_KEYWORDS | mitosis | 1.69E-27 |
| GOTERM_CC_FAT | GO:0015630~microtubule cytoskeleton | 1.04E-23 |
| GOTERM_CC_FAT | GO:0005819~spindle | 9.53E-23 |
| GOTERM_BP_FAT | GO:0051301~cell division | 2.28E-22 |
| GOTERM_CC_FAT | GO:0044430~cytoskeletal part | 3.33E-18 |
| GOTERM_CC_FAT | GO:0005856~cytoskeleton | 2.46E-14 |
| GOTERM_CC_FAT | GO:0043232~intracellular non-membrane-bounded organelle | 2.21E-13 |
| GOTERM_CC_FAT | GO:0043228~non-membrane-bounded organelle | 2.21E-13 |
| SP_PIR_KEYWORDS | cytoskeleton | 3.33E-09 |
| | | |
| | | |
| **Annotation Cluster 2** | **Enrichment Score: 10.491290872132929** | |
| | | |
| Category | Term | PValue |
| GOTERM_BP_FAT | GO:0007017~microtubule-based process | 2.23E-18 |
| GOTERM_BP_FAT | GO:0000226~microtubule cytoskeleton organization | 4.31E-12 |
| GOTERM_BP_FAT | GO:0007010~cytoskeleton organization | 1.01E-07 |
| GOTERM_BP_FAT | GO:0007051~spindle organization | 1.11E-06 |
| | | |
| | | |
| **Annotation Cluster 3** | **Enrichment Score: 5.770467300698389** | |
| | | |
| Category | Term | PValue |
| INTERPRO | IPR019821:Kinesin, motor region, conserved site | 8.88E-09 |
| INTERPRO | IPR001752:Kinesin, motor region | 8.88E-09 |
| UP_SEQ_FEATURE | domain:Kinesin-motor | 1.52E-08 |
| SMART | SM00129:KISc | 4.50E-08 |
| GOTERM_CC_FAT | GO:0005874~microtubule | 1.50E-07 |
| GOTERM_MF_FAT | GO:0003777~microtubule motor activity | 2.88E-07 |
| SP_PIR_KEYWORDS | microtubule | 6.43E-07 |
| SP_PIR_KEYWORDS | motor protein | 6.11E-06 |
| GOTERM_MF_FAT | GO:0003774~motor activity | 1.23E-05 |
| GOTERM_BP_FAT | GO:0007018~microtubule-based movement | 1.53E-05 |
| UP_SEQ_FEATURE | nucleotide phosphate-binding region:ATP | 2.25E-05 |

| | | |
|---|---|---|
| UP_SEQ_FEATURE | region of interest:Globular | 2.31E-03 |
| SP_PIR_KEYWORDS | coiled coil | 1.07E-02 |

## Enriched annotation of drugs:

| Annotation type | Enrichment term | q-value |
|---|---|---|
| ATC code (2nd level) | P01; Antiprotozoals | 4.72E-02 |

## Drug-induced transcriptional module: HL60-4

## 48 genes, 14 chemicals

## Functional enrichment of genes:

| **Annotation Cluster 1** | **Enrichment Score: 3.066730868831405** | |
|---|---|---|
| | | |
| Category | Term | PValue |
| GOTERM_BP_FAT | GO:0042330~taxis | 8.00E-06 |
| GOTERM_BP_FAT | GO:0006935~chemotaxis | 8.00E-06 |
| SP_PIR_KEYWORDS | chemotaxis | 2.41E-05 |
| GOTERM_BP_FAT | GO:0007626~locomotory behavior | 1.51E-04 |
| GOTERM_BP_FAT | GO:0009611~response to wounding | 1.99E-04 |
| GOTERM_BP_FAT | GO:0007610~behavior | 3.68E-04 |
| GOTERM_BP_FAT | GO:0006954~inflammatory response | 3.92E-04 |
| SP_PIR_KEYWORDS | disulfide bond | 2.99E-03 |
| GOTERM_MF_FAT | GO:0042379~chemokine receptor binding | 6.38E-03 |
| KEGG_PATHWAY | hsa04060:Cytokine-cytokine receptor interaction | 9.02E-03 |
| UP_SEQ_FEATURE | disulfide bond | 4.36E-02 |
| KEGG_PATHWAY | hsa04062:Chemokine signaling pathway | 5.18E-02 |
| SP_PIR_KEYWORDS | Secreted | 5.21E-02 |
| | | |
| | | |
| **Annotation Cluster 2** | **Enrichment Score: 2.1440682441168186** | |
| | | |
| Category | Term | PValue |
| GOTERM_BP_FAT | GO:0032101~regulation of response to external stimulus | 1.33E-03 |
| GOTERM_BP_FAT | GO:0032102~negative regulation of response to external stimulus | 6.38E-03 |
| GOTERM_BP_FAT | GO:0048585~negative regulation of response to stimulus | 4.36E-02 |

## Enriched annotation of drugs:

| Annotation type | Enrichment term | q-value |
|---|---|---|
| ATC code (2nd level) | N05; Psycholeptics | 6.04E-02 |
| Side effect | pheochromocytoma | 3.50E-02 |
| Side effect | mitral insufficiency | 5.07E-02 |

## Drug-induced transcriptional module: HL60-5

## 32 genes, 18 chemicals

## Functional enrichment of genes:

| **Annotation Cluster 1** | **Enrichment Score: 1.4436898003851075** | |
|---|---|---|
| | | |
| Category | Term | PValue |
| GOTERM_BP_FAT | GO:0006414~translational elongation | 5.34E-03 |
| KEGG_PATHWAY | hsa03010:Ribosome | 5.51E-03 |
| SP_PIR_KEYWORDS | ribonucleoprotein | 6.02E-03 |

| | | |
|---|---|---|
| GOTERM_CC_FAT | GO:0022625~cytosolic large ribosomal subunit | 7.81E-03 |
| SP_PIR_KEYWORDS | ribosomal protein | 1.15E-02 |
| GOTERM_MF_FAT | GO:0003735~structural constituent of ribosome | 1.20E-02 |
| GOTERM_CC_FAT | GO:0015934~large ribosomal subunit | 1.64E-02 |
| GOTERM_CC_FAT | GO:0030529~ribonucleoprotein complex | 1.92E-02 |
| SP_PIR_KEYWORDS | ribosome | 2.29E-02 |
| GOTERM_CC_FAT | GO:0005840~ribosome | 2.38E-02 |
| GOTERM_CC_FAT | GO:0022626~cytosolic ribosome | 3.45E-02 |
| GOTERM_MF_FAT | GO:0005198~structural molecule activity | 4.00E-02 |
| GOTERM_CC_FAT | GO:0033279~ribosomal subunit | 5.98E-02 |
| GOTERM_BP_FAT | GO:0006412~translation | 6.93E-02 |
| GOTERM_CC_FAT | GO:0044445~cytosolic part | 8.60E-02 |
| SP_PIR_KEYWORDS | protein biosynthesis | 1.05E-01 |
| GOTERM_MF_FAT | GO:0003723~RNA binding | 1.47E-01 |
| GOTERM_CC_FAT | GO:0043228~non-membrane-bounded organelle | 3.68E-01 |
| GOTERM_CC_FAT | GO:0043232~intracellular non-membrane-bounded organelle | 3.68E-01 |
| GOTERM_CC_FAT | GO:0005829~cytosol | 3.97E-01 |

## Enriched annotation of drugs:

No significant annotation terms found.

## Drug-induced transcriptional module: HL60-6

## 28 genes, 23 chemicals

## Functional enrichment of genes:

| **Annotation Cluster 1** | **Enrichment Score: 2.673348435257455** | |
|---|---|---|
| | | |
| Category | Term | PValue |
| UP_SEQ_FEATURE | repeat:WD 5 | 1.15E-03 |
| INTERPRO | IPR019782:WD40 repeat 2 | 1.23E-03 |
| INTERPRO | IPR017986:WD40 repeat, region | 1.42E-03 |
| UP_SEQ_FEATURE | repeat:WD 4 | 1.56E-03 |
| INTERPRO | IPR019781:WD40 repeat, subgroup | 1.60E-03 |
| UP_SEQ_FEATURE | repeat:WD 3 | 1.98E-03 |
| UP_SEQ_FEATURE | repeat:WD 2 | 2.15E-03 |
| UP_SEQ_FEATURE | repeat:WD 1 | 2.15E-03 |
| SP_PIR_KEYWORDS | wd repeat | 2.19E-03 |
| INTERPRO | IPR001680:WD40 repeat | 2.22E-03 |
| INTERPRO | IPR019775:WD40 repeat, conserved site | 2.51E-03 |
| SMART | SM00320:WD40 | 2.95E-03 |
| INTERPRO | IPR015943:WD40/YVTN repeat-like | 3.23E-03 |
| UP_SEQ_FEATURE | repeat:WD 6 | 6.90E-03 |

## Enriched annotation of drugs:

No significant annotation terms found.

## Drug-induced transcriptional module: HL60-7

## 77 genes, 24 chemicals

## Functional enrichment of genes:

| **Annotation Cluster 1** | **Enrichment Score: 3.3849701939923955** | |
|---|---|---|
| | | |
| Category | Term | PValue |

| | | |
|---|---|---|
| UP_SEQ_FEATURE | zinc finger region:C2H2-type 7 | 7.67E-07 |
| UP_SEQ_FEATURE | zinc finger region:C2H2-type 6 | 1.49E-06 |
| INTERPRO | IPR001909:Krueppel-associated box | 1.65E-06 |
| UP_SEQ_FEATURE | domain:KRAB | 2.15E-06 |
| SMART | SM00349:KRAB | 2.34E-06 |
| UP_SEQ_FEATURE | zinc finger region:C2H2-type 8 | 2.47E-06 |
| INTERPRO | IPR015880:Zinc finger, C2H2-like | 3.20E-06 |
| SMART | SM00355:ZnF_C2H2 | 4.24E-06 |
| UP_SEQ_FEATURE | zinc finger region:C2H2-type 17 | 5.06E-06 |
| UP_SEQ_FEATURE | zinc finger region:C2H2-type 5 | 5.32E-06 |
| UP_SEQ_FEATURE | zinc finger region:C2H2-type 12 | 5.44E-06 |
| INTERPRO | IPR013087:Zinc finger, C2H2-type/integrase, DNA-binding | 6.45E-06 |
| UP_SEQ_FEATURE | zinc finger region:C2H2-type 11 | 1.43E-05 |
| UP_SEQ_FEATURE | zinc finger region:C2H2-type 3 | 1.47E-05 |
| INTERPRO | IPR007087:Zinc finger, C2H2-type | 1.66E-05 |
| UP_SEQ_FEATURE | zinc finger region:C2H2-type 16 | 1.72E-05 |
| UP_SEQ_FEATURE | zinc finger region:C2H2-type 13 | 2.24E-05 |
| UP_SEQ_FEATURE | zinc finger region:C2H2-type 10 | 3.29E-05 |
| UP_SEQ_FEATURE | zinc finger region:C2H2-type 15 | 4.08E-05 |
| UP_SEQ_FEATURE | zinc finger region:C2H2-type 4 | 5.43E-05 |
| UP_SEQ_FEATURE | zinc finger region:C2H2-type 2 | 7.13E-05 |
| SP_PIR_KEYWORDS | zinc-finger | 5.38E-04 |
| UP_SEQ_FEATURE | zinc finger region:C2H2-type 9 | 6.25E-04 |
| UP_SEQ_FEATURE | zinc finger region:C2H2-type 18 | 9.32E-04 |
| GOTERM_MF_FAT | GO:0008270~zinc ion binding | 3.11E-03 |
| GOTERM_BP_FAT | GO:0006350~transcription | 3.19E-03 |
| SP_PIR_KEYWORDS | zinc | 3.35E-03 |
| GOTERM_MF_FAT | GO:0003677~DNA binding | 6.27E-03 |
| UP_SEQ_FEATURE | zinc finger region:C2H2-type 1 | 6.39E-03 |
| UP_SEQ_FEATURE | zinc finger region:C2H2-type 14 | 9.92E-03 |
| SP_PIR_KEYWORDS | metal-binding | 1.02E-02 |
| SP_PIR_KEYWORDS | nucleus | 1.50E-02 |
| SP_PIR_KEYWORDS | Transcription | 1.97E-02 |
| SP_PIR_KEYWORDS | dna-binding | 2.13E-02 |
| GOTERM_BP_FAT | GO:0045449~regulation of transcription | 2.29E-02 |
| UP_SEQ_FEATURE | zinc finger region:C2H2-type 1; degenerate | 2.42E-02 |
| PIR_SUPERFAMILY | PIRSF005559:zinc finger protein ZFP-36 | 2.45E-02 |
| GOTERM_MF_FAT | GO:0046914~transition metal ion binding | 2.56E-02 |
| GOTERM_BP_FAT | GO:0006355~regulation of transcription, DNA-dependent | 2.62E-02 |
| GOTERM_MF_FAT | GO:0046872~metal ion binding | 2.99E-02 |
| GOTERM_BP_FAT | GO:0051252~regulation of RNA metabolic process | 3.23E-02 |
| GOTERM_MF_FAT | GO:0043169~cation binding | 3.25E-02 |
| GOTERM_MF_FAT | GO:0043167~ion binding | 3.72E-02 |
| SP_PIR_KEYWORDS | zinc finger | 5.22E-02 |
| SP_PIR_KEYWORDS | transcription regulation | 6.18E-02 |
| SP_PIR_KEYWORDS | DNA binding | 1.02E-01 |

## Enriched annotation of drugs:

No significant annotation terms found.

## <u>Drug-induced transcriptional module: HL60-8</u>

**84 genes, 28 chemicals**

## Functional enrichment of genes:

| **Annotation Cluster 1** | **Enrichment Score: 5.213611174152916** | |
|---|---|---|
| | | |
| Category | Term | PValue |
| GOTERM_CC_FAT | GO:0031981~nuclear lumen | 1.69E-06 |
| GOTERM_CC_FAT | GO:0031974~membrane-enclosed lumen | 4.58E-06 |
| GOTERM_CC_FAT | GO:0005654~nucleoplasm | 6.52E-06 |
| GOTERM_CC_FAT | GO:0070013~intracellular organelle lumen | 7.08E-06 |
| GOTERM_CC_FAT | GO:0043233~organelle lumen | 1.01E-05 |
| SP_PIR_KEYWORDS | nucleus | 1.45E-05 |
| | | |
| | | |
| **Annotation Cluster 2** | **Enrichment Score: 2.518298160745594** | |
| | | |
| Category | Term | PValue |
| SP_PIR_KEYWORDS | rna-binding | 1.57E-04 |
| GOTERM_MF_FAT | GO:0003723~RNA binding | 3.69E-04 |
| UP_SEQ_FEATURE | domain:RRM 3 | 3.66E-03 |
| UP_SEQ_FEATURE | domain:RRM 1 | 4.06E-03 |
| UP_SEQ_FEATURE | domain:RRM 2 | 4.06E-03 |
| INTERPRO | IPR000504:RNA recognition motif, RNP-1 | 1.05E-02 |
| INTERPRO | IPR012677:Nucleotide-binding, alpha-beta plait | 1.15E-02 |
| SMART | SM00360:RRM | 1.69E-02 |
| | | |
| | | |
| **Annotation Cluster 3** | **Enrichment Score: 2.117483909241662** | |
| | | |
| Category | Term | PValue |
| GOTERM_BP_FAT | GO:0016071~mRNA metabolic process | 6.25E-05 |
| GOTERM_BP_FAT | GO:0006397~mRNA processing | 1.08E-04 |
| GOTERM_BP_FAT | GO:0000375~RNA splicing, via transesterification reactions | 2.51E-04 |
| GOTERM_BP_FAT | GO:0000377~RNA splicing, via transesterification reactions with bulged adenosine as nucleophile | 2.51E-04 |
| GOTERM_BP_FAT | GO:0000398~nuclear mRNA splicing, via spliceosome | 2.51E-04 |
| GOTERM_BP_FAT | GO:0008380~RNA splicing | 2.87E-04 |
| GOTERM_BP_FAT | GO:0006396~RNA processing | 2.08E-03 |
| SP_PIR_KEYWORDS | mrna splicing | 3.78E-03 |
| SP_PIR_KEYWORDS | mrna processing | 9.14E-03 |
| GOTERM_MF_FAT | GO:0031202~RNA splicing factor activity, transesterification mechanism | 1.70E-02 |
| GOTERM_BP_FAT | GO:0000245~spliceosome assembly | 3.38E-02 |
| KEGG_PATHWAY | hsa03040:Spliceosome | 4.39E-02 |
| SP_PIR_KEYWORDS | Spliceosome | 5.39E-02 |
| GOTERM_CC_FAT | GO:0005681~spliceosome | 8.21E-02 |
| GOTERM_BP_FAT | GO:0034621~cellular macromolecular complex subunit organization | 9.02E-02 |
| GOTERM_BP_FAT | GO:0022618~ribonucleoprotein complex assembly | 1.11E-01 |
| GOTERM_BP_FAT | GO:0034622~cellular macromolecular complex assembly | 1.65E-01 |
| GOTERM_CC_FAT | GO:0030529~ribonucleoprotein complex | 3.33E-01 |
| GOTERM_BP_FAT | GO:0022613~ribonucleoprotein complex biogenesis | 4.32E-01 |

## Enriched annotation of drugs:

## <u>Drug-induced transcriptional module: HL60-9</u>

## 60 genes, 29 chemicals

## Functional enrichment of genes:

| **Annotation Cluster 1** | **Enrichment Score: 27.825120128126844** | |
|---|---|---|
| | | |

| Category | Term | PValue |
|---|---|---|
| SP_PIR_KEYWORDS | endoplasmic reticulum | 3.31E-35 |
| GOTERM_CC_FAT | GO:0005783~endoplasmic reticulum | 4.52E-28 |
| GOTERM_CC_FAT | GO:0044432~endoplasmic reticulum part | 2.24E-22 |
| | | |
| | | |
| **Annotation Cluster 2** | **Enrichment Score: 11.529074784265054** | |
| | | |
| Category | Term | PValue |
| GOTERM_CC_FAT | GO:0044432~endoplasmic reticulum part | 2.24E-22 |
| GOTERM_CC_FAT | GO:0012505~endomembrane system | 1.32E-15 |
| GOTERM_CC_FAT | GO:0005789~endoplasmic reticulum membrane | 9.78E-15 |
| GOTERM_CC_FAT | GO:0042175~nuclear envelope-endoplasmic reticulum network | 2.29E-14 |
| GOTERM_BP_FAT | GO:0015031~protein transport | 6.75E-14 |
| GOTERM_BP_FAT | GO:0045184~establishment of protein localization | 8.02E-14 |
| GOTERM_BP_FAT | GO:0046907~intracellular transport | 9.00E-13 |
| GOTERM_BP_FAT | GO:0008104~protein localization | 1.04E-12 |
| GOTERM_BP_FAT | GO:0006886~intracellular protein transport | 9.17E-12 |
| GOTERM_CC_FAT | GO:0031090~organelle membrane | 1.90E-11 |
| GOTERM_BP_FAT | GO:0034613~cellular protein localization | 3.24E-11 |
| GOTERM_BP_FAT | GO:0070727~cellular macromolecule localization | 3.57E-11 |
| SP_PIR_KEYWORDS | protein transport | 1.99E-09 |
| SP_PIR_KEYWORDS | transport | 1.15E-04 |
| UP_SEQ_FEATURE | topological domain:Cytoplasmic | 7.52E-04 |
| | | |
| | | |
| **Annotation Cluster 3** | **Enrichment Score: 7.14742746478565** | |
| | | |
| Category | Term | PValue |
| UP_SEQ_FEATURE | short sequence motif:Prevents secretion from ER | 1.95E-15 |
| SP_PIR_KEYWORDS | signal | 4.12E-04 |
| UP_SEQ_FEATURE | signal peptide | 4.50E-04 |

## Enriched annotation of drugs:

| **Annotation type** | **Enrichment term** | **q-value** |
|---|---|---|
| Drug target | CYP1B1;cytochrome P450, family 1, subfamily B, polypeptide 1 | 1.52E-02 |
| ATC code (2nd level) | G03; Sex hormones and modulators of the genital system | 4.72E-02 |
| Side effect | bacterial infections | 3.87E-02 |

## Drug-induced transcriptional module: HL60-10

## 106 genes, 8 chemicals

## Functional enrichment of genes:

| **Annotation Cluster 1** | **Enrichment Score: 1.5713418731670632** | |
|---|---|---|
| | | |
| Category | Term | PValue |
| GOTERM_CC_FAT | GO:0005654~nucleoplasm | 1.58E-03 |
| GOTERM_CC_FAT | GO:0070013~intracellular organelle lumen | 3.88E-02 |
| GOTERM_CC_FAT | GO:0044451~nucleoplasm part | 4.61E-02 |
| GOTERM_CC_FAT | GO:0043233~organelle lumen | 4.67E-02 |
| GOTERM_CC_FAT | GO:0031981~nuclear lumen | 4.95E-02 |
| GOTERM_CC_FAT | GO:0031974~membrane-enclosed lumen | 5.74E-02 |

## Enriched annotation of drugs:

No significant annotation terms found.

## Drug-induced transcriptional module: HL60-11

## 100 genes, 12 chemicals

## Functional enrichment of genes:

| **Annotation Cluster 1** | **Enrichment Score: 1.6371820778882493** | |
|---|---|---|
| | | |
| Category | Term | PValue |
| SP_PIR_KEYWORDS | GTP binding | 3.64E-04 |
| GOTERM_MF_FAT | GO:0005525~GTP binding | 4.29E-03 |
| GOTERM_MF_FAT | GO:0019001~guanyl nucleotide binding | 4.41E-03 |
| GOTERM_MF_FAT | GO:0032561~guanyl ribonucleotide binding | 4.41E-03 |
| SP_PIR_KEYWORDS | P-loop | 4.87E-03 |
| GOTERM_MF_FAT | GO:0003924~GTPase activity | 5.36E-03 |
| UP_SEQ_FEATURE | nucleotide phosphate-binding region:GTP | 1.33E-02 |
| SP_PIR_KEYWORDS | nucleotide binding | 1.85E-02 |
| SP_PIR_KEYWORDS | gtp-binding | 2.10E-02 |
| INTERPRO | IPR005225:Small GTP-binding protein | 8.97E-02 |
| GOTERM_BP_FAT | GO:0016192~vesicle-mediated transport | 1.38E-01 |
| SP_PIR_KEYWORDS | lipoprotein | 4.24E-01 |
| GOTERM_BP_FAT | GO:0007264~small GTPase mediated signal transduction | 6.42E-01 |
| GOTERM_BP_FAT | GO:0007242~intracellular signaling cascade | 8.67E-01 |
| | | |
| | | |
| **Annotation Cluster 2** | **Enrichment Score: 1.4600742320537519** | |
| | | |
| Category | Term | PValue |
| GOTERM_CC_FAT | GO:0000793~condensed chromosome | 2.16E-02 |
| GOTERM_CC_FAT | GO:0000228~nuclear chromosome | 4.03E-02 |
| GOTERM_CC_FAT | GO:0000794~condensed nuclear chromosome | 4.78E-02 |

## Enriched annotation of drugs:

No significant annotation terms found.

## <u>Drug-induced transcriptional module: HL60-12</u>

## 25 genes, 6 chemicals

## Functional enrichment of genes:

| **Annotation Cluster 1** | **Enrichment Score: 1.3752974022752658** | |
|---|---|---|
| | | |
| Category | Term | PValue |
| UP_SEQ_FEATURE | active site:Phosphocysteine intermediate | 8.82E-03 |
| GOTERM_MF_FAT | GO:0004725~protein tyrosine phosphatase activity | 1.54E-02 |
| SP_PIR_KEYWORDS | protein phosphatase | 2.14E-02 |
| GOTERM_MF_FAT | GO:0004721~phosphoprotein phosphatase activity | 3.86E-02 |
| GOTERM_MF_FAT | GO:0016791~phosphatase activity | 7.45E-02 |
| SP_PIR_KEYWORDS | hydrolase | 6.70E-01 |

## Enriched annotation of drugs:

No significant annotation terms found.

## <u>Drug-induced transcriptional module: HL60-13</u>

## 24 genes, 6 chemicals

## Functional enrichment of genes:

| **Annotation Cluster 1** | **Enrichment Score: 1.4270650338962307** | |
|---|---|---|
| | | |

| Category | Term | PValue |
|---|---|---|
| SP_PIR_KEYWORDS | mrna processing | 1.26E-02 |
| GOTERM_BP_FAT | GO:0008380~RNA splicing | 1.65E-02 |
| GOTERM_BP_FAT | GO:0006396~RNA processing | 1.68E-02 |
| GOTERM_BP_FAT | GO:0006397~mRNA processing | 2.06E-02 |
| GOTERM_BP_FAT | GO:0016071~mRNA metabolic process | 2.87E-02 |
| SP_PIR_KEYWORDS | mrna splicing | 6.31E-02 |
| GOTERM_MF_FAT | GO:0003723~RNA binding | 1.18E-01 |
| SP_PIR_KEYWORDS | rna-binding | 2.47E-01 |

### Enriched annotation of drugs:

| Annotation type | Enrichment term | q-value |
|---|---|---|
| Drug target | CYP1B1;cytochrome P450, family 1, subfamily B, polypeptide 1 | 9.42E-04 |

## <u>Drug-induced transcriptional module: HL60-14</u>

### 24 genes, 13 chemicals

### Functional enrichment of genes:

No significant annotation clusters found.

### Enriched annotation of drugs:

No significant annotation terms found.

## <u>Drug-induced transcriptional module: HL60-15</u>

### 94 genes, 34 chemicals

### Functional enrichment of genes:

| **Annotation Cluster 1** | **Enrichment Score: 3.492451306127592** | |
|---|---|---|
| | | |
| Category | Term | PValue |
| SP_PIR_KEYWORDS | signal | 1.44E-05 |
| UP_SEQ_FEATURE | signal peptide | 1.48E-05 |
| UP_SEQ_FEATURE | glycosylation site:N-linked (GlcNAc...) | 4.18E-04 |
| SP_PIR_KEYWORDS | glycoprotein | 5.06E-04 |
| UP_SEQ_FEATURE | topological domain:Extracellular | 1.30E-03 |
| UP_SEQ_FEATURE | disulfide bond | 1.87E-03 |
| SP_PIR_KEYWORDS | disulfide bond | 3.26E-03 |
| | | |
| | | |
| **Annotation Cluster 2** | **Enrichment Score: 2.6609288051515803** | |
| | | |
| Category | Term | PValue |
| GOTERM_BP_FAT | GO:0009617~response to bacterium | 4.42E-07 |
| GOTERM_BP_FAT | GO:0002684~positive regulation of immune system process | 1.25E-05 |
| GOTERM_BP_FAT | GO:0002237~response to molecule of bacterial origin | 2.52E-05 |
| GOTERM_BP_FAT | GO:0032496~response to lipopolysaccharide | 1.41E-04 |
| GOTERM_BP_FAT | GO:0006955~immune response | 1.47E-04 |
| GOTERM_BP_FAT | GO:0050778~positive regulation of immune response | 2.11E-04 |
| GOTERM_BP_FAT | GO:0006952~defense response | 2.44E-04 |
| GOTERM_BP_FAT | GO:0042742~defense response to bacterium | 5.77E-04 |
| GOTERM_BP_FAT | GO:0048584~positive regulation of response to stimulus | 6.91E-04 |
| GOTERM_BP_FAT | GO:0006954~inflammatory response | 1.77E-03 |
| GOTERM_BP_FAT | GO:0051240~positive regulation of multicellular organismal process | 3.42E-03 |

| | | |
|---|---|---|
| GOTERM_BP_FAT | GO:0045087~innate immune response | 4.68E-03 |
| GOTERM_BP_FAT | GO:0001817~regulation of cytokine production | 6.13E-03 |
| GOTERM_BP_FAT | GO:0048545~response to steroid hormone stimulus | 7.96E-03 |
| GOTERM_BP_FAT | GO:0001818~negative regulation of cytokine production | 1.76E-02 |
| GOTERM_BP_FAT | GO:0002824~positive regulation of adaptive immune response based on somatic recombination of immune receptors built from immunoglobulin superfamily domains | 1.94E-02 |
| GOTERM_BP_FAT | GO:0002821~positive regulation of adaptive immune response | 2.13E-02 |
| GOTERM_BP_FAT | GO:0001819~positive regulation of cytokine production | 2.47E-02 |
| GOTERM_BP_FAT | GO:0002822~regulation of adaptive immune response based on somatic recombination of immune receptors built from immunoglobulin superfamily domains | 5.20E-02 |
| GOTERM_BP_FAT | GO:0002819~regulation of adaptive immune response | 5.48E-02 |
| GOTERM_BP_FAT | GO:0042493~response to drug | 6.63E-02 |
| GOTERM_BP_FAT | GO:0002526~acute inflammatory response | 9.85E-02 |
| GOTERM_BP_FAT | GO:0051241~negative regulation of multicellular organismal process | 2.31E-01 |
| | | |
| | | |
| **Annotation Cluster 3** | **Enrichment Score: 2.4955008902446507** | |
| | | |
| Category | Term | PValue |
| GOTERM_CC_FAT | GO:0031224~intrinsic to membrane | 3.62E-04 |
| SP_PIR_KEYWORDS | receptor | 3.84E-04 |
| SP_PIR_KEYWORDS | transmembrane protein | 4.13E-04 |
| UP_SEQ_FEATURE | glycosylation site:N-linked (GlcNAc...) | 4.18E-04 |
| SP_PIR_KEYWORDS | glycoprotein | 5.06E-04 |
| UP_SEQ_FEATURE | topological domain:Extracellular | 1.30E-03 |
| GOTERM_CC_FAT | GO:0016021~integral to membrane | 2.32E-03 |
| UP_SEQ_FEATURE | transmembrane region | 2.37E-03 |
| SP_PIR_KEYWORDS | transmembrane | 2.56E-03 |
| UP_SEQ_FEATURE | topological domain:Cytoplasmic | 3.69E-03 |
| SP_PIR_KEYWORDS | membrane | 1.28E-02 |
| GOTERM_CC_FAT | GO:0031226~intrinsic to plasma membrane | 1.45E-02 |
| GOTERM_CC_FAT | GO:0005886~plasma membrane | 2.13E-02 |
| GOTERM_CC_FAT | GO:0005887~integral to plasma membrane | 3.10E-02 |
| GOTERM_CC_FAT | GO:0044459~plasma membrane part | 3.66E-01 |

## Enriched annotation of drugs:

| **Annotation type** | **Enrichment term** | **q-value** |
|---|---|---|
| Drug target | NR3C1;nuclear receptor subfamily 3, group C, member 1 (glucocorticoid receptor) | 1.52E-02 |
| ATC code (2nd level) | D07; Corticosteroids, dermatological preparations | 8.02E-20 |
| ATC code (2nd level) | H02; Corticosteroids for systemic use | 8.27E-07 |
| ATC code (2nd level) | R01; Nasal preparations | 4.40E-06 |
| ATC code (2nd level) | R03; Drugs for obstructive airway diseases | 2.26E-03 |
| ATC code (2nd level) | C05; Vasoprotectives | 5.94E-02 |
| ATC code (2nd level) | A07; Antidiarrheals, intestinal anti-inflammatory/anti-infective agents | 6.04E-02 |
| ATC code (2nd level) | D10; Anti-acne preparations | 6.69E-02 |
| Side effect | striae | 2.34E-14 |
| Side effect | skin atrophy | 4.21E-10 |
| Side effect | hypopigmentation | 2.49E-07 |
| Side effect | miliaria | 3.04E-07 |
| Side effect | hypertrichosis | 6.29E-05 |
| Side effect | folliculitis | 6.29E-05 |
| Side effect | cataract | 6.29E-05 |
| Side effect | Aseptic necrosis | 2.90E-04 |

| | | |
|---|---|---|
| Side effect | paraparesis | 7.45E-04 |
| Side effect | nasal septum perforation | 7.45E-04 |
| Side effect | alkalosis | 8.11E-04 |
| Side effect | ocular infection | 8.24E-04 |
| Side effect | exophthalmos | 1.98E-03 |
| Side effect | paraplegia | 3.07E-03 |
| Side effect | glaucoma | 3.47E-03 |
| Side effect | osteoporosis | 7.51E-03 |
| Side effect | infection | 1.40E-02 |
| Side effect | pseudotumor cerebri | 1.77E-02 |
| Side effect | latent diabetes | 3.80E-02 |
| Side effect | arachnoiditis | 3.82E-02 |
| Side effect | abscess | 4.52E-02 |
| Side effect | pathological fracture | 4.88E-02 |
| Side effect | papilledema | 4.88E-02 |
| Side effect | hypernatremia | 9.97E-02 |

## Drug-induced transcriptional module: HL60-16

### 86 genes,  9 chemicals

### Functional enrichment of genes:

| **Annotation Cluster 1** | **Enrichment Score: 2.368317049363443** | |
|---|---|---|
| | | |
| Category | Term | PValue |
| GOTERM_BP_FAT | GO:0048839~inner ear development | 1.75E-03 |
| GOTERM_BP_FAT | GO:0043583~ear development | 3.83E-03 |
| GOTERM_BP_FAT | GO:0007423~sensory organ development | 1.17E-02 |
| | | |
| | | |
| **Annotation Cluster 2** | **Enrichment Score: 1.7185844063394211** | |
| | | |
| Category | Term | PValue |
| SP_PIR_KEYWORDS | Secreted | 1.43E-03 |
| GOTERM_CC_FAT | GO:0005576~extracellular region | 2.11E-03 |
| SP_PIR_KEYWORDS | signal | 1.37E-02 |
| UP_SEQ_FEATURE | signal peptide | 1.38E-02 |
| SP_PIR_KEYWORDS | glycoprotein | 1.98E-02 |
| GOTERM_CC_FAT | GO:0044421~extracellular region part | 3.02E-02 |
| UP_SEQ_FEATURE | glycosylation site:N-linked (GlcNAc...) | 3.92E-02 |
| UP_SEQ_FEATURE | disulfide bond | 4.43E-02 |
| SP_PIR_KEYWORDS | disulfide bond | 6.12E-02 |
| GOTERM_CC_FAT | GO:0005615~extracellular space | 1.79E-01 |

### Enriched annotation of drugs:

No significant annotation terms found.

## Drug-induced transcriptional module: HL60-17

### 21 genes,  27 chemicals

## Functional enrichment of genes:

| **Annotation Cluster 1** | **Enrichment Score: 1.88444241791161** | |
|---|---|---|
| | | |
| Category | Term | PValue |
| GOTERM_BP_FAT | GO:0051249~regulation of lymphocyte activation | 1.15E-02 |
| GOTERM_BP_FAT | GO:0002694~regulation of leukocyte activation | 1.32E-02 |
| GOTERM_BP_FAT | GO:0050865~regulation of cell activation | 1.46E-02 |

## Enriched annotation of drugs:

| **Annotation type** | **Enrichment term** | **q-value** |
|---|---|---|
| Drug target | ADRB2;adrenergic, beta-2-, receptor, surface | 4.54E-03 |
| ATC code (2nd level) | R03; Drugs for obstructive airway diseases | 1.46E-03 |
| ATC code (2nd level) | G02; Other gynecologicals | 4.72E-02 |

## Drug-induced transcriptional module: HL60-18

## 22 genes, 7 chemicals

## Functional enrichment of genes:

No significant annotation clusters found.

## Enriched annotation of drugs:

| **Annotation type** | **Enrichment term** | **q-value** |
|---|---|---|
| ATC code (2nd level) | C01; Cardiac therapy | 2.79E-02 |

## Drug-induced transcriptional module: HL60-19

## 54 genes, 6 chemicals

## Functional enrichment of genes:

| **Annotation Cluster 1** | **Enrichment Score: 1.3786462760838656** | |
|---|---|---|
| | | |
| Category | Term | PValue |
| GOTERM_BP_FAT | GO:0050657~nucleic acid transport | 1.32E-02 |
| GOTERM_BP_FAT | GO:0050658~RNA transport | 1.32E-02 |
| GOTERM_BP_FAT | GO:0051236~establishment of RNA localization | 1.32E-02 |
| GOTERM_BP_FAT | GO:0006403~RNA localization | 1.41E-02 |
| GOTERM_BP_FAT | GO:0015931~nucleobase, nucleoside, nucleotide and nucleic acid transport | 1.65E-02 |
| GOTERM_BP_FAT | GO:0006405~RNA export from nucleus | 1.78E-02 |
| GOTERM_BP_FAT | GO:0051168~nuclear export | 3.67E-02 |
| SP_PIR_KEYWORDS | mRNA transport | 5.13E-02 |
| GOTERM_BP_FAT | GO:0051028~mRNA transport | 7.21E-02 |
| GOTERM_BP_FAT | GO:0006913~nucleocytoplasmic transport | 1.82E-01 |
| GOTERM_BP_FAT | GO:0051169~nuclear transport | 1.82E-01 |
| SP_PIR_KEYWORDS | rna-binding | 6.67E-01 |

## Enriched annotation of drugs:

No significant annotation terms found.

## Drug-induced transcriptional module: HL60-20

## 23 genes, 6 chemicals

## Functional enrichment of genes:

| **Annotation Cluster 1** | **Enrichment Score: 5.246434490449879** | |
|---|---|---|
| | | |
| Category | Term | PValue |
| SP_PIR_KEYWORDS | nucleosome core | 4.79E-12 |
| INTERPRO | IPR007125:Histone core | 5.27E-12 |
| SP_PIR_KEYWORDS | citrullination | 1.23E-11 |
| GOTERM_CC_FAT | GO:0000786~nucleosome | 2.28E-10 |
| SP_PIR_KEYWORDS | methylation | 7.43E-10 |
| GOTERM_BP_FAT | GO:0006334~nucleosome assembly | 3.55E-09 |
| GOTERM_BP_FAT | GO:0031497~chromatin assembly | 4.61E-09 |
| GOTERM_CC_FAT | GO:0032993~protein-DNA complex | 6.45E-09 |
| GOTERM_BP_FAT | GO:0065004~protein-DNA complex assembly | 7.51E-09 |
| GOTERM_BP_FAT | GO:0034728~nucleosome organization | 8.43E-09 |
| KEGG_PATHWAY | hsa05322:Systemic lupus erythematosus | 1.13E-08 |
| GOTERM_BP_FAT | GO:0006323~DNA packaging | 4.15E-08 |
| SP_PIR_KEYWORDS | chromosomal protein | 4.33E-08 |
| GOTERM_BP_FAT | GO:0006333~chromatin assembly or disassembly | 7.41E-08 |
| INTERPRO | IPR009072:Histone-fold | 8.71E-07 |
| INTERPRO | IPR002119:Histone H2A | 1.33E-06 |
| SMART | SM00414:H2A | 1.81E-06 |
| GOTERM_CC_FAT | GO:0000785~chromatin | 3.05E-06 |
| PIR_SUPERFAMILY | PIRSF002048:histone H2A | 7.27E-06 |
| SP_PIR_KEYWORDS | acetyllysine | 1.66E-05 |
| GOTERM_BP_FAT | GO:0034622~cellular macromolecular complex assembly | 3.60E-05 |
| GOTERM_BP_FAT | GO:0034621~cellular macromolecular complex subunit organization | 6.73E-05 |
| GOTERM_BP_FAT | GO:0006325~chromatin organization | 8.22E-05 |
| SP_PIR_KEYWORDS | isopeptide bond | 1.71E-04 |
| GOTERM_CC_FAT | GO:0044427~chromosomal part | 1.71E-04 |
| GOTERM_BP_FAT | GO:0065003~macromolecular complex assembly | 3.17E-04 |
| UP_SEQ_FEATURE | cross-link:Glycyl lysine isopeptide (Lys-Gly) (interchain with G-Cter in ubiquitin) | 3.38E-04 |
| SP_PIR_KEYWORDS | ubl conjugation | 3.52E-04 |
| GOTERM_BP_FAT | GO:0051276~chromosome organization | 3.78E-04 |
| GOTERM_CC_FAT | GO:0005694~chromosome | 4.17E-04 |
| GOTERM_BP_FAT | GO:0043933~macromolecular complex subunit organization | 4.36E-04 |
| SP_PIR_KEYWORDS | methylated amino acid | 5.72E-04 |
| SP_PIR_KEYWORDS | DNA binding | 1.93E-03 |
| SP_PIR_KEYWORDS | acetylation | 1.24E-02 |
| SP_PIR_KEYWORDS | dna-binding | 1.71E-02 |
| GOTERM_MF_FAT | GO:0003677~DNA binding | 4.60E-02 |
| SP_PIR_KEYWORDS | nucleus | 2.64E-01 |
| UP_SEQ_FEATURE | mutagenesis site | 4.28E-01 |
| GOTERM_CC_FAT | GO:0043228~non-membrane-bounded organelle | 4.33E-01 |
| GOTERM_CC_FAT | GO:0043232~intracellular non-membrane-bounded organelle | 4.33E-01 |

## Enriched annotation of drugs:

No significant annotation terms found.

## <u>Drug-induced transcriptional module: HL60-21</u>

## 29 genes, 8 chemicals

## Functional enrichment of genes:

No significant annotation clusters found.

## Enriched annotation of drugs:

No significant annotation terms found.

## Drug-induced transcriptional module: HL60-22

## 23 genes, 84 chemicals

## Functional enrichment of genes:

| **Annotation Cluster 1** | **Enrichment Score: 1.4336548719326556** | |
|---|---|---|
| | | |
| Category | Term | PValue |
| GOTERM_BP_FAT | GO:0016192~vesicle-mediated transport | 1.23E-02 |
| GOTERM_BP_FAT | GO:0007010~cytoskeleton organization | 2.98E-02 |
| GOTERM_CC_FAT | GO:0043228~non-membrane-bounded organelle | 3.12E-02 |
| GOTERM_CC_FAT | GO:0043232~intracellular non-membrane-bounded organelle | 3.12E-02 |
| GOTERM_BP_FAT | GO:0030036~actin cytoskeleton organization | 4.89E-02 |
| GOTERM_BP_FAT | GO:0030029~actin filament-based process | 5.26E-02 |
| GOTERM_MF_FAT | GO:0008092~cytoskeletal protein binding | 5.62E-02 |
| GOTERM_CC_FAT | GO:0005856~cytoskeleton | 6.61E-02 |

## Enriched annotation of drugs:

No significant annotation terms found.

## Drug-induced transcriptional module: HL60-23

## 21 genes, 86 chemicals

## Functional enrichment of genes:

| **Annotation Cluster 1** | **Enrichment Score: 1.4187668866743832** | |
|---|---|---|
| | | |
| Category | Term | PValue |
| UP_SEQ_FEATURE | domain:Leucine-zipper | 6.33E-04 |
| UP_SEQ_FEATURE | DNA-binding region:Basic motif | 9.45E-04 |
| INTERPRO | IPR004827:Basic-leucine zipper (bZIP) transcription factor | 3.35E-03 |
| SMART | SM00338:BRLZ | 7.57E-03 |
| GOTERM_MF_FAT | GO:0046983~protein dimerization activity | 1.06E-02 |
| SP_PIR_KEYWORDS | DNA binding | 1.46E-02 |
| GOTERM_CC_FAT | GO:0031981~nuclear lumen | 2.48E-02 |
| GOTERM_MF_FAT | GO:0003677~DNA binding | 2.49E-02 |
| SP_PIR_KEYWORDS | transcription regulation | 2.69E-02 |
| SP_PIR_KEYWORDS | activator | 3.15E-02 |
| SP_PIR_KEYWORDS | Transcription | 3.17E-02 |
| GOTERM_MF_FAT | GO:0043565~sequence-specific DNA binding | 3.72E-02 |
| SP_PIR_KEYWORDS | dna-binding | 4.95E-02 |
| GOTERM_CC_FAT | GO:0070013~intracellular organelle lumen | 5.88E-02 |
| GOTERM_MF_FAT | GO:0030528~transcription regulator activity | 6.02E-02 |
| GOTERM_CC_FAT | GO:0043233~organelle lumen | 6.33E-02 |
| GOTERM_BP_FAT | GO:0045449~regulation of transcription | 6.47E-02 |
| GOTERM_CC_FAT | GO:0031974~membrane-enclosed lumen | 6.89E-02 |
| GOTERM_BP_FAT | GO:0006350~transcription | 7.02E-02 |
| GOTERM_BP_FAT | GO:0006355~regulation of transcription, DNA-dependent | 9.03E-02 |
| GOTERM_BP_FAT | GO:0051252~regulation of RNA metabolic process | 1.01E-01 |
| SP_PIR_KEYWORDS | nucleus | 1.06E-01 |
| GOTERM_MF_FAT | GO:0016563~transcription activator activity | 1.62E-01 |
| GOTERM_CC_FAT | GO:0005654~nucleoplasm | 1.99E-01 |
| SP_PIR_KEYWORDS | ubl conjugation | 2.50E-01 |
| GOTERM_BP_FAT | GO:0006357~regulation of transcription from RNA polymerase II | 3.47E-01 |

| | | |
|---|---|---|
| | promoter | |
| GOTERM_MF_FAT | GO:0003700~transcription factor activity | 3.67E-01 |

## Enriched annotation of drugs:

## Drug-induced transcriptional module: HL60-24

## 24 genes,  136 chemicals

## Functional enrichment of genes:

| **Annotation Cluster 1** | **Enrichment Score: 1.309522392123892** | |
|---|---|---|
| | | |
| Category | Term | PValue |
| GOTERM_CC_FAT | GO:0030426~growth cone | 4.94E-03 |
| GOTERM_CC_FAT | GO:0030427~site of polarized growth | 5.24E-03 |
| GOTERM_CC_FAT | GO:0030424~axon | 3.16E-02 |
| GOTERM_CC_FAT | GO:0043005~neuron projection | 1.15E-01 |
| GOTERM_CC_FAT | GO:0042995~cell projection | 3.36E-01 |
| SP_PIR_KEYWORDS | disease mutation | 4.40E-01 |

## Enriched annotation of drugs:

No significant annotation terms found.

## Drug-induced transcriptional module: HL60-25

## 33 genes,  32 chemicals

## Functional enrichment of genes:

| **Annotation Cluster 1** | **Enrichment Score: 24.612895134806372** | |
|---|---|---|
| | | |
| Category | Term | PValue |
| SP_PIR_KEYWORDS | lipid synthesis | 1.37E-35 |
| SP_PIR_KEYWORDS | Steroid biosynthesis | 6.90E-33 |
| GOTERM_BP_FAT | GO:0006694~steroid biosynthetic process | 2.19E-27 |
| GOTERM_BP_FAT | GO:0016126~sterol biosynthetic process | 3.12E-27 |
| GOTERM_BP_FAT | GO:0008610~lipid biosynthetic process | 1.69E-26 |
| GOTERM_BP_FAT | GO:0016125~sterol metabolic process | 2.79E-26 |
| GOTERM_BP_FAT | GO:0008202~steroid metabolic process | 1.45E-25 |
| SP_PIR_KEYWORDS | sterol biosynthesis | 3.66E-25 |
| GOTERM_BP_FAT | GO:0008203~cholesterol metabolic process | 1.18E-20 |
| KEGG_PATHWAY | hsa00100:Steroid biosynthesis | 2.83E-19 |
| GOTERM_BP_FAT | GO:0006695~cholesterol biosynthetic process | 4.81E-19 |
| SP_PIR_KEYWORDS | Cholesterol biosynthesis | 1.70E-18 |
| | | |
| | | |
| **Annotation Cluster 2** | **Enrichment Score: 9.809420479594774** | |
| | | |
| Category | Term | PValue |
| GOTERM_BP_FAT | GO:0006694~steroid biosynthetic process | 2.19E-27 |
| KEGG_PATHWAY | hsa00100:Steroid biosynthesis | 2.83E-19 |
| SP_PIR_KEYWORDS | oxidoreductase | 2.90E-12 |
| GOTERM_BP_FAT | GO:0055114~oxidation reduction | 1.30E-10 |
| SP_PIR_KEYWORDS | nadp | 3.70E-08 |
| GOTERM_CC_FAT | GO:0005789~endoplasmic reticulum membrane | 9.07E-07 |
| GOTERM_CC_FAT | GO:0042175~nuclear envelope-endoplasmic reticulum network | 1.44E-06 |
| GOTERM_CC_FAT | GO:0044432~endoplasmic reticulum part | 6.22E-06 |
| GOTERM_CC_FAT | GO:0012505~endomembrane system | 8.68E-05 |
| GOTERM_CC_FAT | GO:0031090~organelle membrane | 1.32E-03 |
| | | |

| | | |
|---|---|---|
| **Annotation Cluster 3** | **Enrichment Score: 7.369766431780055** | |
| | | |
| Category | Term | PValue |
| SP_PIR_KEYWORDS | oxidoreductase | 2.90E-12 |
| SP_PIR_KEYWORDS | endoplasmic reticulum | 1.11E-11 |
| GOTERM_CC_FAT | GO:0005783~endoplasmic reticulum | 6.32E-11 |
| GOTERM_BP_FAT | GO:0055114~oxidation reduction | 1.30E-10 |
| UP_SEQ_FEATURE | transmembrane region | 1.91E-06 |
| SP_PIR_KEYWORDS | transmembrane | 2.06E-06 |
| SP_PIR_KEYWORDS | membrane | 6.37E-06 |
| GOTERM_CC_FAT | GO:0016021~integral to membrane | 2.09E-04 |
| GOTERM_CC_FAT | GO:0031224~intrinsic to membrane | 3.39E-04 |

## Enriched annotation of drugs:

No significant annotation terms found.

## Drug-induced transcriptional module: HL60-26

## 140 genes, 10 chemicals

## Functional enrichment of genes:

No significant annotation clusters found.

## Enriched annotation of drugs:

| Annotation type | Enrichment term | q-value |
|---|---|---|
| Side effect | heartburn | 9.97E-02 |

## Drug-induced transcriptional module: HL60-27

## 193 genes, 7 chemicals

## Functional enrichment of genes:

| | | |
|---|---|---|
| **Annotation Cluster 1** | **Enrichment Score: 1.8270750622353058** | |
| | | |
| Category | Term | PValue |
| INTERPRO | IPR001781:Zinc finger, LIM-type | 1.20E-02 |
| SP_PIR_KEYWORDS | LIM domain | 1.23E-02 |
| SMART | SM00132:LIM | 1.44E-02 |
| UP_SEQ_FEATURE | domain:LIM zinc-binding 2 | 1.85E-02 |
| UP_SEQ_FEATURE | domain:LIM zinc-binding 1 | 1.85E-02 |
| | | |
| | | |
| **Annotation Cluster 2** | **Enrichment Score: 1.3645350185498748** | |
| | | |
| Category | Term | PValue |
| GOTERM_CC_FAT | GO:0031982~vesicle | 3.18E-02 |
| GOTERM_CC_FAT | GO:0031988~membrane-bounded vesicle | 4.02E-02 |
| GOTERM_CC_FAT | GO:0031410~cytoplasmic vesicle | 4.31E-02 |
| GOTERM_CC_FAT | GO:0016023~cytoplasmic membrane-bounded vesicle | 6.32E-02 |

## Enriched annotation of drugs:

No significant annotation terms found.

## Drug-induced transcriptional module: HL60-28

### 245 genes, 6 chemicals

### Functional enrichment of genes:

| **Annotation Cluster 1** | **Enrichment Score: 2.0395676754683163** | |
|---|---|---|
| | | |
| Category | Term | PValue |
| GOTERM_CC_FAT | GO:0031090~organelle membrane | 1.67E-03 |
| GOTERM_CC_FAT | GO:0005789~endoplasmic reticulum membrane | 3.19E-03 |
| GOTERM_CC_FAT | GO:0042175~nuclear envelope-endoplasmic reticulum network | 5.22E-03 |
| GOTERM_CC_FAT | GO:0012505~endomembrane system | 1.03E-02 |
| GOTERM_CC_FAT | GO:0044432~endoplasmic reticulum part | 2.23E-02 |
| SP_PIR_KEYWORDS | endoplasmic reticulum | 2.42E-02 |
| GOTERM_CC_FAT | GO:0005783~endoplasmic reticulum | 3.41E-02 |
| | | |
| | | |
| **Annotation Cluster 2** | **Enrichment Score: 1.7001249816557327** | |
| | | |
| Category | Term | PValue |
| KEGG_PATHWAY | hsa04130:SNARE interactions in vesicular transport | 1.17E-03 |
| UP_SEQ_FEATURE | domain:Longin | 4.36E-03 |
| INTERPRO | IPR010908:Longin | 4.62E-03 |
| GOTERM_BP_FAT | GO:0006888~ER to Golgi vesicle-mediated transport | 1.16E-02 |
| UP_SEQ_FEATURE | domain:v-SNARE coiled-coil homology | 2.39E-02 |
| INTERPRO | IPR001388:Synaptobrevin | 2.52E-02 |
| COG_ONTOLOGY | Intracellular trafficking and secretion | 1.65E-01 |
| GOTERM_CC_FAT | GO:0031410~cytoplasmic vesicle | 9.18E-01 |
| | | |
| | | |
| **Annotation Cluster 3** | **Enrichment Score: 1.637653474676585** | |
| | | |
| Category | Term | PValue |
| GOTERM_BP_FAT | GO:0048193~Golgi vesicle transport | 1.38E-03 |
| SP_PIR_KEYWORDS | golgi apparatus | 4.67E-02 |
| GOTERM_CC_FAT | GO:0005794~Golgi apparatus | 5.65E-02 |
| GOTERM_BP_FAT | GO:0016192~vesicle-mediated transport | 7.73E-02 |

### Enriched annotation of drugs:

No significant annotation terms found.

## Drug-induced transcriptional module: HL60-29

### 293 genes, 7 chemicals

### Functional enrichment of genes:

| **Annotation Cluster 1** | **Enrichment Score: 3.1495767421125063** | |
|---|---|---|
| | | |
| Category | Term | PValue |
| SP_PIR_KEYWORDS | signal | 9.06E-05 |
| UP_SEQ_FEATURE | signal peptide | 9.38E-05 |
| GOTERM_CC_FAT | GO:0044421~extracellular region part | 1.17E-04 |
| SP_PIR_KEYWORDS | Secreted | 2.83E-04 |
| GOTERM_CC_FAT | GO:0005615~extracellular space | 2.86E-04 |
| GOTERM_CC_FAT | GO:0005576~extracellular region | 7.31E-04 |
| SP_PIR_KEYWORDS | glycoprotein | 2.07E-03 |
| SP_PIR_KEYWORDS | disulfide bond | 3.20E-03 |
| UP_SEQ_FEATURE | disulfide bond | 3.66E-03 |

| | | |
|---|---|---|
| UP_SEQ_FEATURE | glycosylation site:N-linked (GlcNAc...) | 2.24E-02 |
| | | |
| | | |
| **Annotation Cluster 2** | **Enrichment Score: 2.932952579342855** | |
| | | |
| Category | Term | PValue |
| GOTERM_BP_FAT | GO:0022610~biological adhesion | 1.07E-03 |
| GOTERM_BP_FAT | GO:0007155~cell adhesion | 1.07E-03 |
| SP_PIR_KEYWORDS | cell adhesion | 1.38E-03 |
| | | |
| | | |
| **Annotation Cluster 3** | **Enrichment Score: 2.7572901756515735** | |
| | | |
| Category | Term | PValue |
| GOTERM_CC_FAT | GO:0022627~cytosolic small ribosomal subunit | 2.62E-06 |
| SP_PIR_KEYWORDS | ribosome | 9.47E-06 |
| GOTERM_CC_FAT | GO:0022626~cytosolic ribosome | 2.48E-05 |
| GOTERM_BP_FAT | GO:0006414~translational elongation | 2.95E-05 |
| GOTERM_CC_FAT | GO:0015935~small ribosomal subunit | 3.85E-05 |
| KEGG_PATHWAY | hsa03010:Ribosome | 2.11E-04 |
| GOTERM_CC_FAT | GO:0033279~ribosomal subunit | 4.17E-04 |
| SP_PIR_KEYWORDS | protein biosynthesis | 6.26E-04 |
| GOTERM_CC_FAT | GO:0044445~cytosolic part | 7.04E-04 |
| GOTERM_MF_FAT | GO:0003735~structural constituent of ribosome | 3.85E-03 |
| SP_PIR_KEYWORDS | ribosomal protein | 4.65E-03 |
| GOTERM_BP_FAT | GO:0006412~translation | 4.73E-03 |
| GOTERM_CC_FAT | GO:0005840~ribosome | 5.50E-03 |
| SP_PIR_KEYWORDS | ribonucleoprotein | 2.13E-02 |
| GOTERM_MF_FAT | GO:0005198~structural molecule activity | 2.37E-02 |
| GOTERM_CC_FAT | GO:0030529~ribonucleoprotein complex | 6.87E-02 |
| SP_PIR_KEYWORDS | blocked amino end | 1.89E-01 |
| GOTERM_CC_FAT | GO:0005829~cytosol | 6.11E-01 |
| GOTERM_MF_FAT | GO:0003723~RNA binding | 8.10E-01 |

## Enriched annotation of drugs:

No significant annotation terms found.

## Rat Liver Dataset:

## Drug-induced transcriptional module: RatLiver-1

## 57 genes,  44 chemicals

## Functional enrichment of genes:

| | | |
|---|---|---|
| **Annotation Cluster 1** | **Enrichment Score: 10.42242170628625** | |
| | | |
| Category | Term | PValue |
| GOTERM_CC_FAT | GO:0005739~mitochondrion | 7.56E-16 |
| GOTERM_CC_FAT | GO:0031966~mitochondrial membrane | 2.34E-11 |
| GOTERM_CC_FAT | GO:0044429~mitochondrial part | 2.92E-11 |
| GOTERM_CC_FAT | GO:0005740~mitochondrial envelope | 6.03E-11 |
| GOTERM_CC_FAT | GO:0031090~organelle membrane | 1.06E-10 |
| GOTERM_CC_FAT | GO:0005743~mitochondrial inner membrane | 1.82E-10 |
| GOTERM_CC_FAT | GO:0019866~organelle inner membrane | 3.91E-10 |
| GOTERM_CC_FAT | GO:0031967~organelle envelope | 7.77E-10 |
| GOTERM_CC_FAT | GO:0031975~envelope | 8.64E-10 |
| | | |
| | | |
| **Annotation Cluster 2** | **Enrichment Score: 6.348889789060915** | |
| | | |

| Category | Term | PValue |
|---|---|---|
| GOTERM_BP_FAT | GO:0006631~fatty acid metabolic process | 3.73E-21 |
| SP_PIR_KEYWORDS | fatty acid metabolism | 3.11E-14 |
| GOTERM_BP_FAT | GO:0019395~fatty acid oxidation | 2.42E-12 |
| GOTERM_BP_FAT | GO:0034440~lipid oxidation | 2.42E-12 |
| GOTERM_BP_FAT | GO:0006635~fatty acid beta-oxidation | 7.04E-12 |
| SP_PIR_KEYWORDS | lipid metabolism | 6.34E-11 |
| GOTERM_BP_FAT | GO:0009062~fatty acid catabolic process | 6.45E-11 |
| GOTERM_BP_FAT | GO:0030258~lipid modification | 1.35E-10 |
| GOTERM_BP_FAT | GO:0044242~cellular lipid catabolic process | 1.02E-08 |
| UP_SEQ_FEATURE | active site:Proton acceptor | 1.84E-08 |
| GOTERM_BP_FAT | GO:0016054~organic acid catabolic process | 8.31E-08 |
| GOTERM_BP_FAT | GO:0046395~carboxylic acid catabolic process | 8.31E-08 |
| GOTERM_BP_FAT | GO:0016042~lipid catabolic process | 7.62E-07 |
| GOTERM_BP_FAT | GO:0055114~oxidation reduction | 2.59E-06 |
| GOTERM_MF_FAT | GO:0050662~coenzyme binding | 3.76E-06 |
| SP_PIR_KEYWORDS | oxidoreductase | 6.36E-06 |
| GOTERM_MF_FAT | GO:0048037~cofactor binding | 3.13E-05 |
| GOTERM_BP_FAT | GO:0033540~fatty acid beta-oxidation using acyl-CoA oxidase | 3.07E-04 |
| SP_PIR_KEYWORDS | acetylation | 3.46E-04 |
| INTERPRO | IPR013786:Acyl-CoA dehydrogenase/oxidase, N-terminal | 1.62E-03 |
| INTERPRO | IPR006091:Acyl-CoA oxidase/dehydrogenase, central region | 1.62E-03 |
| INTERPRO | IPR013764:Acyl-CoA oxidase/dehydrogenase, type1/2, C-terminal | 1.62E-03 |
| GOTERM_MF_FAT | GO:0003995~acyl-CoA dehydrogenase activity | 1.74E-03 |
| SP_PIR_KEYWORDS | Flavoprotein | 5.97E-03 |
| GOTERM_MF_FAT | GO:0051287~NAD or NADH binding | 6.25E-03 |
| SP_PIR_KEYWORDS | FAD | 7.00E-03 |
| UP_SEQ_FEATURE | binding site:Substrate | 3.03E-02 |
| GOTERM_MF_FAT | GO:0050660~FAD binding | 3.73E-02 |
| GOTERM_MF_FAT | GO:0009055~electron carrier activity | 6.54E-02 |
| | | |
| | | |
| **Annotation Cluster 3** | **Enrichment Score: 5.29633239339971** | |
| | | |
| Category | Term | PValue |
| SP_PIR_KEYWORDS | peroxisome | 7.78E-10 |
| GOTERM_CC_FAT | GO:0042579~microbody | 4.53E-09 |
| GOTERM_CC_FAT | GO:0005777~peroxisome | 4.53E-09 |
| UP_SEQ_FEATURE | short sequence motif:Microbody targeting signal | 9.30E-07 |
| GOTERM_CC_FAT | GO:0044438~microbody part | 7.85E-04 |
| GOTERM_CC_FAT | GO:0044439~peroxisomal part | 7.85E-04 |
| GOTERM_CC_FAT | GO:0005778~peroxisomal membrane | 6.82E-03 |
| GOTERM_CC_FAT | GO:0031903~microbody membrane | 6.82E-03 |

## Drug-induced transcriptional module: RatLiver-2

## 54 genes, 49 chemicals

## Functional enrichment of genes:

| **Annotation Cluster 1** | **Enrichment Score: 2.1946100328286176** | |
|---|---|---|
| | | |
| Category | Term | PValue |
| KEGG_PATHWAY | rno04210:Apoptosis | 3.54E-04 |
| GOTERM_BP_FAT | GO:0009617~response to bacterium | 4.67E-03 |
| GOTERM_BP_FAT | GO:0032496~response to lipopolysaccharide | 6.26E-03 |
| GOTERM_BP_FAT | GO:0002237~response to molecule of bacterial origin | 8.02E-03 |
| GOTERM_BP_FAT | GO:0019221~cytokine-mediated signaling pathway | 1.80E-02 |
| KEGG_PATHWAY | rno04060:Cytokine-cytokine receptor interaction | 4.55E-02 |
| | | |

| | | |
|---|---|---|
| **Annotation Cluster 2** | **Enrichment Score: 1.9945647647252696** | |
| | | |
| Category | Term | PValue |
| GOTERM_BP_FAT | GO:0009611~response to wounding | 5.06E-05 |
| GOTERM_BP_FAT | GO:0006954~inflammatory response | 6.20E-05 |
| GOTERM_BP_FAT | GO:0006952~defense response | 2.49E-04 |
| GOTERM_BP_FAT | GO:0050900~leukocyte migration | 8.05E-04 |
| GOTERM_BP_FAT | GO:0002526~acute inflammatory response | 3.00E-03 |
| GOTERM_BP_FAT | GO:0030595~leukocyte chemotaxis | 6.29E-03 |
| GOTERM_BP_FAT | GO:0060326~cell chemotaxis | 7.32E-03 |
| GOTERM_BP_FAT | GO:0016477~cell migration | 9.39E-03 |
| SP_PIR_KEYWORDS | inflammatory response | 1.04E-02 |
| GOTERM_BP_FAT | GO:0042060~wound healing | 1.83E-02 |
| GOTERM_BP_FAT | GO:0051674~localization of cell | 2.05E-02 |
| GOTERM_BP_FAT | GO:0048870~cell motility | 2.05E-02 |
| GOTERM_BP_FAT | GO:0007626~locomotory behavior | 2.91E-02 |
| GOTERM_BP_FAT | GO:0042330~taxis | 2.93E-02 |
| GOTERM_BP_FAT | GO:0006935~chemotaxis | 2.93E-02 |
| GOTERM_CC_FAT | GO:0005615~extracellular space | 4.12E-02 |
| GOTERM_BP_FAT | GO:0006928~cell motion | 4.40E-02 |
| SP_PIR_KEYWORDS | Secreted | 5.58E-02 |
| GOTERM_CC_FAT | GO:0044421~extracellular region part | 1.30E-01 |
| GOTERM_BP_FAT | GO:0007610~behavior | 1.56E-01 |
| GOTERM_BP_FAT | GO:0006955~immune response | 3.88E-01 |
| | | |
| | | |
| **Annotation Cluster 3** | **Enrichment Score: 1.7953360637596505** | |
| | | |
| Category | Term | PValue |
| GOTERM_BP_FAT | GO:0042493~response to drug | 3.80E-03 |
| GOTERM_BP_FAT | GO:0042981~regulation of apoptosis | 5.35E-03 |
| GOTERM_BP_FAT | GO:0043067~regulation of programmed cell death | 5.75E-03 |
| GOTERM_BP_FAT | GO:0010941~regulation of cell death | 5.89E-03 |
| GOTERM_BP_FAT | GO:0006916~anti-apoptosis | 1.13E-02 |
| GOTERM_BP_FAT | GO:0043066~negative regulation of apoptosis | 2.18E-02 |
| GOTERM_BP_FAT | GO:0043069~negative regulation of programmed cell death | 2.29E-02 |
| GOTERM_BP_FAT | GO:0060548~negative regulation of cell death | 2.31E-02 |
| GOTERM_BP_FAT | GO:0045449~regulation of transcription | 7.81E-01 |

## Drug-induced transcriptional module: RatLiver-3

## 45 genes,  43 chemicals

## Functional enrichment of genes:

| | | |
|---|---|---|
| **Annotation Cluster 1** | **Enrichment Score: 1.6296315648475048** | |
| | | |
| Category | Term | PValue |
| GOTERM_MF_FAT | GO:0005509~calcium ion binding | 1.38E-03 |
| GOTERM_MF_FAT | GO:0046872~metal ion binding | 1.98E-02 |
| GOTERM_MF_FAT | GO:0043169~cation binding | 2.20E-02 |
| GOTERM_MF_FAT | GO:0043167~ion binding | 2.53E-02 |
| SP_PIR_KEYWORDS | metal-binding | 4.69E-01 |
| | | |
| | | |
| **Annotation Cluster 2** | **Enrichment Score: 1.550470199092995** | |
| | | |
| Category | Term | PValue |
| GOTERM_MF_FAT | GO:0005509~calcium ion binding | 1.38E-03 |

| | | |
|---|---|---|
| UP_SEQ_FEATURE | calcium-binding region:2 | 1.76E-02 |
| UP_SEQ_FEATURE | calcium-binding region:1 | 2.16E-02 |
| UP_SEQ_FEATURE | domain:EF-hand 1 | 3.29E-02 |
| UP_SEQ_FEATURE | domain:EF-hand 2 | 3.66E-02 |
| INTERPRO | IPR018247:EF-HAND 1 | 6.03E-02 |
| INTERPRO | IPR018249:EF-HAND 2 | 6.28E-02 |
| SP_PIR_KEYWORDS | calcium | 6.68E-02 |
| INTERPRO | IPR011992:EF-Hand type | 6.96E-02 |
| | | |
| | | |
| **Annotation Cluster 3** | **Enrichment Score: 1.3110145664077861** | |
| | | |
| Category | Term | PValue |
| GOTERM_BP_FAT | GO:0045859~regulation of protein kinase activity | 2.89E-02 |
| GOTERM_BP_FAT | GO:0043549~regulation of kinase activity | 3.29E-02 |
| GOTERM_MF_FAT | GO:0019899~enzyme binding | 3.69E-02 |
| GOTERM_BP_FAT | GO:0051338~regulation of transferase activity | 3.71E-02 |
| GOTERM_BP_FAT | GO:0042325~regulation of phosphorylation | 7.51E-02 |
| GOTERM_BP_FAT | GO:0051174~regulation of phosphorus metabolic process | 8.23E-02 |
| GOTERM_BP_FAT | GO:0019220~regulation of phosphate metabolic process | 8.23E-02 |

## Drug-induced transcriptional module: RatLiver-4

## 41 genes, 44 chemicals

## Functional enrichment of genes:

| | | |
|---|---|---|
| **Annotation Cluster 1** | **Enrichment Score: 2.9386134031413946** | |
| | | |
| Category | Term | PValue |
| GOTERM_BP_FAT | GO:0009410~response to xenobiotic stimulus | 8.73E-07 |
| GOTERM_BP_FAT | GO:0006805~xenobiotic metabolic process | 3.95E-05 |
| KEGG_PATHWAY | rno00980:Metabolism of xenobiotics by cytochrome P450 | 1.90E-02 |
| KEGG_PATHWAY | rno00982:Drug metabolism | 2.68E-02 |
| GOTERM_BP_FAT | GO:0010033~response to organic substance | 1.16E-01 |
| | | |
| | | |
| **Annotation Cluster 2** | **Enrichment Score: 2.8027556023899676** | |
| | | |
| Category | Term | PValue |
| GOTERM_BP_FAT | GO:0055114~oxidation reduction | 2.31E-04 |
| SP_PIR_KEYWORDS | oxidoreductase | 3.21E-03 |
| SP_PIR_KEYWORDS | nadp | 5.26E-03 |
| | | |
| | | |
| **Annotation Cluster 3** | **Enrichment Score: 2.7595274676874575** | |
| | | |
| Category | Term | PValue |
| GOTERM_BP_FAT | GO:0051351~positive regulation of ligase activity | 2.43E-05 |
| GOTERM_BP_FAT | GO:0031397~negative regulation of protein ubiquitination | 2.59E-05 |
| GOTERM_BP_FAT | GO:0051340~regulation of ligase activity | 3.52E-05 |
| GOTERM_BP_FAT | GO:0031396~regulation of protein ubiquitination | 1.03E-04 |
| GOTERM_BP_FAT | GO:0031400~negative regulation of protein modification process | 2.11E-04 |
| GOTERM_BP_FAT | GO:0051436~negative regulation of ubiquitin-protein ligase activity during mitotic cell cycle | 4.57E-04 |
| GOTERM_BP_FAT | GO:0031145~anaphase-promoting complex-dependent proteasomal ubiquitin-dependent protein catabolic process | 4.57E-04 |
| SP_PIR_KEYWORDS | proteasome | 4.79E-04 |
| GOTERM_BP_FAT | GO:0051352~negative regulation of ligase activity | 4.82E-04 |
| GOTERM_BP_FAT | GO:0051444~negative regulation of ubiquitin-protein ligase activity | 4.82E-04 |

| | | |
|---|---|---|
| GOTERM_CC_FAT | GO:0000502~proteasome complex | 5.32E-04 |
| GOTERM_BP_FAT | GO:0051437~positive regulation of ubiquitin-protein ligase activity during mitotic cell cycle | 5.36E-04 |
| GOTERM_BP_FAT | GO:0051439~regulation of ubiquitin-protein ligase activity during mitotic cell cycle | 5.93E-04 |
| GOTERM_BP_FAT | GO:0051443~positive regulation of ubiquitin-protein ligase activity | 5.93E-04 |
| GOTERM_BP_FAT | GO:0051438~regulation of ubiquitin-protein ligase activity | 7.88E-04 |
| GOTERM_BP_FAT | GO:0031398~positive regulation of protein ubiquitination | 9.78E-04 |
| GOTERM_BP_FAT | GO:0043632~modification-dependent macromolecule catabolic process | 1.10E-03 |
| GOTERM_BP_FAT | GO:0019941~modification-dependent protein catabolic process | 1.10E-03 |
| GOTERM_BP_FAT | GO:0006511~ubiquitin-dependent protein catabolic process | 1.15E-03 |
| GOTERM_BP_FAT | GO:0032269~negative regulation of cellular protein metabolic process | 1.18E-03 |
| GOTERM_BP_FAT | GO:0051248~negative regulation of protein metabolic process | 1.31E-03 |
| GOTERM_BP_FAT | GO:0010498~proteasomal protein catabolic process | 1.55E-03 |
| GOTERM_BP_FAT | GO:0043161~proteasomal ubiquitin-dependent protein catabolic process | 1.55E-03 |
| GOTERM_BP_FAT | GO:0051603~proteolysis involved in cellular protein catabolic process | 1.62E-03 |
| GOTERM_BP_FAT | GO:0044257~cellular protein catabolic process | 1.69E-03 |
| GOTERM_BP_FAT | GO:0030163~protein catabolic process | 2.22E-03 |
| GOTERM_BP_FAT | GO:0032270~positive regulation of cellular protein metabolic process | 3.33E-03 |
| GOTERM_BP_FAT | GO:0000278~mitotic cell cycle | 3.49E-03 |
| GOTERM_BP_FAT | GO:0051247~positive regulation of protein metabolic process | 3.96E-03 |
| GOTERM_BP_FAT | GO:0043086~negative regulation of catalytic activity | 4.02E-03 |
| GOTERM_BP_FAT | GO:0044265~cellular macromolecule catabolic process | 4.92E-03 |
| GOTERM_BP_FAT | GO:0006508~proteolysis | 5.07E-03 |
| GOTERM_BP_FAT | GO:0032268~regulation of cellular protein metabolic process | 5.75E-03 |
| GOTERM_BP_FAT | GO:0031399~regulation of protein modification process | 7.46E-03 |
| GOTERM_BP_FAT | GO:0043085~positive regulation of catalytic activity | 7.54E-03 |
| GOTERM_BP_FAT | GO:0044092~negative regulation of molecular function | 8.53E-03 |
| GOTERM_BP_FAT | GO:0009057~macromolecule catabolic process | 8.72E-03 |
| GOTERM_BP_FAT | GO:0044093~positive regulation of molecular function | 1.29E-02 |
| GOTERM_BP_FAT | GO:0031401~positive regulation of protein modification process | 1.34E-02 |
| KEGG_PATHWAY | rno03050:Proteasome | 1.34E-02 |
| GOTERM_BP_FAT | GO:0022402~cell cycle process | 1.71E-02 |
| GOTERM_BP_FAT | GO:0007049~cell cycle | 3.89E-02 |
| GOTERM_BP_FAT | GO:0010604~positive regulation of macromolecule metabolic process | 7.87E-02 |
| GOTERM_BP_FAT | GO:0010605~negative regulation of macromolecule metabolic process | 1.04E-01 |

## Drug-induced transcriptional module: RatLiver-5

## 44 genes, 24 chemicals

## Functional enrichment of genes:

| **Annotation Cluster 1** | **Enrichment Score: 2.5047323038031193** | |
|---|---|---|
| | | |
| Category | Term | PValue |
| GOTERM_CC_FAT | GO:0048770~pigment granule | 5.45E-04 |
| GOTERM_CC_FAT | GO:0042470~melanosome | 5.45E-04 |
| GOTERM_CC_FAT | GO:0016023~cytoplasmic membrane-bounded vesicle | 5.08E-03 |
| GOTERM_CC_FAT | GO:0031988~membrane-bounded vesicle | 6.10E-03 |
| GOTERM_CC_FAT | GO:0031410~cytoplasmic vesicle | 8.94E-03 |
| GOTERM_CC_FAT | GO:0031982~vesicle | 1.14E-02 |
| | | |
| | | |
| **Annotation Cluster 2** | **Enrichment Score: 1.4607351114498444** | |
| | | |
| Category | Term | PValue |
| GOTERM_MF_FAT | GO:0017016~Ras GTPase binding | 9.91E-03 |
| GOTERM_MF_FAT | GO:0031267~small GTPase binding | 1.23E-02 |
| GOTERM_MF_FAT | GO:0051020~GTPase binding | 1.53E-02 |

| | | |
|---|---|---|
| GOTERM_MF_FAT | GO:0019899~enzyme binding | 9.76E-02 |
| GOTERM_CC_FAT | GO:0005886~plasma membrane | 2.74E-01 |

## Drug-induced transcriptional module: RatLiver-6

## 40 genes, 20 chemicals

## Functional enrichment of genes:

| **Annotation Cluster 1** | **Enrichment Score: 2.3396706492445296** | |
|---|---|---|
| | | |
| Category | Term | PValue |
| GOTERM_BP_FAT | GO:0006974~response to DNA damage stimulus | 5.06E-05 |
| GOTERM_BP_FAT | GO:0033554~cellular response to stress | 1.16E-04 |
| GOTERM_BP_FAT | GO:0006259~DNA metabolic process | 2.14E-03 |
| SP_PIR_KEYWORDS | DNA damage | 2.38E-02 |
| GOTERM_BP_FAT | GO:0006281~DNA repair | 8.49E-02 |
| SP_PIR_KEYWORDS | cytoplasm | 3.61E-01 |
| | | |
| | | |
| **Annotation Cluster 2** | **Enrichment Score: 2.311507387005395** | |
| | | |
| Category | Term | PValue |
| GOTERM_BP_FAT | GO:0006974~response to DNA damage stimulus | 5.06E-05 |
| GOTERM_BP_FAT | GO:0009314~response to radiation | 1.53E-03 |
| GOTERM_BP_FAT | GO:0009411~response to UV | 7.98E-03 |
| GOTERM_BP_FAT | GO:0009628~response to abiotic stimulus | 2.02E-02 |
| GOTERM_BP_FAT | GO:0051726~regulation of cell cycle | 2.70E-02 |
| GOTERM_BP_FAT | GO:0009416~response to light stimulus | 3.99E-02 |
| | | |
| | | |
| **Annotation Cluster 3** | **Enrichment Score: 1.9952027603453215** | |
| | | |
| Category | Term | PValue |
| GOTERM_BP_FAT | GO:0008285~negative regulation of cell proliferation | 4.94E-03 |
| GOTERM_BP_FAT | GO:0042326~negative regulation of phosphorylation | 5.22E-03 |
| GOTERM_BP_FAT | GO:0045936~negative regulation of phosphate metabolic process | 6.26E-03 |
| GOTERM_BP_FAT | GO:0010563~negative regulation of phosphorus metabolic process | 6.26E-03 |
| GOTERM_BP_FAT | GO:0042325~regulation of phosphorylation | 2.02E-02 |
| GOTERM_BP_FAT | GO:0051174~regulation of phosphorus metabolic process | 2.30E-02 |
| GOTERM_BP_FAT | GO:0019220~regulation of phosphate metabolic process | 2.30E-02 |
| | | |
| | | |
| **Annotation Cluster 4** | **Enrichment Score: 1.9183400618864614** | |
| | | |
| Category | Term | PValue |
| GOTERM_BP_FAT | GO:0006974~response to DNA damage stimulus | 5.06E-05 |
| GOTERM_BP_FAT | GO:0042981~regulation of apoptosis | 2.93E-04 |
| GOTERM_BP_FAT | GO:0043067~regulation of programmed cell death | 3.21E-04 |
| GOTERM_BP_FAT | GO:0010941~regulation of cell death | 3.31E-04 |
| GOTERM_BP_FAT | GO:0043068~positive regulation of programmed cell death | 1.66E-03 |
| GOTERM_BP_FAT | GO:0043066~negative regulation of apoptosis | 1.68E-03 |
| GOTERM_BP_FAT | GO:0010942~positive regulation of cell death | 1.72E-03 |
| GOTERM_BP_FAT | GO:0043069~negative regulation of programmed cell death | 1.79E-03 |
| GOTERM_BP_FAT | GO:0060548~negative regulation of cell death | 1.82E-03 |
| GOTERM_BP_FAT | GO:0043523~regulation of neuron apoptosis | 2.85E-03 |
| GOTERM_BP_FAT | GO:0043065~positive regulation of apoptosis | 1.07E-02 |
| GOTERM_BP_FAT | GO:0048608~reproductive structure development | 6.23E-02 |
| GOTERM_BP_FAT | GO:0048610~reproductive cellular process | 7.79E-02 |
| GOTERM_BP_FAT | GO:0048232~male gamete generation | 1.39E-01 |

| | | |
|---|---|---|
| GOTERM_BP_FAT | GO:0007283~spermatogenesis | 1.39E-01 |
| GOTERM_BP_FAT | GO:0003006~reproductive developmental process | 1.71E-01 |
| GOTERM_BP_FAT | GO:0007276~gamete generation | 2.11E-01 |
| GOTERM_BP_FAT | GO:0019953~sexual reproduction | 2.59E-01 |
| GOTERM_BP_FAT | GO:0032504~multicellular organism reproduction | 3.29E-01 |
| GOTERM_BP_FAT | GO:0048609~reproductive process in a multicellular organism | 3.29E-01 |
| UP_SEQ_FEATURE | transmembrane region | 7.26E-01 |

## Drug-induced transcriptional module: RatLiver-7

### 52 genes, 18 chemicals

### Functional enrichment of genes:

No significant annotation clusters found.

## Drug-induced transcriptional module: RatLiver-8

### 26 genes, 40 chemicals

### Functional enrichment of genes:

| **Annotation Cluster 1** | **Enrichment Score: 2.6198261364306132** | |
|---|---|---|
| | | |
| Category | Term | PValue |
| GOTERM_BP_FAT | GO:0019941~modification-dependent protein catabolic process | 3.31E-04 |
| GOTERM_BP_FAT | GO:0043632~modification-dependent macromolecule catabolic process | 3.31E-04 |
| GOTERM_BP_FAT | GO:0051603~proteolysis involved in cellular protein catabolic process | 4.62E-04 |
| GOTERM_BP_FAT | GO:0044257~cellular protein catabolic process | 4.80E-04 |
| GOTERM_BP_FAT | GO:0030163~protein catabolic process | 6.06E-04 |
| GOTERM_BP_FAT | GO:0044265~cellular macromolecule catabolic process | 1.22E-03 |
| SP_PIR_KEYWORDS | ubl conjugation pathway | 1.88E-03 |
| GOTERM_BP_FAT | GO:0009057~macromolecule catabolic process | 2.02E-03 |
| GOTERM_CC_FAT | GO:0000151~ubiquitin ligase complex | 2.39E-03 |
| GOTERM_BP_FAT | GO:0032446~protein modification by small protein conjugation | 6.25E-03 |
| GOTERM_BP_FAT | GO:0070647~protein modification by small protein conjugation or removal | 9.42E-03 |
| GOTERM_BP_FAT | GO:0006508~proteolysis | 1.29E-02 |
| GOTERM_MF_FAT | GO:0019899~enzyme binding | 2.17E-02 |
| GOTERM_BP_FAT | GO:0010604~positive regulation of macromolecule metabolic process | 7.78E-02 |
| | | |
| | | |
| **Annotation Cluster 2** | **Enrichment Score: 2.2959735232514418** | |
| | | |
| Category | Term | PValue |
| GOTERM_BP_FAT | GO:0006281~DNA repair | 1.38E-03 |
| GOTERM_BP_FAT | GO:0006974~response to DNA damage stimulus | 3.61E-03 |
| GOTERM_BP_FAT | GO:0006259~DNA metabolic process | 8.58E-03 |
| GOTERM_BP_FAT | GO:0033554~cellular response to stress | 1.53E-02 |

## Drug-induced transcriptional module: RatLiver-9

### 41 genes, 22 chemicals

### Functional enrichment of genes:

| **Annotation Cluster 1** | **Enrichment Score: 7.15132925602242** | |
|---|---|---|
| | | |

| Category | Term | PValue |
|---|---|---|
| KEGG_PATHWAY | rno03010:Ribosome | 4.89E-14 |
| GOTERM_BP_FAT | GO:0006414~translational elongation | 2.56E-12 |
| SP_PIR_KEYWORDS | ribosomal protein | 1.20E-11 |
| SP_PIR_KEYWORDS | ribonucleoprotein | 6.73E-11 |
| SP_PIR_KEYWORDS | ribosome | 1.45E-10 |
| GOTERM_CC_FAT | GO:0022626~cytosolic ribosome | 2.11E-10 |
| GOTERM_CC_FAT | GO:0022627~cytosolic small ribosomal subunit | 1.62E-09 |
| GOTERM_MF_FAT | GO:0003735~structural constituent of ribosome | 6.11E-09 |
| GOTERM_CC_FAT | GO:0033279~ribosomal subunit | 7.03E-09 |
| GOTERM_CC_FAT | GO:0015935~small ribosomal subunit | 8.32E-09 |
| SP_PIR_KEYWORDS | protein biosynthesis | 2.59E-08 |
| GOTERM_CC_FAT | GO:0044445~cytosolic part | 4.75E-08 |
| GOTERM_CC_FAT | GO:0005840~ribosome | 6.23E-08 |
| GOTERM_BP_FAT | GO:0006412~translation | 1.24E-07 |
| GOTERM_MF_FAT | GO:0005198~structural molecule activity | 2.94E-06 |
| GOTERM_CC_FAT | GO:0030529~ribonucleoprotein complex | 3.14E-06 |
| GOTERM_CC_FAT | GO:0005829~cytosol | 4.40E-06 |
| GOTERM_CC_FAT | GO:0043232~intracellular non-membrane-bounded organelle | 9.70E-03 |
| GOTERM_CC_FAT | GO:0043228~non-membrane-bounded organelle | 9.70E-03 |
| GOTERM_CC_FAT | GO:0005730~nucleolus | 5.75E-02 |
| GOTERM_CC_FAT | GO:0031981~nuclear lumen | 1.78E-01 |
| | | |
| | | |
| **Annotation Cluster 2** | **Enrichment Score: 1.8173259408120002** | |
| | | |
| Category | Term | PValue |
| GOTERM_BP_FAT | GO:0006805~xenobiotic metabolic process | 1.92E-03 |
| GOTERM_BP_FAT | GO:0009410~response to xenobiotic stimulus | 2.43E-03 |
| KEGG_PATHWAY | rno00980:Metabolism of xenobiotics by cytochrome P450 | 1.90E-02 |
| KEGG_PATHWAY | rno00982:Drug metabolism | 2.68E-02 |
| GOTERM_CC_FAT | GO:0042175~nuclear envelope-endoplasmic reticulum network | 3.48E-02 |
| GOTERM_CC_FAT | GO:0012505~endomembrane system | 1.51E-01 |
| | | |
| | | |
| **Annotation Cluster 3** | **Enrichment Score: 1.6894639241179537** | |
| | | |
| Category | Term | PValue |
| GOTERM_BP_FAT | GO:0042254~ribosome biogenesis | 1.85E-03 |
| GOTERM_BP_FAT | GO:0022613~ribonucleoprotein complex biogenesis | 5.46E-03 |
| GOTERM_BP_FAT | GO:0006364~rRNA processing | 1.20E-02 |
| GOTERM_BP_FAT | GO:0016072~rRNA metabolic process | 1.27E-02 |
| GOTERM_BP_FAT | GO:0034470~ncRNA processing | 4.54E-02 |
| GOTERM_BP_FAT | GO:0034660~ncRNA metabolic process | 8.29E-02 |
| GOTERM_BP_FAT | GO:0006396~RNA processing | 2.58E-01 |

## Drug-induced transcriptional module: RatLiver-10

## 27 genes, 11 chemicals

## Functional enrichment of genes:

| **Annotation Cluster 1** | **Enrichment Score: 1.8710698730964943** | |
|---|---|---|
| | | |
| Category | Term | PValue |
| UP_SEQ_FEATURE | short sequence motif:Microbody targeting signal | 2.23E-03 |
| SP_PIR_KEYWORDS | peroxisome | 7.47E-03 |
| GOTERM_CC_FAT | GO:0042579~microbody | 1.16E-02 |
| GOTERM_CC_FAT | GO:0005777~peroxisome | 1.16E-02 |
| GOTERM_CC_FAT | GO:0005739~mitochondrion | 1.95E-01 |

## Drug-induced transcriptional module: RatLiver-11

### 32 genes, 28 chemicals

### Functional enrichment of genes:

No significant annotation clusters found.

## Drug-induced transcriptional module: RatLiver-12

### 59 genes, 24 chemicals

### Functional enrichment of genes:

| **Annotation Cluster 1** | **Enrichment Score: 9.922034005694815** | |
|---|---|---|
| | | |
| Category | Term | PValue |
| SP_PIR_KEYWORDS | lipid synthesis | 2.04E-16 |
| GOTERM_BP_FAT | GO:0008610~lipid biosynthetic process | 2.80E-16 |
| GOTERM_BP_FAT | GO:0016126~sterol biosynthetic process | 3.12E-16 |
| GOTERM_BP_FAT | GO:0006695~cholesterol biosynthetic process | 8.72E-15 |
| GOTERM_BP_FAT | GO:0016125~sterol metabolic process | 6.55E-14 |
| GOTERM_BP_FAT | GO:0008203~cholesterol metabolic process | 1.69E-12 |
| GOTERM_BP_FAT | GO:0006694~steroid biosynthetic process | 2.18E-12 |
| SP_PIR_KEYWORDS | Steroid biosynthesis | 3.41E-12 |
| GOTERM_BP_FAT | GO:0008202~steroid metabolic process | 1.58E-10 |
| KEGG_PATHWAY | rno00100:Steroid biosynthesis | 3.10E-10 |
| SP_PIR_KEYWORDS | sterol biosynthesis | 4.12E-10 |
| KEGG_PATHWAY | rno00900:Terpenoid backbone biosynthesis | 1.01E-08 |
| SP_PIR_KEYWORDS | Cholesterol biosynthesis | 1.05E-08 |
| GOTERM_BP_FAT | GO:0008299~isoprenoid biosynthetic process | 2.20E-06 |
| GOTERM_BP_FAT | GO:0006720~isoprenoid metabolic process | 4.23E-05 |
| SP_PIR_KEYWORDS | Isoprene biosynthesis | 3.64E-04 |
| SP_PIR_KEYWORDS | magnesium | 2.28E-02 |
| | | |
| | | |
| **Annotation Cluster 2** | **Enrichment Score: 3.6259050142621034** | |
| | | |
| Category | Term | PValue |
| KEGG_PATHWAY | rno00100:Steroid biosynthesis | 3.10E-10 |
| GOTERM_CC_FAT | GO:0005789~endoplasmic reticulum membrane | 1.42E-06 |
| GOTERM_CC_FAT | GO:0042175~nuclear envelope-endoplasmic reticulum network | 2.23E-06 |
| GOTERM_BP_FAT | GO:0055114~oxidation reduction | 4.01E-06 |
| GOTERM_CC_FAT | GO:0005783~endoplasmic reticulum | 5.85E-06 |
| GOTERM_CC_FAT | GO:0044432~endoplasmic reticulum part | 7.61E-06 |
| SP_PIR_KEYWORDS | oxidoreductase | 1.17E-04 |
| GOTERM_CC_FAT | GO:0012505~endomembrane system | 1.61E-04 |
| SP_PIR_KEYWORDS | endoplasmic reticulum | 1.65E-04 |
| GOTERM_CC_FAT | GO:0031090~organelle membrane | 2.20E-04 |
| SP_PIR_KEYWORDS | membrane | 1.37E-02 |
| SP_PIR_KEYWORDS | transmembrane | 2.42E-02 |
| UP_SEQ_FEATURE | transmembrane region | 2.64E-02 |
| GOTERM_CC_FAT | GO:0016021~integral to membrane | 3.51E-01 |
| GOTERM_CC_FAT | GO:0031224~intrinsic to membrane | 4.14E-01 |
| UP_SEQ_FEATURE | topological domain:Cytoplasmic | 6.37E-01 |
| | | |
| | | |
| **Annotation Cluster 3** | **Enrichment Score: 2.9162158446736655** | |

| | | |
|---|---|---|
| Category | Term | PValue |
| GOTERM_BP_FAT | GO:0006633~fatty acid biosynthetic process | 1.22E-04 |
| GOTERM_BP_FAT | GO:0006631~fatty acid metabolic process | 4.10E-04 |
| UP_SEQ_FEATURE | short sequence motif:Histidine box-1 | 6.32E-04 |
| UP_SEQ_FEATURE | short sequence motif:Histidine box-3 | 6.32E-04 |
| UP_SEQ_FEATURE | short sequence motif:Histidine box-2 | 6.32E-04 |
| GOTERM_BP_FAT | GO:0046394~carboxylic acid biosynthetic process | 1.61E-03 |
| GOTERM_BP_FAT | GO:0016053~organic acid biosynthetic process | 1.61E-03 |
| GOTERM_MF_FAT | GO:0005506~iron ion binding | 2.33E-03 |
| KEGG_PATHWAY | rno01040:Biosynthesis of unsaturated fatty acids | 8.60E-03 |
| SP_PIR_KEYWORDS | Fatty acid biosynthesis | 1.04E-02 |

## Drug-induced transcriptional module: RatLiver-13

### 24 genes, 47 chemicals

### Functional enrichment of genes:

| **Annotation Cluster 1** | **Enrichment Score: 1.451569173527932** | |
|---|---|---|
| | | |
| Category | Term | PValue |
| GOTERM_MF_FAT | GO:0000166~nucleotide binding | 5.87E-03 |
| GOTERM_MF_FAT | GO:0032555~purine ribonucleotide binding | 8.45E-03 |
| GOTERM_MF_FAT | GO:0032553~ribonucleotide binding | 8.48E-03 |
| GOTERM_MF_FAT | GO:0017076~purine nucleotide binding | 1.07E-02 |
| GOTERM_MF_FAT | GO:0005524~ATP binding | 1.49E-02 |
| GOTERM_MF_FAT | GO:0032559~adenyl ribonucleotide binding | 1.62E-02 |
| GOTERM_MF_FAT | GO:0030554~adenyl nucleotide binding | 2.05E-02 |
| GOTERM_MF_FAT | GO:0001883~purine nucleoside binding | 2.19E-02 |
| GOTERM_MF_FAT | GO:0001882~nucleoside binding | 2.27E-02 |
| SP_PIR_KEYWORDS | nucleotide-binding | 2.93E-02 |
| SP_PIR_KEYWORDS | atp-binding | 4.36E-02 |
| SP_PIR_KEYWORDS | kinase | 4.55E-02 |
| INTERPRO | IPR000719:Protein kinase, core | 1.03E-01 |
| GOTERM_BP_FAT | GO:0006796~phosphate metabolic process | 1.12E-01 |
| GOTERM_BP_FAT | GO:0006793~phosphorus metabolic process | 1.13E-01 |
| GOTERM_MF_FAT | GO:0004672~protein kinase activity | 1.55E-01 |
| GOTERM_BP_FAT | GO:0006468~protein amino acid phosphorylation | 2.15E-01 |
| GOTERM_BP_FAT | GO:0016310~phosphorylation | 2.66E-01 |

## Drug-induced transcriptional module: RatLiver-14

### 36 genes, 12 chemicals

### Functional enrichment of genes:

No significant annotation clusters found.

## Drug-induced transcriptional module: RatLiver-15

### 48 genes, 44 chemicals

### Functional enrichment of genes:

| **Annotation Cluster 1** | **Enrichment Score: 2.535177042882967** | |
|---|---|---|

| | | |
|---|---|---|
| Category | Term | PValue |
| GOTERM_CC_FAT | GO:0000267~cell fraction | 1.52E-05 |
| GOTERM_BP_FAT | GO:0015980~energy derivation by oxidation of organic compounds | 2.19E-04 |
| GOTERM_CC_FAT | GO:0005625~soluble fraction | 2.51E-04 |
| GOTERM_BP_FAT | GO:0019318~hexose metabolic process | 2.41E-03 |
| GOTERM_BP_FAT | GO:0005996~monosaccharide metabolic process | 3.78E-03 |
| GOTERM_BP_FAT | GO:0006091~generation of precursor metabolites and energy | 4.27E-03 |
| GOTERM_CC_FAT | GO:0005626~insoluble fraction | 6.91E-03 |
| GOTERM_BP_FAT | GO:0006006~glucose metabolic process | 1.30E-02 |
| GOTERM_CC_FAT | GO:0005624~membrane fraction | 2.00E-02 |
| GOTERM_CC_FAT | GO:0005792~microsome | 4.54E-02 |
| GOTERM_CC_FAT | GO:0042598~vesicular fraction | 4.88E-02 |
| | | |
| | | |
| **Annotation Cluster 2** | **Enrichment Score: 1.8788171521789185** | |
| | | |
| Category | Term | PValue |
| GOTERM_BP_FAT | GO:0051186~cofactor metabolic process | 2.37E-03 |
| GOTERM_BP_FAT | GO:0046496~nicotinamide nucleotide metabolic process | 5.88E-03 |
| GOTERM_BP_FAT | GO:0006769~nicotinamide metabolic process | 6.16E-03 |
| GOTERM_BP_FAT | GO:0019362~pyridine nucleotide metabolic process | 6.45E-03 |
| GOTERM_BP_FAT | GO:0009820~alkaloid metabolic process | 6.45E-03 |
| GOTERM_BP_FAT | GO:0006733~oxidoreduction coenzyme metabolic process | 9.32E-03 |
| GOTERM_MF_FAT | GO:0048037~cofactor binding | 1.01E-02 |
| GOTERM_BP_FAT | GO:0006732~coenzyme metabolic process | 1.01E-02 |
| GOTERM_BP_FAT | GO:0043603~cellular amide metabolic process | 1.11E-02 |
| GOTERM_MF_FAT | GO:0050662~coenzyme binding | 2.56E-02 |
| GOTERM_BP_FAT | GO:0019748~secondary metabolic process | 2.69E-02 |
| GOTERM_BP_FAT | GO:0055114~oxidation reduction | 2.69E-02 |
| GOTERM_CC_FAT | GO:0005739~mitochondrion | 5.43E-01 |
| | | |
| | | |
| **Annotation Cluster 3** | **Enrichment Score: 1.7615960894581595** | |
| | | |
| Category | Term | PValue |
| GOTERM_MF_FAT | GO:0048037~cofactor binding | 1.01E-02 |
| GOTERM_BP_FAT | GO:0009063~cellular amino acid catabolic process | 1.15E-02 |
| GOTERM_BP_FAT | GO:0009310~amine catabolic process | 1.47E-02 |
| GOTERM_BP_FAT | GO:0016054~organic acid catabolic process | 3.03E-02 |
| GOTERM_BP_FAT | GO:0046395~carboxylic acid catabolic process | 3.03E-02 |

## Drug-induced transcriptional module: RatLiver-16

## 38 genes, 65 chemicals

## Functional enrichment of genes:

| **Annotation Cluster 1** | **Enrichment Score: 2.4520233227557857** | |
|---|---|---|
| | | |
| Category | Term | PValue |
| SP_PIR_KEYWORDS | lipid metabolism | 1.25E-04 |
| SP_PIR_KEYWORDS | fatty acid metabolism | 7.15E-03 |
| GOTERM_BP_FAT | GO:0006631~fatty acid metabolic process | 4.91E-02 |
| | | |
| | | |
| **Annotation Cluster 2** | **Enrichment Score: 2.2566587319239972** | |
| | | |
| Category | Term | PValue |
| GOTERM_CC_FAT | GO:0031090~organelle membrane | 1.11E-04 |

| | | |
|---|---|---|
| GOTERM_CC_FAT | GO:0031967~organelle envelope | 1.36E-04 |
| GOTERM_CC_FAT | GO:0031975~envelope | 1.42E-04 |
| GOTERM_CC_FAT | GO:0044429~mitochondrial part | 7.45E-04 |
| GOTERM_CC_FAT | GO:0031966~mitochondrial membrane | 1.04E-03 |
| GOTERM_CC_FAT | GO:0005740~mitochondrial envelope | 1.41E-03 |
| GOTERM_CC_FAT | GO:0005739~mitochondrion | 4.27E-03 |
| KEGG_PATHWAY | rno00071:Fatty acid metabolism | 6.98E-03 |
| GOTERM_CC_FAT | GO:0005741~mitochondrial outer membrane | 1.45E-02 |
| GOTERM_CC_FAT | GO:0031968~organelle outer membrane | 1.95E-02 |
| GOTERM_CC_FAT | GO:0019867~outer membrane | 2.10E-02 |
| GOTERM_CC_FAT | GO:0012505~endomembrane system | 5.06E-02 |
| GOTERM_CC_FAT | GO:0005743~mitochondrial inner membrane | 1.30E-01 |
| GOTERM_CC_FAT | GO:0019866~organelle inner membrane | 1.44E-01 |
| SP_PIR_KEYWORDS | mitochondrion | 3.61E-01 |
| | | |
| | | |
| **Annotation Cluster 3** | **Enrichment Score: 1.8973204664838073** | |
| | | |
| Category | Term | PValue |
| GOTERM_CC_FAT | GO:0005792~microsome | 1.13E-06 |
| GOTERM_CC_FAT | GO:0042598~vesicular fraction | 1.38E-06 |
| GOTERM_CC_FAT | GO:0005624~membrane fraction | 1.27E-05 |
| GOTERM_CC_FAT | GO:0005626~insoluble fraction | 1.95E-05 |
| SP_PIR_KEYWORDS | lipid metabolism | 1.25E-04 |
| GOTERM_CC_FAT | GO:0000267~cell fraction | 1.37E-04 |
| GOTERM_CC_FAT | GO:0005783~endoplasmic reticulum | 2.54E-02 |
| GOTERM_CC_FAT | GO:0012505~endomembrane system | 5.06E-02 |
| GOTERM_CC_FAT | GO:0005789~endoplasmic reticulum membrane | 7.82E-02 |
| GOTERM_CC_FAT | GO:0042175~nuclear envelope-endoplasmic reticulum network | 8.67E-02 |
| SP_PIR_KEYWORDS | endoplasmic reticulum | 1.04E-01 |
| GOTERM_CC_FAT | GO:0044432~endoplasmic reticulum part | 1.15E-01 |
| SP_PIR_KEYWORDS | membrane | 2.01E-01 |
| UP_SEQ_FEATURE | topological domain:Cytoplasmic | 3.37E-01 |
| GOTERM_CC_FAT | GO:0005886~plasma membrane | 6.44E-01 |
| UP_SEQ_FEATURE | transmembrane region | 6.83E-01 |
| SP_PIR_KEYWORDS | transmembrane | 7.27E-01 |
| UP_SEQ_FEATURE | topological domain:Extracellular | 9.06E-01 |
| GOTERM_CC_FAT | GO:0016021~integral to membrane | 9.08E-01 |
| GOTERM_CC_FAT | GO:0031224~intrinsic to membrane | 9.26E-01 |

## Drug-induced transcriptional module: RatLiver-17

## 22 genes, 31 chemicals

## Functional enrichment of genes:

| **Annotation Cluster 1** | **Enrichment Score: 2.62701551348663** | |
|---|---|---|
| | | |
| Category | Term | PValue |
| GOTERM_CC_FAT | GO:0005783~endoplasmic reticulum | 6.91E-07 |
| KEGG_PATHWAY | rno00980:Metabolism of xenobiotics by cytochrome P450 | 1.45E-06 |
| GOTERM_MF_FAT | GO:0009055~electron carrier activity | 3.67E-06 |
| SP_PIR_KEYWORDS | endoplasmic reticulum | 5.22E-06 |
| GOTERM_BP_FAT | GO:0009404~toxin metabolic process | 1.86E-05 |
| SP_PIR_KEYWORDS | microsome | 2.52E-05 |
| GOTERM_BP_FAT | GO:0042537~benzene and derivative metabolic process | 3.47E-05 |
| GOTERM_BP_FAT | GO:0055114~oxidation reduction | 3.47E-05 |
| KEGG_PATHWAY | rno00830:Retinol metabolism | 8.97E-05 |
| GOTERM_BP_FAT | GO:0019748~secondary metabolic process | 1.41E-04 |
| SP_PIR_KEYWORDS | chromoprotein | 1.99E-04 |

| | | |
|---|---|---|
| GOTERM_CC_FAT | GO:0005789~endoplasmic reticulum membrane | 2.68E-04 |
| GOTERM_CC_FAT | GO:0042175~nuclear envelope-endoplasmic reticulum network | 3.38E-04 |
| GOTERM_BP_FAT | GO:0006805~xenobiotic metabolic process | 3.39E-04 |
| GOTERM_BP_FAT | GO:0010033~response to organic substance | 3.53E-04 |
| GOTERM_BP_FAT | GO:0009410~response to xenobiotic stimulus | 4.30E-04 |
| GOTERM_BP_FAT | GO:0006778~porphyrin metabolic process | 4.96E-04 |
| GOTERM_BP_FAT | GO:0033013~tetrapyrrole metabolic process | 4.96E-04 |
| GOTERM_MF_FAT | GO:0020037~heme binding | 6.11E-04 |
| SP_PIR_KEYWORDS | metalloprotein | 6.18E-04 |
| SP_PIR_KEYWORDS | oxidoreductase | 6.26E-04 |
| GOTERM_CC_FAT | GO:0005792~microsome | 6.33E-04 |
| GOTERM_CC_FAT | GO:0044432~endoplasmic reticulum part | 6.33E-04 |
| COG_ONTOLOGY | Secondary metabolites biosynthesis, transport, and catabolism | 6.74E-04 |
| GOTERM_MF_FAT | GO:0046906~tetrapyrrole binding | 6.86E-04 |
| UP_SEQ_FEATURE | metal ion-binding site:Iron (heme axial ligand) | 7.09E-04 |
| GOTERM_CC_FAT | GO:0042598~vesicular fraction | 7.09E-04 |
| GOTERM_MF_FAT | GO:0070330~aromatase activity | 8.27E-04 |
| GOTERM_CC_FAT | GO:0019898~extrinsic to membrane | 1.17E-03 |
| GOTERM_MF_FAT | GO:0016712~oxidoreductase activity, acting on paired donors, with incorporation or reduction of molecular oxygen, reduced flavin or flavoprotein as one donor, and incorporation of one atom of oxygen | 1.22E-03 |
| SP_PIR_KEYWORDS | heme | 1.25E-03 |
| KEGG_PATHWAY | rno00380:Tryptophan metabolism | 2.01E-03 |
| KEGG_PATHWAY | rno00140:Steroid hormone biosynthesis | 2.01E-03 |
| GOTERM_CC_FAT | GO:0031090~organelle membrane | 2.33E-03 |
| GOTERM_BP_FAT | GO:0034754~cellular hormone metabolic process | 2.34E-03 |
| PIR_SUPERFAMILY | PIRSF000045:cytochrome P450 CYP2D6 | 2.74E-03 |
| INTERPRO | IPR002401:Cytochrome P450, E-class, group I | 3.40E-03 |
| SP_PIR_KEYWORDS | electron transfer | 3.62E-03 |
| GOTERM_CC_FAT | GO:0005624~membrane fraction | 4.04E-03 |
| GOTERM_MF_FAT | GO:0005506~iron ion binding | 4.17E-03 |
| GOTERM_CC_FAT | GO:0005626~insoluble fraction | 5.07E-03 |
| INTERPRO | IPR017973:Cytochrome P450, C-terminal region | 5.69E-03 |
| GOTERM_BP_FAT | GO:0042493~response to drug | 5.71E-03 |
| INTERPRO | IPR017972:Cytochrome P450, conserved site | 5.86E-03 |
| INTERPRO | IPR001128:Cytochrome P450 | 6.21E-03 |
| GOTERM_BP_FAT | GO:0042445~hormone metabolic process | 6.83E-03 |
| SP_PIR_KEYWORDS | iron | 7.37E-03 |
| SP_PIR_KEYWORDS | Monooxygenase | 9.54E-03 |
| SP_PIR_KEYWORDS | nadp | 1.33E-02 |
| GOTERM_BP_FAT | GO:0010817~regulation of hormone levels | 1.38E-02 |
| GOTERM_CC_FAT | GO:0000267~cell fraction | 1.43E-02 |
| GOTERM_CC_FAT | GO:0012505~endomembrane system | 1.57E-02 |
| GOTERM_BP_FAT | GO:0051186~cofactor metabolic process | 2.21E-02 |
| GOTERM_BP_FAT | GO:0007584~response to nutrient | 2.30E-02 |
| GOTERM_BP_FAT | GO:0014070~response to organic cyclic substance | 2.40E-02 |
| SP_PIR_KEYWORDS | membrane | 3.20E-02 |
| GOTERM_BP_FAT | GO:0031667~response to nutrient levels | 4.10E-02 |
| GOTERM_CC_FAT | GO:0044429~mitochondrial part | 4.48E-02 |
| GOTERM_BP_FAT | GO:0009991~response to extracellular stimulus | 4.64E-02 |
| GOTERM_CC_FAT | GO:0031967~organelle envelope | 5.20E-02 |
| GOTERM_CC_FAT | GO:0031975~envelope | 5.30E-02 |
| GOTERM_CC_FAT | GO:0031966~mitochondrial membrane | 1.10E-01 |
| GOTERM_CC_FAT | GO:0005740~mitochondrial envelope | 1.23E-01 |
| SP_PIR_KEYWORDS | transmembrane protein | 1.97E-01 |
| SP_PIR_KEYWORDS | metal-binding | 2.22E-01 |
| GOTERM_MF_FAT | GO:0046872~metal ion binding | 2.26E-01 |
| GOTERM_MF_FAT | GO:0043169~cation binding | 2.35E-01 |
| GOTERM_MF_FAT | GO:0043167~ion binding | 2.47E-01 |
| GOTERM_CC_FAT | GO:0005739~mitochondrion | 3.15E-01 |
| GOTERM_MF_FAT | GO:0046914~transition metal ion binding | 3.79E-01 |
| | | |

| | | |
|---|---|---|
| **Annotation Cluster 2** | **Enrichment Score: 2.1978829898039955** | |
| | | |
| Category | Term | PValue |
| KEGG_PATHWAY | rno00980:Metabolism of xenobiotics by cytochrome P450 | 1.45E-06 |
| GOTERM_BP_FAT | GO:0010033~response to organic substance | 3.53E-04 |
| KEGG_PATHWAY | rno00982:Drug metabolism | 5.56E-03 |
| GOTERM_BP_FAT | GO:0042493~response to drug | 5.71E-03 |
| GOTERM_BP_FAT | GO:0048545~response to steroid hormone stimulus | 4.34E-02 |
| GOTERM_BP_FAT | GO:0009725~response to hormone stimulus | 1.16E-01 |
| GOTERM_BP_FAT | GO:0009719~response to endogenous stimulus | 1.40E-01 |
| SP_PIR_KEYWORDS | acetylation | 2.28E-01 |

## Drug-induced transcriptional module: RatLiver-18

### 25 genes, 34 chemicals

### Functional enrichment of genes:

No significant annotation clusters found.

## Drug-induced transcriptional module: RatLiver-19

### 26 genes, 79 chemicals

### Functional enrichment of genes:

No significant annotation clusters found.

## Drug-induced transcriptional module: RatLiver-20

### 21 genes, 60 chemicals

### Functional enrichment of genes:

| **Annotation Cluster 1** | **Enrichment Score: 1.9660417256744969** | |
|---|---|---|
| | | |
| Category | Term | PValue |
| SP_PIR_KEYWORDS | ligase | 8.51E-04 |
| KEGG_PATHWAY | rno00970:Aminoacyl-tRNA biosynthesis | 1.74E-03 |
| GOTERM_MF_FAT | GO:0004812~aminoacyl-tRNA ligase activity | 2.39E-03 |
| GOTERM_MF_FAT | GO:0016875~ligase activity, forming carbon-oxygen bonds | 2.39E-03 |
| GOTERM_MF_FAT | GO:0016876~ligase activity, forming aminoacyl-tRNA and related compounds | 2.39E-03 |
| GOTERM_CC_FAT | GO:0005829~cytosol | 3.22E-03 |
| GOTERM_MF_FAT | GO:0030554~adenyl nucleotide binding | 5.92E-03 |
| GOTERM_MF_FAT | GO:0001883~purine nucleoside binding | 6.47E-03 |
| GOTERM_MF_FAT | GO:0001882~nucleoside binding | 6.73E-03 |
| GOTERM_MF_FAT | GO:0017076~purine nucleotide binding | 1.52E-02 |
| SP_PIR_KEYWORDS | atp-binding | 1.69E-02 |
| GOTERM_MF_FAT | GO:0005524~ATP binding | 1.99E-02 |
| GOTERM_MF_FAT | GO:0032559~adenyl ribonucleotide binding | 2.15E-02 |
| GOTERM_MF_FAT | GO:0000166~nucleotide binding | 3.45E-02 |
| SP_PIR_KEYWORDS | nucleotide-binding | 4.31E-02 |
| GOTERM_MF_FAT | GO:0032555~purine ribonucleotide binding | 4.72E-02 |
| GOTERM_MF_FAT | GO:0032553~ribonucleotide binding | 4.73E-02 |
| UP_SEQ_FEATURE | nucleotide phosphate-binding region:ATP | 7.44E-02 |

| | | |
|---|---|---|
| GOTERM_BP_FAT | GO:0006412~translation | 9.69E-02 |

## Drug-induced transcriptional module: RatLiver-21

### 39 genes, 89 chemicals

### Functional enrichment of genes:

No significant annotation clusters found.

## Drug-induced transcriptional module: RatLiver-22

### 88 genes, 16 chemicals

### Functional enrichment of genes:

| **Annotation Cluster 1** | **Enrichment Score: 2.71662955752944** | |
|---|---|---|
| | | |
| Category | Term | PValue |
| GOTERM_BP_FAT | GO:0042330~taxis | 1.03E-05 |
| GOTERM_BP_FAT | GO:0006935~chemotaxis | 1.03E-05 |
| GOTERM_BP_FAT | GO:0050900~leukocyte migration | 3.79E-04 |
| GOTERM_BP_FAT | GO:0030595~leukocyte chemotaxis | 1.42E-03 |
| GOTERM_BP_FAT | GO:0007626~locomotory behavior | 1.57E-03 |
| GOTERM_BP_FAT | GO:0060326~cell chemotaxis | 1.78E-03 |
| GOTERM_BP_FAT | GO:0007610~behavior | 3.92E-03 |
| GOTERM_BP_FAT | GO:0016477~cell migration | 4.68E-03 |
| GOTERM_BP_FAT | GO:0006954~inflammatory response | 9.94E-03 |
| GOTERM_BP_FAT | GO:0051674~localization of cell | 1.39E-02 |
| GOTERM_BP_FAT | GO:0048870~cell motility | 1.39E-02 |
| GOTERM_MF_FAT | GO:0005125~cytokine activity | 2.19E-02 |
| GOTERM_BP_FAT | GO:0006928~cell motion | 3.94E-02 |
| | | |
| | | |
| **Annotation Cluster 2** | **Enrichment Score: 2.5377688456635803** | |
| | | |
| Category | Term | PValue |
| GOTERM_BP_FAT | GO:0051240~positive regulation of multicellular organismal process | 1.22E-04 |
| KEGG_PATHWAY | rno04623:Cytosolic DNA-sensing pathway | 3.58E-04 |
| KEGG_PATHWAY | rno04621:NOD-like receptor signaling pathway | 1.12E-03 |
| GOTERM_BP_FAT | GO:0001817~regulation of cytokine production | 2.42E-03 |
| GOTERM_BP_FAT | GO:0001819~positive regulation of cytokine production | 1.04E-02 |
| GOTERM_BP_FAT | GO:0051046~regulation of secretion | 1.61E-02 |
| GOTERM_BP_FAT | GO:0060341~regulation of cellular localization | 8.62E-02 |
| | | |
| | | |
| **Annotation Cluster 3** | **Enrichment Score: 2.456157125398821** | |
| | | |
| Category | Term | PValue |
| GOTERM_BP_FAT | GO:0006955~immune response | 1.43E-07 |
| GOTERM_BP_FAT | GO:0045321~leukocyte activation | 5.50E-07 |
| GOTERM_BP_FAT | GO:0002684~positive regulation of immune system process | 1.20E-06 |
| GOTERM_BP_FAT | GO:0001775~cell activation | 1.76E-06 |
| GOTERM_BP_FAT | GO:0050870~positive regulation of T cell activation | 4.86E-06 |
| GOTERM_BP_FAT | GO:0046649~lymphocyte activation | 7.33E-06 |
| GOTERM_BP_FAT | GO:0051094~positive regulation of developmental process | 1.62E-05 |
| GOTERM_BP_FAT | GO:0051251~positive regulation of lymphocyte activation | 2.52E-05 |
| GOTERM_BP_FAT | GO:0002696~positive regulation of leukocyte activation | 4.16E-05 |
| GOTERM_BP_FAT | GO:0050867~positive regulation of cell activation | 5.13E-05 |

| | | |
|---|---|---|
| GOTERM_BP_FAT | GO:0042110~T cell activation | 5.67E-05 |
| GOTERM_BP_FAT | GO:0050863~regulation of T cell activation | 5.67E-05 |
| GOTERM_BP_FAT | GO:0051249~regulation of lymphocyte activation | 2.27E-04 |
| GOTERM_BP_FAT | GO:0002694~regulation of leukocyte activation | 4.14E-04 |
| GOTERM_BP_FAT | GO:0050865~regulation of cell activation | 5.19E-04 |
| GOTERM_CC_FAT | GO:0009897~external side of plasma membrane | 5.33E-04 |
| GOTERM_BP_FAT | GO:0045582~positive regulation of T cell differentiation | 1.10E-03 |
| GOTERM_BP_FAT | GO:0045621~positive regulation of lymphocyte differentiation | 1.31E-03 |
| GOTERM_BP_FAT | GO:0030098~lymphocyte differentiation | 2.57E-03 |
| GOTERM_BP_FAT | GO:0045580~regulation of T cell differentiation | 3.01E-03 |
| GOTERM_BP_FAT | GO:0045597~positive regulation of cell differentiation | 4.10E-03 |
| GOTERM_BP_FAT | GO:0045619~regulation of lymphocyte differentiation | 4.89E-03 |
| GOTERM_BP_FAT | GO:0045058~T cell selection | 5.17E-03 |
| GOTERM_BP_FAT | GO:0002521~leukocyte differentiation | 6.65E-03 |
| GOTERM_BP_FAT | GO:0030217~T cell differentiation | 7.36E-03 |
| KEGG_PATHWAY | rno05340:Primary immunodeficiency | 2.66E-02 |
| GOTERM_BP_FAT | GO:0030097~hemopoiesis | 4.76E-02 |
| GOTERM_BP_FAT | GO:0050671~positive regulation of lymphocyte proliferation | 4.93E-02 |
| GOTERM_BP_FAT | GO:0032946~positive regulation of mononuclear cell proliferation | 5.08E-02 |
| GOTERM_BP_FAT | GO:0070665~positive regulation of leukocyte proliferation | 5.39E-02 |
| GOTERM_CC_FAT | GO:0005768~endosome | 5.57E-02 |
| GOTERM_BP_FAT | GO:0048534~hemopoietic or lymphoid organ development | 6.43E-02 |
| GOTERM_BP_FAT | GO:0002520~immune system development | 7.36E-02 |
| GOTERM_BP_FAT | GO:0044093~positive regulation of molecular function | 8.52E-02 |
| GOTERM_BP_FAT | GO:0050670~regulation of lymphocyte proliferation | 9.88E-02 |
| GOTERM_BP_FAT | GO:0032944~regulation of mononuclear cell proliferation | 1.01E-01 |
| GOTERM_BP_FAT | GO:0070663~regulation of leukocyte proliferation | 1.05E-01 |
| GOTERM_BP_FAT | GO:0043085~positive regulation of catalytic activity | 1.30E-01 |
| SP_PIR_KEYWORDS | immune response | 1.47E-01 |
| GOTERM_BP_FAT | GO:0042325~regulation of phosphorylation | 2.21E-01 |
| GOTERM_BP_FAT | GO:0051174~regulation of phosphorus metabolic process | 2.41E-01 |
| GOTERM_BP_FAT | GO:0019220~regulation of phosphate metabolic process | 2.41E-01 |
| GOTERM_BP_FAT | GO:0045860~positive regulation of protein kinase activity | 3.21E-01 |
| GOTERM_BP_FAT | GO:0033674~positive regulation of kinase activity | 3.39E-01 |
| GOTERM_BP_FAT | GO:0051347~positive regulation of transferase activity | 3.58E-01 |
| GOTERM_BP_FAT | GO:0045859~regulation of protein kinase activity | 4.90E-01 |
| GOTERM_BP_FAT | GO:0043549~regulation of kinase activity | 5.16E-01 |
| GOTERM_BP_FAT | GO:0051338~regulation of transferase activity | 5.42E-01 |

## Drug-induced transcriptional module: RatLiver-23

## 45 genes,  25 chemicals

## Functional enrichment of genes:

| **Annotation Cluster 1** | **Enrichment Score: 3.0393296689433926** | |
|---|---|---|
| | | |
| Category | Term | PValue |
| GOTERM_BP_FAT | GO:0010557~positive regulation of macromolecule biosynthetic process | 3.72E-07 |
| GOTERM_BP_FAT | GO:0031328~positive regulation of cellular biosynthetic process | 6.65E-07 |
| GOTERM_BP_FAT | GO:0009891~positive regulation of biosynthetic process | 8.06E-07 |
| GOTERM_BP_FAT | GO:0006350~transcription | 9.27E-07 |
| SP_PIR_KEYWORDS | dna-binding | 1.38E-06 |
| UP_SEQ_FEATURE | domain:Leucine-zipper | 2.92E-06 |
| GOTERM_BP_FAT | GO:0045449~regulation of transcription | 3.28E-06 |
| GOTERM_BP_FAT | GO:0010604~positive regulation of macromolecule metabolic process | 6.59E-06 |
| GOTERM_MF_FAT | GO:0030528~transcription regulator activity | 8.53E-06 |
| INTERPRO | IPR004827:Basic-leucine zipper (bZIP) transcription factor | 1.64E-05 |
| UP_SEQ_FEATURE | DNA-binding region:Basic motif | 1.76E-05 |
| GOTERM_MF_FAT | GO:0003677~DNA binding | 1.97E-05 |

| | | |
|---|---|---|
| GOTERM_BP_FAT | GO:0010033~response to organic substance | 2.18E-05 |
| GOTERM_MF_FAT | GO:0003700~transcription factor activity | 3.29E-05 |
| GOTERM_BP_FAT | GO:0045941~positive regulation of transcription | 3.86E-05 |
| GOTERM_MF_FAT | GO:0043565~sequence-specific DNA binding | 3.92E-05 |
| GOTERM_BP_FAT | GO:0010628~positive regulation of gene expression | 4.66E-05 |
| SMART | SM00338:BRLZ | 4.79E-05 |
| SP_PIR_KEYWORDS | transcription regulation | 6.55E-05 |
| GOTERM_BP_FAT | GO:0006355~regulation of transcription, DNA-dependent | 7.20E-05 |
| GOTERM_BP_FAT | GO:0045893~positive regulation of transcription, DNA-dependent | 8.44E-05 |
| INTERPRO | IPR011616:bZIP transcription factor, bZIP-1 | 8.58E-05 |
| KEGG_PATHWAY | rno04010:MAPK signaling pathway | 8.71E-05 |
| GOTERM_MF_FAT | GO:0016563~transcription activator activity | 8.80E-05 |
| GOTERM_BP_FAT | GO:0051254~positive regulation of RNA metabolic process | 8.97E-05 |
| GOTERM_BP_FAT | GO:0051252~regulation of RNA metabolic process | 9.26E-05 |
| SP_PIR_KEYWORDS | DNA binding | 9.52E-05 |
| GOTERM_BP_FAT | GO:0045935~positive regulation of nucleobase, nucleoside, nucleotide and nucleic acid metabolic process | 1.06E-04 |
| SP_PIR_KEYWORDS | Transcription | 1.23E-04 |
| GOTERM_MF_FAT | GO:0003690~double-stranded DNA binding | 1.28E-04 |
| GOTERM_BP_FAT | GO:0051173~positive regulation of nitrogen compound metabolic process | 1.32E-04 |
| SP_PIR_KEYWORDS | nucleus | 1.74E-04 |
| GOTERM_MF_FAT | GO:0043566~structure-specific DNA binding | 5.57E-04 |
| GOTERM_BP_FAT | GO:0006357~regulation of transcription from RNA polymerase II promoter | 5.75E-04 |
| GOTERM_BP_FAT | GO:0006351~transcription, DNA-dependent | 1.06E-03 |
| GOTERM_BP_FAT | GO:0032774~RNA biosynthetic process | 1.15E-03 |
| GOTERM_BP_FAT | GO:0009612~response to mechanical stimulus | 1.92E-03 |
| GOTERM_MF_FAT | GO:0046983~protein dimerization activity | 2.44E-03 |
| GOTERM_CC_FAT | GO:0031981~nuclear lumen | 2.45E-03 |
| GOTERM_BP_FAT | GO:0006366~transcription from RNA polymerase II promoter | 3.60E-03 |
| GOTERM_BP_FAT | GO:0045944~positive regulation of transcription from RNA polymerase II promoter | 7.45E-03 |
| GOTERM_BP_FAT | GO:0051726~regulation of cell cycle | 7.98E-03 |
| GOTERM_BP_FAT | GO:0009628~response to abiotic stimulus | 8.72E-03 |
| GOTERM_CC_FAT | GO:0070013~intracellular organelle lumen | 9.29E-03 |
| SP_PIR_KEYWORDS | Proto-oncogene | 1.01E-02 |
| GOTERM_CC_FAT | GO:0043233~organelle lumen | 1.13E-02 |
| GOTERM_CC_FAT | GO:0031974~membrane-enclosed lumen | 1.30E-02 |
| KEGG_PATHWAY | rno05200:Pathways in cancer | 1.34E-02 |
| GOTERM_CC_FAT | GO:0005654~nucleoplasm | 1.40E-02 |
| GOTERM_BP_FAT | GO:0051591~response to cAMP | 1.80E-02 |
| GOTERM_BP_FAT | GO:0031668~cellular response to extracellular stimulus | 1.86E-02 |
| GOTERM_MF_FAT | GO:0046982~protein heterodimerization activity | 2.23E-02 |
| GOTERM_BP_FAT | GO:0009314~response to radiation | 2.34E-02 |
| KEGG_PATHWAY | rno05210:Colorectal cancer | 2.45E-02 |
| GOTERM_BP_FAT | GO:0014070~response to organic cyclic substance | 3.02E-02 |
| GOTERM_BP_FAT | GO:0034097~response to cytokine stimulus | 5.05E-02 |
| GOTERM_CC_FAT | GO:0044451~nucleoplasm part | 6.19E-02 |
| GOTERM_BP_FAT | GO:0007611~learning or memory | 6.74E-02 |
| GOTERM_CC_FAT | GO:0043232~intracellular non-membrane-bounded organelle | 7.98E-02 |
| GOTERM_CC_FAT | GO:0043228~non-membrane-bounded organelle | 7.98E-02 |
| GOTERM_CC_FAT | GO:0005730~nucleolus | 1.14E-01 |
| GOTERM_CC_FAT | GO:0044427~chromosomal part | 1.40E-01 |
| GOTERM_CC_FAT | GO:0005694~chromosome | 1.71E-01 |
| GOTERM_BP_FAT | GO:0042493~response to drug | 2.85E-01 |
| GOTERM_CC_FAT | GO:0005856~cytoskeleton | 3.25E-01 |
| GOTERM_BP_FAT | GO:0007610~behavior | 3.99E-01 |
| GOTERM_CC_FAT | GO:0044430~cytoskeletal part | 4.42E-01 |
| GOTERM_BP_FAT | GO:0050877~neurological system process | 8.68E-01 |
| GOTERM_BP_FAT | GO:0050890~cognition | 9.08E-01 |
| | | |
| | | |

| Annotation Cluster 2 | Enrichment Score: 2.1806475525196998 | |
|---|---|---|
| | | |
| Category | Term | PValue |
| GOTERM_BP_FAT | GO:0042127~regulation of cell proliferation | 1.08E-03 |
| GOTERM_BP_FAT | GO:0009968~negative regulation of signal transduction | 3.80E-03 |
| GOTERM_BP_FAT | GO:0010648~negative regulation of cell communication | 6.22E-03 |
| GOTERM_BP_FAT | GO:0008285~negative regulation of cell proliferation | 1.00E-02 |
| GOTERM_MF_FAT | GO:0008134~transcription factor binding | 4.88E-02 |
| | | |
| | | |
| **Annotation Cluster 3** | **Enrichment Score: 2.006098158940342** | |
| | | |
| Category | Term | PValue |
| GOTERM_BP_FAT | GO:0010962~regulation of glucan biosynthetic process | 1.67E-03 |
| GOTERM_BP_FAT | GO:0005979~regulation of glycogen biosynthetic process | 1.67E-03 |
| GOTERM_BP_FAT | GO:0032885~regulation of polysaccharide biosynthetic process | 1.67E-03 |
| GOTERM_BP_FAT | GO:0032881~regulation of polysaccharide metabolic process | 2.25E-03 |
| GOTERM_BP_FAT | GO:0043467~regulation of generation of precursor metabolites and energy | 4.73E-03 |
| GOTERM_BP_FAT | GO:0043255~regulation of carbohydrate biosynthetic process | 4.73E-03 |
| GOTERM_BP_FAT | GO:0010906~regulation of glucose metabolic process | 8.42E-03 |
| GOTERM_BP_FAT | GO:0010675~regulation of cellular carbohydrate metabolic process | 1.04E-02 |
| GOTERM_BP_FAT | GO:0006109~regulation of carbohydrate metabolic process | 1.13E-02 |
| GOTERM_BP_FAT | GO:0031329~regulation of cellular catabolic process | 2.02E-02 |
| GOTERM_BP_FAT | GO:0009894~regulation of catabolic process | 4.12E-02 |
| GOTERM_CC_FAT | GO:0000267~cell fraction | 4.97E-02 |
| KEGG_PATHWAY | rno04910:Insulin signaling pathway | 5.97E-02 |
| GOTERM_MF_FAT | GO:0019899~enzyme binding | 1.43E-01 |

## Drug-induced transcriptional module: RatLiver-24

## 40 genes, 27 chemicals

## Functional enrichment of genes:

| Annotation Cluster 1 | Enrichment Score: 4.150332351989817 | |
|---|---|---|
| | | |
| Category | Term | PValue |
| GOTERM_BP_FAT | GO:0006414~translational elongation | 1.27E-10 |
| SP_PIR_KEYWORDS | protein biosynthesis | 3.37E-08 |
| KEGG_PATHWAY | rno03010:Ribosome | 1.44E-07 |
| SP_PIR_KEYWORDS | ribosome | 2.57E-07 |
| GOTERM_CC_FAT | GO:0022626~cytosolic ribosome | 3.01E-07 |
| GOTERM_CC_FAT | GO:0044445~cytosolic part | 5.59E-07 |
| GOTERM_CC_FAT | GO:0022627~cytosolic small ribosomal subunit | 3.46E-06 |
| GOTERM_CC_FAT | GO:0033279~ribosomal subunit | 3.48E-06 |
| SP_PIR_KEYWORDS | ribosomal protein | 6.03E-06 |
| GOTERM_CC_FAT | GO:0015935~small ribosomal subunit | 9.98E-06 |
| GOTERM_BP_FAT | GO:0006412~translation | 1.47E-05 |
| SP_PIR_KEYWORDS | ribonucleoprotein | 1.62E-05 |
| GOTERM_MF_FAT | GO:0003735~structural constituent of ribosome | 9.89E-05 |
| GOTERM_CC_FAT | GO:0005840~ribosome | 3.14E-04 |
| GOTERM_CC_FAT | GO:0005829~cytosol | 8.00E-04 |
| SP_PIR_KEYWORDS | rrna-binding | 8.45E-04 |
| GOTERM_MF_FAT | GO:0019843~rRNA binding | 2.19E-03 |
| GOTERM_CC_FAT | GO:0030529~ribonucleoprotein complex | 2.86E-03 |
| GOTERM_MF_FAT | GO:0005198~structural molecule activity | 3.24E-03 |
| GOTERM_CC_FAT | GO:0043228~non-membrane-bounded organelle | 6.58E-02 |
| GOTERM_CC_FAT | GO:0043232~intracellular non-membrane-bounded organelle | 6.58E-02 |
| SP_PIR_KEYWORDS | rna-binding | 1.71E-01 |

| | | |
|---|---|---|
| GOTERM_MF_FAT | GO:0003723~RNA binding | 3.22E-01 |
| SP_PIR_KEYWORDS | cytoplasm | 5.27E-01 |

## Drug-induced transcriptional module: RatLiver-25

### 23 genes, 20 chemicals

### Functional enrichment of genes:

| Annotation Cluster 1 | Enrichment Score: 1.9298011792749328 | |
|---|---|---|
| | | |
| Category | Term | PValue |
| SP_PIR_KEYWORDS | fatty acid metabolism | 2.58E-03 |
| SP_PIR_KEYWORDS | lipid metabolism | 1.06E-02 |
| GOTERM_CC_FAT | GO:0005739~mitochondrion | 2.22E-02 |
| GOTERM_BP_FAT | GO:0006631~fatty acid metabolic process | 3.12E-02 |

## Drug-induced transcriptional module: RatLiver-26

### 26 genes, 14 chemicals

### Functional enrichment of genes:

No significant annotation clusters found.

## Drug-induced transcriptional module: RatLiver-27

### 22 genes, 25 chemicals

### Functional enrichment of genes:

| Annotation Cluster 1 | Enrichment Score: 2.8979186104431416 | |
|---|---|---|
| | | |
| Category | Term | PValue |
| GOTERM_BP_FAT | GO:0000375~RNA splicing, via transesterification reactions | 4.17E-04 |
| GOTERM_BP_FAT | GO:0000377~RNA splicing, via transesterification reactions with bulged adenosine as nucleophile | 4.17E-04 |
| GOTERM_BP_FAT | GO:0000398~nuclear mRNA splicing, via spliceosome | 4.17E-04 |
| GOTERM_BP_FAT | GO:0008380~RNA splicing | 1.27E-03 |
| GOTERM_BP_FAT | GO:0006397~mRNA processing | 2.01E-03 |
| GOTERM_BP_FAT | GO:0016071~mRNA metabolic process | 3.05E-03 |
| GOTERM_BP_FAT | GO:0006396~RNA processing | 9.12E-03 |

## Drug-induced transcriptional module: RatLiver-28

### 41 genes, 16 chemicals

### Functional enrichment of genes:

No significant annotation clusters found.

## Drug-induced transcriptional module: RatLiver-29

### 48 genes, 13 chemicals

### Functional enrichment of genes:

No significant annotation clusters found.

## Drug-induced transcriptional module: RatLiver-30

### 22 genes, 47 chemicals

### Functional enrichment of genes:

No significant annotation clusters found.

## Drug-induced transcriptional module: RatLiver-31

### 27 genes, 15 chemicals

### Functional enrichment of genes:

| **Annotation Cluster 1** | **Enrichment Score: 1.9545606369998447** | |
|---|---|---|
| | | |
| Category | Term | PValue |
| GOTERM_CC_FAT | GO:0005694~chromosome | 1.86E-03 |
| GOTERM_CC_FAT | GO:0044427~chromosomal part | 1.18E-02 |
| GOTERM_CC_FAT | GO:0043232~intracellular non-membrane-bounded organelle | 1.58E-02 |
| GOTERM_CC_FAT | GO:0043228~non-membrane-bounded organelle | 1.58E-02 |
| GOTERM_CC_FAT | GO:0000785~chromatin | 3.07E-02 |
| | | |
| | | |
| **Annotation Cluster 2** | **Enrichment Score: 1.6956363561526215** | |
| | | |
| Category | Term | PValue |
| GOTERM_CC_FAT | GO:0031941~filamentous actin | 3.14E-04 |
| GOTERM_CC_FAT | GO:0005884~actin filament | 1.85E-03 |
| GOTERM_CC_FAT | GO:0015629~actin cytoskeleton | 4.28E-02 |
| GOTERM_CC_FAT | GO:0044430~cytoskeletal part | 3.01E-01 |
| GOTERM_CC_FAT | GO:0005856~cytoskeleton | 4.44E-01 |

## Drug-induced transcriptional module: RatLiver-32

### 48 genes, 11 chemicals

### Functional enrichment of genes:

| **Annotation Cluster 1** | **Enrichment Score: 1.5236254657995059** | |
|---|---|---|
| | | |
| Category | Term | PValue |
| INTERPRO | IPR013069:BTB/POZ | 1.45E-02 |
| INTERPRO | IPR011333:BTB/POZ fold | 3.47E-02 |
| INTERPRO | IPR000210:BTB/POZ-like | 3.54E-02 |
| SMART | SM00225:BTB | 4.51E-02 |
| | | |
| | | |
| **Annotation Cluster 2** | **Enrichment Score: 1.5101971650811603** | |
| | | |
| Category | Term | PValue |
| GOTERM_BP_FAT | GO:0051223~regulation of protein transport | 2.58E-02 |
| GOTERM_BP_FAT | GO:0070201~regulation of establishment of protein localization | 2.82E-02 |
| GOTERM_BP_FAT | GO:0032880~regulation of protein localization | 4.04E-02 |
| | | |
| | | |
| **Annotation Cluster 3** | **Enrichment Score: 1.3976261570647686** | |

| | | |
|---|---|---|
| | | |
| Category | Term | PValue |
| GOTERM_BP_FAT | GO:0006508~proteolysis | 1.07E-02 |
| GOTERM_BP_FAT | GO:0019941~modification-dependent protein catabolic process | 3.26E-02 |
| GOTERM_BP_FAT | GO:0043632~modification-dependent macromolecule catabolic process | 3.26E-02 |
| GOTERM_BP_FAT | GO:0070647~protein modification by small protein conjugation or removal | 3.55E-02 |
| GOTERM_BP_FAT | GO:0051603~proteolysis involved in cellular protein catabolic process | 4.07E-02 |
| GOTERM_BP_FAT | GO:0044257~cellular protein catabolic process | 4.17E-02 |
| GOTERM_BP_FAT | GO:0030163~protein catabolic process | 4.86E-02 |
| GOTERM_BP_FAT | GO:0044265~cellular macromolecule catabolic process | 7.60E-02 |
| GOTERM_BP_FAT | GO:0009057~macromolecule catabolic process | 1.04E-01 |

## Drug-induced transcriptional module: RatLiver-33

## 26 genes, 112 chemicals

## Functional enrichment of genes:

| **Annotation Cluster 1** | **Enrichment Score: 2.269214353722061** | |
|---|---|---|
| | | |
| Category | Term | PValue |
| GOTERM_BP_FAT | GO:0040012~regulation of locomotion | 2.75E-04 |
| GOTERM_BP_FAT | GO:0040017~positive regulation of locomotion | 6.94E-04 |
| GOTERM_BP_FAT | GO:0030334~regulation of cell migration | 2.77E-03 |
| GOTERM_BP_FAT | GO:0051270~regulation of cell motion | 3.88E-03 |
| GOTERM_BP_FAT | GO:0030335~positive regulation of cell migration | 1.09E-02 |
| GOTERM_BP_FAT | GO:0051272~positive regulation of cell motion | 1.28E-02 |
| GOTERM_BP_FAT | GO:0008361~regulation of cell size | 4.04E-02 |
| GOTERM_BP_FAT | GO:0032535~regulation of cellular component size | 6.01E-02 |
| | | |
| | | |
| **Annotation Cluster 2** | **Enrichment Score: 1.5174092756641167** | |
| | | |
| Category | Term | PValue |
| GOTERM_BP_FAT | GO:0044092~negative regulation of molecular function | 1.32E-02 |
| KEGG_PATHWAY | rno04062:Chemokine signaling pathway | 3.56E-02 |
| GOTERM_BP_FAT | GO:0010648~negative regulation of cell communication | 5.97E-02 |

## Drug-induced transcriptional module: RatLiver-34

## 22 genes, 11 chemicals

## Functional enrichment of genes:

| **Annotation Cluster 1** | **Enrichment Score: 2.7530656991198064** | |
|---|---|---|
| | | |
| Category | Term | PValue |
| UP_SEQ_FEATURE | domain:LIM zinc-binding 1 | 1.56E-05 |
| UP_SEQ_FEATURE | domain:LIM zinc-binding 2 | 1.56E-05 |
| GOTERM_BP_FAT | GO:0060537~muscle tissue development | 2.66E-05 |
| GOTERM_BP_FAT | GO:0007517~muscle organ development | 5.88E-05 |
| SMART | SM00132:LIM | 6.79E-05 |
| SP_PIR_KEYWORDS | LIM domain | 7.65E-05 |
| INTERPRO | IPR001781:Zinc finger, LIM-type | 1.29E-04 |
| GOTERM_CC_FAT | GO:0030017~sarcomere | 1.35E-04 |
| GOTERM_BP_FAT | GO:0007507~heart development | 1.44E-04 |
| GOTERM_CC_FAT | GO:0044449~contractile fiber part | 1.98E-04 |
| GOTERM_CC_FAT | GO:0030016~myofibril | 2.47E-04 |

| | | |
|---|---|---|
| UP_SEQ_FEATURE | domain:LIM zinc-binding 3 | 2.56E-04 |
| GOTERM_CC_FAT | GO:0043292~contractile fiber | 3.11E-04 |
| GOTERM_BP_FAT | GO:0014706~striated muscle tissue development | 6.28E-04 |
| GOTERM_CC_FAT | GO:0005856~cytoskeleton | 7.05E-04 |
| GOTERM_CC_FAT | GO:0030018~Z disc | 1.44E-03 |
| GOTERM_CC_FAT | GO:0031674~I band | 2.08E-03 |
| GOTERM_BP_FAT | GO:0048738~cardiac muscle tissue development | 2.60E-03 |
| SP_PIR_KEYWORDS | cytoplasm | 1.28E-02 |
| SP_PIR_KEYWORDS | metal-binding | 1.95E-02 |
| GOTERM_CC_FAT | GO:0043232~intracellular non-membrane-bounded organelle | 2.04E-02 |
| GOTERM_CC_FAT | GO:0043228~non-membrane-bounded organelle | 2.04E-02 |
| GOTERM_CC_FAT | GO:0044430~cytoskeletal part | 5.70E-02 |
| GOTERM_MF_FAT | GO:0046872~metal ion binding | 1.14E-01 |
| GOTERM_MF_FAT | GO:0043169~cation binding | 1.21E-01 |
| GOTERM_MF_FAT | GO:0043167~ion binding | 1.30E-01 |
| SP_PIR_KEYWORDS | zinc | 1.75E-01 |
| GOTERM_MF_FAT | GO:0046914~transition metal ion binding | 3.08E-01 |
| GOTERM_MF_FAT | GO:0008270~zinc ion binding | 3.80E-01 |
| | | |
| | | |
| **Annotation Cluster 2** | **Enrichment Score: 2.391471604892135** | |
| | | |
| Category | Term | PValue |
| GOTERM_BP_FAT | GO:0060537~muscle tissue development | 2.66E-05 |
| GOTERM_BP_FAT | GO:0007517~muscle organ development | 5.88E-05 |
| GOTERM_BP_FAT | GO:0014706~striated muscle tissue development | 6.28E-04 |
| GOTERM_BP_FAT | GO:0006874~cellular calcium ion homeostasis | 1.20E-03 |
| GOTERM_BP_FAT | GO:0055074~calcium ion homeostasis | 1.29E-03 |
| GOTERM_BP_FAT | GO:0006875~cellular metal ion homeostasis | 1.53E-03 |
| GOTERM_BP_FAT | GO:0055065~metal ion homeostasis | 1.70E-03 |
| GOTERM_BP_FAT | GO:0030005~cellular di-, tri-valent inorganic cation homeostasis | 2.35E-03 |
| GOTERM_BP_FAT | GO:0055066~di-, tri-valent inorganic cation homeostasis | 2.72E-03 |
| GOTERM_BP_FAT | GO:0030003~cellular cation homeostasis | 3.29E-03 |
| GOTERM_BP_FAT | GO:0006937~regulation of muscle contraction | 3.35E-03 |
| GOTERM_BP_FAT | GO:0055080~cation homeostasis | 4.57E-03 |
| GOTERM_BP_FAT | GO:0044057~regulation of system process | 7.89E-03 |
| GOTERM_BP_FAT | GO:0006873~cellular ion homeostasis | 1.18E-02 |
| GOTERM_BP_FAT | GO:0055082~cellular chemical homeostasis | 1.23E-02 |
| GOTERM_BP_FAT | GO:0050801~ion homeostasis | 1.48E-02 |
| GOTERM_BP_FAT | GO:0019725~cellular homeostasis | 2.03E-02 |
| GOTERM_BP_FAT | GO:0048514~blood vessel morphogenesis | 2.45E-02 |
| GOTERM_BP_FAT | GO:0048878~chemical homeostasis | 2.53E-02 |
| GOTERM_BP_FAT | GO:0001568~blood vessel development | 3.63E-02 |
| GOTERM_BP_FAT | GO:0001944~vasculature development | 3.83E-02 |
| GOTERM_BP_FAT | GO:0042592~homeostatic process | 6.46E-02 |

## Drug-induced transcriptional module: RatLiver-35

## 33 genes,  15 chemicals

## Functional enrichment of genes:

| **Annotation Cluster 1** | **Enrichment Score: 1.498135478092717** | |
|---|---|---|
| | | |
| Category | Term | PValue |
| GOTERM_MF_FAT | GO:0042623~ATPase activity, coupled | 1.00E-02 |
| GOTERM_MF_FAT | GO:0042626~ATPase activity, coupled to transmembrane movement of substances | 2.00E-02 |
| GOTERM_MF_FAT | GO:0043492~ATPase activity, coupled to movement of substances | 2.08E-02 |
| GOTERM_MF_FAT | GO:0016820~hydrolase activity, acting on acid anhydrides, catalyzing | 2.20E-02 |

| | | |
|---|---|---|
| | transmembrane movement of substances | |
| GOTERM_MF_FAT | GO:0016887~ATPase activity | 2.21E-02 |
| GOTERM_MF_FAT | GO:0015405~P-P-bond-hydrolysis-driven transmembrane transporter activity | 2.45E-02 |
| GOTERM_MF_FAT | GO:0015399~primary active transmembrane transporter activity | 2.45E-02 |
| SP_PIR_KEYWORDS | transport | 8.53E-01 |
| | | |
| | | |
| **Annotation Cluster 2** | **Enrichment Score: 1.4216338143066967** | |
| | | |
| Category | Term | PValue |
| GOTERM_CC_FAT | GO:0070013~intracellular organelle lumen | 1.50E-02 |
| GOTERM_CC_FAT | GO:0043233~organelle lumen | 1.81E-02 |
| GOTERM_CC_FAT | GO:0031974~membrane-enclosed lumen | 2.08E-02 |
| GOTERM_CC_FAT | GO:0031981~nuclear lumen | 3.66E-01 |
| | | |
| | | |
| **Annotation Cluster 3** | **Enrichment Score: 1.350617731778604** | |
| | | |
| Category | Term | PValue |
| GOTERM_BP_FAT | GO:0044271~nitrogen compound biosynthetic process | 7.47E-03 |
| GOTERM_BP_FAT | GO:0009165~nucleotide biosynthetic process | 7.77E-02 |
| GOTERM_BP_FAT | GO:0034654~nucleobase, nucleoside, nucleotide and nucleic acid biosynthetic process | 8.26E-02 |
| GOTERM_BP_FAT | GO:0034404~nucleobase, nucleoside and nucleotide biosynthetic process | 8.26E-02 |

## Drug-induced transcriptional module: RatLiver-36

## 88 genes, 37 chemicals

## Functional enrichment of genes:

| | | |
|---|---|---|
| **Annotation Cluster 1** | **Enrichment Score: 10.927986479827775** | |
| | | |
| Category | Term | PValue |
| GOTERM_CC_FAT | GO:0043232~intracellular non-membrane-bounded organelle | 1.44E-14 |
| GOTERM_CC_FAT | GO:0043228~non-membrane-bounded organelle | 1.44E-14 |
| GOTERM_CC_FAT | GO:0015630~microtubule cytoskeleton | 4.30E-13 |
| GOTERM_CC_FAT | GO:0044430~cytoskeletal part | 1.17E-08 |
| GOTERM_CC_FAT | GO:0005856~cytoskeleton | 2.21E-07 |
| | | |
| | | |
| **Annotation Cluster 2** | **Enrichment Score: 10.307922853480648** | |
| | | |
| Category | Term | PValue |
| GOTERM_BP_FAT | GO:0007049~cell cycle | 1.36E-19 |
| GOTERM_BP_FAT | GO:0022402~cell cycle process | 2.43E-17 |
| GOTERM_BP_FAT | GO:0022403~cell cycle phase | 3.14E-17 |
| GOTERM_BP_FAT | GO:0000279~M phase | 2.15E-15 |
| GOTERM_BP_FAT | GO:0000278~mitotic cell cycle | 3.06E-11 |
| GOTERM_BP_FAT | GO:0000087~M phase of mitotic cell cycle | 4.05E-10 |
| GOTERM_BP_FAT | GO:0051301~cell division | 1.10E-08 |
| SP_PIR_KEYWORDS | cell division | 1.93E-08 |
| GOTERM_BP_FAT | GO:0000280~nuclear division | 1.10E-07 |
| GOTERM_BP_FAT | GO:0007067~mitosis | 1.10E-07 |
| SP_PIR_KEYWORDS | cell cycle | 1.53E-07 |
| GOTERM_BP_FAT | GO:0048285~organelle fission | 1.73E-07 |
| SP_PIR_KEYWORDS | mitosis | 5.26E-05 |

| | | |
|---|---|---|
| | | |
| | | |
| **Annotation Cluster 3** | **Enrichment Score: 7.502095627267259** | |
| | | |
| Category | Term | PValue |
| GOTERM_CC_FAT | GO:0005694~chromosome | 1.44E-13 |
| GOTERM_CC_FAT | GO:0044427~chromosomal part | 2.64E-13 |
| GOTERM_CC_FAT | GO:0031981~nuclear lumen | 1.77E-07 |
| GOTERM_CC_FAT | GO:0031974~membrane-enclosed lumen | 4.26E-06 |
| GOTERM_CC_FAT | GO:0070013~intracellular organelle lumen | 6.84E-06 |
| GOTERM_CC_FAT | GO:0043233~organelle lumen | 1.15E-05 |
| GOTERM_CC_FAT | GO:0005654~nucleoplasm | 1.34E-05 |

## Drug-induced transcriptional module: RatLiver-37

### 54 genes, 31 chemicals

### Functional enrichment of genes:

No significant annotation clusters found.

## Drug-induced transcriptional module: RatLiver-38

### 47 genes, 21 chemicals

### Functional enrichment of genes:

| **Annotation Cluster 1** | **Enrichment Score: 1.8767781236376273** | |
|---|---|---|
| | | |
| Category | Term | PValue |
| GOTERM_BP_FAT | GO:0051094~positive regulation of developmental process | 1.88E-03 |
| GOTERM_BP_FAT | GO:0051240~positive regulation of multicellular organismal process | 3.48E-02 |
| GOTERM_BP_FAT | GO:0045597~positive regulation of cell differentiation | 3.59E-02 |
| | | |
| | | |
| **Annotation Cluster 2** | **Enrichment Score: 1.4739045915319766** | |
| | | |
| Category | Term | PValue |
| INTERPRO | IPR013069:BTB/POZ | 1.70E-02 |
| INTERPRO | IPR011333:BTB/POZ fold | 4.03E-02 |
| INTERPRO | IPR000210:BTB/POZ-like | 4.12E-02 |
| SMART | SM00225:BTB | 4.51E-02 |

## Drug-induced transcriptional module: RatLiver-39

### 50 genes, 15 chemicals

### Functional enrichment of genes:

| **Annotation Cluster 1** | **Enrichment Score: 1.3291071285300828** | |
|---|---|---|
| | | |
| Category | Term | PValue |
| GOTERM_BP_FAT | GO:0015031~protein transport | 1.37E-03 |
| GOTERM_BP_FAT | GO:0045184~establishment of protein localization | 1.43E-03 |

| | | |
|---|---|---|
| GOTERM_BP_FAT | GO:0008104~protein localization | 3.42E-03 |
| GOTERM_BP_FAT | GO:0046907~intracellular transport | 4.94E-03 |
| GOTERM_CC_FAT | GO:0005794~Golgi apparatus | 5.31E-03 |
| GOTERM_BP_FAT | GO:0048193~Golgi vesicle transport | 1.60E-02 |
| GOTERM_BP_FAT | GO:0016192~vesicle-mediated transport | 1.75E-02 |
| GOTERM_BP_FAT | GO:0006887~exocytosis | 2.93E-02 |
| SP_PIR_KEYWORDS | coiled coil | 3.41E-02 |
| GOTERM_CC_FAT | GO:0031410~cytoplasmic vesicle | 5.89E-02 |
| GOTERM_CC_FAT | GO:0031982~vesicle | 7.01E-02 |
| GOTERM_BP_FAT | GO:0032940~secretion by cell | 8.18E-02 |
| SP_PIR_KEYWORDS | cytoplasmic vesicle | 9.82E-02 |
| GOTERM_BP_FAT | GO:0046903~secretion | 1.26E-01 |
| GOTERM_BP_FAT | GO:0006886~intracellular protein transport | 1.41E-01 |
| GOTERM_BP_FAT | GO:0016044~membrane organization | 1.44E-01 |
| GOTERM_BP_FAT | GO:0034613~cellular protein localization | 1.68E-01 |
| GOTERM_BP_FAT | GO:0070727~cellular macromolecule localization | 1.70E-01 |
| SP_PIR_KEYWORDS | protein transport | 2.13E-01 |
| GOTERM_CC_FAT | GO:0044459~plasma membrane part | 2.48E-01 |
| GOTERM_CC_FAT | GO:0031224~intrinsic to membrane | 9.28E-01 |
| SP_PIR_KEYWORDS | membrane | 9.83E-01 |

## Drug-induced transcriptional module: RatLiver-40

## 90 genes, 12 chemicals

## Functional enrichment of genes:

| **Annotation Cluster 1** | **Enrichment Score: 2.2274658082648884** | |
|---|---|---|
| | | |
| Category | Term | PValue |
| GOTERM_MF_FAT | GO:0016887~ATPase activity | 1.93E-04 |
| GOTERM_MF_FAT | GO:0042623~ATPase activity, coupled | 2.68E-04 |
| SP_PIR_KEYWORDS | helicase | 2.11E-03 |
| SP_PIR_KEYWORDS | nucleotide-binding | 4.69E-03 |
| GOTERM_MF_FAT | GO:0017076~purine nucleotide binding | 4.70E-03 |
| GOTERM_MF_FAT | GO:0032555~purine ribonucleotide binding | 7.79E-03 |
| GOTERM_MF_FAT | GO:0032553~ribonucleotide binding | 7.84E-03 |
| GOTERM_MF_FAT | GO:0004386~helicase activity | 8.47E-03 |
| GOTERM_MF_FAT | GO:0000166~nucleotide binding | 1.04E-02 |
| SP_PIR_KEYWORDS | atp-binding | 1.19E-02 |
| GOTERM_MF_FAT | GO:0030554~adenyl nucleotide binding | 1.51E-02 |
| GOTERM_MF_FAT | GO:0001883~purine nucleoside binding | 1.72E-02 |
| GOTERM_MF_FAT | GO:0001882~nucleoside binding | 1.83E-02 |
| GOTERM_MF_FAT | GO:0005524~ATP binding | 2.14E-02 |
| GOTERM_MF_FAT | GO:0032559~adenyl ribonucleotide binding | 2.45E-02 |
| | | |
| | | |
| **Annotation Cluster 2** | **Enrichment Score: 2.1861810176559757** | |
| | | |
| Category | Term | PValue |
| GOTERM_MF_FAT | GO:0016887~ATPase activity | 1.93E-04 |
| GOTERM_MF_FAT | GO:0042623~ATPase activity, coupled | 2.68E-04 |
| SP_PIR_KEYWORDS | helicase | 2.11E-03 |
| GOTERM_MF_FAT | GO:0004386~helicase activity | 8.47E-03 |
| SMART | SM00487:DEXDc | 9.93E-03 |
| SMART | SM00490:HELICc | 1.27E-02 |
| INTERPRO | IPR014001:DEAD-like helicase, N-terminal | 1.97E-02 |
| INTERPRO | IPR014021:Helicase, superfamily 1 and 2, ATP-binding | 1.97E-02 |
| INTERPRO | IPR001650:DNA/RNA helicase, C-terminal | 2.50E-02 |
| GOTERM_MF_FAT | GO:0008026~ATP-dependent helicase activity | 2.81E-02 |

| | | |
|---|---|---|
| GOTERM_MF_FAT | GO:0070035~purine NTP-dependent helicase activity | 2.81E-02 |

## Drug-induced transcriptional module: RatLiver-41

### 86 genes,  24 chemicals

### Functional enrichment of genes:

| **Annotation Cluster 1** | **Enrichment Score: 2.2054695178260886** | |
|---|---|---|
| | | |
| Category | Term | PValue |
| GOTERM_BP_FAT | GO:0015031~protein transport | 1.85E-04 |
| GOTERM_BP_FAT | GO:0045184~establishment of protein localization | 1.99E-04 |
| GOTERM_BP_FAT | GO:0008104~protein localization | 7.79E-04 |
| GOTERM_BP_FAT | GO:0006886~intracellular protein transport | 1.02E-02 |
| GOTERM_BP_FAT | GO:0034613~cellular protein localization | 1.58E-02 |
| GOTERM_BP_FAT | GO:0070727~cellular macromolecule localization | 1.62E-02 |
| GOTERM_BP_FAT | GO:0046907~intracellular transport | 2.56E-02 |
| GOTERM_MF_FAT | GO:0008565~protein transporter activity | 4.33E-02 |
| SP_PIR_KEYWORDS | protein transport | 1.71E-01 |
| | | |
| | | |
| **Annotation Cluster 2** | **Enrichment Score: 2.0356553285867167** | |
| | | |
| Category | Term | PValue |
| GOTERM_CC_FAT | GO:0005769~early endosome | 3.44E-04 |
| GOTERM_CC_FAT | GO:0005768~endosome | 4.13E-03 |
| GOTERM_BP_FAT | GO:0016192~vesicle-mediated transport | 5.15E-02 |
| KEGG_PATHWAY | rno04144:Endocytosis | 9.87E-02 |
| | | |
| | | |
| **Annotation Cluster 3** | **Enrichment Score: 1.8002355816952795** | |
| | | |
| Category | Term | PValue |
| GOTERM_MF_FAT | GO:0016881~acid-amino acid ligase activity | 1.10E-03 |
| GOTERM_MF_FAT | GO:0004842~ubiquitin-protein ligase activity | 1.22E-03 |
| GOTERM_MF_FAT | GO:0016879~ligase activity, forming carbon-nitrogen bonds | 2.25E-03 |
| GOTERM_MF_FAT | GO:0019787~small conjugating protein ligase activity | 3.64E-03 |
| GOTERM_BP_FAT | GO:0043632~modification-dependent macromolecule catabolic process | 9.92E-03 |
| GOTERM_BP_FAT | GO:0019941~modification-dependent protein catabolic process | 9.92E-03 |
| GOTERM_BP_FAT | GO:0044265~cellular macromolecule catabolic process | 1.03E-02 |
| GOTERM_BP_FAT | GO:0051603~proteolysis involved in cellular protein catabolic process | 1.41E-02 |
| GOTERM_BP_FAT | GO:0044257~cellular protein catabolic process | 1.46E-02 |
| SP_PIR_KEYWORDS | ubl conjugation pathway | 1.57E-02 |
| GOTERM_BP_FAT | GO:0030163~protein catabolic process | 1.87E-02 |
| GOTERM_BP_FAT | GO:0009057~macromolecule catabolic process | 1.91E-02 |
| GOTERM_BP_FAT | GO:0006508~proteolysis | 2.54E-02 |
| GOTERM_BP_FAT | GO:0016567~protein ubiquitination | 5.53E-02 |
| GOTERM_BP_FAT | GO:0032446~protein modification by small protein conjugation | 7.42E-02 |
| GOTERM_BP_FAT | GO:0070647~protein modification by small protein conjugation or removal | 1.07E-01 |
| KEGG_PATHWAY | rno04120:Ubiquitin mediated proteolysis | 1.54E-01 |
| GOTERM_BP_FAT | GO:0006511~ubiquitin-dependent protein catabolic process | 1.79E-01 |

## Drug-induced transcriptional module: RatLiver-42

### 54 genes,  11 chemicals

### Functional enrichment of genes:

| Annotation Cluster 1 | Enrichment Score: 3.0810207319883665 | |
|---|---|---|
| | | |
| Category | Term | PValue |
| GOTERM_CC_FAT | GO:0005654~nucleoplasm | 2.10E-04 |
| GOTERM_CC_FAT | GO:0031981~nuclear lumen | 5.10E-04 |
| GOTERM_CC_FAT | GO:0070013~intracellular organelle lumen | 1.06E-03 |
| GOTERM_CC_FAT | GO:0044451~nucleoplasm part | 1.06E-03 |
| GOTERM_CC_FAT | GO:0043233~organelle lumen | 1.46E-03 |
| GOTERM_CC_FAT | GO:0031974~membrane-enclosed lumen | 1.85E-03 |
| | | |
| | | |
| **Annotation Cluster 2** | **Enrichment Score: 2.0744481175420155** | |
| | | |
| Category | Term | PValue |
| GOTERM_MF_FAT | GO:0032555~purine ribonucleotide binding | 3.79E-03 |
| GOTERM_MF_FAT | GO:0032553~ribonucleotide binding | 3.81E-03 |
| SP_PIR_KEYWORDS | nucleotide-binding | 5.29E-03 |
| GOTERM_MF_FAT | GO:0017076~purine nucleotide binding | 5.64E-03 |
| GOTERM_MF_FAT | GO:0005524~ATP binding | 6.61E-03 |
| GOTERM_MF_FAT | GO:0032559~adenyl ribonucleotide binding | 7.59E-03 |
| GOTERM_MF_FAT | GO:0000166~nucleotide binding | 8.88E-03 |
| GOTERM_MF_FAT | GO:0030554~adenyl nucleotide binding | 1.13E-02 |
| GOTERM_MF_FAT | GO:0001883~purine nucleoside binding | 1.27E-02 |
| GOTERM_MF_FAT | GO:0001882~nucleoside binding | 1.34E-02 |
| SP_PIR_KEYWORDS | atp-binding | 4.10E-02 |
| | | |
| | | |
| **Annotation Cluster 3** | **Enrichment Score: 1.9275761597176784** | |
| | | |
| Category | Term | PValue |
| GOTERM_BP_FAT | GO:0070647~protein modification by small protein conjugation or removal | 6.40E-04 |
| GOTERM_BP_FAT | GO:0016567~protein ubiquitination | 2.48E-03 |
| GOTERM_BP_FAT | GO:0032446~protein modification by small protein conjugation | 3.99E-03 |
| GOTERM_MF_FAT | GO:0016879~ligase activity, forming carbon-nitrogen bonds | 2.23E-02 |
| GOTERM_MF_FAT | GO:0004842~ubiquitin-protein ligase activity | 3.78E-02 |
| GOTERM_MF_FAT | GO:0019787~small conjugating protein ligase activity | 6.44E-02 |
| GOTERM_MF_FAT | GO:0016881~acid-amino acid ligase activity | 9.35E-02 |

## Drug-induced transcriptional module: RatLiver-43

## 141 genes, 32 chemicals

## Functional enrichment of genes:

| Annotation Cluster 1 | Enrichment Score: 2.5015487875003153 | |
|---|---|---|
| | | |
| Category | Term | PValue |
| GOTERM_BP_FAT | GO:0010629~negative regulation of gene expression | 2.05E-04 |
| GOTERM_BP_FAT | GO:0045892~negative regulation of transcription, DNA-dependent | 2.58E-04 |
| GOTERM_BP_FAT | GO:0010604~positive regulation of macromolecule metabolic process | 2.73E-04 |
| GOTERM_BP_FAT | GO:0051253~negative regulation of RNA metabolic process | 2.85E-04 |
| GOTERM_BP_FAT | GO:0045449~regulation of transcription | 2.86E-04 |
| GOTERM_BP_FAT | GO:0016481~negative regulation of transcription | 4.05E-04 |
| GOTERM_BP_FAT | GO:0010605~negative regulation of macromolecule metabolic process | 5.84E-04 |
| GOTERM_BP_FAT | GO:0000122~negative regulation of transcription from RNA polymerase II promoter | 6.41E-04 |
| GOTERM_BP_FAT | GO:0031328~positive regulation of cellular biosynthetic process | 7.89E-04 |
| GOTERM_BP_FAT | GO:0051252~regulation of RNA metabolic process | 8.08E-04 |
| GOTERM_BP_FAT | GO:0009891~positive regulation of biosynthetic process | 9.40E-04 |

| | | |
|---|---|---|
| GOTERM_BP_FAT | GO:0045934~negative regulation of nucleobase, nucleoside, nucleotide and nucleic acid metabolic process | 9.50E-04 |
| GOTERM_BP_FAT | GO:0045893~positive regulation of transcription, DNA-dependent | 1.04E-03 |
| GOTERM_BP_FAT | GO:0051172~negative regulation of nitrogen compound metabolic process | 1.08E-03 |
| GOTERM_BP_FAT | GO:0051254~positive regulation of RNA metabolic process | 1.12E-03 |
| GOTERM_BP_FAT | GO:0045941~positive regulation of transcription | 1.13E-03 |
| GOTERM_MF_FAT | GO:0030528~transcription regulator activity | 1.23E-03 |
| GOTERM_BP_FAT | GO:0010628~positive regulation of gene expression | 1.39E-03 |
| GOTERM_BP_FAT | GO:0051173~positive regulation of nitrogen compound metabolic process | 1.43E-03 |
| GOTERM_BP_FAT | GO:0010557~positive regulation of macromolecule biosynthetic process | 1.47E-03 |
| GOTERM_BP_FAT | GO:0006355~regulation of transcription, DNA-dependent | 1.49E-03 |
| GOTERM_BP_FAT | GO:0010558~negative regulation of macromolecule biosynthetic process | 1.57E-03 |
| GOTERM_BP_FAT | GO:0031327~negative regulation of cellular biosynthetic process | 1.88E-03 |
| GOTERM_BP_FAT | GO:0009890~negative regulation of biosynthetic process | 2.20E-03 |
| GOTERM_BP_FAT | GO:0006357~regulation of transcription from RNA polymerase II promoter | 3.17E-03 |
| GOTERM_BP_FAT | GO:0045935~positive regulation of nucleobase, nucleoside, nucleotide and nucleic acid metabolic process | 3.34E-03 |
| GOTERM_BP_FAT | GO:0045944~positive regulation of transcription from RNA polymerase II promoter | 3.64E-03 |
| SP_PIR_KEYWORDS | nucleus | 5.02E-03 |
| GOTERM_MF_FAT | GO:0016564~transcription repressor activity | 8.89E-03 |
| GOTERM_BP_FAT | GO:0006350~transcription | 2.08E-02 |
| GOTERM_MF_FAT | GO:0043566~structure-specific DNA binding | 2.67E-02 |
| GOTERM_MF_FAT | GO:0003677~DNA binding | 5.99E-02 |
| SP_PIR_KEYWORDS | transcription regulation | 9.16E-02 |
| SP_PIR_KEYWORDS | Transcription | 1.26E-01 |
| GOTERM_MF_FAT | GO:0003700~transcription factor activity | 1.33E-01 |
| SP_PIR_KEYWORDS | activator | 1.77E-01 |
| SP_PIR_KEYWORDS | dna-binding | 3.16E-01 |
| GOTERM_MF_FAT | GO:0043565~sequence-specific DNA binding | 3.60E-01 |
| | | |
| | | |
| **Annotation Cluster 2** | **Enrichment Score: 2.458812727917162** | |
| | | |
| Category | Term | PValue |
| GOTERM_BP_FAT | GO:0042127~regulation of cell proliferation | 2.45E-03 |
| GOTERM_BP_FAT | GO:0008284~positive regulation of cell proliferation | 3.90E-03 |
| GOTERM_BP_FAT | GO:0050678~regulation of epithelial cell proliferation | 4.41E-03 |
